# Potential of Low Dose Leuco-Methylthioninium Bis(Hydromethanesulphonate) (LMTM) Monotherapy for Treatment of Mild Alzheimer’s Disease: Cohort Analysis as Modified Primary Outcome in a Phase III Clinical Trial

**DOI:** 10.3233/JAD-170560

**Published:** 2017-11-28

**Authors:** Gordon K. Wilcock, Serge Gauthier, Giovanni B. Frisoni, Jianping Jia, Jiri H. Hardlund, Hans J. Moebius, Peter Bentham, Karin A. Kook, Bjoern O. Schelter, Damon J. Wischik, Charles S. Davis, Roger T. Staff, Vesna Vuksanovic, Trevor Ahearn, Luc Bracoud, Kohkan Shamsi, Ken Marek, John Seibyl, Gernot Riedel, John M.D. Storey, Charles R. Harrington, Claude M. Wischik

**Affiliations:** a Nuffield Department of Clinical Neurosciences, University of Oxford, Oxford, UK; b McGill Centre for Studies in Aging, Alzheimer’s Disease Research Unit, and Douglas Mental Health University Institute, Montreal, QC, Canada; c University Hospitals and University of Geneva, Geneva, Switzerland; d Beijing Institute for Brain Disorders Alzheimer’s Disease Centre, Beijing, China; e TauRx Therapeutics, Aberdeen, UK; f Moebius-Consult, Baar, Switzerland; g Birmingham and Solihull Mental Health Foundation Trust, Birmingham, UK; h Salamandra LLC, Bethesda, MD, USA; i Institute for Complex Systems and Mathematical Biology, University of Aberdeen, Aberdeen, UK; j Computer Laboratory, University of Cambridge, Cambridge, UK; k CSD Biostatistics, Tucson, AZ, USA; l Aberdeen Biomedical Imaging Centre, School of Medicine, Medical Sciences and Nutrition, University of Aberdeen, Aberdeen, UK; m BioClinica, Lyon, France; n RadMD, New York, NY, USA; o MNI Imaging, New Haven, CT, USA; p School of Medicine, Medical Sciences and Nutrition, University of Aberdeen, Aberdeen, UK; q Department of Chemistry, University of Aberdeen, Aberdeen, UK

**Keywords:** ADAS-cog, Alzheimer’s disease, amyloid protein, clinical trial, cohort study, methylthioninium, tau protein, treatment

## Abstract

**Background:**

LMTM is being developed as a treatment for AD based on inhibition of tau aggregation.

**Objectives:**

To examine the efficacy of LMTM as monotherapy in non-randomized cohort analyses as modified primary outcomes in an 18-month Phase III trial in mild AD.

**Methods:**

Mild AD patients (*n* = 800) were randomly assigned to 100 mg twice a day or 4 mg twice a day. Prior to unblinding, the Statistical Analysis Plan was revised to compare the 100 mg twice a day as monotherapy subgroup (*n* = 79) versus 4 mg twice a day as randomized (*n* = 396), and 4 mg twice a day as monotherapy (*n* = 76) versus 4 mg twice a day as add-on therapy (*n* = 297), with strong control of family-wise type I error.

**Results:**

The revised analyses were statistically significant at the required threshold of *p* < 0.025 in both comparisons for change in ADAS-cog, ADCS-ADL, MRI atrophy, and glucose uptake. The brain atrophy rate was initially typical of mild AD in both add-on and monotherapy groups, but after 9 months of treatment, the rate in monotherapy patients declined significantly to that reported for normal elderly controls. Differences in severity or diagnosis at baseline between monotherapy and add-on patients did not account for significant differences in favor of monotherapy.

**Conclusions:**

The results are consistent with earlier studies in supporting the hypothesis that LMTM might be effective as monotherapy and that 4 mg twice a day may serve as well as higher doses. A further suitably randomized trial is required to test this hypothesis.

## INTRODUCTION

In Alzheimer’s disease (AD), clinical deterioration [1], imaging markers of loss of neuronal function [2–4], and progression of brain atrophy as measured by MRI volumetry [5] progress in parallel with the accumulation of aggregated tau. Proteolytically stable aggregates of tau protein can be measured in the neocortex in the prodromal phase of the disease from Braak stage 2 onwards [6–8], which occurs at least 20 years before neurofibrillary pathology is seen in neocortex or dementia symptoms appear [9], and the levels increase exponentially as the disease progresses [8]. Tau protein has the capacity to form toxic proteolytically resistant oligomers which seed further tau aggregation in an autocatalytic manner [10] and propagate the pathology into previously healthy brain regions [11], most likely accounting for the stereotyped pattern of spread of pathology [6, 7, 12]. Targeting tau protein aggregation therefore offers an attractive therapeutic possibility as a disease modifying treatment of AD.

A Phase II placebo-controlled clinical trial tested the potential utility of the methylthioninium moiety (MT) as monotherapy in mild to moderate AD in patients not taking cholinesterase inhibitors or memantine. This study, using the oxidized form of MT (as methylthioninium chloride, MTC) as monotherapy, showed that MT at a dose of 138 mg/day produced a significant treatment effect on the Alzheimer’s Disease Assessment Scale–cognitive subscale (ADAS-cog) at 24 weeks compared with placebo, supported by evidence of benefit on the functional neuroimaging outcomes(hexa-methyl-propyl-amine-oxime single photon emission computed tomography (HMPAO-SPECT) [13] and ^18^F-fluorodeoxyglucose-positron emission tomography (^18^F-FDG-PET) [14]). A newly developed form of the MT moiety, as leuco-methylthioninium bis(hydromethanesulphonate) (LMTM), is much better absorbed than the oxidized MTC tested previously [13, 15] and has been taken forward for Phase III clinical trials. As the dihydromethanesulfonate salt, LMTM stabilizes the reduced form of the MT moiety in the solid state. Following absorption, the dissociated MT moiety is distributed and excreted in an equilibrium between oxidized and reduced forms that depends on the local pH and redox environment.

The as-randomised analysis of an earlier Phase III trial (TRx237-015) has been reported [16]. Both studies were designed to compare higher doses of LMTM in the range 150–250 mg/day with a low dose of 4 mg twice a day intended as a urinary discolorant to maintain blinding. It was assumed that this low dose would be ineffective, since a dose of 69 mg MT/day as MTC was found to have reduced efficacy in the Phase II study [13]. Neither of the Phase III studies showed any difference on primary or secondary outcomes between the high doses and 4 mg twice a day in the as-randomized comparisons. In the first study, treatment status with cholinesterase inhibitors and/or memantine was found to be a significant covariate in the primary analysis model [16]. Exploratory analyses showed that this was due to significantly lower rates of progression on clinical and brain atrophy endpoints in patients receiving any of the LMTM doses as monotherapy, including 4 mg twice a day, which did not appear to be explicable by cohort differences in severity at baseline.

The results of study TRx-237-015, which became available prior to database lock and unblinding of the present study, suggested the hypothesis that LMTM might be effective only as monotherapy and that the minimum effective dose might be substantially lower for LMTM than that previously identified using MTC [13, 15]. As the originally intended analysis was unlikely to achieve its intended purpose, we modified the primary analyses and treatment comparisons in the TRx-237-005 Statistical Analysis Plan prior to database lock and unblinding to investigate whether the monotherapy differences could be confirmed as observational cohort comparisons defined as primary outcomes with strong control of family-wise type I error in the second independent study. The monotherapy cohort comparisons which were of particular interest in light of the earlier study were: (A) 100 mg twice a day a monotherapy compared with 4 mg twice a day as originally randomized, and (B) 4 mg twice a day as monotherapy compared with 4 mg twice a day as add-on to standard AD treatments. Each of these comparisons was required to reach a statistical threshold of 0.025 on both cognitive (ADAS-cog) and functional (ADCS-ADL) outcomes for the analysis to meet the modified primary statisticalendpoints.

## MATERIALS AND METHODS

### Study design and participants, randomization and masking, and outcomes

The study was designed as an 18-months phase III, randomized, controlled, double-blind, parallel-group trial conducted at 108 sites in Canada, United States, Australia, and Europe. Eligible patients had to be younger than 90 years with a diagnosis of probable AD according to criteria from the National Institute of Aging and the Alzheimer’s Association, with mild severity defined by Mini-Mental State Examination (MMSE) score of 20–26 inclusive and a Clinical Dementia Rating (CDR) total score of 0.5 or 1.0. Concomitant use of acetylcholinesterase inhibitors or memantine (or both) was permitted provided this was at a stable dose for at least 18 weeks before randomization to minimize the initial symptomatic effects of these treatments. Concomitant use of serotonergicantidepressant, antipsychotic (except clozapine or olanzapine), and sedative medications was also permitted at stable doses where clinically feasible. Each patient had one or more study partners participate with them in the trial. Patients were excluded from the study if they had a significant CNS disorder other than AD. A detailed list of inclusion and exclusion criteria is in the protocol provided in the Supplementary Materials.

Patients were randomly assigned to receive LMTM 100 mg twice a day (expressed as methylthioninium base equivalent) or LMTM 4 mg twice a day. The low dose was selected as a control to permit masking for potential urinary discoloration and was assumed to be inactive in light of the earlier Phase II study using MTC [13]. The randomization was stratified according to geographical region (two levels: North America or Europe/Australia), use of AD-labeled comedications (two levels: using or not using), and severity of AD (two levels: CDR 0.5 or CDR 1.0). The randomization file and investigational medicinal product kit list were unavailable to personnel involved in conducting the study and analyzing the data. Study participants, their informant(s), and all assessors remained masked to treatment assignment throughout the study, and safety assessors were not permitted to be involved in the primary efficacyassessments.

The two doses were provided in identical blister packages as identically appearing oral tablets to be taken for up to 78 weeks. The co-primary outcomes were the 11-item Alzheimer’s Disease Assessment Scale–cognitive subscale (ADAS-cog) and the 23-item Alzheimer’s Disease Co-operative Study–Activities of Daily Living (ADCS-ADL) scale measured at baseline and every 13 weeks thereafter, with the final on-treatment visit at week 78, and a final off-treatment safety assessment at week 82. Other outcomes included the Alzheimer’s Disease Cooperative Study–Clinical Global Impression of Change scale (ADCS-CGIC), administered by an independent rater at the same visits as ADAS-cog and ADCS-ADL, and MMSE, Neuropsychiatric Inventory (NPI), and Montgomery-*Å*sberg Rating Scale (MADRS, administered at screening and weeks 26, 52, and 78 with MMSE again at week 82). MRI scans were undertaken at baseline or screening and at weeks 13, 26, 39, 52, 65, and 78 (or at early termination) using a standardized protocol at all sites and volumetric analyses were performed by a central imaging core laboratory (BioClinica). Volumetric data were used to measure changes in lateral ventricular volume, whole brain volume, and estimated mean of left and right for temporoparietal and hippocampal volumes. ^18^F-fluorodeoxyglucose PET (^18^F-FDG-PET) imaging was done at screening and weeks 39 and 78. Changes in cerebrospinal fluid (CSF) total tau, phospho-tau, and amyloid-β_1–42_ between baseline (any time during screening before first dose of study drug) and week 78 (or early termination) were measured in a subsample of patients who consented to a lumbar puncture. Resource Utilization in Dementia (RUD)-lite instrument score was also determined but is not reported at this time.

Patients were monitored throughout the study for adverse events using clinical laboratory tests (including measurement of methaemoglobin by pulse CO-oximetry), physical and neurological examinations, and 12-lead electrocardiograms (ECG) at all clinic visits (screening, baseline, and weeks 2, 6, 13, 26, 39, 52, 65, 78, and 82). Patients were also assessed at all visits for suicidal ideation and intent using the Columbia-Suicide Severity Rating Scale [17], and were systematically monitored for potential serotonin syndrome using a rating scale derived from four published diagnostic criteria [18], because of a theoretical potential for serotonin syndrome [19]. By protocol, amyloid related imaging abnormalities, serotonin toxicity, and suicidality were reported as serious adverse events.

### Statistical analysis

*Post-hoc* analyses of the earlier trial in mild to moderate AD trial (TRx-237-015 [16]) suggested the hypothesis that LMTM might be effective only as monotherapy. We therefore revised the originally intended primary analysis prior to database lock and data unblinding to examine whether differences in favor of monotherapy could be confirmed as primary outcomes in non-randomized observational cohort comparisons based on the use of a two-sided test with an *α* of 0.025 to control family-wise Type I error. The final Statistical Analysis Plan is provided in Supplementary Materials. The primary statistical tests were (Comparison A) comparison of patients receiving 100 mg twice a day as monotherapy with the control arm as randomized (all patients receiving 4 mg twice a day regardless of AD comedication status with cholinesterase inhibitors and/or memantine), and (Comparison B) comparison of patients receiving 4 mg twice a day as monotherapy with those receiving 4 mg twice a day as add-on to approved AD-labelled treatments. Exploratory analyses included comparison of patients receiving 100 mg twice a day as monotherapy with those receiving 100 mg twice a day as add-on to approved AD-labelled treatments (Comparison C), and comparisons according to co-medication status with approved AD treatments in patients receiving either dose of LMTM as pooled subgroups.

The primary analyses were performed in the modified intent-to-treat (mITT) population defined to include all randomized subjects who took at least one dose of the study drug and had both a baseline and at least one post-baseline non-follow-up efficacy assessment. The primary analyses were specified as a mixed model, repeated-measures analysis with an unstructured covariance matrix and no imputation for missing data. The model included visit (six levels corresponding to assessments at weeks 13, 26, 39, 52, 65, and 78), treatment group (two levels, 4 mg or 100 mg twice a day), baseline severity (CDR, two levels), geographic region (two levels: US/Canada, Europe/Australia) and baseline ADAS-cog or ADCS-ADL score. Alzheimer’s-comedication status at baseline (two levels: current ongoing use or not ongoing use) was included in the model as the interaction terms: *status x treatment* and *status x visit*. The individual tests were implemented through contrasts. We used the same method for all secondary analyses in predefined gated sequences such that no further adjustment of *α* (0.025) was needed. Further sensitivity analyses were undertaken to determine whether baseline differences could account for differences in rate of progression by addition of a further term (*baseline-variablex visit*) to the primary analysis model. Characteristics tested included baseline score of the outcome of interest, APOE *ɛ*4 frequency, vascular pathology burden (Fazekas score estimating the lesion load in the brain), hippocampal atrophy, temporoparietal volume and temporal lobe SUVR determined by ^18^F-FDG-PET. This rate-correction term was added as either a continuous or categorical variable as appropriate.

The new Statistical Analysis Plan substituted the primary monotherapy subgroup comparisons (Comparisons A and B) in an intended exploratory two time-point analysis to compare whether the effect sizes on the co-primary outcomes were larger at 18 months than at 9 months to support a treatment effect on slope. Another prespecified exploratory analysis investigated the primary and secondary outcomes in CDR 0.5 and CDR 1.0 subgroups separately according to the primary subgroup comparisons (Comparisons A and B) and Comparison C as further exploratory analysis. Finally, exploratory contrasts comparing treatment subgroups according to dose and AD comedication status were performed.

Exploratory *post-hoc* analyses were conducted to compare within-cohort annualized rates of whole brain atrophy in patients receiving LMTM as monotherapy and as add-on initially and after 9 months of treatment using the same mixed model except with time as a continuous variable. The annualized rates output from this analysis for months 0–6 and months 12–18 respectively were compared with the rates for mild AD and normal aging reported from the Alzheimer’s Disease Neuroimaging Initiative (ADNI) in years 1 and 2 using *t*-tests [20]. Decline on the ADAS-cog scale in patients receiving LMTM as monotherapy or as add-on therapy was also compared with that reported for mild AD in the placebo arms of recent Phase III studies [21, 22] and currently available ADNI data (https://ida.loni.usc.edu/collaboration/access/appLicense.jsp). Voxel-based morphometry was used to compare monotherapy patients at baseline with elderly controls in a well-characterized ongoing birth-cohort study [23] using a statistical parametric mapping package in analyses controlled for age, sex and total intracranial volume. Inferior temporal gyrus ^18^F-FDG-PET SUVR normalized with respect to pons was determined at baseline and compared with the values reported for mild AD, MCI, and normal elderly controls using the same methodology [24]. Frontal, parietal, and temporal lobe ^18^F-FDG-PET SUVR data normalized with respect to pons and cerebellum were also analyzed. Coordinates permitting determination of nucleus basalis volume were kindly provided by Ingo Kilimann [25].

Safety analyses were based on the safety population comprising all patients who received at least one dose of study drug, with summaries presented according to dose and AD co-medication status.

Data analyses specified in the Statistical Analysis Plan were undertaken independently of the funder by SynteractHCR (Carlsbad, CA, USA) using SAS 9.4 (Enterprise Guide v7.1). The results were verified and additional exploratory analyses were provided by one of the co-authors (BOS) using R version 3.3.0 (2016-05-03). Additional voxel-based morphometric analyses were provided by VV, TA, and RTS using the Statistical Parametric Mapping (SPM12) software package (http://www.fil.ion.ucl.ac.uk/spm/). This trial is registered at http://www.clinicaltrials.gov (NCT01689233) and the European Union Clinical Trials Registry (21012-002847-28).

### Role of the funding source

The funder of the study took the lead in study design, undertaking the study, data interpretation, and initial drafting of the report.

## RESULTS

**Fig.1:**
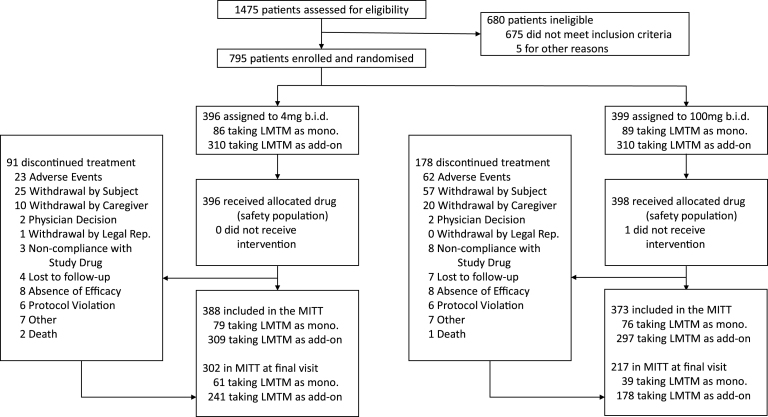
Trial profile.

The baseline demographic and clinical characteristics of the mITT population are presented in Table 1 and the trial profile is presented in Fig. 1 according to dose and treatment status with cholinesterase inhibitors and/or memantine. 419 of 606 (69%) patients taking LMTM in combination with cholinesterase inhibitors and/or memantine completed the study. Of those not receiving standard AD treatments, 100 of 155 (65%) completed the study. The retention of patients receiving 100 mg twice a day (217/373, 58%) was substantially lower than for patients receiving 4 mg twice a day (302/388, 78%). ^18^F-FDG-PET data were available for 759 patients at baseline. Of these, 605 and 154 were randomized to receive LMTM as add-on to standard treatments or as monotherapy, and 389 and 83, respectively, had scans available at 78 weeks. Lumbar puncture data were available for 255 patients at baseline, of whom 174 and 81, respectively, received LMTM as add-on treatment or monotherapy, with 66 and 15, respectively, available at the end of the study.

**Table 1:** Baseline characteristics of mITT population

Characteristic	LMTM 4 mg twice a day as add-on (*n* = 309)	LMTM 100 mg twice a day as add-on (*n* = 297)	LMTM 4 mg twice a day as monotherapy (*n* = 79)	LMTM 100 mg twice a day as monotherapy (*n* = 76)
Age (years)
Mean (SD)	71.1 (8.8)	70.6 (8.7)	68.4 (9.8)	69.1 (9.7)
Median (IQR)	72.0 (65,78)	71.0 (66,76)	70.0 (61,77)	68.5 (62,78)
Sex
Male, *n* (%)	154 (50)	146 (49)	31 (39)	29 (38)
Female, *n* (%)y	155 (50)	151 (51)	48 (61)	47 (62)
Race
Amer. Indian or Alaska Native, *n* (%)	2(<1%)	5 (2%)	1 (1%)	1 (1%)
Asian, *n* (%)	2(<1%)	3 (1%)	0	1 (1%)
Black or African American, n (%)	10 (4%)	5 (2%)	5 (6%)	4 (5%)
White, *n* (%)	287 (93%)	273 (92%)	70 (89%)	63 (83%)
Other, *n* (%)	4 (1%)	4 (1%)	1 (1%)	4 (5%)
Mixed Race, *n* (%)	1(<1%)	4 (1%)	0	0
Years since diagnosis
Mean (SD)	2.7 (2.2)	2.3 (2.1)	1.5 (1.6)	2.0 (2.0)
Median (IQR)	2.3 (1.0,3.8)	1.8 (0.7,3.1)	0.8 (0.3,2.3)	1.4 (0.4,3.4)
Dementia severity
CDR 0.5, *n* (%)	180 (58%)	177 (60%)	63 (80%)	59 (78%)
CDR 1, *n* (%)	129 (42%)	120 (40%)	16 (20%)	17 (22%)
MMSE
Mean (SD)	22.9 (2.0)	22.9 (2.0)	23.5 (1.9)	23.6 (1.9)
Median (IQR)	23.0 (21,25)	23.0 (21,26)	23.0 (22,25)	24.0 (22,25)
ADAS-Cog:
Mean (SD)	18.0 (7.1)	18.4 (6.9)	15.4 (7.5)	14.5 (6.0)
Median (IQR)	17.0 (13,22)	17.3 (14,22)	14.0 (10,20)	13.3 (11,18)
ADCS-ADL:
Mean (SD)	66.8 (8.0)	66.4 (7.9)	70.0 (6.0)	69.5 (6.2)
Median (IQR)	68.0 (63,73)	69.0 (63,72)	71.0 (67,74)	71.0 (66,74)
Whole brain volume (cm^3^)
Mean (SD)	972 (118)	971 (111)	973 (108)	962 (97)
Median (IQR)	973 (890,1051)	964 (894,1035)	976 (895,1030)	954 (895,1020)
Lateral ventricular volume (cm^3^)
Mean (SD)	54 (25)	50 (23)	41 (22)	40 (22)
Median (IQR)	49 (34,68)	46 (34,63)	38 (23,58)	35 (23,54)
Hippocampal volume (cm^3^)
Mean (SD)	6.0 (1.2)	5.9 (1.2)	6.6 (1.2)	6.4 (1.1)
Median (IQR)	6.0 (5.1,6.7)	5.8 (5.0,6.7)	6.5 (5.8,7.6)	6.3 (5.6,7.2)
Temporal lobe ^18^F-FDG-PET (SUVR)
Mean (SD)	1.18 (0.12)	1.18 (0.11)	1.21 (0.13)	1.23 (0.11)
AD-approved co-medications
AChEI only, *n* (%)	185 (60%)	187 (63%)	0	2 (3%)
Memantine only, *n* (%)	25 (8%)	19 (6%)	0	1 (1%)
AChEI and memantine, *n* (%)	94 (30%)	88 (30%)	2 (3%)	0
CSF biomarkers (ng/L)
Total tau, mean (SD) [*n*]	108.2 (65.2) [67]	106.5 (52.0) [64]	72.5 (31.9) [22]	131.2 (75.5) [14]
Phospho-tau, mean (SD) [*n*]	46.1 (22.6) [68]	43.6 (24.4) [68]	33.1 (16.5) [21]	46.4 (24.6) [14]
Aβ_1 - 42_, mean (SD) [*n*]	295.2 (111.0) [69]	271.5 (107.1) [67]	340.2 (126.7) [22]	387.0 (123.0) [14]
*APOE* genotype
*ɛ*4 allele present, *n* (%)	164 (61%)	156 (63%)	35 (49%)	33 (47%)
*ɛ*4 allele absent, *n* (%)	107 (39%)	91 (37%)	36 (51%)	38 (54%)

### Clinical efficacy outcomes

**Fig.2:**
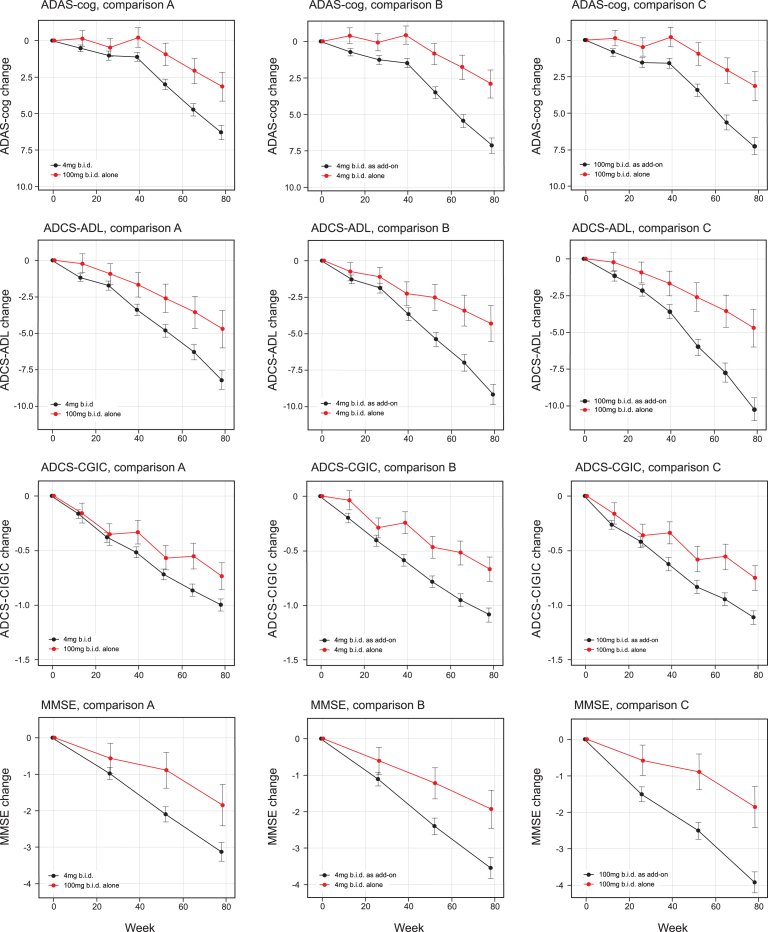
Least squares estimates of mean change from baseline and S.E. on primary and principal secondary outcomes. Primary analyses as defined in the revised Statistical Analysis Plan compared 100 mg twice a day as monotherapy with all patients receiving 4 mg twice a day (control as randomly assigned; comparison A) and 4 mg twice a day as monotherapy with same dose as add-on to approved treatment for AD (comparison B). Comparison C, 100 mg twice a day as monotherapy with same dose as add-on to approved treatment for AD, is also shown.

The treatment comparisons for the co-primary outcomes as defined in the Statistical Analysis Plan finalized prior to database lock were significant at the *α* threshold of 0.025 for 100 mg twice a day as monotherapy compared with all patients receiving 4 mg twice a day as randomly assigned (ADAS-cog effect size –3.14, 95% CI –5.32 to –0.97, *p* = 0.0047; ADCS-ADL effect size 3·49, 95% CI 0.66 to 6.30, *p* = 0.0157 [comparison A, Table 2a, Fig. 2]). The treatment comparisons for the co-primary outcomes were also significant at the *α* threshold of 0.025 for 4 mg twice a day as monotherapy compared with 4 mg twice a day as add-on to approved AD treatments (ADAS-cog effect size –4·22, 95% CI –6.19 to –2.24, *p* < 0.0001; ADCS-ADL effect size 4.85, 95% CI 2.31 to 7.40, *p* = 0.0002 [Comparison B, Table 2a, Fig. 2]). A similar comparison between 100 mg twice a day as monotherapy and as add-on therapy produced similar results (ADAS-cog effect size –4·08, 95% CI –6.07 to –2.08, *p* = 0.0001; ADCS-ADL effect size 5.27, 95% CI 2.70 to 7.81, *p* = 0.0001 [Comparison C, Table 2b, Fig. 2]). There was no difference between 4 mg and 100 mg twice a day as monotherapy in the corresponding monotherapy versus add-on therapy treatment comparisons.

**Table 2a:** Primary and secondary clinical outcomes according to the revised statistical analysis plan to examine LMTM 100 mg twice a day as monotherapy compared with the control arm as randomized (Comparison A), and LMTM 4 mg twice a day as monotherapy compared with 4 mg twice a day as add-on to existing AD treatments (Comparison B)

	Comparison A				Comparison B			
	Baseline	Change from baseline for 4 mg twice a day, as randomized (*n* = 388)	Difference for 100 mg twice day, as monotherapy (*n* = 76)	*p* value	Baseline	Change from baseline for 4 mg twice a day, as add-on (*n* = 309)	Difference for 4 mg twice a day, as monotherapy (*n* = 79)	*p* value
ADAS-cog
Mean	16.97	6.30	–3.14	0.0047	17.45	7.13	–4.22	<0.0001
95% CI	16.32, 17.62	5.34, 7.27	–5.32, –0.97		16.73, 18.17	6.09, 8.18	–6.19,–2.24
ADCS-ADL
Mean	67.75	–8.21	3.49	0.0157	67.40	–9.17	4.85	0.0002
95% CI	67.06, 68.44	–9.46, –6.95	0.66, 6.30		66.63, 68.17	–10.52, –7.82	2.31, 7.40
CGIC
Mean		–1.00	0.27	0.0521		–1.09	0.42	0.0007
95% CI		–1.11, –0.89	–0.00, 0.53			–1.21, –0.96	0.17, 0.66
MMSE
Mean	23.15	–3.22	1.37	0.0289	23.05	–3.54	1.60	0.0045
95% CI	22.67, 23.63	–3.72, –2.71	–1.76, 1.00		22.85, 23.25	–4.09, –2.98	0.50, 2.71
MADRS
Mean	4.90	0.19	–0.38	0.5880	4.81	0.35	–0.80	0.1976
95% CI	4.48, 5.32	–0.35, 0.73	0.14, 2.60		4.35, 5.27	–0.25, 0.94	2.02, 0.42
NPI (total)
Mean	8.15	1.79	0.26	0.8659	7.93	2.17	–1.89	0.1732
95% CI	7.31, 8.99	0.56, 3.02	–2.81, 3.34		7.01, 8.85	0.83, 3.52	4.61, 0.83
NPI (carer distress)
Mean	4.75	0.91	0.31	0.7240	4.70	1.22	–1.54	0.0455
95% CI	4.20, 5.30	0.22, 1.61	–2.02, 1.40		4.09, 5.31	0.46, 1.98	3.05, 0.03

Data expressed as mean with 95% CI.

**Table 2b:** Primary and secondary clinical outcomes comparing LMTM 100 mg twice a day as monotherapy with 100 mg twice a day as add-on to existing AD treatments (Comparison C)

	Comparison C			
	Baseline	Change from baseline for 100 mg twice a day, as add-on (*n* = 297)	Difference for 100 twice a day, as monotherapy (*n* = 76)	*p* value
ADAS-cog
Mean	17.57	7.24	–4.08	0.0001
95% CI	16.87, 18.27	6.08, 8.40	–6.07 –2.08
ADCS-ADL
Mean	67.02	–9.99	5.27	0.0001
95% CI	66.24, 67.80	–11.50, –8.49	2.70, 7.84
CGIC
Mean		–1.10	0.36	0.0053
95% CI		–1.24, –0.96	0.11, 0.62
MMSE
Mean	23.02	–3.90	2.05	0.0007
95% CI	22.81, 23.23	–4.52, –3.28	0.87, 3.23
MADRS
Mean	5.46	0.19	–0.38	0.5880
95% CI	4.95, 5.97	–0.35, 0.73	0.14, 2.60
NPI (total)
Mean	8.67	3.45	–1.39	0.3597
95% CI	7.70, 9.64	1.91, 4.98	–4.36, 1.58
NPI (carer distress)
Mean		2.21	–1.60	0.0554
95% CI		1.34, 3.07	–3.24, 0.04

Data expressed as mean with 95% CI.

The secondary clinical outcomes are also shown in Table 2a according to the two testing sequences for Comparisons A and B and in Table 2b for Comparison C. The results are shown in Fig. 2. Further testing in the Comparison A sequence did not reach the required level of significance for ADCS-CGIC (*p* = 0.0521) or MMSE (*p* = 0.0289). In the Comparison B sequence, the 4 mg twice a day monotherapy dose comparison with add-on therapy was significant at the required level for ADCS-CGIC (*p* = 0.0007) and MMSE (*p* = 0.0045). In the Comparison C sequence, the 100 mg twice a day monotherapy dose comparison with add-on therapy was significant for ADCS-CGIC (*p* = 0.0053) and MMSE (*p* = 0.0007). The treatment effects for MADRS and the total NPI score were not significant in any of Comparisons A, B, or  C, although a directionally supportive difference in favor of monotherapy was seen for 4 mg twice a day on the NPI carer distress scale (*p* = 0.0455).

The decline on the ADAS-cog scale seen in the subgroups receiving LMTM as add-on was indistinguishable from the mean placebo decline reported in two recent Phase III studies ([21, 22]; Supplementary Figure 1). Exclusion of patients prescribed memantine and a cholinesterase inhibitor in combination had minimal effect on the differences seen in favor of LMTM as monotherapy (Supplementary Table 2a, b). In order to test whether baseline differences in severity or other factors could account for the significant differences seen in favor of LMTM as monotherapy, the primary analysis model was expanded to include baseline severity, APOE *ɛ*4 frequency, vascular pathology rating on MRI, hippocampal volume, temporoparietal volume, or baseline temporal lobe ^18^F-FDG-PET SUVR as rate-correction terms to correct for a possible effect on rate of progression. Comparisons A, B, and C for ADAS-cog, ADCS-ADL, and ventricular volume all remained significant after applying this correction (Table 3a, b).

**Table 3a:** Primary analysis model augmented to include an additional *rate correction* term as *baseline-value x visit* to determine whether baseline differences in severity or other characteristics account for differences seen for comparisons A and B at week 78 in primary and principal secondary outcomes. The baseline-value was either continuous or categorical depending on the nature of the variable

			Comparison A			Comparison B		
	Baseline value used as rate correction term		4 mg twice a day as randomized (*n* = 388)	Difference for 100 mg twice a day as monotherapy (*n* = 76)	*p* value	4 mg twice a day as add-on (n-309)	Difference for 4 mg twice a day as monotherapy (*n* = 79)	*p* value
ADAS-cog	ADAS-cog	Mean	6.45	–2.19	0.0356	7.02	–2.85	0.0028
		95% CI	5.56, 7.35	–4.23, –0.15		6.05, 7.99	–4.72, –0.98
	*APOE* *ɛ*4	Mean	6.33	–3.49	0.0028	7.16	–4.06	0.0001
		95% CI	5.31, 7.36	–5.77, –1.20		6.05, 8.27	–6.15, –1.97
	Vascular burden	Mean	6.29	–3.12	0.0051	7.11	–4.14	<0.0001
		95% CI	5.32, 7.26	–5.30, –0.93		6.05, 8.15	–6.12, –2.16
	Hippocampal volume	Mean	6.35	–2.93	0.0085	7.12	–3.88	0.0001
		95% CI	5.38, 7.32	–5.12, –0.75		6.07, 8.17	–5.87, –1.88
	Temporo-parietal volume	Mean	6.32	–2.75	0.0111	7.08	–3.84	0.0001
		95% CI	5.38, 7.25	–4.87, –0.63		6.07, 8.10	–5.77, –1.91
	Temporal lobe ^18^F-FDG-PET SUVR	Mean	6.27	–2.24	0.0389	6.89	–3.14	0.0015
		95% CI	5.34, 7.20	–4.37, –0.11		5.88, 7.90	–5.08, –1.20
ADCS-ADL	ADCS-ADL	Mean	–8.36	2.84	0.0474	–9.15	3.99	0.0021
		95% CI	–9.60, –7.12	0.03, 5.64		–10.49, –7.82	1.45, 6.54
	*APOE* *ɛ*4	Mean	–8.25	4.09	0.0062	–9.36	4.96	0.0003
		95% CI	–9.66, –7.03	1.16, 7.03		–10.78, –7.94	2.30, 7.62
	Vascular burden	Mean	–8.19	3.51	0.0150	–9.15	4.83	0.0002
		95% CI	–9.45, –6.94	0.68, 6.34		–10.50, –7.80	2.28, 7.38
	Hippocampal volume	Mean	–8.23	2.92	0.0426	–9.06	4.16	0.0014
		95% CI	–9.48, –6.98	0.10, 5.73		–10.41, –7.71	1.60, 6.71
	Temporo-parietal volume	Mean	–8.25	2.87	0.0429	–9.11	4.32	0.0007
		95% CI	–9.48, –7.02	0.09, 5.65		–10.44, –7.78	1.81, 6.82
	Temporal lobe ^18^F-FDG-PET SUVR	Mean	–8.20	2.71	0.0581	–8.97	3.87	0.0028
		95% CI	–9.42, –6.97	–0.09, 5.51		–10.29, –7.64	1.33, 6.41
LVV	LVV	Mean	7.25	–1.46	0.0118	7.63	–1.78	0.0006
		95% CI	6.73, 7.76	–2.59, –0.32		7.07, 8.19	–2.79, –0.76
	*APOE* *ɛ*4	Mean	7.22	–2.79	<0.0001	7.93	–3.07	<0.0001
		95% CI	6.62, 7.83	–4.09, –1.48		7.27, 8.59	–4.24, –1.90
	Vascular burden	Mean	7.35	–2.82	<0.0001	8.02	–3.09	<0.0001
		95% CI	6.76, 7.95	–4.11, –1.53		7.38, 8.66	–4.24, –1.94
	Hippocampal volume	Mean	7.36	–2.61	0.0001	7.63	–1.78	0.0006
		95% CI	6.77, 7.96	–3.90, –1.32		7.07, 8.19	–2.79, –0.76
	Temporo-parietal volume	Mean	7.36	–2.70	<0.0001	8.00	–2.92	<0.0001
		95% CI	6.77, 7.96	–3.99, –1.42		7.36, 8.64	–4.07, –1.77
	Temporal lobe ^18^F-FDG-PET SUVR	Mean	7.36	–2.07	0.0010	7.82	–2.13	0.0002
		95% CI	6.79, 7.92	–3.30, –0.84		7.21, 8.43	–3.24, –1.02

**Table 3b:** Revised primary analysis model augmented to include an additional *rate correction* term as *baseline-value x visit* to determine whether baseline differences in severity or other characteristics account for differences seen for Comparison C at week 78 in primary and principal secondary outcomes. The baseline-value was either continuous or categorical depending on the nature of the variable

			Comparison C		
	Baseline value used as rate correction term		100 mg twice a day as add-on (*n* = 297)	Difference for 100 mg twice a day as monotherapy (*n* = 76)	*p* value
ADAS-cog	ADAS-cog	Mean	6.93	–2.66	0.0058
		95% CI	5.85, 8.01	–4.55, –0.77
	*APOE* *ɛ*4	Mean	6.83	–3.98	0.0002
		95% CI	5.56, 8.10	–6.08, –1.88
	Vascular burden	Mean	7.23	–4.06	0.0001
		95% CI	6.07, 8.39	–6.06, –2.06
	Hippocampal volume	Mean	7.15	–3.73	0.0003
		95% CI	5.99, 8.31	–5.74, –1.72
	Temporo-parietal volume	Mean	7.21	–3.64	0.0002
		95% CI	6.08, 8.33	–5.59, –1.69
	Temporal lobe ^18^F-FDG-PET SUVR	Mean	6.95	–2.92	0.0036
		95% CI	5.83, 8.07	–4.89, –0.96
ADCS-ADL	ADCS-ADL	Mean	–9.96	4.44	0.0007
		95% CI	–11.45, –8.47	1.87, 7.00
	*APOE* *ɛ*4	Mean	–9.54	5.29	0.0001
		95% CI	–11.17, –7.90	2.61, 7.96
	Vascular burden	Mean	–9.97	5.29	0.0001
		95% CI	–11.48, –8.46	2.72, 7.87
	Hippocampal volume	Mean	–9.85	4.53	0.0006
		95% CI	–11.34, –8.35	1.95, 7.11
	Temporo-parietal volume	Mean	–9.37	4.56	0.0004
		95% CI	–11.41, –8.45	2.02, 7.09
	Temporal lobe ^18^F-FDG-PET SUVR	Mean	–9.68	4.19	0.0014
		95% CI	–11.16, –8.20	1.62, 6.77
LVV	LVV	Mean	7.72	–1.93	0.0002
		95% CI	7.10, 8.33	–2.94, –0.92
	*APOE* *ɛ*4	Mean	7.41	–2.97	<0.0001
		95% CI	6.66, 8.16	–4.13, –1.81
	Vascular burden	Mean	7.74	–3.21	<0.0001
		95% CI	7.03, 8.46	–4.35, –2.07
	Hippocampal volume	Mean	7.69	–2.94	<0.0001
		95% CI	6.98, 8.40	–4.09, –1.79
	Temporo-parietal volume	Mean	7.71	–3.05	<0.0001
		95% CI	7.00, 8.42	–4.19, –1.91
	Temporal lobe ^18^F-FDG-PET SUVR	Mean	7.53	–2.25	0.0001
		95% CI	6.86, 8.21	–3.36, –1.14

Comparisons A, B and C were examined in patients with CDR 0.5 or CDR 1.0 as separate subgroups (Supplementary Table 3a, b). For the 4 mg twice a day dose, the treatment differences in favor of LMTM monotherapy compared with add-on (Comparison B) were significant at the *p* < 0.025 threshold for ADAS-cog, ADCS-ADL, and ADCS-CGIC in both CDR severity subgroups. For the corresponding comparison at the 100 mg twice a day dose (Comparison C), ADAS-cog and ADCS-ADL were both significant at the *p* < 0.025 threshold only in the CDR 0.5 subgroup, but not in Comparison A at either severity level. The treatment differences in favor of LMTM monotherapy were found to increase significantly over time when comparing treatment effects at 9 and 18 months according to Comparisons B and C, and in Comparison A only for ADAS-cog (Supplementary Table 4).

### MRI, ^18^F-FDG-PET, and CSF outcomes

**Fig.3:**
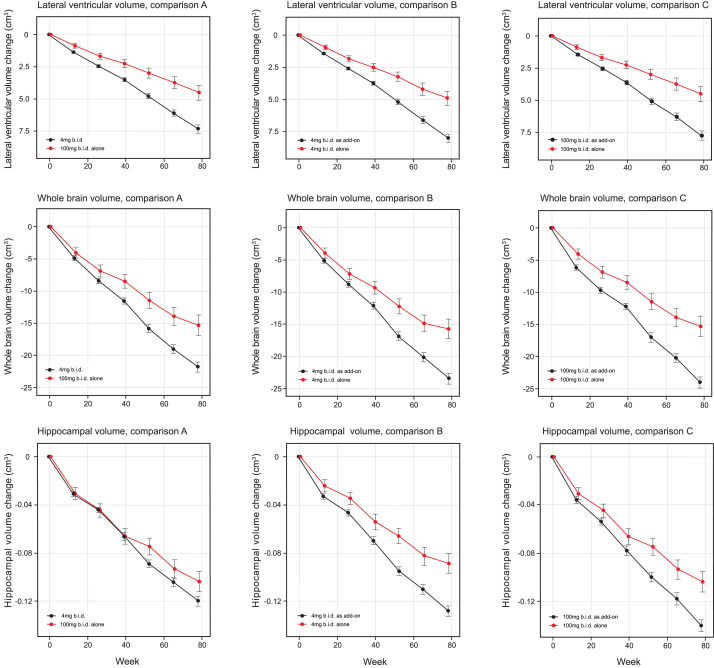
Least squares estimates of mean change from baseline and S.E. on MRI volumetric outcomes: lateral ventricular volume, whole brain volume and hippocampal volume. Comparisons shown are 100 mg twice a day as monotherapy with all patients receiving 4 mg twice a day (control as randomly assigned, Comparison A) and 4 mg twice a day as monotherapy with same dose as add-on to approved treatment for AD (Comparison B) and 100 mg twice a day as monotherapy with same dose as add-on to approved treatment for AD(Comparison C).

MRI volumetric outcomes for lateral ventricular volume, whole brain volume, and hippocampal volume were analyzed according to Comparisons A, B, and C (Table 4a and b, Fig. 3). All comparisons were significantly in favor of LMTM monotherapy at *p*≤0.0002 except hippocampal volume in Comparison A. Similar comparisons in patients with CDR 0.5 or CDR 1.0 as separate subgroups (Supplementary Table 5a, b) showed that the treatment differences in favor of LMTM monotherapy were more consistent for the 4 mg twice a day dose than for the 100 mg twice a day dose at both severity levels.

**Table 4a:** Volumetric MRI outcomes according to the revised statistical analysis plan to examine LMTM 100 mg twice a day as monotherapy compared with the control arm as randomized (Comparison A), and LMTM 4 mg twice a day as monotherapy compared with 4 mg twice a day as add-on to existing AD treatments (Comparison B)

	Comparison A				Comparison B			
	Baseline	Change from baseline for 4 mg twice a day, as randomized (*n* = 388)	Difference for 100 mg twice a day, as monotherapy (*n* = 76)	*p* value	Baseline	Change from baseline for 4 mg twice a day, as add-on (*n* = 309)	Difference for 4 mg twice a day, as monotherapy (*n* = 79)	*p* value
LVV (cm^3^)
Mean	49.01	7.35	–2.83	<0.0001	49.51	8.01	–3.07	<0.0001
95% CI	48.65, 49.37	6.75, 7.94	–4.12, –1.54		49.39, 49.63	7.37, 8.65	–4.23, –1.92
WBV (cm^3^)
Mean	971	–21.83	6.49	0.0002	980	–23.51	7.77	<0.0001
95% CI	960, 982	–23.37, –20.29	3.03, 9.96		965, 995	–25.18, –21.84	4.64,10.90
HV (mm^3^)
Mean	3072	–120	16.19	0.0909	3049	–129	39.71	<0.0001
95% CI	3014, 3130	–128, –111	–2.58, 34.96		2986, 3112	–137, –120	22.69, 56.72

**Table 4b:** Volumetric MRI outcomes comparing LMTM 100 mg twice a day as monotherapy with 100 mg twice a day as add-on to existing AD treatments (Comparison C)

	Comparison C			
	Baseline for 100 mg twice a day, as add-on (*n* = 297)	Change from baseline	Difference for 100 mg twice a day, as monotherapy (*n* = 79)	*p* value
LVV (cm^3^)
Mean	47.80	7.72	–3.21	<0.0001
95% CI	45.27, 50.33	7.01, 5.84	–4.35, –2.07
WBV (cm^3^)
Mean	969	–24.04	8.70	<0.0001
95% CI	948, 972	–25.90, –22.18	5.55, 11.86
HV (mm^3^)
Mean	3014	–140	36	<0.0001
95% CI	2947, 3081	–150, –130	19, 54

**Fig.4:**
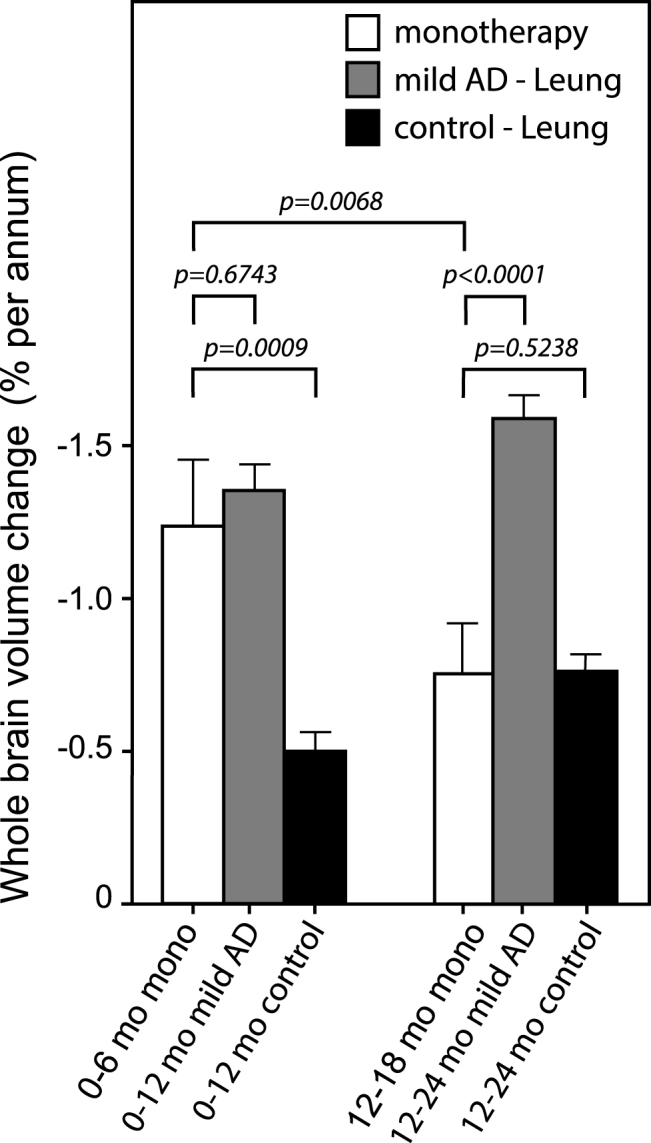
Annualized rate of whole brain atrophy (expressed as %) over months 0–6 and months 12–18 in monotherapy patients. The rates are compared with those reported by Leung et al. for mild AD and normal elderly controls [20].

**Fig.5:**
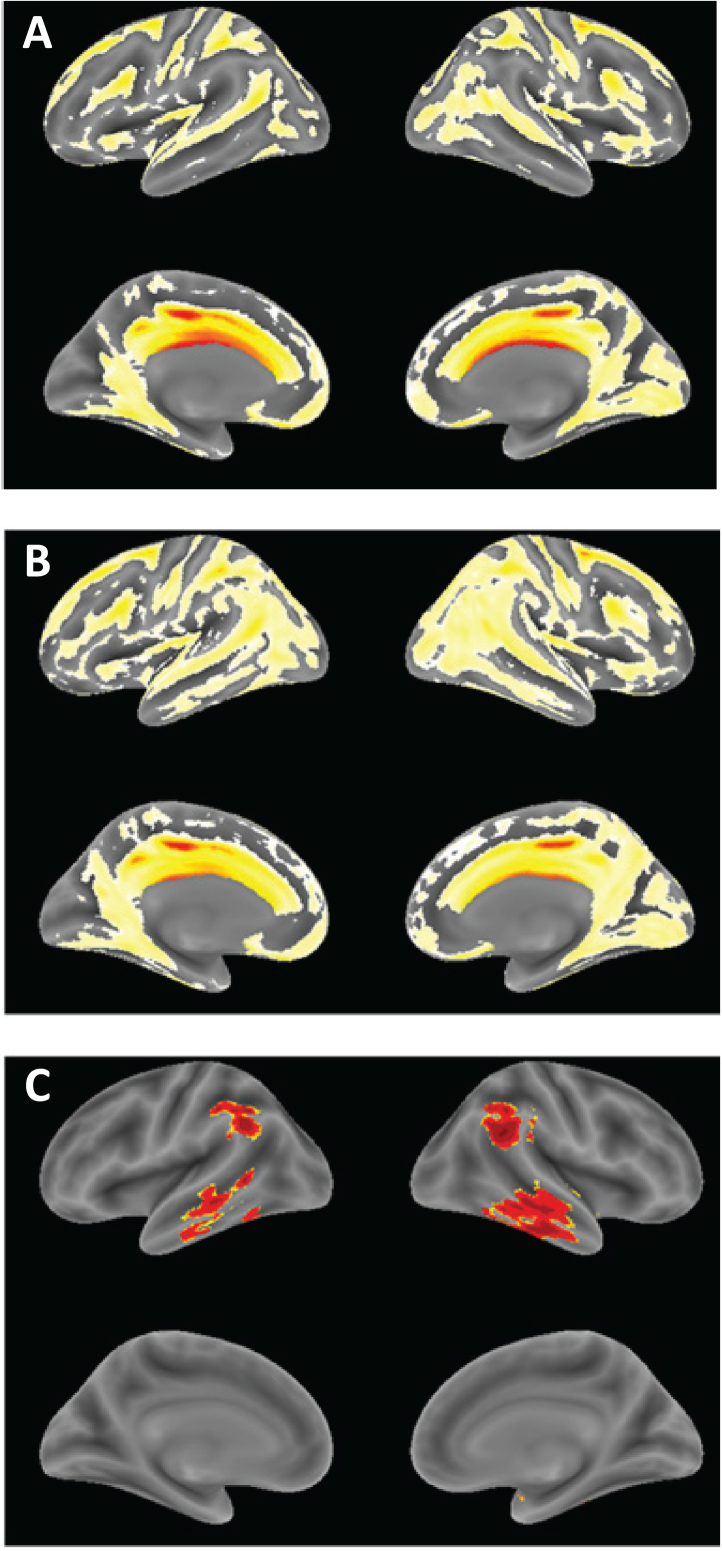
Voxel-based morphometric comparison showing regions of greater atrophy in patients receiving LMTM as monotherapy [N = 157] (A) or as add-on to approved treatments for AD [N = 610], (B) in TRx-237-005 compared with elderly controls [N = 244] from the ongoing Aberdeen birth cohort studies [23, 26], controlled for age, sex and total intracranial volume of each individual. (C) Voxel-based morphometric comparison of monotherapy and add-on patients in TRx-237-005. Data are displayed at a significance threshold corrected for family-wise error at the whole brain level at *p* < 0.05.

The annualized rate of whole brain atrophy over months 0–6 was indistinguishable in both monotherapy and add-on therapy subgroups from those reported [20] for mild AD (*p* = 0.6743 and *p* = 0.2663, respectively), and significantly different from the rate reported in normal elderly controls (*p* < 0.0001 for both). After 9 months of treatment, the rate of progression of atrophy was significantly less than the initial rate in patients receiving LMTM as monotherapy (*p* = 0.0068; Table 5, Fig. 4), but not in patients receiving LMTM as add-on therapy (*p* = 0.3182). The final atrophy rate (months 12–18) in patients receiving LMTM as monotherapy was significantly less than that reported for mild AD [20] (*p* < 0.0001) but similar to normal elderly controls (*p* = 0.5238; Fig. 4). In order to test whether this delayed reduction in rate of atrophy in monotherapy patients could be explained by patients diagnosed clinically as having AD but not having AD-type atrophy at baseline, we undertook a comparison with a well characterized normal aging cohort [23, 26] using voxel-based morphometry. This confirmed that both monotherapy and add-on therapy subgroups had significantly greater atrophy at baseline than normal elderly controls in frontal, temporal and parietal lobes, hippocampus, parahippocampal gyrus, and posterior cingulate cortex (Fig. 5A, B), although add-on therapy patients had somewhat greater temporoparietal atrophy than monotherapy patients(Fig. 5C).

**Table 5:** Comparison of annualized rate of whole brain atrophy for months 0–6 and months 12–18 (cm^3^)

	Months 0–6	Months 12–18	Difference	*p* value
LMTM as monotherapy
Mean	–12.0	–7.3	+4.7	0.0068
95% CI	–16.4, –7.6	–10.2, –4.3	+1.0, +8.5
LMTM as add-on
Mean	–14.0	–13.5	+0.5	0.3182
95% CI	–16.2, –11.8	–15.0, –12.1	–1.4, +2.3

**Fig.6:**
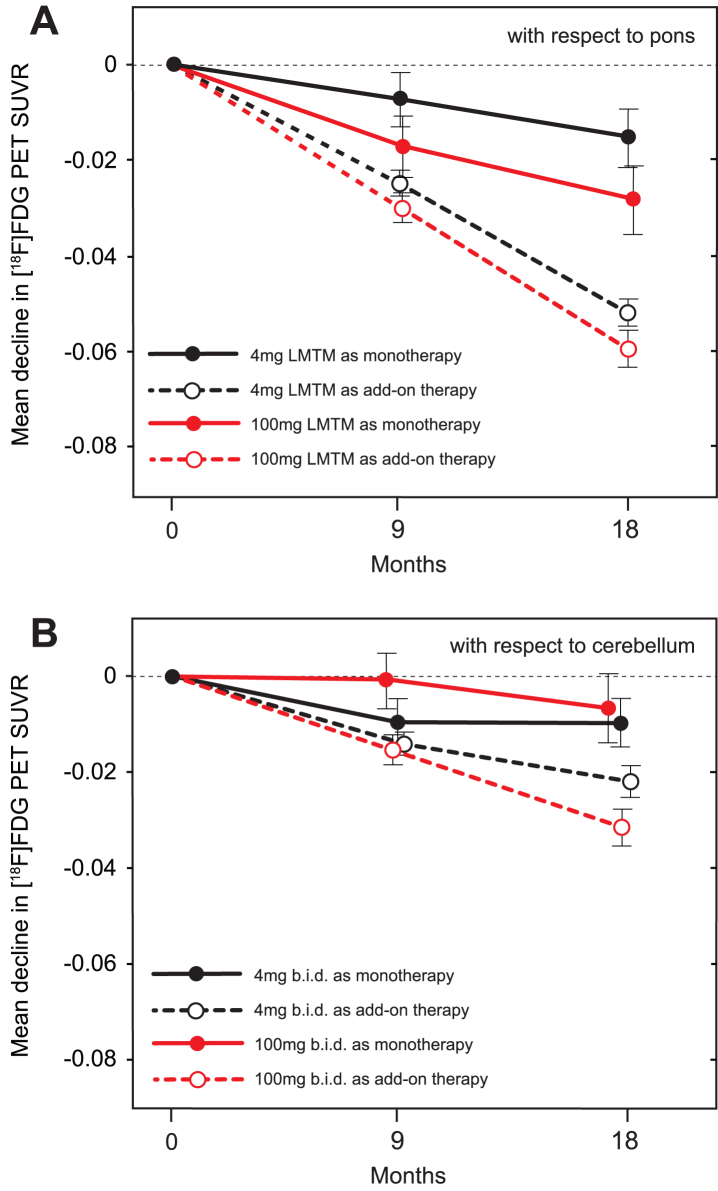
A) Change from baseline at 9-month and 18-month time points for temporal cortex SUVR measured by ^18^F-FDG-PET normalized with respect to pons. B) Change from baseline at 9-month and 18-month time points for temporal cortex SUVR measured by ^18^F-FDG-PET normalized with respect to cerebellum.

Using the same methodology as in the ADNI program [24], baseline ^18^F-FDG-PET SUVR in inferior temporal gyrus normalized with respect to pons was less than that reported for mild AD [24] in both monotherapy (*p* = 0.0032 and *p* = 0.0163, for left and right, respectively) and add-on therapy subgroups (*p* < 0.0001 for both left and right), and substantially less than baseline values reported for MCI and elderly control (*p* < 0.0001 for all; Supplementary Table 6). The results were similar for angular gyrus. Decline in SUVR normalized with respect to pons in frontal, parietal and temporal lobes was significantly less over 18 months in patients receiving LMTM as monotherapy at either the 4 mg or 100 mg twice day doses than in those receiving the same doses as add-on therapy, and in cerebellum only at the 4 mg twice a day dose (Table 6A). These differences were already significant at the 4 mg twice a day dose after 9 months in parietal (*p* = 0.0143) and temporal (*p* = 0.0040; Fig. 6) lobes, but not at the 100 mg twice a day dose. The annual decline in glucose uptake in temporal lobe was significantly less than reported for mild AD [24] at both the 4 mg and 100 mg twice a day doses as monotherapy (*p* < 0.0001 for both) and also as add-on therapy (*p* = 0.0044 and *p* = 0.0355, respectively). Decline in SUVR normalized with respect to cerebellum was likewise significantly less at both doses for LMTM as monotherapy compared with add-on in frontal and parietal cortices at both doses, but only at 100 mg twice a day in temporal cortex (Table 6B).

**Table 6:** ^18^F-FDG-PET SUVR outcomes at 18 months comparing LMTM 100 mg twice a day as monotherapy with 100 mg twice a day as add-on to cholinesterase inhibitors and/or memantine (Comparison C), and LMTM 4 mg twice a day as monotherapy compared with LMTM 4 mg twice a day as add-on to cholinesterase inhibitors and/or memantine (Comparison B)

(A) SUVR normalized with respect to pons							
	Change from baseline for 100 mg twice a day as add-on (*n* = 297)	Difference for 100 mg twice a day as monotherapy (*n* = 75)	*p* value	Change from baseline for 4 mg twice a day as add-on (*n* = 308)	Difference for 4 mg twice a day as monotherapy (*n* = 79)	*p* value
Frontal lobe
Mean	–0.066	+0.045	0.0012	–0.050	+0.045	0.0002
95% CI	–0.079, –0.053	+0.018, +0.072		–0.062, –0.039	+0.021, +0.068
Parietal lobe
Mean	–0.073	+0.045	0.0008	–0.058	+0.050	<0.0001
95% CI	–0.086, –0.061	+0.019, +0.072		–0.069, –0.048	+0.027, +0.073
Temporal lobe
Mean	–0.060	+0.030	0.0002	–0.052	+0.036	<0.0001
95% CI	–0.067, –0.052	+0.014, +0.046		–0.058, –0.045	+0.022, +0.050
Cerebellum
Mean	–0.020	+0.002	0.8661	–0.026	+0.019	0.0440
95% CI	–0.030, –0.010	–0.020, +0.024		–0.035, –0.017	+0.001, 0.038
(B) SUVR normalized with respect to cerebellum							
	Change from baseline for 100 mg twice a day as add-on (*n* = 388)	Difference for 100 mg twice a day as monotherapy (*n* = 76)	*p* value	Change from baseline for 4 mg twice a day as add-on (*n* = 309)	Difference for 4 mg twice a day as monotherapy (*n* = 79)	*p* value
Frontal lobe
Mean	– 0.034	+0.036	0.0003	– 0.018	+0.018	0.0385
95% CI	– 0.044, – 0.025	+0.016, +0.055		– 0.026, – 0.010	+0.001, +0.035
Parietal lobe
Mean	– 0.041	+0.039	0.0001	– 0.025	+0.023	0.0085
95% CI	– 0.051, – 0.032	+0.019, +0.058		– 0.033, – 0.017	+0.006, +0.040
Temporal lobe
Mean	– 0.032	+0.024	0.0016	– 0.022	+0.012	0.0699
95% CI	– 0.039, – 0.024	+0.009, +0.039		– 0.028, – 0.016	– 0.001, +0.025

Comparisons of change in standardized uptake value ratio (SUVR) for brain regions shown relative to cerebellar cortex with correction for atrophy measured by MRI. The decline in glucose uptake was significantly larger for LMTM 100 mg twice a day as add-on therapy than for LMTM 4 mg twice a day as add-on in frontal cortex (*p* = 0.0065), parietal cortex (*p* = 0.0083), and temporal cortex (*p* = 0.0423).

**Fig.7:**
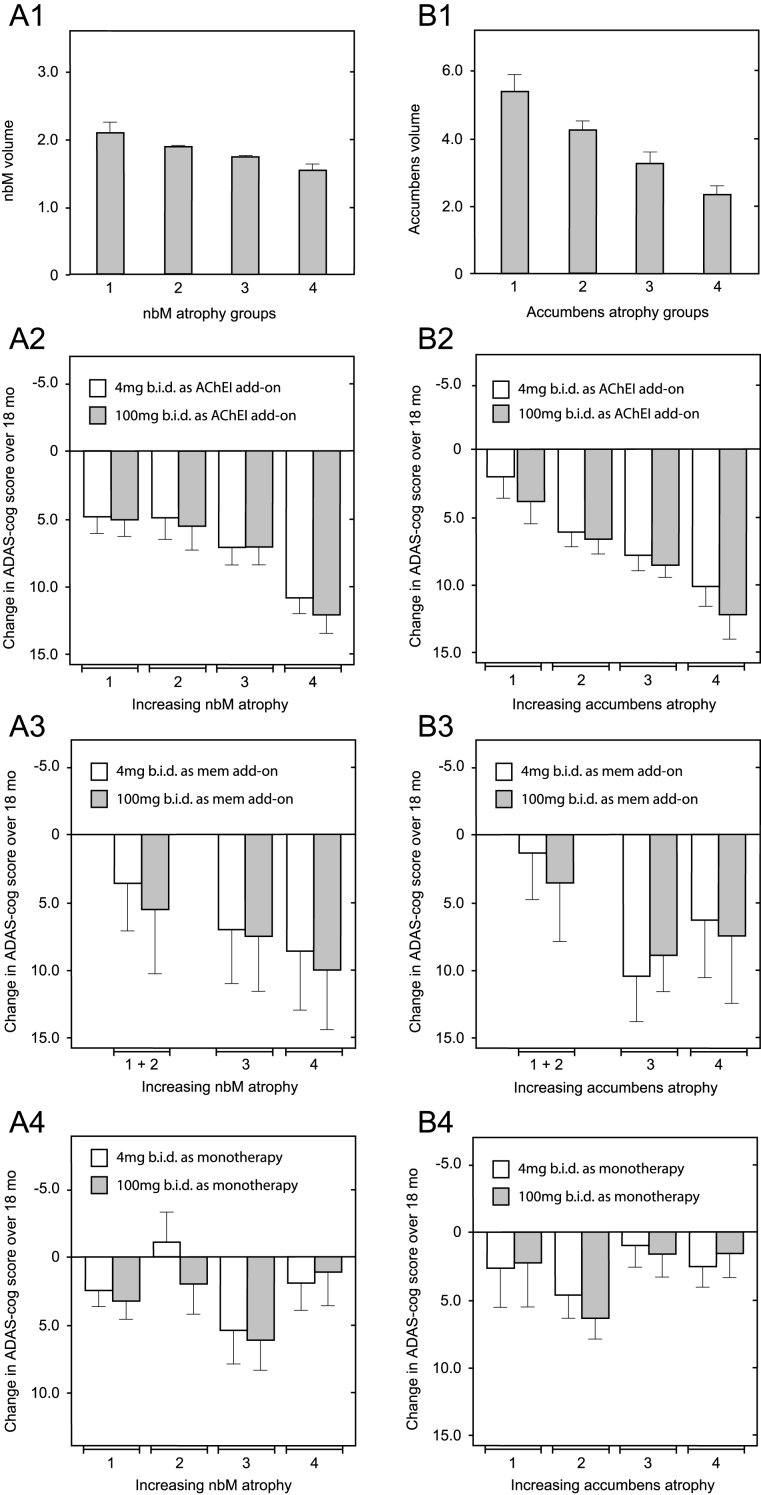
Relationship between decline on the ADAS-cog scale over 18 months and relative volume of nucleus basalis and nucleus accumbens normalized according to AD-comedication treatment status with cholinesterase inhibitors, memantine or LMTM as monotherapy. Mean (±SE) relative volume as a proportion of per-subject whole brain volume (× 10^–4^) of nucleus basalis (A1) and nucleus accumbens (B1) in 4 groups is shown according to increasing degree of atrophy. Illustrative relationship between decline on the ADAS-cog scale over 18 months and nucleus basalis relative atrophy group (A2–A4) and nucleus accumbens relative atrophy group (B2 –B4) in patients receiving LMTM in combination with an acetylcholinesterase inhibitor (A2 & B2), with memantine (A3 & B3) or as monotherapy (A4 & B4). Statistical relationships reported in the main text are for the overall significance of the term *covariate x visit* in explaining cognitive decline.

Since correction for differences in baseline whole brain volume, temporoparietal volume, glucose uptake, and clinical severity did not eliminate the differences favoring patients receiving LMTM as monotherapy over add-on patients, we examined the potential role of relative basal forebrain atrophy. This was determined relative to per-subject whole brain volume to control for overall atrophy. Decline on the ADAS-cog scale in patients receiving LMTM as add-on to cholinesterase inhibitors was strongly dependent on relative atrophy in nucleus basalis of Meynert (Fig. 7A2; *p* = 0.0017) and nucleus accumbens (Fig. 7B2; *p* < 0.0001). In patients receiving LMTM as add-on to memantine, the effect was either weak (nucleus basalis: *p* = 0.0391; Fig. 7A3) or absent (nucleus accumbens: *p* = 0.2453; Fig. 7B3). Basal forebrain atrophy had no influence on treatment outcome in patients receiving LMTM as monotherapy, either for nucleus basalis (*p* = 0.5746; Fig. 7A4) or for nucleus accumbens (*p* = 0.1355; Fig. 7B4). In patients with the greatest atrophy in nucleus basalis, cognitive decline in patients receiving LMTM as monotherapy was significantly less than those receiving LMTM in combination with either cholinesterase inhibitors (*p* < 0.0001) or memantine (*p* = 0.0128). The corresponding effect of nucleus basalis atrophy on cortical glucose uptake was seen only in patients receiving LMTM in combination with a cholinesterase inhibitor (*p* = 0.0043), but not in combination with memantine (*p* = 0.1384) or in patients receiving LMTM as monotherapy (*p* = 0.9879). Given the importance of basal forebrain atrophy in determining treatment outcome for combination therapy, we examined whether this could explain the differences favoring LMTM as monotherapyby including relative basal forebrain atrophy at baseline as a rate-correction term in the analysis model for ADAS-cog. Again, the treatment differences remained significant for both 4 mg twice a day and 100 mg twice a day as monotherapy compared with add-on therapy (Supplementary Table 7).

Total tau and Aβ_1–42_ in CSF increased significantly (*p* < 0.0001 and *p* = 0.0323 respectively) in subjects taking LMTM as add-on to existing treatments but not in patients taking LMTM as monotherapy. Changes in phosphorylated tau were not significant in either group (Table 7).

**Table 7:** Baseline values and change from baseline to end of study for Aβ_1 - 42_, total tau, and phospho-tau

		Baseline	Change from baseline	*p* value
Aβ_1 - 42_ (ng/L)
LMTM as add-on therapy [*n* = 137&66]	Mean	307	20	0.0323
	95% CI	277, 337	–3, 43
LMTM as monotherapy [*n* = 36&15]	Mean	377	–14	0.6698
	95% CI	310, 444	–75, 48
Total tau (ng/L)
LMTM as add-on therapy [*n* = 136&66]	Mean	110	22	<0.0001
	95% CI	93, 126	11, 34
LMTM as monotherapy [*n* = 36&15]	Mean	112	10	0.2166
	95% CI	756 148	–30, 40
Phospho-tau (ng/L)
LMTM as add-on therapy [*n* = 138&66]	Mean	46	3	0.4987
	95% CI	40, 53	–2, 9
LMTM as monotherapy [*n* = 36&15]	Mean	41	11	0.2166
	95% CI	28, 54	0, 23

Number of samples at baseline and at end of study given as monotherapy dose groups and add-on therapy dose groups pooled, [*n* = baseline & end].

### Safety results

Safety data according to dose and AD co-medication use are reported in Table 8. Gastrointestinal and urinary related adverse events were the most common treatment emergent adverse events, occurring in more patients receiving the 100 mg twice a day dose than in those receiving the 4 mg twice a day dose (Table 8). These were also the most common reasons for discontinuing the 100 mg twice a day dose (40 of 396 patients; 10%) compared with 9 of 399 patients (2%) taking the 4 mg twice a day dose. The incidence of targeted gastrointestinal adverse events was almost twice as high in patients receiving LMTM as add-on therapy regardless of dose (268 of 627 patients; 43%) compared with those receiving LMTM alone (45 of 167 patients; 27%).

**Table 8:** Treatment emergent adverse events occurring in at least 5% of patients receiving LMTM according to approved Alzheimer’s disease co-medication use and dose (denominators based on actual AD-co-medication use)

	4 mg twice a day as add-on (*n* = 313)	100 mg twice a day as add-on (*n* = 317)	4 mg twice a day as monotherapy (*n* = 83)	100 mg twice a day as monotherapy (*n* = 81)
Subjects reporting at least one treatment-emergent adverse event	276 (88.2%)	276 (87.1%)	60 (72.3%)	70 (86.4%)
Blood and lymphatic system disorders	15 (4.8%)	25 (7.9%)	3 (3.6%)	3 (3.7%)
Anemia	6 (1.9%)	17 (5.4%)	3 (3.6%)	2 (2.5%)
Cardiac disorders	24 (7.7%)	25 (7.9%)	7 (8.4%)	3 (3.7%)
Ear and labyrinth disorders	9 (2.9%)	6 (1.9%)	6 (7.2%)	2 (2.5%)
Eye disorders	21 (6.7%)	18 (5.7%)	4 (4.8%)	4 (4.9%)
Gastrointestinal disorders	101 (32.3%)	152 (47.9%)	19 (22.9%)	33 (40.7%)
Diarrhea	60 (19.2%)	100 (31.5%)	7 (8.4%)	24 (29.6%)
Nausea	18 (5.8%)	33 (10.4%)	5 (6.0%)	8 (9.9%)
Vomiting	11 (3.5%)	21 (6.6%)	2 (2.4%)	7 (8.6%)
General disorders and administration site conditions	49 (15.7%)	52 (16.4%)	8 (9.6%)	10 (12.3%)
Fatigue	11 (3.5%)	16 (5.0%)	1 (1.2%)	4 (4.9%)
Infections and infestations	112 (35.8%)	115 (36.3%)	22 (26.5%)	17 (21.0%)
Nasopharyngitis	18 (5.8%)	17 (5.4%)	3 (3.6%)	3 (3.7%)
Urinary tract infection	32 (10.2%)	42 (13.2%)	2 (2.4%)	5 (6.2%)
Injury, poisoning and procedural complications	74 (23.6%)	59 (18.6%)	10 (12.0%)	15 (18.5%)
Fall	42 (13.4%)	30 (9.5%)	7 (8.4%)	6 (7.4%)
Investigations	84 (26.8%)	93 (29.3%)	17 (20.5%)	18 (22.2%)
Blood folate decreased	15 (4.8%)	23 (7.3%)	3 (3.6%)	7 (8.6%)
Weight decreased	7 (2.2%)	12 (3.8%)	4 (4.8%)	6 (7.4%)
Metabolism and nutrition disorders	42 (13.4%)	49 (15.5%)	6 (7.2%)	17 (21.0%)
Decreased appetite	5 (1.6%)	19 (6.0%)	0	4 (4.9%)
Folate deficiency	3 (1.0%)	9 (2.8%)	1 (1.2%)	5 (6.2%)
Musculoskeletal and connective tissue disorders	70 (22.4%)	58 (18.3%)	13 (15.7%)	21 (25.9%)
Back pain	16 (5.1%)	14 (4.4%)	2 (2.4%)	8 (9.9%)
Nervous system disorders	108 (34.5%)	112 (35.3%)	22 (26.5%)	17 (21.0%)
Dizziness	24 (7.7%)	26 (8.2%)	3 (3.6%)	4 (4.9%)
Headache	18 (5.8%)	22 (6.9%)	6 (7.2%)	5 (6.2%)
Psychiatric disorders	81 (25.9%)	99 (31.2%)	16 (19.3%)	15 (18.5%)
Agitation	16 (5.1%)	24 (7.6%)	0	0
Anxiety	23 (7.3%)	22 (6.9%)	5 (6.0%)	3 (3.7%)
Confusional state	7 (2.2%)	17 (5.4%)	0	5 (6.2%)
Depression	23 (7.3%)	15 (4.7%)	5 (6.0%)	2 (2.5%)
Renal and urinary disorders	39 (12.5%)	97 (30.6%)	7 (8.4%)	17 (21.0%)
Dysuria	1 (0.3%)	31 (9.8%)	2 (2.4%)	4 (4.9%)
Pollakiuria	8 (2.6%)	26 (8.2%)	2 (2.4%)	2 (2.5%)
Urinary incontinence	11 (3.5%)	22 (6.9%)	1 (1.2%)	2 (2.5%)
Respiratory, thoracic and mediastinal disorders	41 (13.1%)	32 (10.1%)	5 (6.0%)	7 (8.6%)
Cough	16 (5.1%)	13 (4.1%)	2 (2.4%)	2 (2.5%)
Skin and subcutaneous tissue disorders	41 (13.1%)	38 (12.0%)	4 (4.8%)	10 (12.3%)
Vascular disorders	25 (8.0%)	24 (7.6%)	4 (4.8%)	3 (3.7%)

## DISCUSSION

There was no evidence of any difference on any of the primary or secondary endpoints in the as-randomized analyses defined in the protocol comparing all patients receiving LMTM at a dose of 100 mg twice a day and those receiving 4 mg twice a day. In the non-randomized cohort comparisons defined as the primary outcomes in the revised Statistical Analysis Plan finalized prior to database lock and unblinding, both primary Comparisons A and B met the required statistical threshold of *p* < 0.025 for both co-primary clinical outcomes (ADAS-cog and ADCS-ADL), as well as for volumetric MRI and glucose uptake biomarker outcomes. Patients receiving LMTM as monotherapy at either of the two doses tested had consistently better outcomes than patients receiving the same doses as add-on to cholinesterase inhibitors and/or memantine, and patients receiving 100 mg twice as day as monotherapy had better outcomes than patients receiving 4 mg twice a day as randomized. There was no difference between 4 mg and 100 mg twice a day as monotherapy in the corresponding monotherapy versus add-on therapy treatment comparisons.

The confirmation of the same pattern of results in this second independent study argues against either the present findings or those reported as *post hoc* findings from the earlier mild/moderate AD study [16] being the result of chance in small subgroups, although the monotherapy subgroups remain small in the present study (155 or 20% in total in the mITT analyses). It is also unlikely that the earlier findings are explicable by inclusion of non-Western geographies, since the present study was conducted in North America, Western Europe, and Australia. A clinical placebo effect in patients coming into a trial setting after previously not receiving active treatment cannot explain the same pattern of results seen in both the MRI brain atrophy and glucose uptake data as seen in the clinical data. A difference in withdrawal rates between patients taking or not taking standard AD treatments is also unlikely, since the overall retention rates over 18 months were similar in monotherapy (65%) and add-on (69%) treatment groups.

The pattern of atrophy at baseline in patients receiving LMTM as monotherapy was typical of mild AD and significantly different from a cohort of well characterized normal elderly controls [26]. The annualized rate of whole brain atrophy in these patients over the first 6 months was also similar to that reported for mild AD and significantly different from normal elderly controls [20]. Likewise glucose uptake in inferior temporal gyrus was comparable in both monotherapy and add-on patients to that reported for mild AD [24] and significantly different from MCI or normal elderly controls [24]. In addition to meeting clinical diagnostic criteria for mild AD, the baseline imaging data therefore confirm that the patients not prescribed cholinesterase inhibitors or memantine were typical of mild AD.

Patients not receiving standard AD treatments were somewhat less impaired at study entry on the ADAS-cog, ADCS-ADL, and MMSE scales, as well as in ventricular, temporoparietal, and hippocampal volumes, and temporal lobe glucose uptake. It is therefore possible that this difference in severity at baseline might have accounted for significant differences in progression. However, baseline severity was included as an additive term in the primary analysis models and was therefore corrected for as an additive effect. We further tested whether baseline severity or other patient characteristics could explain differences in rate of progression by undertaking sensitivity analyses with additional rate-correction terms in the analysis model. If differences in baselinecharacteristics explain the differences in rate of progression over 18 months, then the significant differences in favor of LMTM as monotherapy would be expected to disappear when rate was corrected for baseline effects. In a similar analysis for patients with MMSE 20–26 in the currently available ADNI data set, this correction for baseline severity eliminated the apparent differences in rate of progression (publication in preparation). Rate-correction for differences in clinical severity at baseline, APOE *ɛ*4 frequency, vascular pathology load, hippocampal atrophy, temporoparietal atrophy, glucose uptake in the temporal lobe, and basal forebrain atrophy did not eliminate the significant differences in favor of LMTM monotherapy for ADAS-cog, ADCS-ADL, or lateral ventricular volume. We further examined whether the differences in favor of LMTM as monotherapy depend on inclusion of patients receiving a cholinesterase inhibitor and memantine in combination as this was found to predict more rapid decline in an MCI cohort [27]. Removing them had minimal effect on the estimates or significance of the treatment differences. It therefore appears unlikely that the relatively minor differences in severity or the other characteristics at baseline explain the significant outcome differences in favor of LMTM monotherapy over 18 months.

An analysis that is free of potential between-cohort confounding effects is the within-cohort comparison of annualized rate of whole brain atrophy at study entry and after 9 months of treatment with LMTM. We found that in patients receiving LMTM as monotherapy there was a significant delayedreduction in the annualized rate of whole brain atrophy. As noted above, monotherapy patients entered the study with an initial rate of progression of whole brain atrophy typical of mild AD and significantly greater than reported for normal elderly controls [20]. After receiving LMTM as monotherapy for 9 months, the rate was reduced to that reported for normal elderly controls and was significantly less than expected for mild AD [20]. Similarly, the decline in temporal lobe glucose uptake in patients receiving LMTM as monotherapy was significantly less than reported for mild AD [24].

On the ADAS-cog scale, treatment response to LMTM in combination with a cholinesterase inhibitor was found to vary inversely with atrophy in the nucleus basalis and nucleus accumbens corrected for whole brain volume and clinical severity. A similar effect was also seen for cortical glucose uptake. The corresponding effect of basal forebrain atrophy was weaker for the LMTM/memantine combination. Both of these basal forebrain nuclei are known to be affected by tau aggregation pathology [28, 29], and may precede cortical pathology [30]. The inverse relationship we report is the opposite of that reported for the response to donepezil [31], which varies in proportion with basal forebrain atrophy, and donepezil has also been shown recently to reduce the rate of progression of basal forebrain atrophy in prodromal AD [32]. The response to LMTM in combination with cholinesterase inhibitors appears therefore to differ from the response to cholinesterase inhibitors alone. In contrast, cognitive decline in patients receiving LMTM as monotherapy does not vary according to the severity of basal forebrain atrophy. These differences in treatment response cannot therefore be attributed simply to cohort differences in rates of disease progression between patients prescribed or not prescribed such treatments. It also cannot be attributed to relative lack of pathology, since patients with the greatest basal forebrain atrophy responded significantly better to monotherapy than to combination treatment. Rather, our findings point to pharmacological differences in the effects of LMTM on target neurons which depend on presence or absence of activating drugs and on the indirect effects of basal forebrainpathology.

The role of nucleus basalis in determining treatment response may help to provide some insight into the possible mechanism underlying the negative interaction with cholinesterase inhibitors. Ascending cholinergic projections originating predominantly from nucleus basalis provide both direct activation [33] and indirect inhibitory modulation of cortical pyramidal cells [34]. Memantine also increases release of acetylcholine in nucleus accumbens [35] which modulates cortical activity indirectly. We have previously reported increased expression of synaptic proteins at Braak stages 3 and 4 in neocortex AD which would be consistent with disinhibition [7]. Long-term inhibition of cholinesterase activity combined with loss of inhibitory modulation may therefore result in chronic hyperactivation of pyramidal cells in cortex which are the principal sites of neurofibrillary degeneration in AD [36]. It is possible that the relative severity of basal forebrain pathology together with chronic cholinesterase inhibition may determine the degree of hyperactivation of cortical pyramidal cells and that this may impair the clearance of tau monomers released by MT [10].

The potential for LMTM to be active at the low dose of 4 mg twice a day and the lack of dose-response was unexpected given the results of an earlier Phase II placebo-controlled study using the oxidized form of the methylthioninium (MT) moiety as methylthioninium chloride (MTC) [13]. The dihydromethanesulfonate salt was developed to stabilize the reduced form of the MT moiety in the solid state to permit dosing in the reduced form and so overcome the absorption limitations observed for the oxidized form using MTC [15, 37]. LMTM is now known to have a 20-fold better red cell uptake than MTC *in vivo* and also better brain uptake [15]. We have reported that the estimated steady state trough brain concentration of MT, at the minimum effective dose of MTC, is 0.1–0.2μM [15]. A population pharmacokinetic analysis using blood samples collected in the course of the present study, combined with rat and pig data to estimate brain levels, suggests that the estimated brain concentration of MT at the 4 mg twice a day dose is in the range 0.05–0.2μM (publication in preparation). The concentration required for dissolution of paired helical filaments isolated from AD brain tissue [37] and oligomers *in vitro* is approximately 1/10th that of aggregated tau, implying that a concentration of 0.05μM may be adequate *in vivo*, given the brain concentrations of aggregated tau that have been reported inAD [38–40].

A concentration of approximately 0.05μM also appears to be adequate for a range of other potentially beneficial effects of the MT moiety such as enhancement of autophagy [41] and enhancement of mitochondrial function [42–49]. There is no dose-response for oligomer disaggregation *in vitro*, and higher doses of LMTM do not result in greater reduction in tau pathology in transgenic mouse models [50]. This suggests that there may be a critical threshold for activity at the tau aggregation inhibitor target, and the effect of higher doses on pathology may plateau or may even become negative at brain concentrations above 1μM [50]. Higher doses of MTC are also less effective inducers of mitochondrial biogenesis and of NF-E2-related factor 2 (Nrf2) which control pathways available for clearance of proteotoxic proteins in tau transgenic mice [51], and are less effective for memory enhancement in wild type rodents [46]. Several results in the present study also suggest that 4 mg twice a day may serve better than 100 mg twice a day. The clinical differences in favor of 4 mg twice a day were seen at both CDR 0.5 and 1.0, but only at CDR 0.5 at the higher dose, and the glucose uptake difference in temporal cortex occurred earlier at the lower dose.

The lower dose of 4 mg twice a day had a better overall clinical profile than 100 mg twice a day. The withdrawal rate over 18 months for the 4 mg twice a day dose was less (25%, 94/296) than at 100 mg twice a day (46%, 182/399), and the adverse event profile was more benign with respect to the diarrhea, dysuria, and decreased hemoglobin. There is no increased risk of cerebral microhemorrhages or edema with LMTM even at the higher dose, since the ARIA rates observed in both Phase III studies were similar to those previously reported for placebo controls [21, 22].

In addition to inhibition of tau aggregation [37], enhancement of autophagy [41], and induction of proteotoxic clearance pathways at low concentration [51], the MT moiety has several pharmacological actions which are consistent with potentially neuroprotective, antioxidant and symptomatic effects [46]. Enhancement of mitochondrial metabolism [42–49] has been proposed as a mechanism underlying acute neurocognitive benefits in wild-type rodents [46] and in healthy young human volunteers [46, 52, 53]. We show that the difference in glucose uptake between LMTM monotherapy and add-on therapy is 2–3-fold greater in neocortical regions affected by neurofibrillary degeneration [6–8] than in cerebellum which is not affected [54]. We also show that the cortical benefits in favor of LMTM monotherapy can be seen when SUVR is normalized with respect to cerebellum, thereby correcting for general effects on mitochondrial metabolism. This, together with the contrasting effects of LMTM on total tau (although not phospho-tau) in CSF in monotherapy and add-on patients, supports the idea that the benefits in favor of LMTM as monotherapy are linked to tau pathology and to tau metabolism.

The differences in favor of LMTM as monotherapy are based on non-randomized cohort comparisons, albeit defined *a priori* as statistically primary outcomes for the modified analyses we report here. Using modern statistical techniques, estimates of treatment effects from cohort comparisons are comparable with randomized controlled studies in most disease areas [55]. The differences between LMTM as monotherapy and add-on therapy are likewise comparable with the effect sizes found in the earlier placebo controlled monotherapy study of MTC at a comparable brain concentration of MT [13]. Although we have excluded differences in severity, extent of brain atrophy, severity of glucose uptake deficit, AD diagnosis, and concomitant vascular pathology at baseline as explaining the treatment differences in favor of LMTM monotherapy, non-obvious or unmeasured confounding factors cannot be excluded without a further randomized clinical trial comparing LMTM monotherapy with true placebo. However, the same pattern of results has been seen now in two separate Phase III studies, implying that the effects are consistent across studies. The differences favoring monotherapy are also internally consistent across a range of clinical outcomes, and the clinical outcomes are consistent with the neuroimaging outcomes in both studies. Since measurable cohort differences in diagnosis or severity do not appear to explain the treatment differences we report, we believe that the within- and between-study consistency of the results cannot be dismissed lightly, particularly given the urgent need for new treatments in AD [56]. If the results are confirmed in a further suitably randomized clinical trial, they point to clinical and biological effects of LMTM as monotherapy at the safe and well-tolerated dose of 4 mg twice a day which could provide a clinically meaningful addition to the available treatment optionsfor AD.

## Supplementary Material

Supplementary Material

Supplementary Material

Supplementary Material
