# Supplementary material for: Potential of Low Dose Leuco-Methylthioninium Bis(Hydromethanesulphonate) (LMTM) Monotherapy for Treatment of Mild Alzheimer’s Disease: Cohort Analysis as Modified Primary Outcome in a Phase III Clinical Trial
Source: J Alzheimers Dis. 2017 Nov 28;61(1):435–57. doi: 10.3233/JAD-170560 (PMC5734125; doi:10.3233/JAD-170560)

SUPPLEMENTARY MATERIAL

Potential of low dose leuco-methylthioninium bis(hydromethanesulphonate) (LMTM) monotherapy for treatment of mild Alzheimer's disease: observational cohort analysis as modified primary outcome in a phase III clinical trial

Gordon K. Wilcock, Serge Gauthier, Giovanni B. Frisoni, Jianping Jia, Jiri H. Hardlund, Hans J. Moebius, Peter Bentham, Karin A. Kook, Bjoern O. Schelter, Damon J. Wischik, Charles S. Davis, Roger T. Staff, Vesna Vuksanovic, Trevor S. Ahearn, Luc Bracoud, Kohkan Shamsi, Ken Marek, John Seibyl, Gernot Reidel, John M.D. Storey, Charles R. Harrington, Claude M Wischik

Correspondence to: Claude M Wischik, School of Medicine, Medical Sciences and Nutrition, University of Aberdeen, Aberdeen AB25 2ZP, UK. E-mail: cmw@taurx.com

Supplementary Tables 1-6

Supplementary Figure 1

Supplementary Table 1

Comparison of monotherapy versus add-on treatment differences for LMTM 4 mg twice a day and 100 mg twice a day.

		Decline for 4 mg twice a day as add-on therapy (n=309)	Difference for 4 mg twice a day, as monotherapy (n=79)	Decline for 100 mg twice a day as add-on therapy (n=297)	Difference for 100 mg twice a day as monotherapy (n=76)	*p* value for monotherapy difference comparison
ADAS-cog	Mean	7.13	-4.22	7.24	-4.08	0.8002
	95% CI	6.09, 8.18	-6.19, -2.24	6.08, 8.40	-6.07, -2.08	
ADCS-ADL	Mean	-9.17	4.85	-9.99	5.27	0.7358
	95% CI	-10.52, -7.82	2.31, 7.40	-11.50, -8.49	2.70, 7.84	

Data expressed as mean with 95% CI.

Supplementary Table 2a

Co-primary outcomes examining LMTM 100 mg twice a day as monotherapy compared with the control arm as randomized (Comparison A), and LMTM 4 mg twice a day as monotherapy compared with 4 mg twice a day as add-on to existing Alzheimer's disease treatments (Comparison B) after removal of patients taking a cholinesterase inhibitor in combination with memantine from the analysis.

		Comparison A			Comparison B		
		Change from baseline for 4 mg twice a day, as randomized (n=388)	Difference for 100 mg twice a day, as monotherapy (n=82)	*p* value	Change from baseline for 4 mg twice a day, as add-on (n=316)	Difference for 4 mg twice a day, as monotherapy (n=80)	*p* value
ADAS-cog	Mean	5.79	-2.43	0.0334	6.84	-4.08	0.0001
	95% CI	4.70, 6.88	-4.68, -0.19		5.63, 8.05	-6.12,-2.05	
ADCS-ADL	Mean	-7.84	3.37	0.0235	-9.10	4.86	0.0003
	95% CI	-9.26, -6.42	0.45, 6.29		-10.67, -7.52	2.24, 7.48	

Data expressed as mean with 95% CI.

Supplementary Table 2b

Co-primary outcomes examining LMTM 100 mg twice a day as monotherapy compared with 100 mg twice a day as add-on to existing Alzheimer's disease treatments (Comparison C) after removal of patients taking a cholinesterase inhibitor in combination with memantine from the analysis.

		Comparison C		
		**Change from baseline for 100 mg twice a day, as add-on (n=388)**	**Difference for 100 mg twice a day, as monotherapy (n=82)**	***p* value**
ADAS-cog	Mean	7.28	-3.93	0.0002
	95% CI	5.97, 8.60	-5.98, -1.88	
ADCS-ADL	Mean	-9.99	5.22	0.0001
	95% CI	-11.40, -7.98	2.58, 7.86	

Data expressed as mean with 95% CI.

Supplementary Table 3a

Comparisons for primary and secondary outcomes according to CDR severity at baseline. Differences are shown with respect to the control change from baseline at 78 weeks as specified for comparisons A and B. Two parallel implementations of sequential tests were prespecified to examine LMTM 100 mg twice a day as monotherapy compared with the control arm as randomized (comparison A), and LMTM 4 mg twice a day as monotherapy compared with 4 mg twice a day as add-on to existing Alzheimer's disease treatments (comparison B).

		Comparison A				Comparison B			
		**Baseline**	**Change from baseline for 4 mg twice a day as randomized**	**Difference for 100 mg twice a day as monotherapy**	***p* value**	**Baseline**	**Change from baseline for 4 mg twice a day as add-on**	**Difference for 4 mg twice a day as monotherapy**	***p* value**
**CDR 0.5**			n=242	n=59			n=181	n=61	
ADAS-cog	Mean	15.61	5.22	-2.19	0.0847	16.15	5.96	-2.92	0.0108
	95% CI	14.84, 16.38	4.01, 6.42	-4.67, 0.30		15.30, 17.00	4.63, 7.29	-5.17,-0.67	
ADCS-ADL	Mean	69.85	-6.33	2.89	0.0682	69.51	-7.38	4.08	0.0040
	95% CI	69.15, 70.85	-7.84, -4.82	-0.22, 6.00		68.69, 70.33	-9.05, -5.72	1.31, 6.86	
LVV (cm^3)	Mean	49.60	6.84	-2.67	0.0003	53.03	7.65	-2.88	<0.0001
	95% CI	46.59, 52.61	6.11, 7.56	-4.13, -1.22		49.64, 56.42	6.85, 8.46	-4.16, -1.59	
ADCS-CGIC	Mean		-0.91	0.24	0.1160		-1.02	0.44	0.0014
	95% CI		-1.04, -0.77	-0.06, 0.54			-1.17, -0.87	0.17, 0.71	
MMSE	Mean	23.62	-2.68	0.86	0.2272	23.61	-3.06	1.48	0.0214
	95% CI	23.38, 23.86	-3.12, -2.04	-0.54, 2.26		23.33, 23.89	-3.77, -2.34	0.22, 2.74	
MADRS	Mean	4.92	-0.06	-0.40	0.6323	4.98	0.11	-0.69	0.3540
	95% CI	4.32, 5.22	-1.86, 0.71	-2.03, 1.23		4.27, 5.69	-0.68, 0.91	-2.13, 0.76	
NPI	Mean	7.05	1.79	0.26	0.8659	7.20	2.17	-1.89	0.1732
	95% CI	6.80, 8.10	0.56, 3.02	-2.81, 3.34		5.99, 8.41	0.83, 3.52	4.61, 0.83	
**CDR 1.0**			n=146	n=17			n=128	n=18	
ADAS-cog	Mean	20.51	8.24	-4.87	0.0396	20.55	8.87	-6.69	0.0020
	95% CI	19.22, 21.80	6.61, 9.88	-9.52, -0.23		19.18, 21.92	7.17, 10.56	-10.94,-2.45	
ADCS-ADL	Mean	63.33	-8.21	3.48.62	0.0157	62.86	-9.17	4.85	0.0002
	95% CI	61.85, 64.81	-9.46, -6.95	0.66, 6.30		61.26, 64.46	-10.52, -7.82	2.31, 7.40	
LVV (cm^3)	Mean	53.31	8.24	-2.48	0.0871	54.50	8.52	-3.15	0.0186
	95% CI	48.95, 57.67	7.20, 9.29	-5.31, 0.36		49.77, 59.23	7.45, 9.60	-5.77, -0.53	
ADCS-CGIC	Mean		-1.00	0.27	0.0521		-1.09	0.42	0.0007
	95% CI		-1.11, -0.89	-0.00, 0.53			-1.21, -0.96	0.17, 0.66	
MMSE	Mean	22.12	-3.97	2.51	0.0632	21.99	-4.06	0.98	0.4139
	95% CI	21.83, 22.41	-4.82, -3.12	-0.14, 5.17		21.69, 22.29	-4.96, -3.17	-1.37, 3.33	
MADRS	Mean	4.63	0.64	-0.15	0.9148	4.36	0.73	-1.00	0.4227
	95% CI	3.90, 5.36	-0.20, 1.48	-2.85, 2.56		3.62, 5.10	-0.15, 1.60	-3.45, 1.45	
NPI	Mean	9.37	1.79	0.26	0.8659	9.63	2.17	-1.89	0.1732
	95% CI	7.66, 11.08	0.56, 3.02	-2.81, 3.34		7.75, 11.51	0.83, 3.52	4.61, 0.83	

Supplementary Table 3b

Comparisons for primary and secondary outcomes according to CDR severity at baseline, comparing 100 mg twice a day as monotherapy with the same dose as add-on to existing treatments (Comparison C).

		Baseline	Change from baseline for 100 mg twice a day as add-on	Difference for 100 mg twice a day as monotherapy	*p* value
**CDR 0.5**			n=242	n=59	
ADAS-cog	Mean	16.93	6.36	-3.33	0.0038
	95% CI	15.33, 17.27	4.92, 7.39	-5.58, -1.07	
ADCS-ADL	Mean	69.12	-7.87	4.43	0.0019
	95% CI	69.16, 70.08	-9.68, -6.06	1.64, 7.22	
LVV (cm^3)	Mean	45.47	7.20	-3.04	<0.0001
	95% CI	42.35, 48.59	6.33, 8.07	-4.31, -1.76	
CGIC	Mean		-0.97	0.30	0.0362
	95% CI		-1.14, -0.81	0.02, 0.59	
MMSE	Mean	23.27	-3.37	1.63	0.159
	95% CI	22.97, 23.57	-4.17, -2.56	0.31, 2.96	
**CDR 1.0**			n = 146	n = 17	
ADAS-cog	Mean	20.40	8.71	-5.35	0.0164
	95% CI	19.19, 21.61	6.75, 10.68	-9.71, -0.98	
ADCS-ADL	Mean	62.49	-13.13	5.62	0.0624
	95% CI	61.06, 63.92	-15.82, -10.44	-0.29, 11.54	
LVV (cm^3)	Mean	55.86	8.57	-2.80	0.0364
	95% CI	51.17, 60.55	7.32, 9.82	-5.42, -0.18	
CGIC	Mean		-1.33	0.40	0.1613
	95% CI		-1.57, -1.09	-0.16, 0.97	
MMSE	Mean	22.14	-4.56	3.10	0.0198
	95% CI	21.81, 22.47	-5.60, -3.52	0.59, 5.71	

Supplementary Table 4

Two time-point analyses to determine whether observational cohort differences for Comparisons A, B, and C of the co-primary outcomes (ADAS-cog and ADCS-ADL) increase at 18 months compared with 9 months.

Comparison	Outcome		Effect size		Two time-point difference	*p*-value for two time-point difference
			9 months	18 months		
A	ADAS-cog	Mean	-1.32	-3.14	-2.43	0.0174
		95% CI	-2.76, 0.13	-5.32, -0.97	-4.43, -0.43	
		p value	0.0735	0.0047		
	ADCS-ADL	Mean	1.70	3.48	2.37	0.0866
		95% CI	-0.10, 3.50	0.66, 6.31	-0.34, 5.09	
		p value	0.0639	0.0156		
B	ADAS-cog	Mean	-1.91	-4.22	-3.07	0.0006
		95% CI	-3.24, -0.59	6.19, -2.24	4.82, -1.32	
		p value	0.0047	<0.0001		
	ADCS-ADL	Mean	1.40	4.85	4.60	0.0001
		95% CI	-0.27, 3.07	2.31, 7.40	2.24, 6.97	
		p value	0.0995	0.0002		
C	ADAS-cog	Mean	-1.77	-4.08	-3.17	0.0039
		95% CI	-3.18, -0.37	-6.07, -2.08	-5.31, -1.02	
		p value	0.0132	0.0001		
	ADCS-ADL	Mean	1.82	5.27	4.38	0.0026
		95% CI	0.08, 3.55	2.70, 7.84	1.53, 7.22	
		p value	0.0406	0.0001		

Supplementary Table 5a

Comparison of volumetric MRI outcomes according to CDR severity at baseline. Differences are shown with respect to the control change from baseline at 78 weeks as specified for comparisons A and B. Two parallel implementations of sequential tests were prespecified to examine LMTM 100 mg twice a day as monotherapy compared with the control arm as randomized (comparison A), and LMTM 4 mg twice a day as monotherapy compared with 4 mg twice a day as add-on to existing Alzheimer's disease treatments (comparison B).

		Comparison A				Comparison B			
		**Baseline**	**Change from baseline for 4 mg twice a day, as randomized (n=388)**	**Difference for 100 mg twice a day, as monotherapy (n=82)**	***p* value**	**Baseline**	**Change from baseline for 4 mg twice a day, as add-on (n=316)**	**Difference for 4 mg twice a day, as monotherapy (n=80)**	***p* value**
**CDR 0.5**									
LVV (cm^3)	Mean	48.03	6.84	-2.67	0.0003	49.51	7.65	-2.88	<0.0001
	95% CI	45.21, 50.85	6.11, 7.56	-4.13, -1.22		46.39, 52.63	6.85, 8.46	-4.16, -1.59	
WBV (cm^3)	Mean	980	-20.46	6.37	0.0013	980	-22.46	7.26	<0.0001
	95% CI	967, 993	-22.30, -20.09	2.49, 10.25		965, 995	-24.52, -20.40	3.81,10.70	
HV (mm^3)	Mean	3106	-119	20	0.0705	3088	-131	40	0.0001
	95% CI	3036, 3176	-130, -109	-2, 43		3011, 3165	-143, -119	20, 60	
**CDR 1.0**									
LVV (cm^3)	Mean	50.89	8.24	-2.48	0.0871	52.57	8.52	-3.15	0.0186
	95% CI	46.62, 55.16	7.20, 9.29	-5.31, -0.36		48.04, 57.10	7.45, 9.60	-5.77, -0.53	
WBV (cm^3)	Mean	955	-21.83	6.49	0.0002	959	-23.51	7.77	<0.0001
	95% CI	763, 1147	-23.37, -20.29	3.03, 9.96		939, 979	-25.18, -21.84	4.64,10.90	
HV (mm^3)	Mean	3008	-121	1	0.9648	2877, 3093	-125	46	0.0106
	95% CI	2905, 3111	-135, -108	-38, 40			-139, -111	11, 82	

Supplementary Table 5b.

Volumetric MRI outcomes comparing LMTM 100 mg twice a day as monotherapy with 100 mg twice a day as add-on to existing AD treatments (Comparison C).

		Comparison C			
		**Baseline**	**Change from baseline for 100 mg twice a day, as add-on (n=388)**	**Difference for 100 mg twice a day, as monotherapy (n=82)**	***p* value**
**CDR 0.5**					
LVV (cm^3)	Mean	44.47	7.20	-3.04	<0.0001
	95% CI	43.52, 45.52	6.33, 8.07	-4.31, -1.76	
WBV (cm^3)	Mean	975	-22.33	8.24	<0.0001
	95% CI	826, 1124	-24.56, -20.09	4.75, 11.73	
HV (mm^3)	Mean	3093	-140	41	0.0001
	95% CI	3012, 3174	-153, -127	21, 61	
**CDR 1.0**					
LVV (cm^3)	Mean	53.44	8.57	-2.80	0.0364
	95% CI	49.00, 57.88	7.32, 9.82	-5.42, -0.18	
WBV (cm^3)	Mean	960	-26.97	7.99	0.0326
	95% CI	940, 980	-30.28, -23.65	0.75, 15.23	
HV (mm^3)	Mean	2881	-140	20	0.2854
	95% CI	2767, 2995	-157, -124	-17, 57	

Supplementary Table 6.

Mean (± SD) ^{18}F-FDG-PET SUVR in left and right angular gyrus and inferior temporal gyrus normalized with respect to pons in all patients, patients randomized to receive LMTM as add-on to standard AD treatments, and patients randomized to receive LMTM as monotherapy. Comparisons are with respect to mild AD, MCI, and normal elderly controls as reported by Landau et al. [24].

	TRx-237-005			Landau *et al.* (2011)		
	**All**	**LMTM as add-on therapy**	**LMTM as mono-therapy**	**ADNI-AD**	**ADNI-MCI**	**ADNI-Normal**
**Left temporal**	0.93 ± 0.16	0.92 ± 0.16	0.93 ± 0.17	1.04 ± 0.16	1.16 ± 0.14	1.23 ± 0.13
***p*-value (monotherapy vs ADNI)**				0.0032	<0.0001	<0.0001
**Right temporal**	0.98 ± 0.18	0.97 ± 0.17	0.99 ± 0.17	1.06 ± 0.17	1.16 ± 0.12	1.22 ± 0.11
***p*-value (monotherapy vs ADNI)**				0.0163	<0.0001	<0.0001
**Left angular**	0.98 ± 0.20	0.99 ± 0.20	0.98 ± 0.20	1.06 ± 0.18	1.19 ± 0.15	1.29 ± 0.15
***p*-value (monotherapy vs ADNI)**				0.0066	<0.0001	<0.0001
**Right angular**	0.99 ± 0.20	0.97 ± 0.20	0.98 ± 0.19	1.07 ± 0.17	1.20 ± 0.15	1.29 ± 0.14
***p*-value (monotherapy vs ADNI)**				0.0066	<0.0001	<0.0001

Supplementary Table 7.

Primary analysis model for change in ADAS-cog augmented to include two additional rate-correction terms as *baseline-whole-brain-volume x visit* (WBV) and either *baseline-basalis-volume x visit* (NBM) or *baseline-accumbens-volume x visit* (ACC).

		Comparison A (ADAS-cog)			Comparison B (ADAS-cog)			Comparison C (ADAS-cog)		
		**Change from baseline for 4 mg twice a day, as randomized (n=388)**	**Difference for 100 mg twice a day, as monotherapy (n=76)**	***p* value**	**Change from baseline for 4 mg twice a day, as add-on (n=309)**	**Difference for 4 mg twice a day, as monotherapy (n=79)**	***p* value**	**Change from baseline for 100 mg twice a day, as add-on (n=297)**	**Difference for 100 mg twice a day, as monotherapy (n=79)**	***p* value**
WBV & NBM	Mean	6.35	-2.50	0.0378	7.09	-3.64	0.0010	7.70	-3.85	0.0005
	95% CI	5.32, 7.39	-4.85, -0.14		5.96, 8.20	-5.80,-1.48		6.47, 8.94	-6.01, -1.68	
WBV & ACC	Mean	6.96	-2.06	0.0846	6.96	-3.17	0.0038	7.75	-3.48	0.0015
	95% CI	5.85, 8.07	-4.40, 0.28		5.85, 8.07	-5.31,-1.02		6.53, 8.97	-5.63, -1.33	

Supplementary Fig. 1.

Comparison of decline on the ADAS-cog$_{11}$ scale with recent Phase III trials [25, 26] and untreated patients with MMSE 20-26 on the ADAS-cog$_{13}$ scale with scores rescaled by 70/85 (http://adni.loni.usc.edu/data-samples accessed on 12 Oct 2016)

c

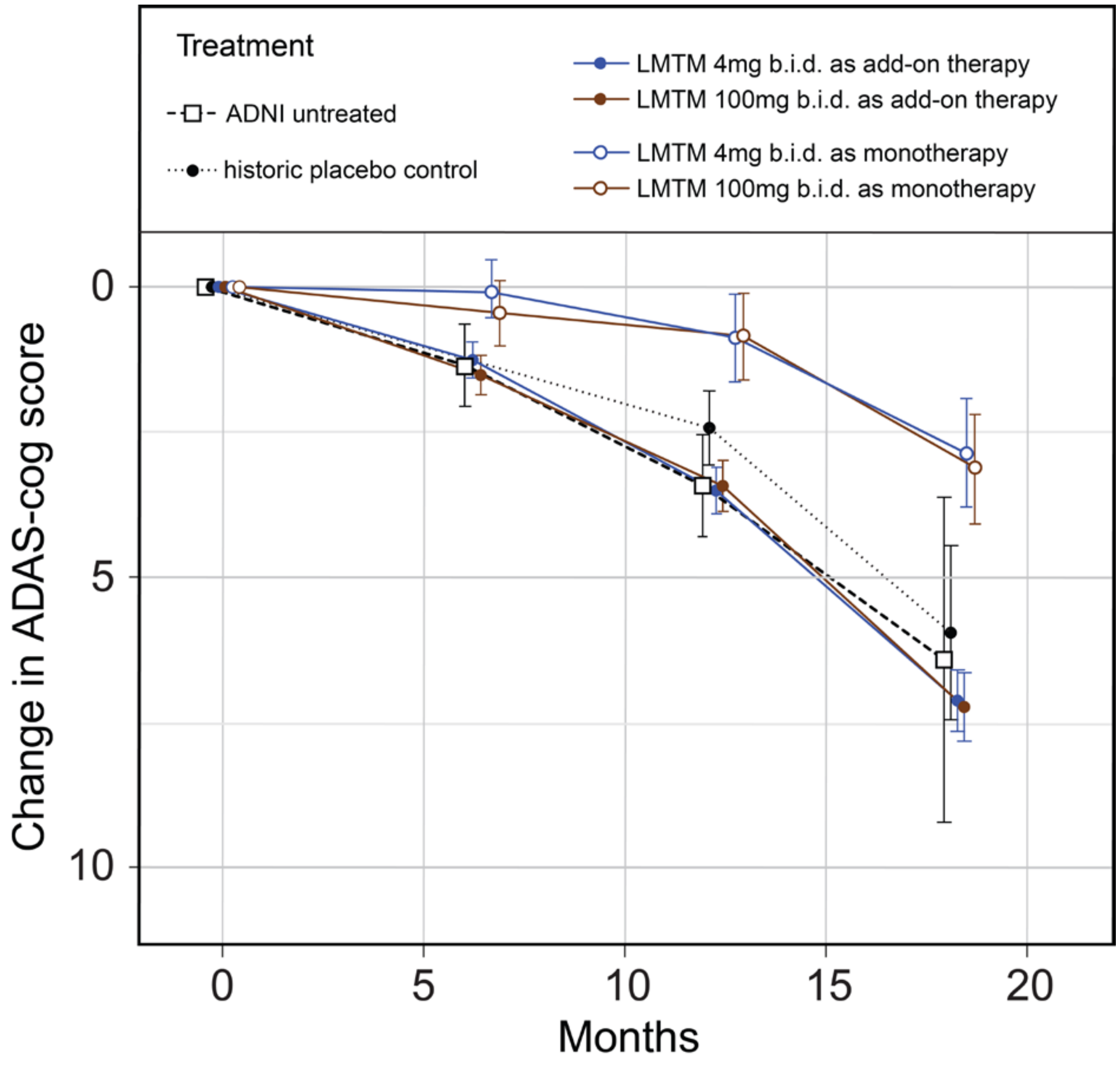

Supplement: Supplementary Material [file jad-61-jad170560-s001.pdf]
