# Supplementary material for: Potential of Low Dose Leuco-Methylthioninium Bis(Hydromethanesulphonate) (LMTM) Monotherapy for Treatment of Mild Alzheimer’s Disease: Cohort Analysis as Modified Primary Outcome in a Phase III Clinical Trial
Source: J Alzheimers Dis. 2017 Nov 28;61(1):435–57. doi: 10.3233/JAD-170560 (PMC5734125; doi:10.3233/JAD-170560)
Supplement: Supplementary Material [file jad-61-jad170560-s002.pdf]

CLINICAL STUDY PROTOCOL

Randomized, Double-Blind, Placebo-Controlled, Parallel-Group, 18-Month Safety and Efficacy Study of Leuco-methylthioninium bis(hydromethanesulfonate) in Subjects with Mild Alzheimer's Disease

STUDY CODE: TRx-237-005

STUDY PHASE: 3

VERSION 7.0 DATED 7 JULY 2015

Previous Versions:

VERSION 6.0 DATED 4 JUNE 2015

VERSION 5.0 DATED 15 MAY 2014

VERSION 4.1 DATED 6 DECEMBER 2013

VERSION 4.0 DATED 30 OCTOBER 2013

VERSION 3.0 DATED 21 AUGUST 2013

VERSION 2.2 DATED 19 MARCH 2013

VERSION 2.1 DATED 12 DECEMBER 2012

VERSION 2.0 DATED 24 OCTOBER 2012

VERSION 1.1 DATED 24 SEPTEMBER 2012

VERSION 1.0 DATED 13 JULY 2012

TauRx Therapeutics Ltd.
Liberty Building
Foresterhill Road
Aberdeen AB25 2ZP
Scotland, UK
Tel: +44 1224 438550
Fax: +44 1224 555173

TauRx Therapeutics Ltd.
3 Shenton Way, #21-04
Shenton House
Singapore 068805
Republic of Singapore

THIS PROTOCOL IS A CONFIDENTIAL DOCUMENT

This Protocol is the property of TauRx Therapeutics Limited. The information within it is confidential and is provided to you, for review by you, your staff, and applicable Ethics Committees. The Protocol must be kept in a confidential manner and must be returned to TauRx upon request. No part of this document may be reproduced in any form without permission from TauRx. By accepting this document, you agree that the information contained therein will not be disclosed to a third party without written authorization from TauRx.

1. PROTOCOL APPROVAL, RESPONSIBLE PERSONNEL, AND INVESTIGATOR SIGNATURES

1.1. Protocol Approval

Sponsor Signatory:

Claude Wischik, MD, PhD
Executive Chairman

TauRx Therapeutics Ltd
Liberty Building
Foresterhill
Aberdeen
AB25 2ZP
Telephone:+ 44 1224 555191
Mobile: + 44 7779 114429

Claude Wischik 8/7/15

Signature Date

Pharmaceutical Physician:

Jiri Hardlund, MD
Chief Medical Officer

TauRx Therapeutics Ltd
Liberty Building
Foresterhill
Aberdeen
AB25 2ZP
Telephone:+ 44 1224 438543
Mobile: + 44 7825 280632

08 JUL 2015

Signature Date

Statistician:

Charles S. Davis, PhD

CSD Biostatistics, Inc.
1005 W. Soft Wind Place
Tucson, AZ 85737
United States
Telephone: +1 520 544 6098
Mobile: +1 858 345 7250

Charles S. Davis 08 July 2015

Signature Date

1.2. Responsible Personnel

<table>
<tr><td>TauRx Global Project Lead for TRx-237-005
Sean Neville
Liberty Building
Foresterhill Road
Aberdeen AB25 2ZP
Scotland, UK
Tel: +44 1224 438578
Fax: +44 1224 555173
Mobile: +44 7867 743 890
E-mail: s.neville@taurx.com</td><td>Global Lead Medical Monitor
Meera Jessani, MD
Worldwide Clinical Trials
1000 Continental Drive
King of Prussia, PA 19406
United States
Tel: +1 610 964 2012
Fax: +1 610 225 0050
Mobile: +1 310 728 5684
E-mail: meera.jessani@wwctrials.com</td></tr>
<tr><td>TauRx Head of Safety and Medical Monitoring
Dr Jiri Hardlund
Liberty Building,
Foresterhill Road
Aberdeen AB25 2ZP
Scotland, UK
Tel: +44 1224 438543
Fax: +44 1224 555173
Mobile: +44 7825 280632
E-mail: JHH@taurx.com</td><td>Pharmacovigilance
Andrew Monaghan, BSc PhD
Director, Global Pharmacovigilance
Isaac Newton Centre, Nottingham Science Park
Nottingham, NG7 2RH, UK
Tel: +44 (0)115 956 7711
Fax: +44 (0)115 922 0960
Mobile: +44 (0)7771 858142
Email: Andrew.monaghan@wwctrials.com</td></tr>
<tr><td>Central Laboratory
Covance, Inc.
Americas
8211 SciCor Drive
Indianapolis, IN 46214-2985
United States
Tel: +1 317 271 1200
Fax: +1 317 273 4030

Europe
7 rue Marcinhes
1217 Geneva
Meyrin Switzerland
Tel: +41 58 822 7000
Fax: +41 58 822 6999

Australia
1 International Business Park
#05-12A/B The Synergy
Singapore 609917
Tel: 65 6560 8793
Fax: 65 6565 5901</td><td rowspan="3">Imaging
MRI Acquisition and Site Interaction
BioClinica, Inc.
100 Overlook Center
Princeton, NJ 08540
United States
Tel: +1 415-817-8945

Oversight and Central Blinded Readers
RadMD, LLC
4920 York Rd, Suite 2EE
Buckingham, PA 18912
Tel: +1 215 348 5644
Fax: +1 610 482 9332
E-mail: kshamsi@rad-md.net

Positron Emission Tomography (PET)
Molecular NeuroImaging LLC and Institute for Neurodegenerative Disorders
60 Temple Street, Suite 8A
New Haven, CT 06510
United States
Tel: +1 203 401 4393
Fax: +1 203 401 4304

Analyses of MRI Data
BioClinica, Inc.
100 Overlook Center
Princeton, NJ 08540
United States
Tel: +1 415-817-8945</td></tr>
<tr><td>MT Concentrations
University of Aberdeen GLP Test Facility
Meston Building
Old Aberdeen
Aberdeen AB24 3UE, UK
Tel: +44 (0) 1224 272945
Fax: +44 (0) 1224 272921</td></tr>
<tr><td>Data Management and Statistics
SynteractHCR, Inc.
5759 Fleet Street, Suite 100
Carlsbad, CA 92008
United States
Tel: + 1 760 268 8200</td></tr>
<tr><td>Study Monitor and Project Management
Worldwide Clinical Trials Limited
2nd Floor, 172 Tottenham Court Rd
London Q1T 7NS, UK
Tel: +44 20 7121 6160</td><td>ECG
BioClinica, Inc.
100 Overlook Center
Princeton, NJ 08540
United States
Tel: +1 301 795 2500</td></tr>
</table>

<table>
<tr><td>IWRS
BioClinica, Inc.
800 Adams Ave
Audubon, PA 19403
United States
Tel: +1 484 928 6736</td><td rowspan="2">Secondary Monitoring of Data and Documentation
Institute for Complex Systems and Mathematical Biology
University of Aberdeen
King’s College
Old Aberdeen AB24 3UE, UK

Exp-e-Data (UK) Limited
Wedale House
Church Wynd
Stow
Selkirkshire
TD1 2QU UK</td></tr>
<tr><td>Statistical Analysis of Pharmacokinetic Data
Institute for Clinical Pharmacodynamics
43 British American Blvd.
Latham, NY 12110
United States</td></tr>
</table>

1.3. Investigator Signature Sheet

By signing below, I agree to the conditions relating to this study as set out in this protocol (TRx-237-005 dated 7 July 2015).

I agree to conduct this study according to Good Clinical Practice (ICH GCP) and applicable regulatory requirements.

I fully understand that any changes instituted by me without previous discussion with TauRx or their designated representative constitute a violation of the protocol.

I agree to adhere to the protocol in all circumstances other than where necessary to protect the well-being of the patient/subject.

I will ensure that the drugs supplied by TauRx will be used only for administration to subjects enrolled in this study and for no other purpose.

Study Site Principal Investigator's Name, Title, Address and Contact Information:

____

____

____

____

____

____

Signature: ____ Date: __________________

SYNOPSIS

Name of Sponsor / Company:<br>**TauRx Therapeutics Ltd (TauRx)**	
**Name of Finished Product:**<br>**Leuco-methylthioninium bis(hydromethanesulfonate) (LMTM, TRx0237) Tablets, 75 mg, 100 mg**	
**Name of Active Ingredient:**<br>**Methylthioninium (MT)**	
**Number and Title of Study:** TRx-237-005: Randomized, Double-Blind, Placebo-Controlled, Parallel-Group, 18-Month Safety and Efficacy Study of Leuco-methylthioninium bis(hydromethanesulfonate) in Subjects with Mild Alzheimer's Disease	
**Study Site(s):** Approximately 100-150 study sites in the Americas, Europe, and Australia	
**Study Duration:**<br>The total duration of participation for an individual subject will be up to 88 weeks, including a Screening period of up to 6 weeks (42 days), a double-blind treatment study of 78 weeks, and a post-treatment assessment 4 weeks after completion of randomized treatment. It is anticipated that the study will have an overall duration of at least 34 months, depending on recruitment rate. In addition, subjects who complete the study, including the off-treatment follow-up visit, may be offered an opportunity to subsequently receive treatment with LMTM in a separate open-label extension study.	**Phase of Development:**<br>Phase 3

Objectives:

Primary:

1. To demonstrate clinical efficacy of leuco-methylthioninium bis(hydromethanesulfonate) (also known as LMTM, TRx0237) in mild Alzheimer's disease based on change from baseline on the following co-primary endpoints:
   - Alzheimer's Disease Assessment Scale – Cognitive Subscale (ADAS-cog_{11})
     and
   - Alzheimer's Disease Cooperative Study – Activities of Daily Living (ADCS-ADL_{23})
2. To assess the safety and tolerability of LMTM 200 mg/day given for up to 78 weeks

Secondary:

3. To further demonstrate disease modification based on the following key secondary endpoint:
   - Reduction in decline in whole brain volume (WBV) using change from Baseline as measured by the Brain Boundary Shift Integral (BBSI) by MRI imaging
4. To evaluate the effect of LMTM on a global measure, Alzheimer's Disease Cooperative Study – Clinical Global Impression of Change (ADCS-CGIC) – independently rated
5. To evaluate the effects of LMTM on other aspects of Alzheimer's disease including cognition (Mini-Mental Status Examination, MMSE), behavior (Neuropsychiatric Inventory, NPI), and mood (Montgomery-Asberg Depression Rating Scale, MADRS)

Exploratory:

6. To determine the effects of LMTM on Alzheimer's disease modification by showing retardation of the rate of brain atrophy by reducing the expected increase in ventricular volume and decline in hippocampal volume as evaluated by MRI
7. To determine the effects of LMTM on reduction in decline in glucose uptake in the temporal lobe on ^{18}F-fluorodeoxyglucose positron emission tomography (FDG-PET) imaging
8. To determine the effects of LMTM on resource utilization using the Resource Utilization in Dementia (RUD) Lite
9. To explore changes in certain cerebrospinal fluid (CSF) biomarkers of Alzheimer's disease (total tau, phospho-tau and $A\beta_{1-42}$) in subjects who are mentally capable of providing their own separate informed consent and specifically agree to have lumbar puncture performed

Name of Sponsor / Company:
TauRx Therapeutics Ltd (TauRx)

Name of Finished Product:
Leuco-methylthioninium bis(hydromethanesulfonate) (LMTM, TRx0237) Tablets, 75 mg, 100 mg

Name of Active Ingredient:
Methylthioninium (MT)

10. To explore the influence of the Apolipoprotein E (ApoE) genotype on the primary and selected secondary outcomes (in subjects by or for whom legally acceptable consent is provided)

Blood will be collected for purposes of population pharmacokinetic analysis. This will be detailed in a separate Statistical Analysis Plan (SAP) and will be reported separately (together with data from other studies).

Study Design:
Multinational, randomized, placebo-controlled, double-blind, parallel-group, 78-week, outpatient study with eight post-baseline on-treatment visits planned (Visits 3 - 10), followed by an off-treatment follow-up visit for all subjects to occur 4 weeks after completion of randomized treatment (Visit 11). Unscheduled visits and housing may occur as needed for assessment.

Number of Subjects:
The target recruitment number is approximately 700 subjects (350 per arm). Subjects who drop out after starting study treatment will not be replaced. Based on the use of a two-sided test at the 5% level of significance and assuming the standard deviation (SD) of the ADAS-cog_{11} change from Baseline to Week 78 to be 8.76 units, this sample size will provide 90% power to detect a mean difference between groups of 2.16 units, under the assumption that the effect size corresponds to a 52% reduction in the expected rate of decline and assuming that the placebo decline is 4.15 ± 8.76 units. This sample size also has 90% power to detect a 38% reduction in the expected rate of decline on the ADCS-ADL_{23} assuming a placebo decline of 9.21 units and SD of 14.06 units.

Subject Population:

***Inclusion Criteria*.**

1. Diagnosis according to the National Institute on Aging (NIA) and Alzheimer’s Association (AA) criteria of:
   - All cause dementia
     and
   - Probable Alzheimer’s disease
2. Clinical Dementia Rating (CDR) total score of 0.5 or 1 (mild) and MMSE score of 20-26 (inclusive) at Screening
3. Age < 90 years at Screening
4. Modified Hachinski ischemic score of ≤ 4 at Screening
5. Females must meet one of the following:
   - Surgically sterile (hysterectomy, bilateral salpingectomy / oophorectomy) for at least 6 months minimum
   - Have undergone bilateral tubal occlusion / ligation at least 6 months prior
   - Post-menopausal for at least 1 year
   - Using adequate contraception (a barrier method [such as condom, diaphragm or cervical/vault cap] with spermicidal foam, gel, film, cream, or suppository; intrauterine device [IUD] or system, or oral or long-acting injected or implanted hormonal contraceptives for at least 3 months prior to Baseline; or vasectomized partner [with the appropriate post-vasectomy documentation of the absence of spermatozoa in the ejaculate]) or true abstinence (when this is in line with the preferred and usual lifestyle of the subject); subjects must be competent to use adequate contraception and to agree to continue to maintain adequate contraception throughout participation in the study
     OR
     In Italy, have avoided a pregnancy for at least 3 months prior to Baseline and accept to avoid a pregnancy throughout participation in the study
6. Subject and/or, in the case of reduced decision-making capacity, legally acceptable representative(s) consistent with national law is/are able to read, understand, and provide written informed consent in the designated language of the study site

**Name of Sponsor / Company:**<br>**TauRx Therapeutics Ltd (TauRx)**
**Name of Finished Product:**<br>**Leuco-methylthioninium bis(hydromethanesulfonate) (LMTM, TRx0237) Tablets, 75 mg, 100 mg**
**Name of Active Ingredient:**<br>**Methylthioninium (MT)**

- In Germany, subjects must be able to provide their own written informed consent (see Sections 14 and 15)

7. Has one or more identified adult caregivers who meet the following criteria:
    - Either lives with the subject or sees the subject on average for ≥ 2 hours/day ≥ 3 days/week, or in the investigator's opinion, the extent of contact is sufficient to provide meaningful assessment of changes in subject behavior and function over time and provide information on safety and tolerability
    - Is willing to provide written informed consent for his/her own participation
    - Is able to read, understand, and speak the designated language at the study site
    - Agrees to accompany the subject to each study visit
    - Is able to verify daily compliance with study drug
8. If currently taking an acetylcholinesterase inhibitor (AChEI), *i.e.*, donepezil, galantamine, or rivastigmine, and/or memantine, at the time of Screening
    - The subject must have been taking such medication(s) for ≥ 3 months
    - The current dosage regimen and dosage form must be within the locally approved dose range and must have remained stable for ≥ 6 weeks
    - It must be planned that the dosage regimen will remain stable throughout participation in the study

    Subjects not being treated with an AChEI or memantine (for ≥ 6 weeks before Screening) may also be enrolled if initiation of an AChEI or memantine is not planned for the time period during which the subject will be participating in this study
9. Able to comply with the study procedures in the view of the investigator

Exclusion Criteria.

1. Significant CNS disorder other than Alzheimer's disease, *e.g.*, Lewy body dementia, Parkinson's disease, multiple sclerosis, progressive supranuclear palsy, hydrocephalus, Huntington's disease, any condition directly or indirectly caused by Transmissible Spongiform Encephalopathy (TSE), Creutzfeldt-Jakob Disease (CJD), variant Creutzfeldt-Jakob Disease (vCJD), or new variant Creutzfeldt-Jakob Disease (nvCJD)
2. Significant intracranial focal or vascular pathology seen on brain MRI scan within a maximum of 42 days before Baseline that would, based on the independent reviewer imaging evaluation, lead to a diagnosis other than probable Alzheimer's disease or that puts the subject at risk of Amyloid Related Imaging Abnormalities (ARIA), including:
    - Large confluent white matter hyperintense lesions (*i.e.*, Fazekas score of 3)
    - Other focal brain lesion(s) judged clinically relevant by the investigator
    - A single area of superficial siderosis
    - > 4 Cerebral microhemorrhages (regardless of their anatomical location or diagnostic characterization as "possible" or "definite")
    - Evidence of a prior macrohemorrhage
3. Clinical evidence or history of any of the following within specified period prior to Baseline:
    - Cerebrovascular accident (2 years)
    - Transient ischemic attack (6 months)
    - Significant head injury with associated loss of consciousness, skull fracture or persisting cognitive impairment (2 years)
    - Other unexplained or recurrent loss of consciousness ≥ 15 minutes (2 years)
4. Epilepsy (a single prior seizure is considered acceptable)
5. Diagnostic and Statistical Manual of Mental Disorders, Fourth Edition – Text Revision (DSM IV-TR) criteria met

**Name of Sponsor / Company:**<br>**TauRx Therapeutics Ltd (TauRx)**
**Name of Finished Product:**<br>**Leuco-methylthioninium bis(hydromethanesulfonate) (LMTM, TRx0237) Tablets, 75 mg, 100 mg**
**Name of Active Ingredient:**<br>**Methylthioninium (MT)**

(and not subsequently revised) for any of the following within specified period:

- Major depressive disorder (current)
- Schizophrenia (lifetime)
- Other psychotic disorders, bipolar disorder (within the past 5 years), or substance (including alcohol) related disorders (within the past 2 years)

6. Metal implants in the head (except dental), pacemaker, cochlear implants, or any other non-removable items that are contraindications to MR imaging; MR compatible prosthetics, clips, stents, or any other device proven to be compatible will be allowed
7. Resides in hospital or moderate to high dependency continuous care facility (residence in low grade assisted living facility where there is sufficient autonomy to permit valid evaluation of activities of daily living is allowed so long as it is not mandated by an order issued either by the judicial or the administrative authorities)
8. History of swallowing difficulties (note: study drug should be swallowed whole and MUST NOT be broken, crushed, chewed, or dissolved in fluids prior to ingestion)
9. Pregnant or breastfeeding
10. Glucose-6-phosphate dehydrogenase (G6PD) deficiency
11. History of significant hematological abnormality or current acute or chronic clinically significant abnormality, including:
    - History of hereditary or acquired methemoglobinemia or baseline measurement of methemoglobin (MetHb) > 2.0% (confirmed on repeat)
    - History of hemoglobinopathy, myelodysplastic syndrome, hemolytic anemia, or splenectomy
    - Screening hemoglobin value (confirmed upon repeat) below age/sex appropriate lower limit of the central laboratory normal range.

    Subjects in whom folate is < 4.0 ng/mL may be entered into the study provided folate supplementation (approximately 1mg/day) is initiated and maintained for the duration of the study (see Section 4.7.8).

    Subjects in whom Vitamin B_{12} is < 150 pg/mL should be evaluated and supplemented as appropriate prior to the initiation of study drug (see Section 4.7.8). If review and correction can be achieved within the 42-day screening window, the subject may be entered into the study; otherwise the subject must be reconsented and rescreened after the deficit has been corrected.
12. Abnormal serum chemistry laboratory value at Screening deemed to be clinically relevant by the investigator, *e.g.*, those considered to have the potential to increase the risk associated with study participation or administration of investigational product and, in the judgment of the investigator, would make the subject inappropriate for entry into this study. In addition, subjects with either of the following abnormalities must be excluded:
    - Creatinine clearance < 30 mL/min at Screening, estimated by the central laboratory according to the Cockcroft and Gault equation
    - Thyroid stimulating hormone (TSH) above laboratory normal range (subject may be treated and rescreened after 3 months)
13. Clinically significant cardiovascular disease or abnormal assessments (in the opinion of the investigator) such as:
    - Hospitalization for acute coronary syndrome (acute myocardial infarction or unstable angina) or symptoms consistent with angina pectoris, within the 12 months preceding Baseline
    - Signs or symptoms of clinical heart failure within the 12 months preceding Baseline
    - Evidence of uncontrolled atrial fibrillation on Screening ECG or history of atrial fibrillation that is not currently controlled (heart rate ≥ 85 bpm and/or inappropriate anticoagulation) or where the QT interval cannot in the opinion of the investigator be assessed by triplicate ECGs taken within an approximate 2- to

**Name of Sponsor / Company:**<br>**TauRx Therapeutics Ltd (TauRx)**
**Name of Finished Product:**<br>**Leuco-methylthioninium bis(hydromethanesulfonate) (LMTM, TRx0237) Tablets, 75 mg, 100 mg**
**Name of Active Ingredient:**<br>**Methylthioninium (MT)**

5-minute interval (if better control of the heart rate and/or of anticoagulation can be achieved after adequate treatment, subject may be entered into the study if still within the 42-day window, or else the subject must be reconsented and rescreened). A cardiology consult should be sought for further ECG evaluation (especially in subjects with left bundle branch block) if deemed necessary by the investigator.

- QTcF (based on mean of three triplicate ECGs, QT corrected for heart rate using Fridericia’s formula) at Screening > 460 msec in males or > 470 msec in females, or low or flat T waves making measurement of QT interval unreliable
- Recent history of poorly controlled hypertension, systolic blood pressure > 160 mmHg, or diastolic blood pressure > 100 mmHg, after 5 minutes in a seated position at Screening
- Hypotension: systolic blood pressure < 100 mmHg after 5 minutes in a seated position at Screening
- Heart rate < 48 bpm or > 96 bpm by measurement of vital signs (after 5 minutes in a seated position) or by ECG at Screening

14. Preexisting or current signs or symptoms of respiratory failure, *e.g.*, caused by chronic obstructive pulmonary disease, bronchial asthma, lung fibrosis, or other disease
    - Subjects with previously diagnosed moderate to severe sleep apnea not adequately controlled (in the opinion of the investigator) should be excluded
15. Concurrent acute or chronic clinically significant (in the opinion of the investigator) immunologic, hepatic (such as presence of encephalopathy or ascites), or endocrine disease (not adequately treated) and/or other unstable or major disease other than Alzheimer’s disease
    - Subjects with hepatitis or primary biliary cirrhosis should be excluded
    - Human T-Cell Lymphocytic Virus Type III (HTLV-III), Lymphadenopathy Associated Virus (LAV), any mutants or derivatives of HLTV-III or LAV, any condition associated with Acquired Immunodeficiency Syndrome or similar condition however named
16. Diagnosis of cancer within the past 2 years prior to Baseline (other than basal cell or squamous cell skin cancer or Stage 1 prostate cancer) unless treatment has resulted in complete freedom from disease for at least 2 years
17. Prior intolerance or hypersensitivity to MT-containing drug, similar organic dyes, or any of the excipients
18. Treatment currently or within 3 months before Baseline with any of the following medications (unless otherwise noted; see Section 4.7.4):
    - Tacrine
    - Antipsychotics
        - Clozapine, olanzapine (and there is no intent to initiate therapy during the course of the study)
        - Other antipsychotics are allowable provided they have not been initiated within 3 months before Baseline and preferably at a stable dose and regimen
    - Carbamazepine, primidone
    - Drugs for which there is a warning or precaution in the labeling about methemoglobinemia at approved doses (*e.g.*, dapsone, local anesthetics such as benzocaine used chronically, primaquine and related antimalarials)
19. Current or prior participation in a clinical trial as follows:
    - Clinical trial of a product for cognition prior to Screening in which the last dose was received within 90 days prior to Screening unless confirmed to have been randomized to placebo
    - A clinical trial of a drug, biologic, device, or medical food in which the last dose was received within 28 days prior to Baseline

Name of Sponsor / Company:
TauRx Therapeutics Ltd (TauRx)

Name of Finished Product:
Leuco-methylthioninium bis(hydromethanesulfonate) (LMTM, TRx0237) Tablets, 75 mg, 100 mg

Name of Active Ingredient:
Methylthioninium (MT)

Dose/Route/Regimen:
Subjects will be randomized 1:1 stratified by geographic region (the Americas or Europe/Australia), use of an AChEI and/or memantine (current ongoing use or not ongoing use), and screening severity (CDR 0.5 or 1) to one of the following oral treatment groups to be given for 78 weeks:

- LMTM 200 mg/day group: LMTM 100 mg twice daily (*b.i.d.*) (one 100-mg tablet in the morning and one 100-mg tablet in the evening) [n=350]
- Placebo group: Placebo twice daily (*b.i.d.*) (one LMTM 4-mg tablet in the morning and one LMTM 4-mg tablet in the evening) [n=350]

A 75-mg tablet is also provided to allow for dose reduction. The placebo group will receive low dose LMTM as a urinary and fecal colorant to maintain blinding. All tablets are of matching appearance.

Interruption of dosing for up to (maximum duration on any one occasion) 30 days during the first 6 months of treatment and/or 14 days during the second 12 months of treatment or 50-mg reduction of daily dose (up to a maximum of two such reductions) may be allowed if the investigator determines this is indicated, *e.g.*, tolerability concerns or laboratory abnormalities. In the event of continued poor tolerance subjects should be withdrawn from treatment (but encouraged to return for the balance of the scheduled study visits).

Methodology:
Following provision of written informed consent by the subject and/or his/her legal representative(s), consistent with national law, eligibility for enrollment will be assessed initially at one or more Screening visits, which will occur no earlier than 42 days before the Baseline visit. MRI images acquired will be sent to an imaging core laboratory where they will be evaluated by a trained technologist for acceptable quality and then reviewed by an independent neuroradiologist (reader) who is trained in the evaluations and is not involved in the clinical conduct of the study to confirm eligibility of the subject. The reader's assessment will be communicated to the site within 5 business days of image transfer to the imaging core laboratory (and resolution of any quality issues).

All Baseline safety assessments will be completed by Baseline (Visit 2, Day 1) (designated pre-dose). For the subset of subjects who consent to undergo lumbar puncture, the procedure may be performed any time pre-dose so long as all screening procedures have been completed and subject eligibility and willingness to continue have been confirmed. Staging and Baseline efficacy assessments can be made on the day before randomization and dosing (including ratings of CDR, MMSE, ADAS-cog_{11}, ADCS-CGIC, ADCS-ADL_{23}, NPI, MADRS, and RUD Lite). Subjects will be randomized and the first dose of study drug will be administered under supervised conditions (dosing may be held subject to eligibility review based on the local interpretation of triplicate ECGs by the investigator and on cardiology consultation if deemed necessary by the investigator). All subjects will be observed for at least 4 hours. During this time, oral temperature and respiratory rate will be measured hourly until discharge. Seated and standing blood pressure and pulse will be measured after approximately 2 hours, methemoglobin and oxygen saturation will be measured after approximately 2.5 hours, a triplicate ECG recording will be made after approximately 3 hours, and immediately thereafter, targeted physical and neurological examinations will be performed. Potential signs and symptoms of serotonin toxicity will be rated and potential cases of serotonin syndrome identified for all subjects (see Section 24.2); an assessment is to be completed just prior to discharge from the clinic. Blood will be collected pre-dose and approximately 3.5 hours post-dose for analysis of MT (restricted to sites that have a refrigerated centrifuge and appropriate freezer capacity). Subjects will be discharged from the clinic provided there are no signs or symptoms of impending serotonin toxicity; otherwise, they will continue to be monitored in the clinic and managed as medically appropriate. Upon discharge, all subjects will receive a supply of study drug to take home for use until Visit 4 (Week 6); a compliance check will be made after 2 weeks (Visit 3).

Subjects who are receiving serotonergic medication and their caregivers are to remain in close proximity to a local hospital and remain in telephone contact with the clinic for at least 12 to 14 hours post-dose and reimbursement for accommodations will be made available if requested by the subject and/or his/her caregiver. They will be provided

**Name of Sponsor / Company:**<br>**TauRx Therapeutics Ltd (TauRx)**
**Name of Finished Product:**<br>**Leuco-methylthioninium bis(hydromethanesulfonate) (LMTM, TRx0237) Tablets, 75 mg, 100 mg**
**Name of Active Ingredient:**<br>**Methylthioninium (MT)**

with monitoring instructions, an oral thermometer, and diary. Temperature should be monitored three times a day (in the morning, afternoon, and evening) until 72 hours after the first dose and recorded in the diary to be returned at Visit 3 (Week 2). Caregivers will be contacted by telephone 5–7, >7–14, >14–24, 44–52, and 68–76 hours post-dose (with a minimum of 1 hour between contacts) and queried for the presence of signs and symptoms of serotonin toxicity in the subjects (see Section 24.3); they will be instructed to have the temperature diary available during these telephone contacts. If indicated, more frequent contacts will be made and the site will assume responsibility for clinical review and hospital referral if indicated.

Subjects will be treated with study drug for 78 weeks (18 months) on an outpatient basis. On-treatment, post-Baseline study visits will occur at time points approximately 2, 6, 13, 26, 39, 52, 65, and 78 weeks after Baseline. Caregivers will be contacted by telephone at approximately 9, 19, 32, 45, 58, and 71 weeks. In the event of tolerability problems subjects and/or caregivers will be asked to contact the investigator and an extra visit will be arranged to assess the subject. All subjects are to return for a follow-up visit 4 weeks after the last dose of double-blind study drug. Efficacy and safety assessments are described below.

Subjects who complete the study, including the off-treatment follow-up visit, may be offered an opportunity to subsequently receive treatment with LMTM in a separate open-label extension study.

The trial will be monitored for safety by a Data Safety Monitoring Board (DSMB) throughout its duration.

Assessments:

Efficacy:
Assessments will be made at Baseline (Visit 2, pre-dose) and after 13, 26, 39, 52, 65, and 78 weeks, and at the 4-week off-treatment follow-up visit by assessors/raters who are not involved in the assessment of safety (with the exception of the C-SSRS and Serotonin Toxicity Assessment) using the following instruments:

- ADAS-cog$_{11}$
- ADCS-ADL$_{23}$
- ADCS-CGIC (a separate rater who remains blinded throughout study to results of other efficacy assessments)

The NPI and MADRS will be rated at Baseline (Visit 2, pre-dose) and after 26, 52, and 78 weeks. The MMSE will be rated at Screening, after 26, 52, and 78 weeks of treatment, and at the 4-week off-treatment follow-up visit.

Brain MRI will be performed at Screening/Baseline and will also be evaluated for whole brain, ventricular and hippocampal volumes at Weeks 13, 26, 39, 52, 65, and 78 (or upon early termination). FDG-PET will also be performed at Screening/Baseline and after 39 and 78 weeks of treatment. FDG-PET data will be evaluated by an independent nuclear physician who is experienced in neuro PET and trained on the study endpoints (and who is otherwise not involved in the clinical conduct of the study). Change in whole brain, ventricular and hippocampal volumes, and in temporal lobe FDG-PET will be quantified at the respective imaging core laboratories.

CDR will only be assessed at Screening to determine a CDR total score for severity/inclusion determination.

Safety and Tolerability:
Safety assessments will be performed during Screening to assess subject eligibility for enrollment. For enrolled subjects, safety assessments will be performed at Baseline (pre-dose and post-dose during the 4-hour observation), at 2, 6, 13, 26, 39, 52, 65, and 78 weeks after Baseline, and approximately 4 weeks after the last dose of study drug. Caregivers will be contacted by telephone at approximately 9, 19, 32, 45, 58, and 71 weeks. All adverse events (AEs), vital signs, 12-lead ECGs, methemoglobin and oxygen saturation, clinical laboratory findings, physical and neurological examinations, potential for serotonin toxicity, brain MRI data, and the potential for suicide or self-harm will be assessed according to the following:

- Adverse events will be recorded from the time informed consent is signed and recording will continue

**Name of Sponsor / Company:**<br>**TauRx Therapeutics Ltd (TauRx)**
**Name of Finished Product:**<br>**Leuco-methylthioninium bis(hydromethanesulfonate) (LMTM, TRx0237) Tablets, 75 mg, 100 mg**
**Name of Active Ingredient:**<br>**Methylthioninium (MT)**

throughout the study, including the follow-up visit (Visit 11). Adverse events with an onset after the first dose of study drug or that worsen in intensity or treatment relationship after the first dose will be considered treatment-emergent.

- Oral (sublingual) temperature and respiratory rate will be recorded at Screening and at each clinic visit thereafter (or upon early termination), including the follow-up visit (Visit 11). At Visit 2, oral temperature and respiratory rate will be recorded within 1 hour prior to dosing and hourly following the first dose while subjects are in the clinic (*i.e.*, for at least 4 hours). Upon discharge, if receiving a product with serotonergic potential, subjects/caregivers will continue to monitor temperature three times a day (in the morning, afternoon, and evening) until 72 hours after the first dose of study drug. These measurements are to be recorded in a diary to be returned to the clinic at Visit 3.
- Blood pressure and pulse will be obtained at Screening, within 1 hour before and approximately 2 hours after administration of the first dose of study drug (Visit 2), and at each visit thereafter (or upon early termination), including the follow-up visit (Visit 11). At Screening and on Day 1, two sets of blood pressure and pulse measurements will be made, the first set after the subject has been at rest in a seated position for approximately 5 minutes, and the second set approximately 2 minutes after the subject rises to a standing position. Otherwise, blood pressure and pulse will be measured with the subject in a seated position for at least 5 minutes.
- Height will be measured at Screening only. Body weight will be measured at Screening, Baseline (Visit 2; Pre-dose), and Visits 4 through 10 while on treatment (or upon early termination), and at the follow-up visit (Visit 11).
- Methemoglobin and oxygen saturation will be measured using a pulse co-oximeter at Screening, within 1 hour before and approximately 2.5 hours after administration of the first dose of study drug (Visit 2), and at each visit thereafter (or upon early termination), including the follow-up visit (Visit 11).
- A 12-lead ECG will be obtained in triplicate at Screening; the site may base eligibility determinations on the central interpretation or the results of the local interpretation in subjects with atrial fibrillation or with intraventricular conduction blocks. In subjects with atrial fibrillation and in subjects with intraventricular conduction blocks, a read should be undertaken locally and a local cardiology consult should be sought to decide on eligibility. Data derived from any triplicate ECG will be documented on the eCRF as the average of the three readings. At Visit 2, the ECG will be obtained in triplicate pre-dose and at approximately 3 hours after the first dose (timed to encompass the anticipated time of the peak MT plasma concentration after the initial dose); dosing decisions should be made on the basis of interpretations of the Screening ECG and local interpretations of the pre-dose ECG, with a cardiology consult if deemed necessary by the investigator, in particular in subjects with controlled atrial fibrillation (heart rate $\leq$ 84 bpm and appropriate anticoagulation) and in subjects with intraventricular conduction blocks. At all other visits (or upon early termination), including the follow-up visit (Visit 11), an ECG measurement will be obtained as a single recording, unless there are emergent abnormalities deemed clinically significant by the investigator, in which case triplicate ECGs should be obtained. For subjects with well controlled atrial fibrillation (heart rate $\leq$ 84 bpm and appropriate anticoagulation) at baseline, monitoring by triplicate ECGs at all subsequent visits is mandatory and data documented on the eCRF will be averages of the three readings.
- Standard clinical laboratory testing, including hematology, blood chemistry, and urinalysis will be performed at Screening, Baseline (Visit 2; Pre-dose), Visits 3 through 10 while on treatment (or upon early termination), and at the follow-up visit (Visit 11). Testing for Vitamin B_{12} and folate will be included at each timepoint. TSH will be measured at Screening and Weeks 26, 52, and 78. Clinical laboratory testing at the Screening visit will additionally include G6PD and haptoglobin. A serum pregnancy test will be collected for all women of childbearing potential at Screening and at each subsequent visit (or upon early termination), including the

**Name of Sponsor / Company:**<br>**TauRx Therapeutics Ltd (TauRx)**
**Name of Finished Product:**<br>**Leuco-methylthioninium bis(hydromethanesulfonate) (LMTM, TRx0237) Tablets, 75 mg, 100 mg**
**Name of Active Ingredient:**<br>**Methylthioninium (MT)**

4-week follow-up visit (Visit 11).

- Complete physical and neurological assessments will be performed at Screening. Targeted assessments will be performed to assess potential signs of serotonin toxicity and, as needed, to evaluate adverse events or change in medical history at pre-dose and approximately 3 hours after administration of the first dose of study drug (Visit 2) (following the ECG measurements); these are to be repeated as needed for subjects who remain in the clinic longer than 4 hours. Thereafter, targeted examinations are to be performed at each subsequent visit (or upon early termination), including the 4-week post-treatment follow-up visit (Visit 11).
- Signs and symptoms of potential serotonin toxicity will be rated in the clinic for all subjects at Baseline (Visit 2) and each subsequent visit (or upon early termination), including the follow-up visit (Visit 11). These will be rated using a 20-item Serotonin Toxicity (Syndrome) Diagnostic Interview and Rating Guide (Section 24.2). This will be conducted pre-dose and for at least 4 hours after the first dose of study drug while the subject is in the clinic (Visit 2); an assessment is to be completed just prior to discharge from the clinic. Caregivers of subjects receiving serotonergic medication will be contacted 5–7, >7–14, >14–24, 44–52, and 68–76 hours post-dose (with a minimum of 1 hour between contacts) and queried for the presence of signs and symptoms of serotonin toxicity using the Serotonin Toxicity Telephone Assessment (Section 24.3); if indicated, more frequent contacts will be made.
- Brain MRI will be performed at Screening/Baseline and at Weeks 13, 26, 39, 52, 65, and 78 and evaluated by the central reader for ARIA, including vasogenic edema, macrohemorrhages, microhemorrhages, or superficial siderosis. Information about presence or absence of ARIA will be communicated to the site by the imaging core laboratory within 5 business days of image transfer (and resolution of any imaging queries). The study drug shall be permanently discontinued if an imaging abnormality consistent with vasogenic edema, macrohemorrhage, an area of superficial siderosis, clinically symptomatic new microhemorrhage(s), or >4 clinically asymptomatic new microhemorrhages are seen. If any of these occur, subjects must be re-scanned every 6 weeks after discontinuation of study drug until imaging abnormalities are resolved or stabilized (the latter to be determined based on three follow-up scans).
- The Columbia-Suicide Severity Rating Scale (C-SSRS) will be applied at Screening (Visit 1), after the first dose at Baseline (Visit 2; prior to discharge from the clinic), and at each visit thereafter (or upon early termination), including the follow-up visit (Visit 11), to capture any respective changes.
- At Screening, medications administered within the last 3 months will be recorded and changes in concomitant medication will be recorded at all visits, including the follow-up visit (Visit 11) as well as at each telephone contact.

Other Assessments:

- A rating using the Research Utilization in Dementia (RUD) Lite questionnaire will be made at Baseline (Visit 2, pre-dose) and repeated on-treatment at Weeks 26, 52, and 78 (or upon study termination) by assessors/raters who are not involved in the assessment of safety (with the exception of the C-SSRS and Serotonin Toxicity Assessment).
- Provided the site has a refrigerated centrifuge and adequate capability to reliably freeze samples, blood will also be collected at Baseline (Visit 2; pre-dose and approximately 3.5 hours post-dose) and, to the extent possible, at each subsequent on-treatment visit through Week 78 (approximately 20 minutes after the ECG recording) for purposes of population pharmacokinetic (PK) analysis of MT concentrations. The time of the prior dose and the time of the blood sample will be recorded.
- A single blood sample for genotyping will be obtained only for subjects by or for whom legally acceptable informed consent for this is provided; the blood sample may be collected any time after eligibility for randomization and continued participation in the study has been confirmed.

**Name of Sponsor / Company:**<br>**TauRx Therapeutics Ltd (TauRx)**
**Name of Finished Product:**<br>**Leuco-methylthioninium bis(hydromethanesulfonate) (LMTM, TRx0237) Tablets, 75 mg, 100 mg**
**Name of Active Ingredient:**<br>**Methylthioninium (MT)**

- Cerebrospinal fluid samples will be obtained at designated study sites only from subjects who are mentally capable of providing their own separate informed consent and specifically agree to have lumbar puncture performed. These samples will be obtained using standard lumbar puncture procedures and will be analyzed for specified biomarkers. Baseline CSF samples may be collected any time prior to the first dose of study drug so long as all screening procedures have been completed and subject eligibility and willingness to continue have been confirmed. A subsequent sample will be collected at the end of treatment (Week 78 or upon early termination).

Primary Statistical Analyses:
The primary efficacy analyses will be performed on a Modified Intent-to-Treat (MITT) population which will include all randomized subjects who took at least one dose of study drug and have a Baseline and at least one post-Baseline efficacy measurement. Subjects will be analyzed for efficacy in the treatment group to which they were randomized.

Co-primary endpoints will be analyzed to demonstrate clinical efficacy. The co-primary endpoints are ADAS-cog_{11} and ADCS-ADL_{23}. Both co-primary endpoints must reach statistical significance (two-tailed alpha of 0.05) for LMTM 200 mg/day to be designated as superior to placebo. These will be analyzed as follows:

The primary analyses of the ADAS-cog_{11} and the ADCS-ADL_{23} will be carried out using restricted maximum likelihood based repeated measures linear mixed models with an unstructured covariance matrix in which no data will be imputed. The model for each of these two endpoints will include fixed effects for treatment group (two levels), time (six levels: corresponding to Weeks 13, 26, 39, 52, 65, and 78) and the treatment group by time interaction. Each model will also include fixed effects for geographic region (two levels: Americas, Europe/Australia) and AChEI and/or memantine status at randomization (two levels: current ongoing use or not ongoing use). In addition, the corresponding baseline measure (ADAS-cog_{11} or ADCS-ADL_{23}) will be included as a covariate. CDR is a stratification variable but only to ensure a balanced design for some exploratory analyses and is not a covariate in the primary or secondary analyses. The Kenward and Roger method of calculating the denominator degrees of freedom will be used for the tests of fixed effects. The primary comparison will be based on the modeled change from Baseline at Week 78 (or Week 65 if fewer than 60% of subjects are retained in either treatment arm at Week 78). The difference between the marginal means (least-square means in SAS), with 95% confidence interval and p-value, will be provided for the LMTM treatment group *versus* placebo.

Sensitivity analyses, additional analyses of the primary variables (such as responder analyses and subgroup analyses), and the analyses of secondary and exploratory endpoints are described in the body of the protocol.

Change in whole brain, ventricular, and hippocampal volume using the Brain, Ventricular and Hippocampal Boundary Shift Integral (BBSI, VBSI and HBSI, respectively) will be evaluated to demonstrate a potential disease modification effect in an Imaging MITT population which will include all randomized subjects who took at least one dose of study drug and have a Baseline and at least one post-Baseline MRI imaging measurement of adequate imaging quality. A restricted maximum likelihood based repeated measures linear mixed model with an unstructured covariance matrix includes fixed effects for treatment group (two levels), time (six levels: corresponding to Weeks 13, 26, 39, 52, 65, and 78), the treatment group by time interaction, and the relevant Baseline volume. The Kenward and Roger method of calculating the denominator degrees of freedom will be used for the tests of fixed effects. The primary comparison will be based on the modeled change from Baseline at Week 78 (or Week 65 if fewer than 60% of subjects are retained in either treatment arm at Week 78). Marginal means (least-square means in SAS) for change from Baseline at Week 78 will be presented for each treatment group. The difference between these marginal means (least-square means in SAS), with 95% confidence interval and p-value, will be provided for the LMTM treatment group *versus* placebo. The change from Baseline to Week 52 in BBSI will also be analyzed using the same type of model. The association between the change in these imaging parameters at Weeks 52 and 78 and change in ADAS-cog_{11} at corresponding weeks will be explored using Pearson's chi-squared test. In addition, the association between the change in these imaging parameters at Week 52 and change in ADAS-cog_{11} at Week 78 will be explored using

**Name of Sponsor / Company:**<br>**TauRx Therapeutics Ltd (TauRx)**
**Name of Finished Product:**<br>**Leuco-methylthioninium bis(hydromethanesulfonate) (LMTM, TRx0237) Tablets, 75 mg, 100 mg**
**Name of Active Ingredient:**<br>**Methylthioninium (MT)**
Pearson's chi-squared test to determine whether the former is predictive of the latter. The thresholds that separate the groups into decliners and non-decliners based on the change in ADAS-cog_{11} and the change in WBV are based on the corresponding mixed effects models as detailed in the statistical analysis section of this protocol.

TABLE OF CONTENTS

PAGE

1. PROTOCOL APPROVAL, RESPONSIBLE PERSONNEL, AND INVESTIGATOR SIGNATURES......2
1.1. PROTOCOL APPROVAL......2
1.2. RESPONSIBLE PERSONNEL......3
1.3. INVESTIGATOR SIGNATURE SHEET......5
SYNOPSIS......6
ABBREVIATIONS......22
2. BACKGROUND......26
2.1. INVESTIGATIONAL PRODUCT......26
2.2. NONCLINICAL DATA......27
2.3. CLINICAL DATA......28
2.3.1. Pharmacokinetics......28
2.3.2. Efficacy......29
2.3.3. Safety......30
2.4. RATIONALE FOR STUDY......32
3. OBJECTIVES......34
3.1. PRIMARY......34
3.2. SECONDARY......34
3.3. EXPLORATORY......35
4. STUDY DESIGN......35
4.1. GENERAL DESCRIPTION......35
4.2. POPULATION......37
4.3. DURATION......37
4.4. SCHEDULE OF ASSESSMENTS......37
4.5. RANDOMIZATION AND BLINDING......42
4.6. STUDY TREATMENT......44
4.7. CONCOMITANT MEDICATION......44
4.7.1. Dementia Medication......44
4.7.2. Drugs with Serotonergic Potential......45
4.7.3. CYP and P-gp Substrates......47
4.7.4. Drugs Used to Manage Behavioral Disturbance......47
4.7.5. Other Medications......48
4.7.6. Dietary Tyramine......48
4.7.7. Contraceptive Measures......48
4.7.8. Folate and Vitamin B_{12}......49

TABLE OF CONTENTS – continued

PAGE

4.8. DATA AND SAFETY MONITORING BOARD / INDEPENDENT DATA MONITORING COMMITTEE 50

5. SELECTION OF SUBJECTS AND CRITERIA FOR WITHDRAWAL 50

5.1. INCLUSION CRITERIA 50

5.2. EXCLUSION CRITERIA 52

5.3. DISCONTINUATION/WITHDRAWAL 55

5.4. TERMINATION OF THE STUDY 56

6. TREATMENTS ADMINISTERED 56

6.1. TREATMENT 57

6.1.1. Active Ingredient 57

6.1.2. Inactive Ingredients 57

6.2. STUDY REGIMENS 57

6.3. DOSE INTERRUPTION / REDUCTION 58

6.3.1. Interruption 58

6.3.2. Dose Reduction 59

6.3.3. Guidance for Selected Adverse Events / Test Abnormalities 60

6.4. PACKAGING, LABELING, AND STORAGE 64

6.5. DISPENSING 64

6.6. COMPLIANCE 65

6.7. ACCOUNTABILITY 65

7. ASSESSMENT OF EFFICACY 66

7.1. RATERS 66

7.2. INSTRUMENTS 67

7.2.1. Alzheimer’s Disease Assessment Scale – Cognitive Subscale (ADAS-cog_{11}) 67

7.2.2. Alzheimer's Disease Cooperative Study – Activities of Daily Living (ADCS-ADL_{23}) 68

7.2.3. Alzheimer's Disease Cooperative Study – Clinical Global Impression of Change (ADCS-CGIC) 68

7.2.4. Neuropsychiatric Inventory (NPI) 69

7.2.5. Montgomery-Asberg Depression Rating Scale (MADRS) 69

7.2.6. Clinical Dementia Rating (CDR) 70

7.2.7. Mini-Mental State Examination (MMSE) 70

8. ASSESSMENT OF SAFETY 71

8.1. ADVERSE EVENTS 71

8.1.1. Intensity 72

8.1.2. Relationship to Study Drug 72

8.1.3. Adverse Events of Special Interest 72

TABLE OF CONTENTS – continued

PAGE

8.1.4. Serious Adverse Events 73
8.1.5. Pregnancy 74
8.1.6. Reporting Requirements and Timeframes 74
8.2. URGENT SAFETY MEASURES 76
8.3. CLINICAL LABORATORY TESTS 77
8.3.1. Serum Chemistry 78
8.3.2. Hematology 78
8.3.3. Urinalysis 79
8.3.4. Other Laboratory Tests 79
8.3.5. Review of Laboratory Results 80
8.4. PULSE CO-OXIMETRY 80
8.5. VITAL SIGN MEASUREMENTS 81
8.5.1. Temperature and Respiratory Rate 81
8.5.2. Blood Pressure and Pulse 81
8.5.3. Weight and Height 82
8.6. ELECTROCARDIOGRAPHY 82
8.7. PHYSICAL AND NEUROLOGICAL EXAMINATIONS 83
8.8. SEROTONIN TOXICITY (SYNDROME) 83
8.9. COLUMBIA-SUICIDE SEVERITY RATING SCALE (C-SSRS) 84
9. OTHER ASSESSMENTS 84
9.1. MAGNETIC RESONANCE IMAGING (MRI) AND ^{18}F-FLUORODEOXYGLUCOSE POSITRON EMISSION TOMOGRAPHY (FDG-PET) 84
9.1.1. General Considerations 84
9.1.2. Site Selection and Qualification 85
9.1.3. Imaging Methods 85
9.1.4. Image Transfer and Quality Assurance 88
9.1.5. Image Evaluation Procedure 89
9.1.6. Site Review 90
9.2. RESOURCE UTILIZATION IN DEMENTIA QUESTIONNAIRE (RUD LITE) 90
9.3. CEREBROSPINAL FLUID BIOMARKERS 91
9.4. MT CONCENTRATIONS 91
9.4.1. Procedure for Blood Sample Collection 92
9.4.2. Packaging, Labeling, and Shipping of Blood Samples 92
9.4.3. Analytical Laboratory 92
9.5. GENOTYPING 92

TABLE OF CONTENTS – continued

PAGE

10. STATISTICAL PLAN **94**
10.1. EFFICACY ENDPOINTS 94
10.1.1. Primary Efficacy Endpoints 94
10.1.2. Secondary Efficacy Endpoints 94
10.1.3. Exploratory and Other Endpoints 94
10.2. SAMPLE SIZE JUSTIFICATION 95
10.2.1. ADAS-cog_{11} 95
10.2.2. ADCS-ADL_{23} 95
10.2.3. ADCS-CGIC 96
10.2.4. MRI 96
10.3. STATISTICAL ANALYSIS 96
10.3.1. Analysis Populations 96
10.3.2. Efficacy Analysis 97
10.3.3. Baseline Characteristics and Concomitant Medications 106
10.3.4. Dosing and Exposure 106
10.3.5. Safety Analysis 107
10.3.6. Concentration of MT 110
10.3.7. Pharmacoeconomics 110
10.3.8. Cerebrospinal Fluid Biomarkers 112
10.3.9. Genotyping 112
10.4. INTERIM ANALYSES 112
11. REGULATORY **113**
12. APPROVAL OF THE PROTOCOL AND AMENDMENTS **114**
13. SERIOUS BREACHES **114**
14. INFORMED CONSENT **114**
15. INVESTIGATOR RESPONSIBILITIES **115**
16. CONFIDENTIALITY AND DATA PROTECTION **116**
17. QUALITY ASSURANCE AND CLINICAL MONITORING **117**
18. DOCUMENTATION **117**
19. PUBLICATION **119**
20. INDEMNITY, INSURANCE AND COMPENSATION **119**
21. ADMINISTRATIVE AND FINANCIAL AGREEMENT **119**
22. STUDY ADMINISTRATION **119**
23. REFERENCES **121**
24. APPENDICES **126**

TABLE OF CONTENTS – continued

PAGE

24.1. ASSESSMENTS BY VISIT 127
24.2. SEROTONIN TOXICITY (SYNDROME) DIAGNOSTIC INTERVIEW AND RATING GUIDE (IN-CLINIC) 142
24.3. SEROTONIN TOXICITY TELEPHONE ASSESSMENT 150
24.4. SUMMARY OF CHANGES TO THE PROTOCOL 151
24.4.1. Protocol Version 1.1 151
24.4.2. Protocol Version 2.0 152
24.4.3. Protocol Version 2.1 156
24.4.4. Protocol Version 2.2 159
24.4.5. Protocol Version 3.0 162
24.4.6. Protocol Version 4.0 168
24.4.7. Protocol Version 4.1 170
24.4.8. Protocol Version 5.0 171
24.4.9. Protocol Version 6.0 173
24.4.10. Protocol Version 7.0 176

ABBREVIATIONS

Abbreviation	Definition
AA	Alzheimer's Association
AChEI	acetylcholinesterase inhibitor
AD	Alzheimer's Disease
ADAS-cog$_{11}$	Alzheimer's Disease Assessment Scale – Cognitive Subscale (11-item)
ADCS-ADL$_{23}$	Alzheimer's Disease Cooperative Study – Activities of Daily Living (23-item)
ADCS-CGIC	Alzheimer's Disease Cooperative Study – Clinical Global Impression of Change
ADNI	Alzheimer's Disease Neuroimaging Initiative
AE	adverse event
AESI	adverse event of special interest
ALT	alanine transaminase
AMG	Arzneimittelgesetz (German Drug Law)
ANCOVA	analysis of covariance
ApoE	Apolipoprotein E
ARIA	amyloid related imaging abnormalities
AST	aspartate transaminase
ATC	Anatomical Therapeutic Classification
AUC	area under the plasma concentration *versus* time curve
β-hCG	Beta subunit of human chorionic gonadotropin
BADL	Basic-ADL
BBSI	Brain Boundary Shift Integral
b.i.d.	twice daily
bpm	beats per minute
°C	Degrees Celsius
C-CASA	Columbia Classification Algorithm for Suicide Assessment
CCA	cost consequence analysis
CDR	Clinical Dementia Rating
CFR	Code of Federal Regulations (United States)
CJD	Creutzfeldt-Jakob Disease
CK	creatine kinase
C_{max}	peak plasma concentration
CNS	Central Nervous System
CPMP	Committee for Proprietary Medicinal Products
CRF	case report form
CSF	cerebrospinal fluid
C-SSRS	Columbia-Suicide Severity Rating Scale
CT	computerized tomography
CUA	cost utility analysis
CV	curriculum vitae
CYP	Cytochrome P450
DICOM	Digital Imaging and Communications in Medicine
DES	Discrete Event Simulations
DNA	deoxyribonucleic acid
DSM-IV-TR	Diagnostic and Statistical Manual of Mental Disorders, Fourth Edition – Text Revision

ABBREVIATIONS – continued

Abbreviation	Definition
DSMB	Data and Safety Monitoring Board
ECG	Electrocardiogram
eCRF	electronic case report form
eGFR	estimated glomerular filtration rate
EMA	European Medicines Agency
EU	European Union
FDA	Food and Drug Administration (United States)
FDG-PET	^{18}F-fluorodeoxyglucose positron emission tomography
g, kg, mg	gram, kilogram, milligram
G6PD	Glucose-6-phosphate dehydrogenase
GCP	Guidelines for Good Clinical Practice
GEE	Generalized Estimating Equation
GGT	gamma-glutamyl transpeptidase
GRE	gradient-recalled echo
HBSI	Hippocampal Boundary Shift Integral
HIPAA	Health Insurance Portability Accountabilty Act
HMPAO-SPECT	hexamethylpropyleneamine oxime - single-photon emission computed tomography
HR	heart rate
HRRT	High Resolution Research Tomograph
HTLV-III	Human T-Cell Lymphocytic Virus Type III
IADL	Instrumental-ADL
ICAD	International Conference on Alzheimer's Disease
ICER	incremental cost effectiveness ratio
ICF	informed consent form
ICH	International Conference on Harmonisation
IEC	Independent Ethics Committee
IND	Investigational New Drug Application
IRB	Institutional Review Board
IRC	Independent Review Charter
IRIS	International Remote Imaging Systems
ITT	intent-to-treat
IUD	intrauterine device
IWRS	interactive web response system
L, mL, dL	liter, milliliter, deciliter
LAV	Lymphadenopathy Associated Virus
LDH	lactate dehydrogenase
LMT	leuco-methylthioninium
LMTB	leuco-methylthioninium bis(hydrobromide)
LMTM	leuco-methylthioninium bis(hydromethanesulfonate)
MADRS	Montgomery-Asberg Depression Rating Scale
MAO A	monoamine oxidase A
MAO B	monoamine oxidase B
MCH	mean corpuscular hemoglobin

ABBREVIATIONS – continued

Abbreviation	Definition
MCHC	mean corpuscular hemoglobin concentration
mCi	millicurie
MCV	mean cell volume
MDRD	Modification of Diet in Renal Disease
MedDRA	Medical Dictionary for Regulatory Activities
MetHb	methemoglobin
MHRA	Medicines and Healthcare products Regulatory Agency (United Kingdom)
min	minute(s)
MITT	modified intent-to-treat
mIU	milli-international units
mmHg	millimeters of mercury
MMSE	Mini-Mental State Examination
MP RAGE	magnetization-prepared rapid acquisition with gradient echo
MR	magnetic resonance
MRI	magnetic resonance imaging
msec	millisecond(s)
MT	Methylthioninium
MTC	methylthioninium chloride
NIA	National Institute on Aging
NMDA	N-methyl-D-aspartate
NPI	Neuropsychiatric Inventory
nvCJD	new variant Creutzfeldt-Jakob Disease
PE	physical examination
PET	positron emission tomography
P-gp	P-glycoprotein
PhEur	European Pharmacopoeia
PK	Pharmacokinetic
PP	Per Protocol
QA	quality assurance
QALY	quality adjusted life years
QTcF	QT interval corrected using Fridericia’s formulae
RBC	red blood cell
rCBF	regional cerebral blood flow
ROI	Region of Interest
rpm	revolutions per minute
RUD	Resource Utilization in Dementia Questionnaire
SAE	serious adverse event
SAP	statistical analysis plan
SD	standard deviation
SNRI	serotonin-norepinephrine reuptake inhibitor
SOC	system organ class
SOP	standard operating procedure
SPGR	spoiled gradient echo

ABBREVIATIONS – continued

Abbreviation	Definition
SPM	statistical parametric mapping
SSRI	selective serotonin reuptake inhibitor
SSS	symptom severity rating score
SUSAR	suspected, unexpected serious adverse reaction
$T_{1/2}$	terminal elimination half-life
T_3	Triiodothyronine
T_4	Thyroxine
TBG	thyroxine binding globulin
TCA	tricyclic antidepressant
TEAE	treatment-emergent adverse event
t.i.d.	three times daily
T_{max}	time of the peak plasma concentration
TSE	Transmissible Spongiform Encephalopathy
TSH	thyroid stimulating hormone
UK	United Kingdom
US	United States
USP	United States Pharmacopoeia
VBSI	Ventricular Boundary Shift Integral
vCJD	variant Creutzfeldt-Jakob Disease
WBC	white blood cell
WBV	whole brain volume
WCT	Worldwide Clinical Trials
WHO	World Health Organization

2. BACKGROUND

Methylthioninium (MT) is proposed for treatment of Alzheimer's disease (AD) and other dementias that involve pathology of the microtubule associated protein tau, and with potential for benefit in other progressive neurodegenerative diseases characterized by pathological protein aggregation. AD is a severe irreversible neurodegenerative disease resulting in complete loss of mental faculties. In Alzheimer's disease, the microtubule associated protein tau is redistributed exponentially into paired helical filaments forming neurofibrillary tangles which correlate with pyramidal cell destruction (Wischik *et al*., 1997). There is a robust clinico-pathological correlation between tau pathology, tau aggregation, and clinical measures of dementia (Bierer *et al*., 1995; Mukaetova-Ladinska *et al*., 2000). These relationships are maintained from the earliest detectable stages of dementia and progress in parallel with clinical deterioration.

Drugs currently available to treat AD, such as acetylcholinesterase inhibitors (AChEIs) and memantine, are symptomatic treatments which address certain central neuronal dysfunctions associated with AD, but are not known to directly affect the neurofibrillary tangles in the brain that represent a core pathological component of AD. Controlled studies with the AChEIs donepezil, rivastigmine, and galantamine have demonstrated small improvements in cognitive tests and global measures of change in selected subjects with mild to moderate Alzheimer's disease over 3-12 months (Birks and Harvey, 2004; Birks *et al*., 2004; Olin and Schneider, 2004). However, improvements in function and behavior have been demonstrated less reliably with AChEIs. Furthermore, although these medications provide benefits for some subjects, their effectiveness often is limited in duration and they do not affect the rate of progression of the disease (AD2000 Collaborative Group, 2004).

Memantine is a noncompetitive, low-affinity, N-methyl-D-aspartate (NMDA) receptor antagonist that might prevent calcium-mediated glutamate excitatory toxicity in AD. Studies with memantine over 6 months in subjects with moderate-to-severe AD have shown small benefits on cognition, global measures, daily living activities, and behavior (Reisberg *et al*., 2003), but benefits in mild-to-moderate AD have been less notable.

Therefore, an unmet need exists to develop new medications for AD that more directly modify the underlying disease pathology and offer longer term greater efficacy. Leuco-methylthioninium bis(hydromethanesulfonate) (LMTM), the investigational product, is believed to have the potential to confer benefits over existing treatments for AD due to its ability to affect the process of tau aggregation responsible for the underlying neurofibrillary pathology of Alzheimer's disease. Available nonclinical and clinical evidence supports the clinical evaluation of LMTM in Alzheimer's disease.

2.1. Investigational Product

The investigational product LMTM (also referred to as leuco-methylthioninium dihydromesylate and LMT.2MSOH, as well as by its code name, TRx0237) is a crystalline form of the reduced form of the active MT moiety. Chemically, the drug is named *N,N,N',N'*-tetramethyl-10*H*-phenothiazine-3,7-diaminium bis(methanesulfonate). It is provided as a solid oral immediate-release dosage form. As a dihydromethanesulfonate salt (also known as mesylate), LMTM stabilizes the reduced form of the MT moiety in the solid state; further, a matching placebo is provided that includes 4 mg LMTM as a urinary and fecal colorant.

The chloride salt of MT, methylthioninium chloride (MTC), is a diaminophenothiazine dye commonly known as methylene blue. It is the main ingredient in a number of proprietary preparations worldwide used at oral doses of up to 65 mg 3 times daily (*t.i.d.*) (50 mg MT *t.i.d.*) or as a component in various multi-ingredient formulations at doses up to 10 mg *t.i.d.* (8 mg MT *t.i.d.*). The most common use is as a urinary antiseptic in chronic urinary tract infection; other uses include treatment of subjects with manic-depressive psychosis, uncomplicated *Plasmodium falciparum* malaria, and urolithiasis. Methylthioninium chloride is approved in the European Union for parenteral administration in the treatment of drug-induced methemoglobinemia; MTC is usually given intravenously at 0.8 to 1.5 mg/kg body weight, administered over 5 minutes. It is also used parenterally in the management of ifosfamide encephalopathy and refractory shock syndromes of various causes, as a tracer in sentinel lymph node biopsy or visualization in parathyroid surgery, as well as a photosensitizing agent for the sterilization of blood products. While documented extensively in the published literature and various pharmacopeia (USP, PhEur), to TauRx's knowledge, all uses other than the intravenous treatment of methemoglobinemia remain experimental and have not been subject to regulatory review.

LMTM, LMTB (leuco-methylthioninium bis[hydrobromide], an earlier reduced salt form under investigation), and MTC all deliver the same active moiety, *i.e.*, MT, following dissociation of the counter ions. The absorption of the reduced leuco-MT (LMT) forms is favored at low pH such as in the stomach. When the MT moiety is dosed as MTC, it is presented in its charged or oxidized form (*i.e.*, MT^+). It is postulated that there is a requirement for an active reduction step at the low pH in the stomach to permit absorption of MT as the uncharged LMT form (which has one additional covalently bound hydrogen). Within cells, the active moiety MT is postulated to be present in an equilibrium between the reduced or oxidized forms, depending on the intracellular milieu, *i.e.*, pH and reductive capacity within that cell. As salts of MT have high solubility in aqueous media and deliver the same active moiety, the nonclinical and clinical experience with MTC and LMTB are relevant to LMTM. MTC and the reduced salts have been shown to result in comparable exposure when compared at equivalent MT doses in humans in the fasting state.

2.2. Nonclinical Data

In cell models, MTC, LMTM, and LMTB have been shown to prevent aggregation and enhance clearance of aggregation-dependent proteolytic products of tau. In transgenic mouse models, facilitation of clearance of tau aggregates and associated improvements in cognitive and motor learning abilities have been demonstrated. Tau pathology in these transgenic mouse models has been shown to be ameliorated in certain brain regions (*e.g.*, entorhinal cortex, hippocampus, and neocortex) following treatment with MTC, LMTB, and LMTM.

The primary toxicity of MT is hematological, manifesting as methemoglobinemia and a regenerative hemolytic anemia. Depending on the species, Heinz body formation also occurs. A no-observed-adverse effect-level has not been clearly established in any species other than rats (3 to 5 mg MT/kg/day regardless of salt or duration, depending on the low dose in a given study). Hematological toxicity was seen in the other species even at the lowest doses studied (0.9 mg MT/kg/day in monkeys and 3 mg MT/kg/day in minipigs). At higher doses, myeloid generation is affected and extramedullary hematopoeisis is seen; pigment (hemosiderin) deposition becomes evident in liver and renal cortical tubules (some lipofuscin also present in minipigs) with no adverse effect on either liver or kidney function evident.

The other potential significant toxicities observed include effects on the heart, described as myocardial necrosis, generally occurring at rapidly lethal doses that exceed the maximum tolerated doses for chronic administration. In the minipig, no treatment-related cardiac toxicity has been observed until supra-lethal doses are achieved. Gastric irritation is seen in rodents and urinary bladder irritation is seen in mice and minipigs (with single cell necrosis in the latter species).

MT and some of its metabolites are genotoxic *in vitro* and damage DNA but are not genotoxic *in vivo.*

As reviewed by the U.S. National Toxicology Program, there is "some evidence of carcinogenic activity"[1] of MTC in male rats based on increased incidences of pancreatic islet cell adenoma and adenoma or carcinoma (combined). There is "some evidence of carcinogenic activity" in male mice based on increased incidences of carcinoma and of adenoma or carcinoma (combined) in the small intestine. The increased incidence of malignant lymphoma in 19 mg MT/kg/day males may be related to the administration of MTC whereas there is "equivocal evidence of carcinogenic activity" in female mice based on marginally increased incidences of malignant lymphoma.

MT has no effect on mating performance or pregnancy rate in Sprague-Dawley rats when administered orally. MT results in embryofetal toxicity when administered orally as evidenced by increased fetal resorptions in rats and (non-statistically) in rabbits; in rats the effects occur at doses that are maternally toxic. Following a single subcutaneous dose of 27 mg MT/kg and above, axial skeletal malformations are observed; neural tube defects, other fetal effects, and post-implantation losses occur at a threshold dose of 38 mg MT/kg.

In a standard study performed in male Long-Evans pigmented rats, MT does not cause phototoxicity.

Additional details regarding findings from nonclinical studies with various salt forms (LMTM, LMTB, and MTC) are described in the Investigator's Brochure.

2.3. Clinical Data

2.3.1. Pharmacokinetics

To date, MT concentrations have been detected using an assay that includes heat- and acid-labile metabolites (conjugates).

LMTM, MTC, and LMTB have been shown to result in comparable exposure when given orally at equivalent MT doses in humans in the fasting state.

In humans, MT is rapidly absorbed when dosed in the fasted state. Median time of the peak plasma concentration (T_{max}) ranges from 1 to 2 hours post-dose when administered as single doses up to 300 mg; T_{max} occurs later at higher doses, up to an average of 2.8 hours post-dose. Peak plasma concentration (C_{max}) and area under the plasma concentration *versus* time curve (AUC) increase linearly with dose, but in a less than proportional fashion at doses greater than 100 mg. Secondary peaks are evident on review of individual plasma *versus* concentration

---

[1] Text in quotation marks refers to the interpretation of results provided by the U.S. National Toxicology Program, the sponsoring organization.

profiles. Based on published information and excretion of radiolabel in urine, absolute bioavailability is estimated to be 72%. Coadministration of the reduced leuco (LMT) form with food does not alter the extent of absorption significantly (peak concentrations are 8% lower); however, peak plasma concentrations are delayed by about 1.5 hours. Steady state is achieved within 3 days of dosing. The volume of distribution of total MT (*i.e.*, including acid- and heat-labile metabolites) administered orally approximates total body volume (mean 71 L). Apparent volume of distribution increases with increase in dose. MT is not highly protein bound (70 to 75%). Across studies, MT concentrations in whole blood samples are approximately half those in plasma. Study of MT metabolism in humans is ongoing; the primary metabolite appears to be a conjugate. Following a single dose, plasma concentrations decline with a mean elimination half-life ($T_{½}$) of 14 hours in younger healthy volunteers and 16 to 22 hours in healthy elderly volunteers. The $T_{½}$ of elimination of radiolabel from plasma ranges from 12.3 to 31.2 hours; elimination from whole blood is slower, ranging from 20.6 to 58.8 hours. An average of 95% of administered radioactivity is recovered over the 216 hours of urine and feces collection, approximately half within 24 hours of dosing. A total of 72% is recovered from the urine (42% within the first 24 hours) and 23% from the feces (5% within the first 24 hours). Renal clearance is less than glomerular filtration rate (GFR). Renal clearance of total radioactivity is 29 mL/min, approximately 71% of total clearance.

2.3.2. Efficacy

In a double-blind, placebo-controlled Phase 2 study (TRx-014-001) of male and female subjects with mild or moderate AD (acetylcholinesterase inhibitors [AChEIs] and memantine excluded), MTC was administered orally at doses of 30 mg *t.i.d.*, 60 mg *t.i.d.*, and 100 mg *t.i.d.* (total doses of 69 mg/day, 138 mg/day, and 228 mg/day MT base equivalents, respectively). MT 138 mg/day appeared to slow the clinical rate of decline on the Alzheimer's Disease Assessment Scale – cognitive subscale (ADAS-cog_{11}) measured over 1 year, an effect that was already evident in patients with moderate disease at baseline within the first 6 months in an analysis of the whole population with severity as an interaction term. The 6-month analysis and methodology were prespecified as part of the primary analysis with the severity term as an interaction covariate. The result in patients with moderate disease severity at 6 months remained significant after correction for multiple comparisons. Longer term efficacy was confirmed in mixed mild/moderate subjects by *post hoc* analyses at 50 weeks and 102 weeks, and efficacy was also confirmed in analyses of a variety of secondary endpoints (Wischik *et al*., 2015). The lower dose (69 mg/day) was below the minimally effective dose. The capsule formulation used limited the absorption of MT at the 228 mg/day dose due to a combination of dose-dependent delay in dissolution of the 100 mg MTC capsules used in the study and a formulation-independent limitation in the ability to absorb MT at the highest dose in the presence of food when administered as MTC. When both of these factors were taken into account, the clinically effective dose available for release within 60 minutes in subjects receiving a nominal dose of 228 mg/day was equivalent to approximately 69 mg/day (Baddeley *et al*., 2015), and the total available dose was equivalent to 109 mg/day (Wischik *et al*., 2015).

Evidence of clinical efficacy was supported by neuroimaging in 138 mild and moderate AD subjects imaged at baseline and at 24 weeks. Despite lack of decline over this time detected by subjective measures such as ADAS-cog in subjects with mild disease at baseline, statistically significant decline in neuronal function as measured by regional cerebral blood flow (rCBF) was detected using HMPAO-SPECT in all predefined lobes in the region-of-interest (ROI) analysis

and was particularly marked in the inferior medial temporal and temporo-parietal regions of the neocortex on the statistical parametric mapping (SPM) analysis, areas characteristically affected by the tau aggregation pathology of AD and the regions primarily affected in the Braak staging system (Braak and Braak, 1991). MTC at a dose of 138 mg MT/day was found to eliminate this decline entirely (Wischik *et al*., 2015). A similar effect was seen in a smaller group of 21 subjects imaged by FDG-PET to measure neuronal function by glucose uptake. The effect on functional neuroimaging markers at 24 weeks in subjects with mild AD was found to be predictive of a clinical response at 50 weeks (Wischik *et al*., 2015). The reduced efficacy of the 100-mg capsule dose (228 mg MT/day) relative to the 60-mg capsule dose (138 mg MT/day) was also confirmed by neuroimaging.

2.3.3. Safety

A total of 284 healthy volunteers have been studied in completed single and multiple dose studies of MT as of October 2014. These include single and multiple doses (expressed as MT base) of MTC 46 to 76 mg and 207 mg/day for 14 days, respectively; LMTB up to 800 mg as a single dose and up to 350 mg/day for 14 days; and LMTM up to 1000 mg as a single dose and up to 450 mg/day for 25.5 days. The most common adverse events (AEs) were gastrointestinal, renal and urinary disorders, and headache.

A total of 321 subjects with AD participated in the 2-year Phase 2 study, TRx-014-001, of whom 307 were exposed to at least one dose of MTC. Diarrhea and related adverse events occurred in 48% at the highest dose, 228 mg MT/day, resulting in discontinuation of 16% overall. Nausea, vomiting, and retching were also common in MTC-treated subjects (8 to 14%), without a dose-response relationship; 3% of subjects discontinued due to vomiting. Dysuria and urgency were more common in MTC-treated subjects as compared to placebo, without a dose-response relationship (22 to 29% of subjects on MTC as compared to 13% of subjects on placebo). Urinary adverse events, including urinary discoloration and staining, resulted in the discontinuation of 6% of the subjects. Other common adverse events seen in ≥ 5% of MTC-treated subjects overall and more common in MTC-treated subjects compared to placebo, were falls and related AEs (not correlated with change in blood pressure but correlated statistically with diarrhea) and rashes (none exfoliative).

Less common, but significant, adverse events that have been associated with MTC given parenterally include anaphylaxis and serotonin syndrome (the latter when MTC has been used intravenously in combination with products that increase serotonin). No cases have been reported to date for oral administration, including in the Phase 2 study where 17% of subjects received oral MTC in combination with products that increase serotonin.

As in animal studies, the primary laboratory abnormality is hematologic. Small, dose-dependent increases in methemoglobin are seen (mean overall increase was from 0.4% in controls to 0.8% in those treated with MTC). The highest value observed in the Phase 2 study has been 8%, not clinically significant; mean methemoglobin levels have remained below 3.5%, a conservative pre-specified threshold. Small reductions in hemoglobin and hematocrit values and compensatory increases in reticulocyte counts are seen, generally not clinically significant. Possible liver function disturbance was evidenced by a single instance of jaundice and a single case of increase in gamma-glutamyl transpeptidase (GGT) and alkaline phosphatase, both of which were reported as possibly related.

In a completed Phase 1 study with LMTM (TRx-237-011), several subjects had protein and blood in the urine shown by dipstick analysis, not correlated with the reports of dysuria. Colorimetric interference by MT on the dipstick tests used confounds interpretation of these results; nonetheless, in some male subjects the presence of blood in the urine was confirmed by microscopy. (Urinalysis was not included in the Phase 2 trial.)

MT has not been associated with changes in vital signs or electrocardiogram (ECG) intervals.

MTC has been shown to be an inhibitor of monoamine oxidase (MAO), with greater potency *in vitro* (*i.e.*, in the oxidized MT^+ form) for the A isoform than the B isoform. The Medicines and Healthcare products Regulatory Agency (MHRA) in the UK, Health Canada, and, most recently, the US Food and Drug Administration (FDA) have issued safety alerts concluding that the concomitant administration of a serotonergic psychiatric medication with intravenous administration of MTC has the potential for a drug interaction causing serotonin syndrome. These are based on spontaneously reported serious adverse events as well as published cases, when MTC was used in intravenous doses of 1 to 8 mg/kg mostly for gland visualization in parathyroidectomy. As a result, MTC should generally not be given intravenously to subjects taking serotonergic drugs unless the benefit is deemed to outweigh the risk (FDA advice) or subjects are observed for at least 4 hours after the MTC dose (MHRA advice). TauRx is aware of only one[2] reported case of serotonin syndrome with MTC administered non-parenterally.

Of the 1713 subjects with AD or bvFTD randomized to receive LMTM (or matching placebo) in the ongoing double-blind Phase 3 studies as of 30 September 2014[3], 383 subjects (22.3%) were concomitantly receiving a selective serotonin reuptake inhibitor (SSRI) and 540 subjects (31.5%) were concomitantly receiving drugs with serotonergic potential. With extensive monitoring implemented in the ongoing double-blind Phase 3 studies using the Serotonin Toxicity (Syndrome) Diagnostic Interview and Rating Guide, there have been 2 cases of possible serotonin toxicity reported as AEs (as of 14 October 2014) based on the Serotonin Toxicity Diagnostic Interview score. Both were mild in intensity and resolved with treatment interruption; the symptoms did not reappear with restarting study drug. Neither subject reported concomitant use of a serotonergic medication or substance. For one of these cases, after 23 days of follow-up, the Investigator concluded that the case did not represent true clinical serotonin toxicity. Although 17% of subjects exposed to MTC in the 2-year Phase 2 study, TRx-014-001, were taking SSRIs or venlafaxine (a serotonin-norepinephrine reuptake inhibitor, SNRI) concomitantly at some point during the study, there was no report of an episode of serotonin syndrome. This may reflect the difference in predominant form of MT (*i.e.*, LMT rather than MT^+) in circulation following oral *versus* intravenous administration. The risk with oral LMTM is unknown. Nonetheless, there is a theoretical risk of increased serotonin levels following administration of LMTM or following coadministration of LMTM with a serotonergic medication. Close monitoring will continue in the ongoing double-blind Phase 3 studies.

In addition to warning subjects and their caregivers of this risk, they are also to be informed that based on this pharmacological profile there is a theoretical potential for a tyramine reaction; TauRx is not aware of any reports of diet-associated hypertensive crisis.

---

[2] Izdes et al., 2014 (administration as a solution via nasogastric tube).
[3] Data available at the time of the Developmental Safety Update Report #3 cut-off date of 14 October 2014.

Additional details regarding clinical findings from studies with LMTM, LMTB, and MTC are described in the Investigator's Brochure.

2.4. Rationale for Study

As noted above, a clinical Phase 2 study of the closely related drug MTC (monotherapy) provided evidence supportive of pursuing additional studies of efficacy in mild to moderate Alzheimer's disease. The overall study design and 18-month treatment period for the current study are considered appropriate for Phase 3 evaluation of efficacy and safety of a putative disease-modifying treatment for Alzheimer's disease in patients with mild severity. The primary and secondary efficacy parameters selected for this study, described in Section 7.2, have been validated and widely used.

Change in whole brain volume (WBV) as measured by the Brain Boundary Shift Integral (BBSI; Freeborough and Fox, 1997) will be used as the principal imaging outcome to support a disease-modifying effect of treatment with LMTM by showing a reduction in the overall rate of brain atrophy at 78 weeks. In addition, exploratory analyses will be conducted to further support an effect on reduction of brain atrophy by analyses of change in hippocampal volume using the Hippocampal Boundary Shift Integral (HBSI; Barnes *et al.*, 2007) and change in ventricular volume using the Ventricular Boundary Shift Integral (VBSI). The change at 39 weeks will also be compared with that observed at the same time in Study TRx-237-015.

Functional neuroimaging using HMPAO-SPECT and FDG-PET during life is correlated with Braak stage post-mortem (Jobst *et al*., 1992; Bradley *et al*., 2002) which reflects the progression of tau pathology in the brain. Functional neuroimaging markers are considered to be more sensitive to disease progression in mild AD than structural volumetric imaging by MRI (Matsuda *et al*., 2002). Evidence suggestive of disease-modifying efficacy was shown in the Phase 2 study. Given the more widespread availability and similar sensitivity of FDG-PET (when performed using computerized tomography [CT] or a brain-dedicated high-resolution device) than HMPAO-SPECT, the former will be used in an exploratory fashion to further support putative disease-modifying efficacy in a mild population. The change at 39 weeks will also be compared with that observed at the same time in Study TRx-237-015.

In subjects who withdraw prematurely, the post-Baseline scans by either MRI or FDG-PET will be repeated within 14 days (and at most 28 days) of discontinuation, as longer periods may be associated with resumption of the decline trajectory as discussed next.

MT acts to catalyze clearance of tau aggregates by converting them to monomers, against a background of endogenous conditions which favor either tau aggregation or limit clearance of aggregates. Withdrawal of treatment would be expected to permit resumption of the aggregation cascade and erosion of benefit. Whether a clinical change is evident in a 28-day time frame is not clear but will be explored by the inclusion of a 4-week post-treatment follow-up visit in all subjects.

The dose level (200 mg/day) and dosage regimens (twice daily) of orally administered LMTM were selected based on available nonclinical and clinical data for LMTM, LMTB, and MTC. In the Phase 2 study of MTC (see Section 2.3.2), potential efficacy was consistently associated with the oral dose of 60 mg *t.i.d.* (46 mg *t.i.d.*, or 138 mg/day, expressed as mg MT), and a dose of 100 mg *t.i.d.* (76 mg *t.i.d.*, or 228 mg/day, expressed as mg MT) was tolerated. Based on clinical pharmacokinetic data, the dose of 200 mg/day administered as two divided doses of LMTM is

expected to produce a steady-state plasma level similar to that achieved at the highest dose of MTC studied to date, 228 mg/day, administered as three divided doses of MTC. The twice daily regimen is consistent with the approximate 16- to 22-hour plasma elimination half-life observed in healthy elderly volunteers following single doses of LMTB.

A small amount of LMTM (4 mg) has been included in the placebo tablets, sufficient to color urine and feces, thereby maintaining the study blind. The low 8 mg/day dose of LMTM to be administered to subjects in the placebo group in this study is believed to be unlikely to cause functional or structural effects in the brain or to result in efficacy in Alzheimer's disease.

Concomitant medications are allowed consistent with the patient population provided there are no safety grounds for exclusion. AChEI and/or memantine use is allowable but not mandatory for subjects enrolled in this study in part because in clinical practice a new treatment for Alzheimer's disease such as LMTM might be anticipated to be administered concomitantly in some countries. To avoid interference with the primary study objectives, the dose and regimen must have been stable as described in Section 4.7.1.

Subjects may also be treated with antidepressant medication. However, because MT^+ is a MAO A and, to a lesser extent, MAO B inhibitor *in vitro*, drugs with serotonergic potential should be used with caution due to the theoretical risk of serotonin syndrome based on intravenous usage. The risk with oral LMTM is unknown. Subjects will be allowed to enter the study on such medication and will be closely monitored, including in the clinic for at least 4 hours after the first dose. They and their caregiver(s) are to remain in close proximity to a local hospital and remain in telephone contact with the clinic for at least 12 to 14 hours after the first dose and reimbursement for accommodations will be made available if requested by subjects and/or their caregivers. They will be instructed in the signs and symptoms of serotonin toxicity and given an oral thermometer and instructed in its use and diary recording. In the intervening time before the next clinic visit, caregivers will be contacted by telephone 5–7, >7–14, >14–24, 44–52, and 68–76 hours post-dose (with a minimum of 1 hour between contacts) and queried for any signs or symptoms of serotonin toxicity in the subject.

Anticipated risks of participation in this study include the potential of experiencing AEs similar to those observed in a previously-conducted Phase 2 study with MTC in subjects with mild or moderate Alzheimer's disease (see Section 2.3.3). Standard safety assessments are included at frequent intervals. Subjects will be monitored for any signs or symptoms of possible methemoglobin increases and monitoring guidelines for dosing are provided; pulse co-oximetry will be used to avoid the potential interference by other colorimetric approaches. ECGs were not obtained in the Phase 2 study with MTC and a study of the potential for MT to alter the QT interval has recently completed; however, data analysis is still ongoing. Therefore, ECGs will continue to be monitored in the study (including approximately 3 hours after the initial dose which is to be administered in the clinic). Routine clinical laboratory testing will be performed throughout the study. Additional monitoring after the first dose includes measurement of blood pressure and pulse with assessment of orthostatic changes. An independent Data and Safety Monitoring Board (DSMB) will be established (see Section 4.8).

Because MT has activity on the central nervous system (CNS), subjects will also be evaluated for suicidal ideation and intent using the C-SSRS. This is consistent with regulatory expectations for CNS-active medications. While not validated for use in this subject population, it has been used

and provides a systematic means of collecting data in dementing syndromes for future potential regulatory use.

MT has been shown *in vitro* to disrupt oligomerization and aggregation of recombinant amyloid protein in solution at concentrations 15- to 89-fold higher than required to cause disaggregation of paired helical filaments isolated from AD brain tissue. Although the *in vivo* relevance of the effect on amyloid aggregates is not known, if MT is to be used for more than 3 months in the treatment of subjects with AD, magnetic resonance imaging (MRI) is recommended to monitor for amyloid related imaging abnormalities (ARIA). The limited data currently available appear to indicate that the imaging abnormalities thought to represent vasogenic edema occur in those who have either overt AD or an earlier stage of cognitive impairment that is considered likely to progress to overt AD over time. In subjects with AD, FDA had initially advised that the imaging be undertaken “at intervals of not less than every 3 months”. Clinical efficacy assessments in this 78-week study are scheduled every 13 weeks. To avoid additional visits, MRI brain scans are to be obtained at this same schedule (which approximates the every 3-month schedule). Although such monitoring is no longer required by the FDA, TauRx will continue to obtain MRI brain scans to collect whole brain volume data as a secondary efficacy endpoint, as well as ventricular and hippocampal volume data as an exploratory endpoint. These MRI brain scans (obtained every 13 weeks) will continue to be evaluated for ARIA.

3. OBJECTIVES

The primary, secondary, and exploratory objectives of this double-blind, placebo-controlled, randomized, parallel-group, 18-month study of LMTM in subjects with mild AD are stated below.

3.1. Primary

The primary efficacy and safety objectives are:

1. To demonstrate clinical efficacy of leuco-methylthioninium bis(hydromethanesulfonate) (also known as LMTM, TRx0237) in mild Alzheimer’s disease based on change from baseline on the following co-primary endpoints:
    - Alzheimer’s Disease Assessment Scale – Cognitive Subscale (ADAS-cog_{11}) and
    - Alzheimer’s Disease Cooperative Study – Activities of Daily Living (ADCS-ADL_{23})
2. To assess the safety and tolerability of LMTM 200 mg/day given for up to 78 weeks

3.2. Secondary

The secondary objectives are:

3. To further demonstrate disease modification based on the following key secondary endpoint:
    - Reduction in decline in whole brain volume (WBV) using change from Baseline as measured by the Brain Boundary Shift Integral (BBSI) by MRI imaging
4. To evaluate the effect of LMTM on a global measure, Alzheimer’s Disease Cooperative Study – Clinical Global Impression of Change (ADCS-CGIC) – independently rated

5. To evaluate the effects of LMTM on other aspects of Alzheimer's disease including cognition (Mini-Mental Status Examination, MMSE), behavior (Neuropsychiatric Inventory, NPI), and mood (Montgomery-Asberg Depression Rating Scale, MADRS)

3.3. Exploratory

The exploratory objectives are:

6. To determine the effects of LMTM on Alzheimer's disease modification by showing retardation of the rate of brain atrophy by reducing the expected increase in ventricular volume and decline in hippocampal volume as evaluated by MRI
7. To determine the effects of LMTM on reduction in decline in glucose uptake in the temporal lobe on ^{18}F-fluorodeoxyglucose positron emission tomography (FDG-PET) imaging
8. To determine the effects of LMTM on resource utilization using the Resource Utilization in Dementia (RUD) Lite
9. To explore changes in certain cerebrospinal fluid (CSF) biomarkers of Alzheimer's disease (total tau, phospho-tau, and $A\beta_{1-42}$) in subjects who are mentally capable of providing their own separate informed consent and specifically agree to have lumbar puncture performed
10. To explore the influence of the Apolipoprotein E (ApoE) genotype on the primary and selected secondary outcomes (in subjects by or for whom legally acceptable consent is provided)

Blood will be collected for purposes of population pharmacokinetic analysis. This will be detailed in a separate Statistical Analysis Plan (SAP) and will be reported separately (together with data from other studies).

4. STUDY DESIGN

4.1. General Description

This study is a multinational, randomized, placebo-controlled, double-blind, parallel-group study of LMTM 200 mg/day (expressed as MT) in subjects with mild Alzheimer's disease. Subjects will be allowed but not required to be receiving concomitant treatment in a stable regimen (*i.e.*, with an AChEI and/or memantine) for Alzheimer's disease.

Eligibility for enrollment will be assessed initially at one or more Screening visits, which are to occur within 42 days of Baseline. Eligible subjects will be randomly assigned (1:1) at the Baseline visit to one of the following two treatment groups: the LMTM 200 mg/day group or the placebo group. The randomization will be stratified by geographic region, screening severity (CDR 0.5 or 1), and use of an AChEI and/or memantine (see Section 4.5 for stratification factors).

Subjects will receive study drug twice daily (*b.i.d.*) for 78 weeks (18 months) (LMTM 100 mg *b.i.d.* or placebo *b.i.d.*). The placebo regimen includes a low dose of LMTM 4 mg *b.i.d.* to maintain blinding (see Sections 4.5 and 6.1.1); hence, the placebo regimen actually contains LMTM 8 mg/day. During the treatment period, interruption of dosing for up to (maximum

duration on any one occasion) 30 days during the first 6 months of treatment and/or 14 days during the second 12 months of treatment or 50-mg reduction of daily dose (up to a maximum of two such reductions; see Section 6.3) will be allowed if indicated (*e.g.*, tolerability concerns or clinical laboratory abnormalities). However, subjects may resume treatment at the full dose if tolerability concerns cease. In the event of continued poor tolerance, subjects should be withdrawn from treatment (but encouraged to return for the balance of the scheduled study visits).

Study visits during the treatment period will occur at time points approximately 2, 6, 13, 26, 39, 52, 65, and 78 weeks after Baseline. Caregivers will be contacted by telephone at approximately 9, 19, 32, 45, 58, and 71 weeks after Baseline. All subjects are to return for a follow-up visit approximately 4 weeks after the last dose of double-blind study drug.

Efficacy and safety assessments as well as imaging for disease modification will be performed at Baseline and at designated visits throughout the treatment period. Separate efficacy and safety raters will be used to maintain blinding; further, the ADCS-CGIC will be rated by a completely independent rater (see further detail in Section 7, Section 8, and Section 9 for efficacy, safety, and imaging and other assessments, respectively).

Subjects who complete treatment through to the final off-treatment follow-up visit (Visit 11) may be offered an opportunity to transition directly into an open-label extension study (separate protocol).

A schematic representation of the study design is provided in Figure 4-1.

Figure 4-1 Schematic Representation of Study Design

**Period**	**←Screening→ (up to 42 days)**	**←——— Double-Blind Treatment [a] ———→ LMTM 200 mg/day or Placebo[b] (78 weeks)**															**←Follow up→ (4 weeks after last dose)**
	**Screening Visit** ↓	**Randomization and first dose** ↓														**Last dose** ↓	**Follow up Visit [c]** ↓
**Visit[d]**	↑ **1**	↑ **2**	↑ **3**	↑ **4**	**(T)**	↑ **5**	**(T)**	↑ **6**	**(T)**	↑ **7**	**(T)**	↑ **8**	**(T)**	↑ **9**	**(T)**	↑ **10**	↑ **11**
**Week**		**0**	**2**	**6**	**9**	**13**	**19**	**26**	**32**	**39**	**45**	**52**	**58**	**65**	**71**	**78**	**82**
**Day (± days)**	**≤ -42**	**1**	**15 (3)**	**43 (3)**	**64 (7)**	**92 (7)**	**134 (7)**	**183 (14)**	**225 (7)**	**274 (14)**	**316 (7)**	**365 (14)**	**407 (7)**	**456 (14)**	**498 (7)**	**547 (14)**	**575 (7)**

[a] During the treatment period, study drug will be administered under double-blind conditions. All subjects will be randomized to receive one tablet twice each day (*b.i.d.*; one tablet each morning and one tablet each evening). Two 50-mg/day dose reductions or dose interruption(s) will be allowed as needed for tolerability (see Section 6.3 for more details).

[b] Placebo group subjects will receive a total daily dose of 8 mg LMTM, administered as one LMTM 4-mg tablet *b.i.d.*; this low dose is included to maintain blinding because LMTM is associated with coloration of urine and feces.

[c] Subjects who complete treatment through the final off-treatment follow-up visit may be offered an opportunity to transition directly into an open-label extension study (separate protocol).

[d] After the Week 6 visit (Visit 4), a telephone contact (T) will occur between each scheduled study site visit (↑) during the treatment period at Weeks 9, 19, 32, 45, 58, and 71.

4.2. Population

Subjects enrolled into this study will have a diagnosis of all cause dementia and probable Alzheimer's disease according to the proposed criteria of the National Institute on Aging (NIA)/Alzheimer's Association (AA). The allowable severity of Alzheimer's disease will be mild as indicated by a Clinical Dementia Rating (CDR) total score of 0.5 or 1 and a Mini-Mental State Examination (MMSE) score of 20 to 26 (inclusive) at Screening (Visit 1).

Subjects taking an AChEI and/or memantine may be enrolled provided they have been taking such medication for ≥ 3 months at Screening and the dosage of such medication has remained stable for the previous ≥ 6 weeks prior to Screening. Subjects never treated with an AChEI or memantine (and for whom initiation of these medications during participation in the study is not planned) may also be enrolled (see Inclusion Criterion No. 8 in Section 5.1) as may subjects previously treated with AChEI or memantine provided they have ceased such concomitant treatment ≥ 6 weeks prior to Screening.

It is planned that approximately 1000 subjects will be screened in order for 700 subjects to be randomized (350 subjects in the LMTM treatment group and 350 subjects in the placebo group). Presuming a withdrawal rate of approximately 40% over 78 weeks, it is anticipated that approximately 420 subjects will complete the study (210 subjects in the LMTM group and 210 subjects in the placebo group, assuming unbiased withdrawal). The basis for this sample size is further discussed in Section 10.2.

The study will be conducted at approximately 100-150 study sites in the Americas, Europe, and Australia.

4.3. Duration

The total duration of participation in this study for an individual subject is planned to be up to approximately 88 weeks, including a Screening period of up to 6 weeks (42 days), a double-blind treatment period of 78 weeks, and a post-treatment follow-up approximately 4 weeks (28 ± 7 days) after completion of randomized treatment. Additional follow-up visits may be scheduled as needed to monitor the resolution or stabilization of adverse effects.

It is anticipated that the study will have an overall duration of at least 34 months, depending on recruitment rate. The current study will be concluded after the last visit for the last subject under this protocol.

4.4. Schedule of Assessments

Subjects will be screened at trial sites for determination of eligibility to enter the study on the basis of diagnostic evaluations, mental state assessments, and safety assessments (clinical laboratory testing, vital sign and pulse co-oximetry, complete physical and neurological examinations, and ECG). The CDR total and MMSE will be rated at Screening (Visit 1). FDG-PET and an MRI of the brain will be performed at Screening or within a maximum of 42 days before Baseline. The efficacy assessments, ADAS-cog_{11} and ADCS-ADL_{23}, will be rated at Baseline (Visit 2, pre-dose) for enrolled subjects and repeated on-treatment at Weeks 13, 26, 39, 52, 65, and 78 (or upon early termination) by assessors/raters who are not involved in the assessment of safety (with the exception of the C-SSRS and Serotonin Toxicity Assessment). The ADCS-CGIC can only be rated by an independent rater not involved in other efficacy or safety assessments. (Baseline efficacy assessments can be made on the day before randomization

and dosing.) The MMSE will be rated after 26, 52, and 78 weeks of treatment. All of these assessments will be repeated at the 4-week off-treatment follow-up visit. The secondary efficacy variables, NPI and MADRS, will be rated at Baseline (Visit 2, pre-dose) for enrolled subjects and repeated on-treatment at Weeks 26, 52, and 78 (or upon early termination) by assessors/raters who are not involved in the assessment of safety (see exceptions noted above). These instruments and the rater restrictions are further described in Section 7.2.

The pharmacoeconomic assessment using the RUD Lite will be rated at Baseline (Visit 2, pre-dose), and after 26, 52, and 78 weeks of treatment (see Section 9.2). This assessment is to be made by assessors/raters who are not involved in the assessment of safety (with the exception of the C-SSRS and Serotonin Toxicity Assessment).

Brain MRI (obtained approximately every 13 weeks) will be evaluated for change in WBV to determine whether there is reduction of whole brain, ventricular and hippocampal atrophy rates over the period of the study. FDG-PET will be performed within the 42 days before Baseline and after 39 and 78 weeks. The purpose is to assess reduction in decline of glucose uptake in the temporal lobes. Change in whole brain, ventricular and hippocampal volumes will be quantified at the imaging core laboratory. FDG-PET data will be evaluated by an independent, nuclear physician who is experienced in neuro PET, not involved in the clinical conduct of the study, and trained on the study endpoints; these are further described in Section 9.1.

Safety assessments (described in Section 8) will be performed throughout study participation, including at Baseline (prior to dosing and during the 4-hour post-dose evaluation) and during the treatment period. Study visits during the treatment period will occur at time points approximately 2, 6, 13, 26, 39, 52, 65, and 78 weeks after Baseline. At each in-clinic visit, adverse events (AEs) will be recorded, vital signs measured, ECGs obtained, targeted physical and neurological examinations performed, clinical laboratory testing (*e.g.*, hematology, serum chemistry panels, urinalysis, Vitamin B_{12}, and folate) performed, Columbia-Suicide Severity Rating Scale (C-SSRS) rated, potential for serotonin toxicity assessed, serum pregnancy test performed (women of childbearing potential only), and MetHb and oxygen saturation measured by pulse co-oximetry. All of these assessments will also be conducted at the 4-week post-treatment follow-up visit (Visit 11). TSH will be measured every 26 weeks with a thyroid hormone panel obtained in the event of abnormality. Subjects will also be followed for occurrence of ARIA by MRI performed after 13, 26, 39, 52, 65, and 78 weeks of treatment. Unscheduled visits and housing may occur as needed for assessment.

During intervening times, caregivers will be contacted by telephone at approximately 9, 19, 32, 45, 58, and 71 weeks. Additional telephone contacts will occur in subjects entering the study on serotonergic medication; in addition to in-clinic observation, caregivers will be contacted by telephone at 5–7, >7–14, >14–24, 44–52, and 68–76 hours after the first dose of study drug (with a minimum of 1 hour between contacts). The telephone interview is presented in Section 24.3.

CSF biomarkers will be explored for a subset of subjects who are mentally capable of providing their own separate informed consent and specifically agree to have lumbar puncture performed. Baseline CSF samples may be collected any time prior to the first dose of study drug so long as all screening procedures have been completed and subject eligibility and willingness to continue have been confirmed. A subsequent sample will be collected at the end of treatment (Week 78 or upon early termination).

Apolipoprotein E genotype also will be determined for subjects by or for whom legally acceptable consent is obtained. A single blood sample may be collected any time after eligibility for randomization and continued participation in the study has been confirmed at Baseline (Visit 2).

Blood will be collected for determination of MT concentrations prior to dosing (Visit 2) and approximately 3.5 hours after the first dose. Blood will also be collected at all subsequent visits during the treatment period (after ECG recording). These collections are restricted to those sites with a refrigerated centrifuge and appropriate freezer capacity.

A schedule of assessments is shown in Table 4-1. Assessments are listed by visit (including unscheduled visits) in Appendix 24.1.

Table 4-1 Schedule of Assessments

Visit Name	Screening	Baseline [a]		Treatment Period (78 Weeks) [b]							EOT / ET [c]	Follow-up[d]
		Day 1		*Safety 1*	*Safety 2*	*Efficacy 1*	*Efficacy 2*	*Efficacy 3*	*Efficacy 4*	*Efficacy 5*	*Efficacy 6*	
Overall Visit Number:	1	2 [e]		3	4	5	6	7	8	9	10	11
Weeks Relative to Baseline Day:<br>Scheduled Study Day:<br>(Allowable Time Window in days):	≤ -42	*Pre-Dose*	*Post-Dose*	2<br>15<br>(± 3)	6<br>43<br>(± 3)	13<br>92<br>(± 7)	26<br>183<br>(± 14)	39<br>274<br>(± 14)	52<br>365<br>(± 14)	65<br>456<br>(± 14)	78<br>547<br>(± 14)	82<br>575<br>(± 7)
Informed Consent (Subject and Caregiver) (M)	X											
**Medical History**												
Demographics	X											
Medical History (M) [f,g]	X											
NIA/AA Diagnosis (M) [h]	X											
FDG-PET [i]	←X→							X			X	
MRI (M) [i]	←X→					X	X	X	X	X	X	
CDR Total [j]	X											
MMSE [j,w]	X						X		X		X	X
**Inclusion/Exclusion Criteria**												
Inclusion/Exclusion Criteria Review (M)	X	X										
Modified Hachinski Ischemic Score (M)	X											
Randomization		X										
**Medication**												
In Clinic First Dose			X									
Study Drug Dispensing			X		X	X	X	X	X	X		
Study Drug Compliance Assessment [k]				X	X	X	X	X	X	X	X	
Concomitant Medication Recording/Review (M) [l]	X	X	X	X	X	X	X	X	X	X	X	X
**Safety Assessments**												
Physical and Neurological Examinations (M) [m]	X	X	X	X	X	X	X	X	X	X	X	X
Adverse Events Review (M) [n]	X	X[3]	X[3]	X[3]	X[3]	X[3]	X[3]	X[3]	X[3]	X[3]	X[3]	X[3]
Blood and Urine Samples for Laboratory Tests [o]	X	X[3]		X[3]	X[3]	X[3]	X[3]	X[3]	X[3]	X[3]	X[3]	X[3]
Blood Sample for Vitamin B_{12} and Folate [o]	X	X[3]		X[3]	X[3]	X[3]	X[3]	X[3]	X[3]	X[3]	X[3]	X[3]
Blood Sample for G6PD, Haptoglobin[o]	X											
Thyroid Stimulating Hormone [p]	X						X		X		X	
Pulse Co-oximetry [q]	X	X[3]	X[3]	X[3]	X[3]	X[3]	X[3]	X[3]	X[3]	X[3]	X[3]	X[3]
Temperature, Respiratory Rate [r]	X	X	X	X	X	X	X	X	X	X	X	X
Blood Pressure and Pulse [s]	X	X	X	X	X	X	X	X	X	X	X	X
Serotonin Toxicity Assessment (M) [t]		X	X	X	X	X	X	X	X	X	X	X
Height	X											
Weight	X	X			X	X	X	X	X	X	X	X
12-Lead Electrocardiogram [u]	X	X	X	X	X	X	X	X	X	X	X	X
C-SSRS [v]	X		X	X	X	X	X	X	X	X	X	X
**Efficacy Assessments**												
ADAS-cog_{11} [w]		X[1]				X[1]	X[1]	X[1]	X[1]	X[1]	X[1]	X[1]
ADCS-CGIC [w]		X[2]				X[2]	X[2]	X[2]	X[2]	X[2]	X[2]	X[2]
ADCS-ADL_{23} [w]		X[1]				X[1]	X[1]	X[1]	X[1]	X[1]	X[1]	X[1]
NPI [x]		X[1]					X[1]		X[1]		X[1]	
MADRS [w]		X[1]					X[1]		X[1]		X[1]	
**Other**												
RUD Lite [y]		X[1]					X[1]		X[1]		X[1]	
Blood Sample for MT Concentration [z]		X	X	X	X	X	X	X	X	X	X	
Blood Sample for Genotyping (optional)		X[aa]										
Cerebrospinal Fluid Sample (M) (optional)		X[bb]									X[bb]	

Notes: (M) = requires medical assessor
X = any study rater including X^1, X^2, or X^3 (ADCS-CGIC rater cannot also rate MMSE; see X^2 below)
X^1 = metrics rater (cannot rate X^2 or X^3)
X^2 = independent ADCS-CGIC rater (cannot rate X^1, X^3, or MMSE and is blinded to ADAS-cog_{11}, ADCS-ADL_{23} and other efficacy results)
X^3 = independent adverse events reviewer (cannot rate X^1 or X^2)

[a] The Baseline visit (Visit 2) may span over 2 days if necessary; Day 1 will commence with the administration of the first in-clinic dose.

[b] Study visits during the treatment period are to occur within stipulated time windows. If a visit is delayed, the next visit should occur earlier to return to the schedule. If assessments/visits are performed outside of the stipulated time windows, these should be categorized/labeled according to the intended visit designation, regardless of being out of window (with the exception of an assessment/visit that is significantly delayed such that it falls into the stipulated time window for the following visit; in these cases, the label to be applied should be that of the following visit). An unscheduled visit is to take place if needed in response to a safety concern; an assessment/visit should only be labeled as unscheduled if it is a visit which was not planned and which was performed in addition to the protocol-defined visits. After the Week 6 visit (Visit 4), a documented telephone contact will be scheduled to occur at Weeks 9, 19, 32, 45, 58, and 71 (± 7 days) and will include AE and concomitant medication review. Additional telephone contacts will occur in subjects entering the study on serotonergic medication at 5–7, >7–14, >14–24, 44–52, and 68–76 hours relative to the first dose (with a minimum of 1 hour between contacts).

[c] The end of treatment (EOT) is defined as completion of the Week 78 visit (Visit 10), or last dose of study drug for subjects who terminate early. If a subject discontinues study drug and/or withdraws from the study prematurely (before completion of Visit 10), then an early termination (ET) visit should be conducted at which time all assessments identified for Visit 10 should be performed.

[d] After completion of the off-treatment safety follow-up assessments at Week 82 (Visit 11), approximately 28 (± 7) days after the Week 78 visit, subjects may be considered for open-label treatment with LMTM in an extension study (separate protocol).

[e] At Visit 2, Baseline assessments (in *Pre-Dose* column) will be performed before administration of the first dose of study drug on Day 1, with the exception of the Efficacy assessments which may be performed on the prior day if necessary; see footnote bb for timing of CSF sample collection. Assessments in the *Post-Dose* column for Visit 2 will be performed after administration of the first dose of study drug, with timing as specified for each parameter.

[f] Medical history within the past 10 years and any other previous history considered clinically relevant by the investigator will be recorded.

[g] Diagnostic and Statistical Manual of Mental Disorders, Fourth Edition – Text Revision (DSM-IV-TR)

[h] National Institute on Aging – Alzheimer's Association (NIA/AA)

[i] ^{18}F-fluorodeoxyglucose positron emission tomography (FDG-PET) and magnetic resonance imaging (MRI) scans will be performed as described in separate FDG-PET and MRI protocols (provided to study sites). The Screening scans must be obtained at Screening or within 42 days before Baseline; results (of sufficient quality) must be available at the Baseline visit for the purpose of inclusion/exclusion review and confirmation of compliance with inclusion and exclusion criteria by an independent neuroradiologist. If the initial Screening/Baseline FDG-PET or MRI scan is not of sufficient quality, then a repeat Screening/Baseline scan may be performed (within 42 days before Visit 2). If the repeat scan cannot be accomplished within the 42-day window, then the subject must be reconsented and rescreened. For subjects who are rescreened for other reasons and an acceptable FDG-PET or MRI scan was already completed during the original screening window, the scan should only be repeated if the original FDG-PET or MRI scan occurred > 42 days prior to Visit 2. The Weeks 39 and 78 scans should be performed within ± 14 days of the target visit. The Week 39 FDG-PET scan can be repeated if the central reader determines a quality issue; the Week 78 FDG-PET scan cannot be repeated. For subjects who terminate early, if the subject's last FDG-PET or MRI scan was performed < 90 days prior to the early termination date, no additional scan is required. If the subject's last FDG-PET or MRI scan was performed ≥ 90 days prior to the early termination date, the FDG-PET or MRI scan must be repeated as part of the early termination visit assessments within the time window of the early termination visit (*i.e.*, ± 14 days of the last dose of study drug). Allowable time window extensions are discussed further in Section 9.1.3. Post-Baseline MRI scans (performed every 13 weeks) will be reviewed by an independent neuroradiologist for evidence of ARIA and quantified at the imaging core laboratory for change in whole brain, ventricular and hippocampal volumes; scans will be repeated every 6 weeks in cases where ARIA is noted to follow resolution/stabilization of ARIA (stabilization is to be based on three follow-up scans).

[j] At Screening, a Clinical Dementia Rating (CDR) total score and MMSE score will be obtained for eligibility determination; these will be subject to independent review. It is permissible for CDR to be undertaken by the independent ADCS-CGIC rater.

[k] Compliance with study drug will be assessed by counting returned tablets (see Section 6.6).

[l] At the Screening visit (Visit 1), medications administered within the past 3 months will be recorded; lifetime history of AChEI and/or memantine use will also be recorded. Concomitant medication use will be reviewed and recorded throughout the study.

[m] Complete physical and neurological examinations will be performed at Screening. Targeted examinations will be performed pre-dose and approximately 3 hours after administration of the first dose of study drug (Visit 2) (following the ECG measurements); these are to be repeated as needed for subjects who remain in clinic longer than 4 hours. Thereafter, targeted examinations are to be performed at each subsequent visit (or upon early termination), including the 4-week post-treatment follow-up visit (Visit 11).

[n] Adverse events will be evaluated and recorded on an ongoing basis starting after signing of informed consent, including at each scheduled visit and at each scheduled telephone contact.

[o] At each identified time point, blood and urine samples will be obtained for chemistry and hematology panels, Vitamin B_{12} levels, folate levels, and urinalysis. At Screening only, haptoglobin levels will be measured and subjects will be screened for G6PD deficiency. For women of childbearing potential, a serum pregnancy test will be performed at Screening, each subsequent study visit during the treatment period (or upon early termination), and at the 4-week follow-up visit.

[p] Thyroid panels (T_3 and T_4) are to be measured at the next visit if TSH is abnormal; further follow-up is to be performed as needed (see Section 8.3.4)

[q] Methemoglobin and oxygen saturation will be measured using a provided pulse co-oximetry device. At Visit 2, measurements will be taken within 1 hour before and approximately 2.5 hours after administration of the first dose of study drug.

[r] At Visit 2, oral (sublingual) temperature and respiratory rate will be measured within 1 hour prior to dosing and then hourly after the first dose of study drug until discharge from the study unit (*i.e*., for at least 4 hours). At discharge, subjects (and their caregivers) using any medication with the potential to increase synaptic levels of serotonin will be instructed on signs and symptoms of potential serotonergic toxicity and given an oral thermometer and diary to record the subject's temperature three times a day (in the morning, afternoon, and evening) for 72 hours. The diary is to be returned to the site at Visit 3.

[s] At Screening and within 1 hour prior to dosing on Day 1, blood pressure and pulse will be measured with subjects in a seated position (for at least 5 minutes) and again 2 minutes after standing; the post-dose measurement on Day 1 is to be made approximately 2 hours post-dose. Thereafter, blood pressure and pulse will be measured with subjects in the seated position only (for at least 5 minutes).

[t] Serotonin toxicity assessments are to be performed for all subjects pre-dose and for at least 4 hours after the first dose of study drug while subjects are in clinic using the Serotonin Toxicity (Syndrome) Diagnostic Interview and Rating Guide (Section 24.2); an assessment is to be completed just prior to discharge from the clinic. Caregivers of subjects receiving serotonergic medication will be contacted 5–7, >7-14, >14–24, 44–52, and 68–76 hours post-dose (with a minimum of 1 hour between contacts) and queried for the presence of signs and symptoms of serotonin toxicity using the Serotonin Toxicity Telephone Assessment (Section 24.3); if indicated, more frequent contacts will be made.

[u] A 12-lead ECG will be obtained in triplicate (within an approximate 2- to 5-minute interval) at the Screening visit and at Visit 2; subsequently (at all other visits or upon early termination), single recordings will be made, unless there is an emergent abnormality which is of clinical significance in the judgment of the investigator, in which case triplicate ECG recordings should be made. For subjects with well controlled atrial fibrillation at Baseline (heart rate ≤ 84 bpm and appropriate anticoagulation), triplicate ECGs are mandatory at every visit after Visit 2. At Visit 2, ECGs will be obtained before administration of the first dose of study drug (dose should be held in response to any significant abnormalities based on local interpretation) and then again approximately 3 hours post-dose. Refer to Section 6.3.3.4 for guidance regarding selected QT interval and ECG abnormalities.

[v] On Day 1 (Visit 2), the Columbia-Suicide Severity Rating Scale (C-SSRS) will be used to evaluate suicidal behavior and suicidal ideation post-dose (prior to discharge from the clinic)

[w] Efficacy assessments that will involve the subject directly include the following: Alzheimer's Disease Assessment Scale – cognitive subscale (11-item) (ADAS-cog_{11}), Alzheimer's Disease Cooperative Study – Clinical Global Impression of Change (ADCS-CGIC), Mini-Mental State Examination (MMSE), and Montgomery-Asberg Depression Rating Scale (MADRS). Alzheimer's Disease Cooperative Study – Activities of Daily Living (23-item) (ADCS-ADL_{23}) is administered to the caregiver. At each applicable time point, efficacy assessments should be performed before ECG recordings, vital signs measurements, and blood sample collection.

[x] Neuropsychiatric Inventory (NPI)

[y] Resource Utilization in Dementia Questionnaire (RUD) Lite

[z] At Visit 2, blood will be collected on two occasions, pre-dose and again approximately 3.5 hours post-dose (only at sites with a refrigerated centrifuge and adequate freezer capacity); time of dose and sample are to be recorded at each collection. Thereafter, blood samples are to be obtained at each designated visit at a time point within approximately 20 minutes after completion of ECG recording measurement. Timing of blood collection relative to last dose should be recorded.

[aa] A single blood sample for genotyping will be obtained only from subjects by or for whom legally acceptable informed consent for this is provided; the blood sample may be collected any time after eligibility for randomization and continued participation in the study has been confirmed.

[bb] Cerebrospinal fluid samples will be obtained at designated study sites only from subjects who are mentally capable of providing their own separate informed consent and specifically agree to have lumbar puncture performed. These samples will be obtained using standard lumbar puncture procedures and will be analyzed for specified biomarkers. Baseline CSF samples may be collected any time prior to the first dose of study drug so long as all screening procedures have been completed and subject eligibility and willingness to continue have been confirmed. A subsequent sample will be collected at the end of treatment (Week 78 or upon early termination).

4.5. Randomization and Blinding

Eligible subjects will be randomly allocated to LMTM 200 mg/day or placebo groups in a 1:1 ratio so that the numbers of subjects assigned will be approximately 350 for the LMTM group

and 350 for the placebo group. Randomization will be on an overall study basis because of the large number of study sites. A central randomization list will be used, with the randomization scheme stratified by geographic region (the Americas or Europe/Australia), use of AChEI and/or memantine (current ongoing use or not ongoing use), and screening severity (CDR 0.5 or 1). The relative number and ratio of subjects in the placebo group *vis à vis* the LMTM group were selected in part to adequately assess progression of AD without study treatment (also see Section 10.2).

After signing consent, subjects will be assigned a unique subject identification number (005-CC-SSS-EE), with the first three digits for the study (005), the next two digits for the country, the next three digits for the site, and the last two digits for the sequential order of enrollment at a given site. Subjects who are rescreened will receive a new identification number; the previous number is also to be recorded by the site. After Screening assessments are performed and eligibility has been confirmed at the Baseline visit, an eligible subject will be randomized. This will be accomplished by the study site using an interactive web response system (IWRS) to assign the next sequential randomization number within the relevant stratum to the eligible subject.

Treatment assignment will remain unknown to the subject and caregiver as well as to the investigator and study site personnel (double-blind) throughout a subject's participation in the study. The randomization list will be maintained in a secure location by individuals who are not directly involved in the conduct of the study. At the end of the study, after the database is locked and subject populations have been determined, treatment assignments will be unblinded.

Regardless of randomized assignment, subjects in the two treatment groups will receive the same regimen of study drug tablets (one tablet *b.i.d.*), and all study drug tablets (LMTM and placebo) will be indistinguishable from one another in appearance. Because MT coloration occurs upon exposure to oxygen and treatment with LMTM is associated with coloration of urine and/or feces, the placebo group study drug regimen will include a low dose of LMTM (8 mg/day total; see also Section 6.1.1).

The blind for an individual subject should not be broken during conduct of the study except in the case of a medical situation for which it is deemed essential to know which treatment the subject has received in order to provide appropriate care. The investigator may in an emergency unblind a specific subject and determine the identity of treatment using the IWRS. Instructions regarding treatment identification using the IWRS will be available in separate guidance documents. In such circumstances, the medical monitor <u>must</u> be contacted and informed of any unblinding as soon as possible. The date, time, and reason for unblinding must be documented.

If a subject is unblinded, study drug will be discontinued and the subject will be followed until resolution or stabilization of the event. He/she will then be discontinued from the study.

Information about any subject for whom a code break or unblinding occurs will be provided to the DSMB by the Sponsor or designee within 15 days (within 7 days in the event of a fatal event).

Information regarding unblinding treatment allocation in relation to reporting suspected unexpected adverse reactions (SUSARs) is provided in Section 8.1.6.

4.6. Study Treatment

The total daily doses planned are LMTM 200 mg/day or placebo. As the placebo contains a small amount of LMTM (4 mg) in order to color the urine and feces, the actual total amounts of MT ingested daily are 200 mg/day or 8 mg/day.

Subjects will be prescribed a total of two study drug tablets daily throughout the 78-week treatment period, administered as one tablet *b.i.d.* (one tablet in the morning and one tablet in the evening). Two 50-mg reductions are allowable (to 150 mg/day and then 100 mg/day). This will be carried out in a manner to maintain blinding; if there is a requirement for dose reduction, alternative study drug packages will be supplied. Treatment may also be interrupted for up to (maximum duration on any one occasion) 30 days during the first 6 months of treatment and/or 14 days during the second 12 months of treatment if necessary.

Study drug and regimens are further described in Section 6.1 and Section 6.2.

4.7. Concomitant Medication

All concomitant medications and medications administered within the past 3 months, as well as a lifetime history of AChEI and/or memantine use, will be recorded at the Screening visit (Visit 1). While in the clinic at Baseline (Visit 2; before and after the first dose of study drug is administered), any medications administered will be recorded. “Medication” is used to encompass prescription and over-the-counter drugs or biologics, vitamins used in supra-pharmacologic doses, alternative pharmacotherapies for dementia, medical foods, and for women, forms of contraception. At each scheduled outpatient visit (Visits 3-11), and at each scheduled telephone contact during the treatment period, any changes to existing concomitant medication and any new concomitant medication will be reviewed and recorded.

Concomitant medications identified at Screening generally should be maintained at a constant dose for the duration of the study if clinically indicated. The investigator should evaluate any changes in the dose of existing concomitant medications and/or initiation of new concomitant medications, and the medical monitor should be contacted to discuss any concerns as needed. The date of commencement, dose, and date of any change of dose of concomitant medications are to be recorded in the electronic case report form (eCRF).

4.7.1. Dementia Medication

Subjects taking an AChEI, memantine, or both may be enrolled if the subject has been taking such medication(s) for ≥ 3 months at Screening, if the current dosage regimen is within the locally approved dose range, if the AChEI and/or memantine total daily dose has remained unchanged for ≥6 weeks prior to Screening, and if the dose is not expected to change during a subject’s participation in this study (see Inclusion Criterion No. 8 in Section 5.1). The only exception is that tacrine is not allowed. A subject who has variability in the prescribed AChEI and/or memantine dose during the 6-week period prior to Screening or who is consistently not compliant with the regimen should not be enrolled.

Ideally, the AChEI and/or memantine dosage and time of dosing should remain unchanged during participation in the study. Due to lack of availability, change from immediate-release memantine to the extended-release formulation (*e.g.*, from 10 mg twice daily to 28 mg once daily) is acceptable. MT may inhibit cytochrome P450 3A4 (CYP3A4), an enzyme system that is involved in the metabolism of two of the AChEIs allowed in the study, donepezil and galantamine. Because drug-drug interaction studies have not yet been performed with these

drugs, it is not known whether systemic exposure to either of these drugs will increase or by how much. Therefore, subjects receiving these drugs should be monitored and if adverse events suggest an increase in systemic exposure, dose adjustment of donepezil or galantamine may be considered. If any change in dose occurs for whatever reason, the subject should continue on study drug unless it is judged to pose a risk to the subject.

Subjects not currently being treated with an AChEI or memantine are also eligible for enrollment if it is anticipated that such medication(s) will not be (re)started during participation in the study. If such a subject has previously taken an AChEI and/or memantine, the last dose must have been taken ≥ 6 weeks prior to Screening.

Subjects who have been receiving a medical food (*e.g.*, Axona, Souvenaid) or a stable dose of alternative pharmacotherapy for dementia (*e.g.*, Vitamin E, folate [in doses up to 5 mg/day; doses of approximately 1 mg/day in the management of folate deficiency are acceptable], a specific neurocognitive vitamin formulation [such as NeuroVits comprising 20 mg Vitamin B_6, 1 mg Vitamin B_{12}, 0.8 mg folate (see Douaud *et al*., 2013)], ginkgo biloba, hormone replacement therapy, treatments related to coconut oil, curcumin) may continue such treatment, but the dose of such therapy must have remained stable for ≥ 6 months before randomization and it must remain constant for the duration of the subject's participation in the study. With the exception of folate, starting such therapy during participation in the study must be avoided.

There are no restrictions for other non-pharmacological treatments during the treatment period.

4.7.2. Drugs with Serotonergic Potential

As the oxidized form of MT (MT^+) is an inhibitor of MAO A and B *in vitro*, due to the potential for drug interaction resulting in serotonin syndrome (toxicity), recent (within five to seven plasma half-lives) or current treatment with any of the following medications (as identified by FDA in the October 2011 Safety Alert and augmented to include drugs not approved in the United States) should be undertaken only if the potential benefit is judged to outweigh the risk. A list (not exhaustive) of such medications and their plasma half-lives (majority taken from U.S. approved product labeling) is given below; see the Investigator's Brochure for the most current information and examples of drugs with serotonergic potential. It should be noted that some of these are available without prescription and their use should also be recorded in the eCRF.

In addition to in-clinic observation (all subjects are to be monitored in the clinic for at least 4 hours after the start of double-blind study drug), additional telephone contacts and monitoring will occur for subjects entering the study on serotonergic medication; see Section 6.3.3.2 for further information.

Drugs with serotonergic potential should not be initiated during the study; alternative therapies should be sought. The subject's general practitioner will be informed of this prohibition in writing. If such medication must be started, the in-clinic and follow-up procedures described in Section 6.3.3.2 are to be followed.

**Generic Name**<br>**Metabolite (if plasma half-life available)**	**Plasma Half-life**<br>**Range (Mean, if available)**
**Selective Serotonin Reuptake Inhibitors (SSRIs)**	
Citalopram	35 hours (30-50% increase in half-life reported in elderly)
Dapoxetene	Biphasic (initial: 1.5 hours; terminal: 20 hours)
Escitalopram	27-32 hours (50% increase in half-life reported in elderly)
Fluoxetine	Acute: 1-3 days, Chronic: 4-6 days
Norfluoxetine	4-16 days (9.3 days)
Fluvoxamine	15.6 hours
Paroxetine	21 hours
Sertraline	26 hours
N-desmethylsertraline	62-104 hours
Vilazodone	25 hours
**Serotonin Norepinephrine Reuptake Inhibitors (SNRIs)**	
Desvenlafaxine	11 hours
Duloxetine	8-17 hours (12 hours); 4-hour increase in elderly half-life
Milnacipran	6-8 hours
d-milnacipran	8-10 hours
l-enatiomer	4-6 hours
Venlafaxine	3-7 hours
O-desmethylvenlafaxine	9-13 hours
**Tricyclic Antidepressant (TCA)**	
Amitriptyline	9-25 hours
Clomipramine	19-37 hours (32 hours)
Desmethylclomipramine	54-77 hours (69 hours)
Desipramine	12-27 hours
Dosulepin (Dothiepin)	19-33 hours (23-46 hours for “its metabolites”)
Doxepin	15.3 hours
Nordoxepin	31 hours
Desmethyldoxepin	28-52 hours
Imipramine	9-28 hours
Nortriptyline	16-93 hours
Protriptyline	55-198 hours
Trimipramine	11-23 hours
**Monoamine Oxidase Inhibitors**	
Isocarboxazid	Unknown
Moclobemide	1-2 hours
Phenelzine	11.6 hours (single-dose)
Selegiline	10 hours
R(-)-N-desmethylselegiline	18-25 hours
R(-)-amphetamine	18-25 hours
R(-)-methamphetamine	18-25 hours
Toloxatone	Unknown
Tranylcypromine	2.5 hours
**Other Psychiatric Medicines with Serotonergic Activity**	
Amoxapine	8 hours
8-hydroxyamoxapine	30 hours
Bupropion	12-30 hours (21 hours)
Hydroxybupropion	15-25 hours (20 hours)
Erythrohydrobupropion	23-43 hours (33 hours)
Threohydrobupropion	24-50 hours (37 hours)
Buspirone	2-3 hours (single dose)
Maprotiline	51 hours
Mirtazapine	20-40 hours
Nefazodone	2-4 hours
Trazodone	10 hours (single dose)

Numerous other non-psychiatric medications also have the potential to interact with the serotonergic system and their use is similarly restricted (*i.e.*, use restricted to those subjects treated at Baseline with additional monitoring by telephone after the first dose of study drug). These include drugs such as:

- MAO inhibitors used for non-psychiatric indications (*e.g.*, rasagiline, isoniazid, procarbazine, linezolid)
- Other medications associated with severe serotonin toxicity (*e.g.*, tramadol, pethidine, fentanyl, chlorphen(ir)amine, dextromethorphan, tryptophan, dexamphetamine)
- Lithium, valproate, metoclopramide, propranolol, ergot alkaloids (bromocriptine, methysergide)
- Drugs used for weight loss (lorcaserin)
- "Triptans" used in the treatment of migraine (sumatriptan, rizatriptan, naratriptan, zolmitriptan, eletriptan, almotriptan, frovatriptan)
- St. John's wort (*Hypericum perforatum*) and *panax ginseng*

Note, dietary supplements containing tryptophan or its metabolite 5-hydroxytryptophan (5-HTP) are considered serotonergic concomitant medications as 5-HTP is a precursor in the biosynthesis of serotonin. Tryptophan is a naturally-occurring protein, however, eating foods containing tryptophan does not significantly increase 5-HTP levels; thus, avoiding dietary sources of tryptophan is not necessary.

In the event of any question, the medical monitor should be consulted.

4.7.3. CYP and P-gp Substrates

Results of a completed drug-drug interaction study indicate that MT is a weak inhibitor of CYP3A4, CYP2C8, and CYP2C19 enzymes (see the Investigator's Brochure for examples of drugs metabolized by these enzymes). The extent to which this occurs within an individual or with a given drug is not known, especially for those drugs with multiple metabolic pathways. Therefore, subjects on drugs known to be metabolized by one or more of these enzymes (especially those that have a narrow therapeutic index) should be closely monitored for adverse events that could suggest an increase in systemic exposure. Dose adjustment of the concomitant medication may be warranted.

LMTM is also a weak inducer of CYP2B6 and the P-glycoprotein (P-gp) transporter (see the Investigator's Brochure for examples of substrates). Coadministration of LMTM with digoxin, a P-gp substrate, was shown to result in decreased concentrations of digoxin. Therefore, it is advisable to obtain a baseline digoxin level in subjects on this drug and to monitor digoxin levels while on study. Any such results obtained from the local laboratory should be entered into the eCRF (together with the time of the prior dose of digoxin and the time of the sample).

4.7.4. Drugs Used to Manage Behavioral Disturbance

Subjects may be treated with antipsychotics (other than clozapine or olanzapine) provided they have been used in a stable dose and regimen for at least 3 months prior to Baseline. There should be no intent to initiate such therapy during the course of the study. Should treatment be initiated, the reason(s) should be clearly documented by indicating one or more of the following reasons: delusions, hallucinations, agitation/aggression, depression, anxiety, elation/euphoria, apathy/indifference, disinhibition, irritability/lability, aberrant motor behavior, nighttime behavior, or appetite/eating change.

"As-needed" use of antipsychotics is to be avoided if possible, but such use does not preclude further participation. Similarly, regular or occasional use of benzodiazepines to manage distress, agitation, *etc.* does not preclude further participation, with the exception of prior to FDG-PET scans (when it is not permitted). Where possible, these should not be used within the 12 hours prior to cognitive testing.

4.7.5. Other Medications

Other medications listed in Exclusion Criterion No. 18 (see Section 5.2) are specifically prohibited during participation in this study.

Anxiolytics and/or sedatives/hypnotics may be used as sedation for claustrophobia or agitation or to manage excessive movement during MRI scans; however, use of sedatives prior to FDG-PET scans is not permitted. Regular or occasional benzodiazepines, chloral hydrate, low dose trazodone[4] (50 mg), or zolpidem may be used as needed at bedtime for sleep.

Unless otherwise prohibited, concomitant medications (preferably at stable doses) considered appropriate by the subject's physician are allowable but should be kept to the minimum possible as clinically indicated. If there are questions about whether or not a medication is permitted in the study, the medical monitor should be consulted.

4.7.6. Dietary Tyramine

Historically, MAO inhibitors as a class have been reported to be associated with hypertensive crises caused by ingestion of foods containing high amounts of tyramine (known as a tyramine or "cheese" reaction). While there is a theoretical potential for a tyramine reaction with MT, there have been no reports to date in subjects taking part in TauRx-sponsored studies, even though there have been no dietary restrictions in these studies. Nonetheless, as a precaution, subjects and their caregivers should be advised about this potential while taking LMTM (see the Investigator´s Brochure for examples of tyramine-rich foods and beverages, such as air-dried, aged or fermented meats and cheeses, fava bean pods, non-pasteurized beers, sauerkraut, and most soybean products). They should also be advised to seek medical care immediately in the event of signs or symptoms of hypertensive crisis (sudden onset of severe headache, nausea, stiff neck, tachycardia or palpitations, profuse sweating, and/or confusion) or other sudden or unusual symptoms following ingestion of tyramine-rich foods or beverages.

4.7.7. Contraceptive Measures

As a precautionary measure, women of childbearing potential (*i.e.*, not documented to be post-menopausal for at least 1 year or not having undergone hysterectomy or bilateral salpingectomy or oophorectomy for at least 6 months minimum) must use adequate contraception, with the exception of female subjects in Italy. Examples of adequate contraception include bilateral tubal ligation or occlusion at least 6 months prior to Baseline; use of a barrier method (condom, diaphragm or cervical/vault cap) with spermicidal foam, gel, film, cream, or suppository; intrauterine device (IUD) or system, or oral or long acting injected or implanted hormonal contraceptives for at least 3 months prior to Baseline; or sexual activity restricted to a vasectomized partner (with the appropriate post-vasectomy documentation of the absence of

---

[4] If trazodone is used in this way, steps must be implemented for monitoring signs and symptoms indicative of potential serotonin toxicity.

spermatozoa in the ejaculate). Abstinence is only acceptable as true abstinence when this is in line with the subject's preferred and usual lifestyle; periodic abstinence (*e.g*., calendar, ovulation, symptothermal, post-ovulation methods) and withdrawal are not acceptable methods of birth control. Subjects must be competent to use adequate contraception and must agree to continue to maintain adequate contraceptive measures throughout study participation and until the final off-treatment visit. In Italy, subjects must have avoided a pregnancy for at least 3 months prior to Baseline and must accept to avoid a pregnancy throughout participation in the study. Women of childbearing potential should be encouraged to return to the clinic in the event of a delayed menstrual period to rule out possible pregnancy.

Males should practice abstinence or use acceptable birth control with any female sexual partner of childbearing potential. The risk of drug secretion through the ejaculate is not fully studied. To ensure that the fetus is not exposed to MT through vaginal absorption, male subjects (including men who have had vasectomies) whose partners are pregnant should use condoms for the duration of the study and for an additional 4 days after cessation of study treatment. The investigator must provide appropriate counsel to male subjects regarding this issue.

4.7.8. Folate and Vitamin B_{12}

The manufacturer of the test kits used by the central laboratory for measuring folate and Vitamin B_{12} (see Section 8.3.4) has established the following normal ranges (healthy U.S. males and females aged 18 years and older): 5.9-24.8 ng/mL for folate and 180-914 pg/mL for Vitamin B_{12}. However, these values are not applicable for all geographies as the food in the United States is supplemented with these vitamins. Prior to initiating study drug, subjects with folate levels < 4.0 ng/mL or Vitamin B_{12} levels < 150 pg/mL (*i.e.*, deficient according to WHO Technical Consultation, 2008, 2012), should be supplemented. Subjects with folate levels < 4.0 ng/mL may be entered into the study provided they are supplemented (approximately 1 mg/day folate) from Visit 2 onwards. Subjects with Vitamin B_{12} levels < 150 pg/mL should be referred to their primary care physician for evaluation and treatment or the medical monitor consulted. There must be a treatment plan in place for any applicable chronic condition. If a condition is diagnosed that the primary care physician believes cannot be reliably or continuously corrected, the subject should be excluded from the study. If review and correction can be achieved within the 42-day screening window, the subject may be entered into the study; otherwise the subject must be reconsented and rescreened after the deficit has been corrected.

Folate and Vitamin B_{12} are necessary for the production and function of red blood cells. Given the risk of anemia with the use of MT, it is important that subjects not become deficient in folate and/or Vitamin B_{12} while on study. Furthermore, low levels of either could be a confounding factor contributing to cognitive impairment (Allen *et al.*, 2013; Moore *et al.*, 2012; Nachum-Biala and Troen, 2012; O'Leary *et al.*, 2012; Malouf and Grimley, 2008; Malouf and Sastre, 2003). For these reasons, folate and Vitamin B_{12} are to be monitored throughout the study. Subjects should be referred to their primary physician for evaluation of causation and correction or the medical monitor consulted in the event that levels of either become deficient. For Vitamin B_{12}, this evaluation may also include measurement of homocysteine and methylmalonic acid (by a local laboratory) to determine whether or not the low levels are physiologically significant.

Chronic hemolytic anemia depletes folate stores, and it is general medical practice to place all hematology patients with chronic hemolytic anemia on prophylactic folate replacement, with a recommended dose of 1 mg by mouth, per day.

4.8. Data and Safety Monitoring Board / Independent Data Monitoring Committee

Safety will be overseen by a DSMB throughout the duration of study conduct. At any time, the DSMB may recommend that dosing be modified or enrollment stopped due to safety concerns; the DSMB may also request to receive additional data unblinded to the subject level in response to identified safety concerns.

Routine meetings are to be scheduled as determined by the DSMB. *Ad hoc* meetings will be convened if needed in response to safety concerns. This DSMB will also be assessing data from other clinical studies sponsored by TauRx with the same active moiety. The DSMB Charter will describe the composition of the DSMB and safety monitoring details, as well as the frequency of meetings needed as the study progresses.

5. SELECTION OF SUBJECTS AND CRITERIA FOR WITHDRAWAL

Subjects may be identified from existing patient populations, by advertisement, or referred by a call center for preliminary eligibility assessment. Subjects referred by the call center will be prescreened prior to referral to trial sites using a variable set of questions derived from a pool of subject or carer dementia rating questionnaires aiming to predict MMSE score without using the MMSE itself, to limit referral of potentially interested subjects to those expected to be in the MMSE 20-26 severity range and potentially meeting other eligibility criteria. Only trial sites (in conjunction with independent imaging reviewers) will determine eligibility for entry into the study, irrespective of referral source.

5.1. Inclusion Criteria

To be eligible for enrollment in this study, a subject must meet all of the following inclusion criteria:

1. Diagnosis according to the National Institute on Aging (NIA) and Alzheimer's Association (AA) criteria of:
   - All cause dementia

   and
   - Probable Alzheimer's disease
2. Clinical Dementia Rating (CDR) total score of 0.5 or 1 (mild) and MMSE score of 20-26 (inclusive) at Screening
3. Age < 90 years at Screening
4. Modified Hachinski ischemic score of ≤ 4 at Screening
5. Females must meet one of the following:
   - Surgically sterile (hysterectomy, bilateral salpingectomy / oophorectomy) for at least 6 months minimum
   - Have undergone bilateral tubal occlusion / ligation at least 6 months prior

- Post-menopausal for at least 1 year
- Using adequate contraception (a barrier method [such as condom, diaphragm or cervical/vault cap] with spermicidal foam, gel, film, cream, or suppository; intrauterine device [IUD] or system, or oral or long-acting injected or implanted hormonal contraceptives for at least 3 months prior to Baseline; or vasectomized partner [with the appropriate post-vasectomy documentation of the absence of spermatozoa in the ejaculate]) or true abstinence (when this is in line with the preferred and usual lifestyle of the subject); subjects must be competent to use adequate contraception and to agree to maintain adequate contraception throughout participation in the study
  OR
  In Italy, have avoided a pregnancy for at least 3 months prior to Baseline and accept to avoid a pregnancy throughout participation in the study

6. Subject and/or, in the case of reduced decision-making capacity, legally acceptable representative(s) consistent with national law is/are able to read, understand, and provide written informed consent in the designated language of the study site
   - In Germany, subjects must be able to provide their own written informed consent (see Sections 14 and 15)
7. Has one or more identified adult caregivers who meet the following criteria:
   - Either lives with the subject or sees the subject on average for ≥ 2 hours/day ≥ 3 days/week, or in the investigator's opinion, the extent of contact is sufficient to provide meaningful assessment of changes in subject behavior and function over time and provide information on safety and tolerability
   - Is willing to provide written informed consent for his/her own participation
   - Is able to read, understand, and speak the designated language at the study site
   - Agrees to accompany the subject to each study visit
   - Is able to verify daily compliance with study drug
8. If currently taking an acetylcholinesterase inhibitor (AChEI), *i.e.*, donepezil, galantamine, or rivastigmine, and/or memantine, at the time of Screening:
   - The subject must have been taking such medication(s) for ≥ 3 months
   - The current dosage regimen and dosage form must be within the locally approved dose range and must have remained stable for ≥ 6 weeks
   - It must be planned that the dosage regimen will remain stable throughout participation in the study

   Subjects not being treated with an AChEI or memantine (for ≥ 6 weeks before Screening) may also be enrolled if initiation of an AChEI or memantine is not planned for the time period during which the subject will be participating in this study
9. Able to comply with the study procedures in the view of the investigator

5.2. Exclusion Criteria

In order to be eligible for this study, a subject must not meet any of the exclusion criteria listed below:

1. Significant CNS disorder other than Alzheimer's disease, *e.g.*, Lewy body dementia, Parkinson's disease, multiple sclerosis, progressive supranuclear palsy, hydrocephalus, Huntington's disease, any condition directly or indirectly caused by Transmissible Spongiform Encephalopathy (TSE), Creutzfeldt-Jakob Disease (CJD), variant Creutzfeldt-Jakob Disease (vCJD), or new variant Creutzfeldt-Jakob Disease (nvCJD)
2. Significant intracranial focal or vascular pathology seen on brain MRI scan within a maximum of 42 days before Baseline that would, based on the independent reviewer imaging evaluation, lead to a diagnosis other than probable Alzheimer's disease or that puts the subject at risk of Amyloid Related Imaging Abnormalities (ARIA), including:
    - Large confluent white matter hyperintense lesions (*i.e.*, Fazekas score of 3)
    - Other focal brain lesion(s) judged clinically relevant by the investigator
    - A single area of superficial siderosis
    - > 4 Cerebral microhemorrhages (regardless of their anatomical location or diagnostic characterization as "possible" or "definite")
    - Evidence of a prior macrohemorrhage
3. Clinical evidence or history of any of the following within specified period prior to Baseline:
    - Cerebrovascular accident (2 years)
    - Transient ischemic attack (6 months)
    - Significant head injury with associated loss of consciousness, skull fracture or persisting cognitive impairment (2 years)
    - Other unexplained or recurrent loss of consciousness ≥ 15 minutes (2 years)
4. Epilepsy (a single prior seizure is considered acceptable)
5. Diagnostic and Statistical Manual of Mental Disorders, Fourth Edition – Text Revision (DSM IV-TR) criteria met (and not subsequently revised) for any of the following within specified period:
    - Major depressive disorder (current)
    - Schizophrenia (lifetime)
    - Other psychotic disorders, bipolar disorder (within the past 5 years), or substance (including alcohol) related disorders (within the past 2 years)
6. Metal implants in the head (except dental), pacemaker, cochlear implants, or any other non-removable items that are contraindications to MR imaging; MR compatible prosthetics, clips, stents, or any other device proven to be compatible will be allowed
7. Resides in hospital or moderate to high dependency continuous care facility (residence in low grade assisted living facility where there is sufficient autonomy to permit valid evaluation of activities of daily living is allowed so long as it is not mandated by an order issued either by the judicial or the administrative authorities)

8. History of swallowing difficulties (note: study drug should be swallowed whole and MUST NOT be broken, crushed, chewed, or dissolved in fluids prior to ingestion)
9. Pregnant or breastfeeding
10. Glucose-6-phosphate dehydrogenase (G6PD) deficiency
11. History of significant hematological abnormality or current acute or chronic clinically significant abnormality, including:
    - History of hereditary or acquired methemoglobinemia or baseline measurement of methemoglobin (MetHb) > 2.0% (confirmed on repeat)
    - History of hemoglobinopathy, myelodysplastic syndrome, hemolytic anemia, or splenectomy
    - Screening hemoglobin value (confirmed upon repeat) below age/sex appropriate lower limit of the central laboratory normal range

    Subjects in whom folate is < 4.0 ng/mL may be entered into the study provided folate supplementation (approximately 1 mg/day) is initiated and maintained for the duration of the study (see Section 4.7.8).

    Subjects in whom Vitamin B_{12} is < 150 pg/mL should be evaluated and supplemented as appropriate prior to the initiation of study drug (see Section 4.7.8). If review and correction can be achieved within the 42-day screening window, the subject may be entered into the study; otherwise the subject must be reconsented and rescreened after the deficit has been corrected.

12. Abnormal serum chemistry laboratory value at Screening deemed to be clinically relevant by the investigator, *e.g*., those considered to have the potential to increase the risk associated with study participation or administration of investigational product and, in the judgment of the investigator, would make the subject inappropriate for entry into this study. In addition, subjects with either of the following abnormalities must be excluded:
    - Creatinine clearance < 30 mL/min at Screening, estimated by the central laboratory according to the Cockcroft and Gault equation
    - Thyroid stimulating hormone (TSH) above laboratory normal range (subject may be treated and rescreened after 3 months)
13. Clinically significant cardiovascular disease or abnormal assessments (in the opinion of the investigator) such as:
    - Hospitalization for acute coronary syndrome (acute myocardial infarction or unstable angina) or symptoms consistent with angina pectoris, within the 12 months preceding Baseline
    - Signs or symptoms of clinical heart failure within the 12 months preceding Baseline
    - Evidence of uncontrolled atrial fibrillation on Screening ECG or history of atrial fibrillation that is not currently controlled (heart rate ≥ 85 bpm and/or inappropriate anticoagulation) or where the QT interval cannot in the opinion of the investigator be assessed by triplicate ECGs taken within an approximate 2- to 5-minute interval (if better control of the heart rate and/or of anticoagulation can

be achieved after adequate treatment, subject may be entered into the study if still within the 42-day window, or else the subject must be reconsented and rescreened). A cardiology consult should be sought for further ECG evaluation (especially in subjects with left bundle branch block) if deemed necessary by the investigator.

- QTcF (based on mean of three triplicate ECGs, QT corrected for heart rate using Fridericia's formula) at Screening > 460 msec in males or > 470 msec in females, or low or flat T waves making measurement of QT interval unreliable
- Recent history of poorly controlled hypertension, systolic blood pressure > 160 mmHg, or diastolic blood pressure > 100 mmHg, after 5 minutes in a seated position at Screening
- Hypotension: systolic blood pressure < 100 mmHg after 5 minutes in a seated position at Screening
- Heart rate < 48 bpm or > 96 bpm by measurement of vital signs (after 5 minutes in a seated position) or by ECG at Screening

14. Preexisting or current signs or symptoms of respiratory failure, *e.g.*, caused by chronic obstructive pulmonary disease, bronchial asthma, lung fibrosis, or other disease

    - Subjects with previously diagnosed moderate to severe sleep apnea not adequately controlled (in the opinion of the investigator) should be excluded

15. Concurrent acute or chronic clinically significant (in the opinion of the investigator) immunologic, hepatic (such as presence of encephalopathy or ascites), or endocrine disease (not adequately treated) and/or other unstable or major disease other than Alzheimer's disease

    - Subjects with hepatitis or primary biliary cirrhosis should be excluded
    - Human T-Cell Lymphocytic Virus Type III (HTLV-III), Lymphadenopathy Associated Virus (LAV), any mutants or derivatives of HLTV-III or LAV, any condition associated with Acquired Immunodeficiency Syndrome or similar condition however named

16. Diagnosis of cancer within the past 2 years prior to Baseline (other than basal cell or squamous cell skin cancer or Stage 1 prostate cancer) unless treatment has resulted in complete freedom from disease for at least 2 years

17. Prior intolerance or hypersensitivity to MT-containing drug, similar organic dyes, or any of the excipients

18. Treatment currently or within 3 months before Baseline with any of the following medications (unless otherwise noted; see Section 4.7.4):

    - Tacrine
    - Antipsychotics
        - o Clozapine, olanzapine (and there is no intent to initiate therapy during the course of the study)
        - o Other antipsychotics are allowable provided they have not been initiated within 3 months before Baseline and preferably at a stable dose and regimen

- Carbamazepine, primidone
- Drugs for which there is a warning or precaution in the labeling about methemoglobinemia at approved doses (*e.g.*, dapsone, local anesthetics such as benzocaine used chronically, primaquine and related antimalarials)

19. Current or prior participation in a clinical trial as follows:
    - Clinical trial of a product for cognition prior to Screening in which the last dose was received within 90 days prior to Screening unless confirmed to have been randomized to placebo
    - A clinical trial of a drug, biologic, device, or medical food in which the last dose was received within 28 days prior to Baseline

Subjects who fail screening may be rescreened if the inclusion or exclusion criterion that initially led to screening failure has changed and may render the subject potentially eligible; initial screening data and rescreening data will be captured in the database. The investigator should contact the medical monitor if there are any questions or concerns.

5.3. Discontinuation/Withdrawal

For a discussion of reasons for permanent discontinuation of study medication on the basis of safety, see Section 6.3. These include, but are not limited to, elevation in MetHb and/or clinically evident hemolytic anemia (Section 6.3.3.1), serotonin syndrome (Section 6.3.3.2), ARIA (Section 6.3.3.3), prolongation of the QT interval on ECG (Section 6.3.3.4), and decrease in renal function (Section 6.3.3.5).

Subjects may discontinue study drug and withdraw (drop out) from the study at any time for any reason. The caregiver may also withdraw his or her consent at any time for any reason. If a caregiver withdraws his or her consent, the subject must then also be withdrawn if alternative arrangements are not available (*e.g.*, an alternate caregiver). Furthermore, the investigator also has the right to discontinue trial medication if he or she judges that treatment is no longer appropriate, the subject's clinical condition is worsening, or for an adverse event.

If study drug is discontinued, the reason should be recorded as one of the following:

- AE
- Death
- Lack of efficacy (including progressive disease or worsening of cognitive capacity; in Germany, this includes loss of the ability to give consent)
- Lost to follow-up
- Withdrawal by subject or legal representative (or caregiver), including specific reasons, wherever possible
- Protocol deviation
- Non-compliance with study drug
- Pregnancy
- Study terminated by Sponsor

- Physician decision, including specific reason(s), wherever possible
- ARIA
- Other (specify)

If the reason for discontinuation of study drug is an AE, the principal event associated with discontinuation must be specified and recorded. In this case, reasonable effort must be made to clearly document the outcome. If the reason for premature discontinuation is an SAE, this must be documented and an SAE form must be completed (also see Sections 8.1.4 and 8.1.5).

For subjects who withdraw from the study for reasons other than death or subject or caregiver consent withdrawn, a visit should be scheduled as soon as possible after the last dose of study drug and identified end of treatment safety evaluations performed. For subjects who withdraw consent or when a caregiver withdraws consent without available alternate caregiver, the investigator should request that the reason be specified and the subject have any clinically indicated safety assessments performed.

Subjects in whom treatment is discontinued or who are lost to follow-up should be encouraged to return for a post-treatment visit approximately 28 days after the last dose of study drug and to continue with study visits until the scheduled end of participation for the subject. Subjects withdrawn from the study will not be replaced nor can they be re-enrolled (or enter the open-label extension trial).

5.4. Termination of the Study

The Sponsor reserves the right to terminate the study for duly justified reasons in accordance with the national laws (*e.g.*, in Germany: §40, section 1 of the Arzneimittelgesetz [AMG]). These reasons include in particular:

- Administrative reasons: *e.g.*, financial reasons
- Interest of subject welfare: *e.g.*, new information or events that result in an unfavorable risk-benefit profile.

6. TREATMENTS ADMINISTERED

Study drug tablets are formulated as blue film-coated oval tablets. All study drug tablets are visually indistinguishable from one another in order to preserve blinding. Two strengths of LMTM will be provided to allow for the randomized dose level and possible dose reduction. The contents of the three tablet strengths used in the study are:

- LMTM tablet containing 100 mg (expressed as MT base equivalent)
- LMTM tablet containing 75 mg (expressed as MT base equivalent)
- Placebo tablet containing LMTM 4 mg (MT base equivalent)

The active and inactive ingredients are described below.

6.1. Treatment

6.1.1. Active Ingredient

The active ingredient is methylthioninium (MT), provided as leuco-methylthioninium bis(hydromethanesulfonate) (LMTM, TRx0237). The chemical name is *N*,*N*,*N'*,*N'*-tetramethyl-10*H*-phenothiazine-3,7-diaminium bis(methanesulfonate).

Placebo tablets contain a low amount of LMTM, 4 mg, to prevent inadvertent unblinding that might otherwise occur because of a known effect of LMTM to cause urine and/or fecal coloration. The small total daily dose, 8 mg/day, is considered unlikely to have significant efficacy in Alzheimer's disease.

The potential for LMTM to cause urinary and/or fecal coloration (or coloration of other bodily fluids) should be explained to the subject and caregiver and will be described in the informed consent form (ICF). If a subject is known to have incontinence, such coloration could adversely affect compliance with study drug unless adequate precautions are taken (*e.g.*, use of incontinence pads). Even with the latter, discoloration in the context of incontinence may prove unacceptable to the subject/caregiver, and subjects should be entered into the study only after careful discussion of this possibility with them. Staining of underclothes and other fabrics is difficult to remove using standard washing products; therefore, subjects and caregivers should be informed about available techniques for washing stained clothing (see the Investigator's Brochure). The possibility of such staining should be clearly discussed with subjects and their caregivers.

6.1.2. Inactive Ingredients

Tablets also contain the following inactive compendial excipients: mannitol, crospovidone, microcrystalline cellulose, and magnesium stearate.

The film coat of study drug tablets contains polyvinyl alcohol-part hydrolyzed, talc, titanium dioxide, Macrogol PEG 3350, lecithin (soya), as well as non-compendial FD&C blue #2 (indigo carmine aluminum lake).

6.2. Study Regimens

Subjects will be randomly assigned to one of two treatment groups as described in Section 4.5: LMTM 200 mg/day or placebo. The planned daily study drug regimen, including the number of tablets and LMTM doses for each of the treatment groups, is summarized in Table 6-1. All LMTM doses cited below represent doses expressed as MT base equivalents. The study supplies are further described in Section 6.4.

Table 6-1 Planned Daily Study Drug Regimen: Tablets and LMTM Dose

Treatment Group	Tablet Contents (Amount of MT)	Number of Tablets			Dose of MT		
		AM	PM	Daily Total	AM	PM	Daily Total
LMTM 200 mg/day	100 mg	1	1	2	100 mg	100 mg	200 mg
Placebo	4 mg	1	1	2	4 mg	4 mg	8 mg

AM = morning; PM = evening

The first dose of study drug (one tablet) will be administered to subjects at the study site after Baseline assessments have been completed, eligibility has been confirmed, and randomization has been performed. Dosing will be under supervised conditions.

Subsequently, study drug tablets will be taken orally twice a day (one tablet each morning and one tablet each evening) on an outpatient basis. Morning and evening doses generally should be separated from one another by $\geq$ 10 hours (with the exception that the first evening dose may be taken after a shorter interval, depending on the timing of the first dose). Tablets may be taken with or without meals. Subjects will be instructed to take each dose of study medication with a full glass of water (see Section 6.5 for discussion of study drug dispensing).

Subjects and caregivers will be instructed that study drug should be swallowed whole and MUST NOT be broken, crushed, chewed, or dissolved in liquids prior to ingestion. If there are swallowing difficulties which prevent taking the medication as instructed, subjects should not be entered into the study. Subjects and caregivers should be warned that if the product is not swallowed immediately and is allowed to dissolve in the mouth, it will cause discoloration of teeth and oral mucosa.

6.3. Dose Interruption / Reduction

Interruption(s) of study drug administration and resumption of dosing at the same or at a reduced dose is allowed. The investigator should contact the medical monitor to discuss any interruptions and dose reductions. If after allowable interruptions, the subject does not tolerate re-introduction of the study drug or a clinically significant laboratory abnormality recurs, then study drug should be discontinued.

Dose interruptions and reductions will be implemented as described below. Guidance for managing selected adverse events / test abnormalities follows in Section 6.3.3.

All dose changes, including interruptions and decreases (and subsequent increases), should be recorded in the eCRF. The reason for each dose reduction and each dose interruption also should be recorded in the eCRF. The medical monitor should be informed of any dose decreases or interruptions.

6.3.1. Interruption

Interruption of study drug administration of up to (maximum duration on any one occasion) 30 days during the first 6 months of treatment and/or 14 days during the second 12 months of treatment may be allowed. More than one interruption is permitted during the study period. The investigator should contact the medical monitor to discuss any interruptions in study drug administration. An interruption should be avoided within 2 weeks before a scheduled visit. If a subject is receiving a reduced dose at the time an interruption starts, then when clinically indicated study drug should be resumed at that same reduced dose level and continued at the reduced dose until the next scheduled study visit, at which time the previous and/or original dose level can be resumed as clinically indicated.

If after allowable interruptions a subject does not tolerate study drug at the reduced (*e.g.*, Step-down 2) dose level (see Section 6.3.2), then study drug should be discontinued. However, every effort should be made to continue with the protocol assessments irrespective of the subject's further clinical management.

6.3.2. Dose Reduction

If a dose reduction is warranted, a step down in dose will be implemented by dispensing new study drug packages to the subject in a manner designed to maintain blinding (the subject will continue to take two tablets daily, but will take tablets from the newly dispensed packages). Until such time that the study visit can be scheduled, a subject may be advised by telephone to either interrupt dosing or to take one fewer tablet daily (with the tablet to be omitted clearly specified by the packaging).

Two dose reduction steps (“step-downs”) will be possible, designated as "Step-down 1" and “Step-down 2".

Step-down 1 will involve a substitution of a 100-mg tablet with a 75-mg tablet (for subjects randomized to LMTM 200 mg/day), thereby decreasing the total daily dose to 150 mg/day (or matching placebo).

Table 6-2 Reduced (Step-Down 1) Doses: Tablets and LMTM Dose

Original Treatment Group	Tablet Contents (amount of MT)	Number of Tablets			Dose of MT		
		AM	***PM***	**Daily Total**	***AM***	***PM***	**Daily Total**
LMTM 200 mg/day	75 mg	1	1	2	75 mg	75 mg	150 mg
Placebo	4 mg	1	1	2	4 mg	4 mg	8 mg

AM = morning; PM = evening

For Step-down 2, subjects will revert to the use of the original study drug packaging. They will be instructed to omit the morning dose (such that the resulting dose is either LMTM 100 mg/day or matching placebo 4 mg/day).

Table 6-3 Reduced (Step-Down 2) Doses: Tablets and LMTM Dose

Original Treatment Group	Tablet Contents (amount of MT)	Number of Tablets			Dose of MT		
		AM	***PM***	**Daily Total**	***AM***	***PM***	**Daily Total**
LMTM 200 mg/day	100 mg	← 1 →		1	-	100 mg	100 mg
Placebo	4 mg	← 1 →		1	-	4 mg	4 mg

AM = morning; PM = evening

For a subject whose dose has been decreased, the investigator subsequently should re-evaluate the subject and consider returning (increasing) the dose back to the previous dose level (*e.g.*, from the Step-down 1 level back to the originally randomized dose level, or from the Step-down 2 level back up to the Step-down 1 level), provided tolerability concerns have resolved. New study drug packages again will be provided in these circumstances. If the dose is increased back

to the previous and/or original dose level, subsequent dose decreases, as well as ensuing increases, in the same manner may be permitted over time if clinically indicated and after consultation with the medical monitor.

6.3.3. Guidance for Selected Adverse Events / Test Abnormalities

Guidelines for the monitoring and management of adverse events of special interest are given below. Clinical circumstances requiring additional evaluation of a subject and/or potential interruption or discontinuation of study drug are discussed.

6.3.3.1. Methemoglobinemia and/or Hemolytic Anemia

Any MetHb value recorded as greater than 3.5% should be confirmed by repeat measurement after 1 week. At any time, an elevated value (greater than 2.0%) should be immediately repeated and confirmed; the procedure for immediate repeat measurements is described in Section 8.4. In the event of elevation, the mean of three readings will be used as the basis for safety monitoring decisions. Dose interruptions and/or dose reductions (described in Sections 6.3.1 and 6.3.2) are allowable in order to manage the event.

Elevations in MetHb and/or clinically evident hemolytic anemia are to be managed as summarized in Table 6-4, including an unscheduled visit after 1 week. (See Section 4.7.8 for guidance on folate and Vitamin B_{12}.)

Table 6-4 Monitoring and Management of Elevated Methemoglobin

Methemoglobin	Monitoring	1-Week Follow-up Outcome	Action
>3.5% - < 5.0%	Unscheduled visit after 1 week	≤3.5%	Continue dosing
		Stable >3.5% - ≤4.5%	Continue with weekly monitoring
		Increased by > 1.0%	Interrupt dosing and continue with weekly monitoring until decreases to ≤3.5%[1]; resume dosing at reduced dose (see Section 6.3.2)
≥5.0%	Discontinue dosing permanently, and continue monitoring until decreases to≤3.5%		

[1] If dosing is held for more than 30 consecutive days during the first 6 months of study or 14 consecutive days during the second 12 months of study, subject should be discontinued (see Section 5.3)

For symptomatic methemoglobinemia or when there are signs and/or symptoms of hemolytic anemia, dosing should be discontinued. In addition to the action taken with the dose, hematology testing (RBC panel as described in Section 8.3.2) should be performed as well as measurement of LDH and total, direct and indirect bilirubin. Haptoglobin values should be obtained within 1 week. If abnormal, repeated measurements should continue to be obtained until normal values are noted or stabilization has occurred (at a level acceptable to the investigator). It is acceptable to perform such repeat testing at a local laboratory provided the results are provided to the site (and recorded in the eCRF). As described in Section 8.3.2, determination of Heinz bodies may also be considered; such testing is to be performed at the local laboratory. If Heinz bodies are present, study medication must be stopped, but hematological indices should be monitored until

this resolves. The medical monitor should be contacted to discuss whether or not resumption of dosing is indicated or for any other questions.

In addition to elevated MetHb, the signs and symptoms of methemoglobinemia or anemia include:

- Cyanosis, headache, anxiety, exercise intolerance, fatigue, confusion, dizziness, tachypnea, palpitation, dysrhythmia, seizures, and coma

Signs of possible hemolytic anemia include:

- Decrease by 20% from Screening in RBC count and/or hemoglobin
- Abnormal RBCs in peripheral blood smear
- Elevation of reticulocyte count to above laboratory normal range
- Increase in LDH or indirect bilirubin, or low haptoglobin

6.3.3.2. Serotonin Syndrome

Subjects are to be monitored for signs and symptoms indicative of potential serotonin toxicity. All subjects should remain in the clinic for at least 4 hours after the first dose of study drug. While in the clinic, subjects should be evaluated by a medically qualified person by targeted physical and neurological examination and measurement of oral (sublingual) temperature, respiratory rate, blood pressure, and pulse. The Serotonin Toxicity (Syndrome) Diagnostic Interview and Rating Guide provides further guidance (see Section 24.2). An assessment is to be completed just prior to discharge from the clinic. If a subject meets any one of four possible criteria for serotonin syndrome, no further study drug should be administered and the subject managed as medically appropriate. Subjects without significant signs and symptoms may be discharged from the study unit.

For subjects entering the study on a serotonergic drug (see Section 4.7.2), subjects and their caregivers should remain in close proximity to a local hospital and remain in telephone contact with the clinic for at least 12 to 14 hours after the first dose of study drug. Reimbursement for accommodations will be made available if requested by the subjects and/or their caregivers. They shall be provided with monitoring instructions, a diary, and an oral thermometer at the time of Day 1 discharge from the clinic and instructed in its use. Temperature should be monitored three times a day (in the morning, afternoon, and evening) until 72 hours after the first dose and will be recorded in the diary to be returned at Visit 3 (Week 2). Should any changes be noted, they should call the clinic and return to the clinic if so instructed or seek urgent medical care. In addition, caregivers are to be contacted 5–7, >7–14, >14–24, 44–52 and 68–76 hours after the first dose of study drug. The contacts should be at least 1 hour apart. If indicated, more frequent contacts will be made, and the site will assume responsibility for clinical review and hospital referral. Instructions for the assessment are provided in the Serotonin Toxicity Telephone Assessment in Section 24.3.

The same procedures apply to any subject newly starting treatment with a serotonergic drug.

Table 6-5 Overview of Monitoring and Management of Serotonin Toxicity (Syndrome)

Signs and Symptoms of Serotonin Toxicity[1]	Monitoring after First Dose and at Each Clinic Visit	Action
**Any one of the following:**<br>Autonomic findings (temperature ≥38 °C, diaphoresis, shivering, tachypnea/dyspnea, diarrhea, hypertension or hypotension)<br>Cognitive changes (agitation/akathisia, elevated mood, insomnia)<br>Neuromuscular changes (tremor; hyperreflexia; spontaneous, inducible, or ocular clonus; muscle rigidity[2]; hypertonia; dizziness; incoordination; or mydriasis)	Targeted PE and neurological examination, focused on the following:<br>deep-tendon reflexes, clonus, and muscle rigidity; size and reactivity of pupils; dryness of oral mucosa; intensity of bowel sounds; skin color; and presence or absence of diaphoresis	Discontinue study drug<br>Appropriate medical management to be undertaken
	Oral (sublingual) temperature	
	Seated blood pressure and pulse	
	MMSE (Screening, Weeks 26, 52, 78, and at the 4-week follow-up visit and as needed to respond to changes)	

[1] See Section 24.2 for an interview and monitoring guide and rating scale
[2] Muscle rigidity can overwhelm other neuromuscular findings and mask the diagnosis

6.3.3.3. ARIA

Brain MRI will be performed at Screening/Baseline and at Weeks 13, 26, 39, 52, 65, and 78 (±14 days) or early termination to monitor for ARIA, including vasogenic edema, macrohemorrhages, microhemorrhages, or an area of superficial siderosis (see Section 9.1). Evaluation of images will be performed centrally by an independent neuroradiologist who is not involved in the clinical trial. Information about presence or absence of ARIA will be communicated to the sites by the imaging core laboratory within 5 business days of image transfer (and resolution of any imaging queries).

The study drug should be permanently discontinued if an imaging abnormality consistent with any of the following occurs:

- vasogenic edema, macrohemorrhage
- an area of superficial siderosis
- clinically symptomatic microhemorrhage(s)
- >4 clinically asymptomatic new microhemorrhages.

The subject and caregiver are to be informed of the reason (informing other sites is discussed in Section 8.1.3). The following signs and symptoms have been reported in trial subjects found to have ARIA: confusion (with or without hallucination) or acute decline in cognition, headache, gait disturbance/ataxia, vomiting or upper gastrointestinal disturbance, and acute development of focal neurological signs. Consideration should be given to treatment with high-dose dexamethasone should symptoms be severe. If ARIA occurs, subjects must be re-scanned every 6 weeks after the discontinuation of the study drug in order to evaluate their stability until the imaging abnormalities are resolved or stabilized (the latter to be determined based on three follow-up scans). Subjects with new microhemorrhage such that there are > 4 must also be discontinued (even if clinically asymptomatic) and be re-scanned every 6 weeks.

The following table provides a summary of the appropriate imaging follow-up to be performed in response to ARIA findings.

Table 6-6 Overview of ARIA Findings Management Plan

Finding Type	Symptomatic?	Action with Study Drug	Recommended Post-Discontinuation Follow Up
Vasogenic edema	Yes or No	Discontinue study drug	Scan subject every 6 weeks after discontinuation of study drug to evaluate resolution/stabilization (stabilization is to be determined based on three follow-up scans).
Macrohemorrhage (≥10 mm)	Yes or No		
Superficial siderosis	Yes or No		
New microhemorrage > 4 (<10 mm)	Yes or No		
New microhemorrhage ≤ 4 (<10 mm)	Yes		
	No	Subject may stay on study until next safety assessment	Not applicable

Source: TRx-237-005 Imaging Charter (BioClinica)

6.3.3.4. QT Interval and ECG Abnormalities

On Day 1 (Baseline), the pre-dose machine-read ECGs should be reviewed to confirm that there are no clinically significant abnormalities (see Exclusion Criterion No. 13) or deviations from the Screening ECG; the average of the three readings should be used for determination of QTcF and heart rate. Dosing may be held subject to eligibility review based on the local interpretation of triplicate ECGs by the investigator. A cardiology consult should be sought prior to dosing if deemed necessary by the investigator.

Subsequent decisions with respect to the ECG may be made on the basis of the local reading, with cardiology consult if deemed appropriate by the investigator. In the event of concern based on the local interpretation, the ECG should be repeated, preferably in triplicate, and doses interrupted if warranted, pending the central read or local cardiology consult.

For subjects entering the study with controlled atrial fibrillation (*i.e.*, heart rate ≤ 84 bpm and appropriate anticoagulation), triplicate ECGs are mandatory at all visits after Visit 2. Data documented on the eCRF will be the average of the three readings. As ECG readings are done locally, a local cardiologist should be consulted for appropriate evaluation of ECGs if deemed necessary by the investigator.

For subjects entering the study with intraventricular conduction defects, a local cardiologist should be consulted for appropriate evaluation of the ECGs. In subjects with left bundle branch block, a cardiologist’s evaluation is strongly recommended.

Treatment should be discontinued if the following treatment-emergent change occurs (on Day 1, based on the means of three pre- and post-dose recordings and single recordings at other time points confirmed by triplicate ECGs in the event of, in the opinion of the investigator, clinically significant abnormality) without other explanatory cause:

- QTcF interval >500 msec: repeat ECGs should be obtained, in triplicate, within 2 weeks; if confirmed by the mean of the repeat ECGs, study drug should be discontinued pending further evaluation.

Other clinically significant changes in the ECG should be discussed with the medical monitor.

6.3.3.5. Other Safety Reasons Requiring Discontinuation

If renal concerns arise and the calculated creatinine clearance is < 30 mL/min, study drug should be discontinued.

6.4. Packaging, Labeling, and Storage

Double-blind study drug will be packaged, labeled, and distributed to study sites by a designated vendor.

Study drug supplied to subjects will be in individual aluminum blister cards containing 14 tablets. An appropriate number of blister cards will be contained within cardboard cartons with sufficient supplies until the next scheduled study visit at which dispensing is planned (see Section 6.5). Additional 14-tablet blister cards will be included in cartons as needed to allow for delays in visit scheduling (or if original medication is lost or damaged).

Study drug package labels will be in compliance with applicable regulatory requirements and will include the statement "Keep out of reach of children", the cautionary statement "Caution: New Drug – Limited by Federal (United States) law to investigational use" and/or "For clinical trial use only" as appropriate, as well as any other locally mandated statements. Labels will also be translated into the local language.

At a minimum, labels will also include the following information: the name and address of the Sponsor, the study code, a unique identifier, and appropriate contact information. In those jurisdictions where required, a re-test or expiry date will be included.

At the study site, study drug must be stored securely (*e.g.*, locked area, pharmacy) and at a temperature not more than 30°C. The temperature at which study drug is stored at the study site will be recorded daily using a centralized temperature monitoring system if this is available. If not, study drug storage temperature will be recorded each working day using a maximum-minimum thermometer. The packaging protects the study drug from light and moisture.

Subjects and caregivers should also be provided with information about required storage conditions. Study drug should be ingested immediately after removal from the blister card.

6.5. Dispensing

With the exception of the first dose administered in the clinic, all dosing will be on an outpatient basis. Study drug will be first dispensed to subjects/caregivers on Day 1 (Baseline, Visit 2) if it is determined that the subject can be discharged from the clinic; enough study drug will be dispensed to last the subject until Visit 4. This will occur after subjects have been observed at the site for at least 4 hours post-dose.

Thereafter, beginning at the Week 6 visit (Visit 4), study drug will be dispensed at each subsequent study visit during the treatment period, except for Week 78 (Visit 10) or the early termination visit, after which administration of study drug under this protocol will cease. Subjects should bring all study medication, including the overage blister card(s), with them to every study visit for compliance assessment.

Subjects and caregivers will be provided with information about storage conditions and taking study drug, including instructions indicating that study drug must be used only as described in this protocol. They will also be informed that tablets should be swallowed whole and not broken,

crushed, chewed, or dissolved in fluids prior to ingestion. If there are swallowing difficulties which prevent taking the medication as instructed, subjects should not be entered into the study. Subjects and caregivers should be warned that if the product is not swallowed immediately and is allowed to dissolve in the mouth, it will cause discoloration of teeth and oral mucosa. In the event of interruption or dose reduction, the subjects and caregivers will be provided with updated dosing instructions.

6.6. Compliance

At each post-Baseline visit during the treatment period, the number of tablets dispensed to the subject/caregiver will be recorded.

At the Week 2 visit (Visit 3) and at each study visit thereafter during the treatment period, the subject/caregiver will bring all unused study drug (provided at the previous study visit or, in the case of the Week 6 visit [Visit 4], provided at Baseline [Visit 2]) to the study site. The number of tablets (all tablets remaining in unopened blister card plus any tablets that have been removed from blister card) will be counted and recorded by study site staff. Empty blister cards and packaging should also be returned by the subject/caregiver to the study site.

Subject compliance with prescribed study drug will also be assessed at each visit by questioning the subject and caregiver. Any apparent discrepancies between the number of tablets taken and the number of tablets which should have been taken since the last visit will be discussed with the subject and caregiver.

Compliance data, including dates of any dose deviations and/or interruptions, and any other pertinent information, will be recorded in the source documentation and on the appropriate field of the eCRF.

If during participation in the study a subject's compliance is determined to be < 80% or > 120% (taking into consideration allowable reductions in the number of tablets taken or dose interruptions), the subject and caregiver should be reeducated about taking study drug properly and the clinical research associate should be informed promptly. If compliance problems are recurrent, the investigator should inform the clinical research associate and contact the medical monitor to determine the course of action.

6.7. Accountability

The investigator or designee will keep a record of all study drug received, and of all study drug dispensed to and returned by subjects.

The investigator will ensure that the supplied study drug will be used only for administration to subjects enrolled in this study and for no other purpose.

The study drug accountability record will be checked by a study monitor at monitoring visits.

All unused and returned study drug will be either returned to the Sponsor or designee or disposed of after study completion according to provided instructions.

7. ASSESSMENT OF EFFICACY

Efficacy will be measured at Screening/Baseline and at scheduled visits during the treatment period and follow-up visit (see Table 4-1 in Section 4.4). Baseline efficacy assessments may be made on the day before randomization and dosing if necessary.

7.1. Raters

Efficacy instruments will be completed by assessors/raters who are not involved in the assessment of safety parameters that could result in unblinding, generally in the local language. Information regarding validation in each geographic region/language is on file with the Sponsor. Each time an efficacy instrument is administered, the name of the person administering the instrument and the date and time of administration will be recorded. For a given subject, raters should remain constant throughout the study if possible. Investigators and other raters will be trained and their proficiency in administering applicable efficacy instruments will be evaluated and documented. If there is to be a change in rater, the new rater cannot proceed until his or her proficiency in administering applicable efficacy instruments has been evaluated and documented. It is important that every effort be made to keep Rater 2 (who administers the ADCS-CGIC) constant throughout the study. If a change is to be made, a per-subject handover must be arranged by way of at least one joint Rater 2 assessment involving both the new and the old Rater 2.

At the screening visit, the CDR total should be performed by the most experienced clinician available (*e.g.*, principal investigator or appropriately experienced designee) in order to make the eligibility determination.

The following are additional rater requirements with respect to specific efficacy and pharmacoeconomic instruments:

- For the ADAS-cog_{11} and ADCS-ADL_{23}, the rater does not need to be blinded to other efficacy results. This rater will be termed Rater 1. This rater will also rate the NPI, MADRS, RUD Lite (see Section 9.2), and will be permitted to rate the C-SSRS and Serotonin Toxicity Assessment.
- The ADCS-CGIC will be performed by an experienced qualified rater who remains blinded throughout the study to results from the ADAS-cog_{11}, ADCS-ADL_{23} and all other efficacy results, as well as to all safety assessment results. The ADCS-CGIC rater therefore will be a separate rater, termed Rater 2, who does not perform any other efficacy or safety ratings. The ADCS-CGIC rater may, however, assess CDR, which is applied only as a screening assessment.

The screening CDR and MMSE questionnaires will be assessed by an independent evaluator at Worldwide Clinical Trials (WCT). The screening assessments will be reviewed by WCT to ensure that subjects meet the inclusion criteria. As necessary, adjudication will occur for any subject where assessments fall outside of the inclusion criteria.

Results of efficacy assessments will be recorded in source documents in a manner such that required assessor blinding is maintained.

Each time an efficacy instrument is administered, the name of the person administering the instrument and the date and time of administration will be recorded. Efficacy assessments should

be performed at approximately the same time of day throughout the study for a given subject, if possible. The sequence of subject and caregiver assessments is given in Table 7-1.

Table 7-1 Overview of Efficacy Assessments: Sequence of Testing by Rater

	Sequence	Subject		Caregiver	
		**Rater 1**	**Rater 2**	**Rater 1**	**Rater 2**
Screening	1	MMSE[5] (~20 minutes)	-	-	-
	2	-	-	CDR total[1] (~30-40 minutes)	-
	3	CDR total[1] (~30-40 minutes)	-	-	-
Baseline and Weeks 13, 26, 39, 52, 65, 78[2] and the 4-week off-treatment follow-up visit, unless otherwise indicated[3]	1	ADAS-cog$_{11}$ (~30-40 minutes)	-	ADCS-ADL$_{23}$ (~30 minutes)	-
	2	-	-	-	ADCS-CGIC (~30 minutes)
	3	MADRS (~30-40 minutes)[3]	ADCS-CGIC (~30 minutes)	-	-
	4	-	-	NPI (~20-30 minutes)[3]	-
Weeks 26, 52, and 78[2] and the 4-week off-treatment follow-up visit	5	MMSE[5] (~20 minutes)	-	-	-
Baseline and Weeks 26, 52, and 78[2]	6	-	-	RUD Lite (~20 minutes)[4]	-

[1] Most experienced rater (*e.g.*, principal investigator or designee), shown here as Rater 1, but may be Rater 2 or Adverse Events Reviewer
[2] Or early termination
[3] Baseline and Weeks 26, 52, and 78 (or upon early termination only)
[4] Discussed in Section 9.2
[5] Shown here as Rater 1, but may be Adverse Events Reviewer

7.2. Instruments

The main efficacy instruments include the following: ADAS-cog$_{11}$ (cognition), ADCS-ADL$_{23}$ (activities of daily living), and ADCS-CGIC (global impression of change). Other aspects of Alzheimer's disease will be rated with the NPI (behavioral symptoms), MADRS (mood), and MMSE (severity staging). All are described briefly below. The CDR, used to establish eligibility, is also described. The RUD Lite (resource utilization) is discussed in Section 9.2.

7.2.1. Alzheimer's Disease Assessment Scale – Cognitive Subscale (ADAS-cog$_{11}$)

The ADAS was designed to evaluate the severity of cognitive and non-cognitive or behavioral symptoms of Alzheimer's disease (Rosen *et al.*, 1984). The ADAS-cog is the cognitive subscale of the ADAS.

The ADAS-cog_{11} consists of 11 tasks that measure memory, orientation, language, and praxis. The total score range is 0-70, with higher scores indicating greater impairment.

The ADAS-cog has been used widely as the primary measure of cognitive change in contemporary studies of Alzheimer's disease. The ADAS-cog has been shown to have good test-retest and inter-rater reliability. The instrument is sensitive to longitudinal change, with scores having been shown to deteriorate by approximately 8-9 points per annum in community patients with Alzheimer's disease (Stern *et al*., 1994) and by approximately 4-6 points in typical Alzheimer's disease study populations (Schneider, 2001).

The ADAS-cog has been shown to have a sufficiently low signal-to-noise ratio to allow for detection of small benefits associated with treatment with AChEIs (Birks and Harvey, 2004).

The ADAS-cog_{11} will be performed at Baseline (Visit 2, pre-dose) and at Weeks 13, 26, 39, 52, 65, and 78 (or early termination) visits. The scale will be repeated at the 4-week off-treatment follow-up visit (Visit 11).

7.2.2. Alzheimer's Disease Cooperative Study – Activities of Daily Living (ADCS-ADL_{23})

The ADCS-ADL_{23} includes 23 items that were derived from a larger set of items describing performance of activities of daily living (ADL) by Alzheimer's disease patients (Galasko *et al*., 2005).

Of 45 ADL items originally evaluated during the creation of this instrument, 27 showed good test-retest reliability over 1-2 months (Galasko *et al*., 1997; Galasko *et al*., 2005). Scores of these items also were shown to correlate with MMSE scores of Alzheimer's disease patients and to decline over 12 months in at least 20% of Alzheimer's disease patients.

The ADCS-ADL_{23} will be performed at Baseline (Visit 2, pre-dose) and at Weeks 13, 26, 39, 52, 65, and 78 (or early termination) visits. The scale will be repeated at the 4-week off-treatment follow-up visit (Visit 11).

7.2.3. Alzheimer's Disease Cooperative Study – Clinical Global Impression of Change (ADCS-CGIC)

The ADCS-CGIC, which was originally designed on the basis of a survey of clinicians and by adapting existing instruments, allows a clinician to rate clinically relevant global changes using an organized but unstructured format. The ADCS-CGIC has been shown to have good reliability, validity, and change sensitivity (Schneider *et al*., 1997), and has been shown to be sensitive to the effects of AChEIs on efficacy in Alzheimer's disease (Rogers *et al*., 1998).

At Baseline, a clinician blinded to other aspects of the study rates 15 areas in three domains (mental and cognitive state; behavior; and social and daily functioning) based on an interview with the subject and caregiver, to be documented in notes in sufficient detail to serve as reference for post-Baseline assessments.

This process is repeated at time points subsequent to initiation of treatment and a change rating is determined using a 7-point Likert scale. The change is to be referred only to Baseline and not the rest of the visits. That is, the only change that is scored at any given visit is the change from baseline, and not from any other preceding visit.

In order to ensure that change score is based on the comparison between the Baseline Visit and the current visit, the Rater 2 will only have access to the Baseline assessment notes for the purpose of rating subsequent change. Rater 2 will not have access to notes from any intermediate visits other than the Baseline visit at any subsequent assessment. It is therefore particularly important that every effort be made for the Rater 2 to remain constant throughout the study. If a change is to be made, a per-subject handover must be arranged by way of at least one joint Rater 2 assessment involving both the new and the old Rater 2.

The change is to be scored as:

1 = marked improvement
2 = moderate improvement
3 = minimal improvement
4 = no change
5 = minimal worsening
6 = moderate worsening
7 = marked worsening

The ADCS-CGIC will be performed at Baseline (Visit 2, pre-dose) and at Weeks 13, 26, 39, 52, 65, and 78 (or early termination) visits. The scale will be repeated at the 4-week off-treatment follow-up visit (Visit 11). At each time point, the caregiver is evaluated first, and then the subject evaluation is performed.

7.2.4. Neuropsychiatric Inventory (NPI)

The NPI assesses behavioral disturbances in dementia. A screening strategy is designed to save time (Cummings *et al*., 1994). The NPI is reliable and valid (Cummings, 1997) and has been reported to be sensitive to the effects of tacrine (Kaufer *et al*., 1996) and metrifonate (Morris *et al*., 1998).

The NPI measures the following behavioral areas: delusions, hallucinations, agitation, depression, anxiety, euphoria, apathy, disinhibition, irritability, aberrant motor behavior, night time behaviors, and appetite and eating disorders.

Symptoms in a particular domain are rated in terms of frequency (0-4) and severity (0-3). Score range is 0-12 for each domain and 0-144 for the total score, where higher scores indicate higher behavioral psychopathology.

The NPI will be completed at Baseline (Visit 2, pre-dose) and at Weeks 26, 52, and 78 (or early termination) visits.

7.2.5. Montgomery-Asberg Depression Rating Scale (MADRS)

The MADRS is a clinician-rated assessment of the subject's level of depression (Montgomery and Asberg, 1979). The measure contains 10 items that measure apparent and reported sadness, inner tension, reduced sleep and appetite, difficulty concentrating, lassitude, inability to feel, and pessimistic and suicidal thoughts. Each item is scored in a range of 0 to 6 points, with higher scores indicating increased depressive symptoms; the MADRS total score ranges from 0 to 60.

The MADRS will be completed at Baseline (Visit 2, pre-dose) and at Weeks 26, 52, and 78 (or early termination) visits.

7.2.6. Clinical Dementia Rating (CDR)

The CDR rates overall severity of dementia (Hughes *et al*., 1982). The CDR has been shown to have good reliability (Morris *et al*., 1997) and validity (Fillenbaum *et al*., 1996) and to correlate with neuropathological status (Morris *et al*., 1988). The scale is sensitive to change over longer time periods (Berg *et al*., 1988).

The CDR is administered using a worksheet and semi-structured interview of the subject and a reliable informant (*e.g*., caregiver) to assess the following 6 domains: memory, orientation, judgment and problem solving, community affairs, home and hobbies, and personal care. Each domain is scored on a 5-point scale, where 0 = normal, 0.5 = questionable impairment, 1 = mild impairment, 2 = moderate impairment, and 3 = severe impairment.

The CDR total score (also sometimes referred to as the CDR global score) is derived from individual (“box”) scores for each of the 6 domains, in accordance with published scoring rules.

The CDR will be performed at the Screening visit to determine a CDR total score for severity/inclusion determination.

7.2.7. Mini-Mental State Examination (MMSE)

The MMSE (Folstein *et al*., 1975) was originally developed to differentiate between psychiatric patients with functional and organic conditions, to quantify the level of cognitive impairment, and to monitor changes over time. The MMSE subsequently has become a widely used and extensively validated cognitive test demonstrating satisfactory reliability, validity, and change sensitivity under a wide variety of conditions (Tombaugh and McIntyre, 1992).

Over a 12-month period, Alzheimer’s disease patients might typically show a decline in MMSE score of 2-4 points (Schneider, 2001).

The utility of the MMSE as a means of assessing treatment response in Alzheimer’s disease has been questioned (Bowie *et al*., 1999), but its status as a clinical outcome measure has been supported by the UK National Institute for Clinical Excellence guidance (NICE, 2001). Furthermore, the MMSE has demonstrated an ability to detect change in clinical studies with AChEIs (Birks and Harvey, 2004).

In an epidemiological study (Mukaetova-Ladinska *et al*., 2000), pre-mortem MMSE scores have been correlated with post-mortem Braak stage (based on the spread of tau pathology through the brain).

The MMSE (a modified version of the form supplied by Psychological Assessment Resources) will be performed at the Screening visit and at Weeks 26, 52, and 78 (or early termination) visits and at the 4-week off-treatment follow-up visit (Visit 11). The MMSE in the current study will be used as a measure of inclusion eligibility, baseline staging, and as a secondary measure of cognitive outcome.

8. ASSESSMENT OF SAFETY

Safety will be assessed over time by means of adverse events (AEs), clinical laboratory tests of blood (*e.g.*, hematology and chemistry panels, Vitamin B_{12} levels, folate levels and TSH) and urine (*e.g.*, urinalysis), vital sign measurements, MetHb and oxygen saturation measurements, ECGs, brain MRI, physical and neurological examinations, serotonin toxicity assessments, and assessment of the potential for suicide or self-harm.

Assessments to be performed in the clinic are described in the subsections that follow. At intervening times (at 9, 19, 32, 45, 58, and 71 weeks ± 7 days), caregivers of subjects are to be contacted by telephone and an unscheduled visit is to take place if needed in response to a safety concern. Similarly, an unscheduled visit may become necessary (with in-clinic monitoring) if a drug with serotonergic potential is to be initiated (see Section 4.7.2). Imaging-based safety assessments are discussed in Section 9.1.

Over the course of the entire study (including the post-treatment follow-up visit), the total volume of blood collected from each subject will not exceed approximately 260 mL.

8.1. Adverse Events

An AE is any unfavorable or unintended sign, symptom, or disease, whether or not considered related to the study treatment. This also includes events resulting from medication error or inappropriate use. AE recording will begin at the time the ICF is signed. Thereafter, AEs will be ascertained by asking the subject (and caregiver) how the subject has been since the last visit. A clinical abnormality, laboratory test value abnormality, or imaging abnormality that the investigator deems to be clinically significant should be recorded as an AE.

Every attempt should be made to describe the AE in terms of a diagnosis. Once a clear diagnosis has been made, individual signs and symptoms shall not be recorded unless they represent atypical or extreme manifestations of the diagnosis, in which case they should be reported as separate events. Events leading up to a diagnosis should be retained. If a clear diagnosis cannot be established, each sign and symptom must be recorded individually. Consistent with this, signs or symptoms indicated by the Serotonin Toxicity (Syndrome) Diagnostic Interview and Rating Guide (In Clinic) (Section 24.2) or Serotonin Toxicity Telephone Assessment (Section 24.3) that are judged to represent an AE should be recorded as such. However, if any of the criteria for serotonin syndrome are met (see Serotonin Syndrome Worksheet in Section 24.2), the AE is to be recorded as "Serotonin Syndrome", rather than the individual terms.

All AEs must be fully recorded in the source documents and in the eCRF, regardless of whether or not the event is considered related to study drug.

For all AEs, the investigator must pursue and obtain information adequate both to determine the outcome of the AE and to assess whether it meets the criteria for classification as an AE of special interest (Section 8.1.3) or as a Serious Adverse Event (SAE) or pregnancy requiring immediate notification (see Sections 8.1.4 and 8.1.5).

Follow-up of an AE, even after the final dose of study drug, is required if the AE or its sequelae persist. Follow-up is required, including beyond the scheduled final off-treatment visit if needed, until the AE or its sequelae resolve or stabilize at a level acceptable to the investigator.

For each AE, information recorded will include the following: the date when the AE started, the date when the AE stopped (or whether it remained ongoing), the intensity of the AE (see

Section 8.1.1), the relationship of the AE to study drug (see Section 8.1.2), action taken with regard to study drug (none, dose reduction, interrupted, or discontinued), other drug therapy (no change, new medication, altered medication, or both of the latter), outcome, and whether or not the AE was considered an SAE (see Section 8.1.4). On Day 1, a separate AE form will be used, and will include time of dose and time of AE onset.

8.1.1. Intensity

The intensity (severity) of each AE will be assessed by the investigator and graded as mild, moderate, or severe, as follows:

- Mild: An AE that is easily tolerated by the subject, causes minimal discomfort and does not interfere with everyday activities
- Moderate: An AE that is sufficiently discomforting to interfere with normal everyday activities
- Severe: An AE that prevents normal everyday activities

8.1.2. Relationship to Study Drug

The investigator will make a judgment considering whether or not, in his or her opinion, each AE is related to the study drug according to classifications described here. However, even if the investigator feels that there is no relationship to the study drug, the AE should be reported nevertheless. For each AE, the relationship or association (causality) of the AE to study drug will be assessed by the investigator and characterized as not related, unlikely related, possibly related, or related as follows:

- Not related: If there is a confirmed cause of the AE (other medical condition, other therapy) which does not involve the study drug
- Unlikely related: If the temporal association between the AE and the study drug is such that the AE is not likely to be related to the study drug
- Possibly related: If the AE shows a reasonable temporal association to study drug administration but could be due to the subject’s clinical state or other therapies administered
- Related: If the AE shows a reasonable temporal association to study drug administration and cannot be explained by the known characteristics of the subject’s clinical state

8.1.3. Adverse Events of Special Interest

Several adverse events are of special interest because additional steps are to be taken by the investigator to assess and manage them. The medical monitor shall be informed (either directly by the laboratory, the core imaging laboratory or the site, depending on the nature of the event). AESIs include:

- Methemoglobin values > 3.5% (confirmed on repeat result; see Section 8.4), signs or symptoms consistent with methemoglobinemia or hemolytic anemia, or observation of Heinz bodies. For purposes of this study, only post-randomization methemoglobin values meeting these criteria would be considered an AESI and whether or not this is considered an SAE depends on medical and scientific judgment.

- A case meeting any one of the four criteria for serotonin syndrome outlined in Section 24.2 is a medically significant event and thus will be reported and handled as an SAE (see Section 8.1.6)
- Any possible case of ARIA (see Section 6.3.3.3 and Section 9.1.5.2) is a medically significant event and thus will be reported to the Sponsor and the procedure will be as for an SAE (see Section 8.1.6); asymptomatic subjects with ≤ 4 new microhemorrhages need not be handled as an SAE. All investigators should be notified of the possible occurrence of ARIA, its imaging manifestations, and the clinical signs and symptoms that may accompany this phenomenon. They should also be told of the measures to be taken should ARIA occur.

See Section 6.3.3 for additional details regarding assessment and dose interruption, reduction, or discontinuation in response to any of these AESIs.

Each AESI should be recorded as for any other adverse event.

8.1.4. Serious Adverse Events

An SAE is defined as any event that:

- Results in death (including suicide)
- Is life-threatening
- Results in inpatient hospitalization or prolongation of existing inpatient hospitalization
    - Planned admissions for respite care are not to be considered an SAE (the medical monitor should be contacted for confirmation regarding whether or not an admission for respite care should be considered planned or unplanned). Unplanned admissions for respite care will constitute an SAE unless it is as a result of caregiver needs that are independent of the subject's condition.
    - An admission or prolongation of existing hospitalization because the subject does not want to be discharged, or because the caregiver is unable or unwilling to care for the subject, is not to be considered an SAE.
    - Admissions to a hospital that were planned or anticipated before the start of the study for an unrelated pre-existing medical condition are not to be considered an SAE.
- Results in persistent or significant disability or incapacity
- Is a congenital anomaly or birth defect
- Other emergent medically significant events that require immediate medical or surgical intervention. For purposes of this study, the following will be considered medically significant events and thus reported to the Sponsor and the procedure will be as for SAEs whether or not they meet any other criteria for being an SAE:
    - Any post-randomization case of suicidal ideation, intent, or action or self-injurious behavior (see Section 8.9)
    - Possible post-randomization cases of serotonin toxicity and ARIA (see Section 8.1.3)

Medical and scientific judgment should be exercised in deciding whether an event is serious and whether expedited reporting is appropriate in other situations, such as important medical events that may not be immediately life-threatening or result in death or hospitalization but may jeopardize the subject or may require intervention to prevent one of the outcomes listed in the definitions above.

All SAEs must be reported on the eCRF. An assessment should be made by the investigator of whether the event is study drug-related, *i.e.*, is ‘causally’ related to the study drug.

8.1.5. Pregnancy

Pregnancy is to be considered an immediately reportable event. This includes pregnancy of a female subject or a female sexual partner of a male subject.

Subjects who become pregnant during the clinical study should discontinue study drug immediately and contact the investigator.

Subjects should be instructed to notify the investigator of a pregnancy either during the treatment period of the study or within 3 months after the last dose of study drug. Whenever possible, a pregnancy should be followed to term, any premature terminations reported, and the status of the mother and child should be reported to the Sponsor after delivery.

Although the pregnancy is not considered to be an AE or SAE, any pregnancy complications should be recorded as AEs or SAEs (if applicable). Any pregnancy should be followed through delivery for the observation of any SAE. Therefore, regardless of whether or not a pregnancy is actually considered an SAE, a pregnancy form should be completed for all pregnancies. Pregnancies should initially be reported in the Pregnancy Notification Form, Part I and sent by e-mail to the following address:

drugsafety@wwctrials.com.

When the outcome of the pregnancy is known, site personnel will complete the Pregnancy Notification Form, Part II and e-mail it to the following address:

drugsafety@wwctrials.com.

All data related to pregnancy, pregnancy outcome, and SAE associated with pregnancy will be recorded in a safety database maintained by personnel responsible for pharmacovigilance at Worldwide Clinical Trials.

8.1.6. Reporting Requirements and Timeframes

The requirements for reporting SAEs and pregnancies are described below. A Safety Management Plan will be implemented to further describe and document the process for safety reporting.

8.1.6.1. Investigator Reporting of SAEs and Pregnancy to Sponsor

If any of the adverse events are SAEs as defined by this protocol (see Section 8.1.4) or a pregnancy (see Section 8.1.5), special procedures will be followed. All such events will be reported to the Sponsor designee, Worldwide Clinical Trials, immediately (and not exceeding 24 hours following knowledge of the event) and followed by follow-up reports as soon as possible, whether or not the events are deemed study drug-related.

Serious Adverse Events must be reported by entering the SAE information in the AE/SAE section of the Electronic Data Capture (EDC) system. The information provided in the EDC system should be as complete as possible, but must contain the following minimum fields:

- Subject number
- Brief description of the SAE (diagnosis or signs/symptoms)
- Serious criteria
- Causality assessment
- Assessment of the intensity of the event

WCT Drug Safety will receive notification of the initial SAE *via* an e-mail alert generated from the EDC system. In the event of any temporary disruption of the EDC system, an alternative SAE reporting mechanism will be available to site personnel; in this instance, a paper SAE Report Form will be available. Site personnel will complete the paper SAE report form, scan and e-mail it within 24 hours to the following address:

drugsafety@wwctrials.com.

Site personnel must complete the AE/SAE section with the SAE information as soon as the EDC system becomes available. Serious adverse events that are ongoing should be followed until resolved or stabilized to a level acceptable to the investigator.

The investigator is obliged to provide additional information as requested by the medical monitor. In general, this will include a description of the event in sufficient detail to allow for a complete medical assessment of the case and independent determination of possible causality. In the case of a subject death, a summary of available autopsy findings, if performed, must be submitted as soon as possible to the contract research organization. However, any supporting information provided should not reveal a subject’s identity beyond the agreed study identifier. The investigator should ensure that information reported is accurate and consistent.

Information not available at the time of the initial report (*e.g.*, an end date for the AE, laboratory values received after the report, or hospital discharge summary) must be documented on a follow-up form. All follow-up information must be reported in the same timelines as initial information.

Any SAEs considered related to the study drug and discovered by the investigator at any interval after the study must also be reported to the Sponsor within 24 hours following knowledge of the event.

8.1.6.2. Sponsor Reporting of SUSARs to Regulatory Authorities

SUSARs are adverse events that are believed to be related to an investigational medicinal product and are both unexpected (*i.e.*, the nature or severity is not expected from the information provided in the Investigator's Brochure) and serious. As stated in the EU ‘CT-3’ Communication from the Commission (2011/C 172/01) and the US Code of Federal Regulations (21 CFR 312.32), for there to be a reasonable possibility of a causal relationship between the event and study drug there must be facts (evidence) or arguments to suggest a causal relationship. Final assessment of expectedness for purposes of regulatory reporting is the responsibility of the Sponsor.

It is the responsibility of the Sponsor to determine whether a reported SAE fits the classification of a SUSAR and to notify the investigator of the decision as soon as possible.

Requirements and unblinding procedures for SUSAR reporting are described below. All SUSAR reporting, whether determined following unblinding during study conduct or apparent only after the study has ended, will adhere to European Directives 2001/20/EC, 21 CFR 312.32 of the U.S. Code of Federal Regulations, Health Canada Food and Drug Regulation C.05.014, and other regions as applicable.

8.1.6.2.1. Unblinding Treatment Allocation

Generally, only SUSARs for which the treatment allocation of the subject is unblinded should be reported by the Sponsor to the pertinent regulatory authorities.

When an event may be a SUSAR, the blind should be broken only for that specific subject. The blind should be maintained for individuals responsible for the ongoing conduct of the study (*e.g.*, management, monitors and investigators) and those responsible for data analysis and interpretation of results at the conclusion of the study (*e.g.*, biometrics personnel).

Unblinded information should only be accessible to those who need to be involved in the safety reporting to pertinent regulatory authorities, independent ethics committees/independent review boards (IECs/IRBs) and DSMBs, or individuals performing ongoing safety evaluations during the trial.

8.1.6.2.2. Fatal or Life-threatening SUSARs

It is the responsibility of the Sponsor to report fatal or life-threatening SUSARs to the pertinent regulatory authorities as soon as possible, but no later than 7 calendar days (or earlier if locally mandated). These will be reported to the DSMB in parallel.

8.1.6.2.3. Other SUSARs

It is the responsibility of the Sponsor to report other SUSARs to the pertinent regulatory authorities as soon as possible, but no later than 15 calendar days (or earlier if locally mandated). These will be reported to the DSMB in parallel.

8.1.6.3. Reporting to IECs/IRBs

The IEC/IRB will be notified of any SUSARs according to local regulations and within the designated timeframe.

The investigator (or prospectively agreed to designee) is required to notify the IEC/IRB of any fatal SUSAR as soon as possible but no later than 7 calendar days after he/she first became aware of the reaction (or earlier if locally mandated).

The investigator (or prospectively agreed to designee) is required to notify the IEC/IRB of any other SUSAR as soon as possible but no later than 15 calendar days after he/she first became aware of the reaction (or earlier if locally mandated).

8.2. Urgent Safety Measures

The Sponsor and investigator may take appropriate urgent safety measures in order to protect the subjects of the clinical study against any immediate hazard to their health or safety.

The Sponsor and the Medical Monitor or designated deputy will be notified of any urgent safety measures taken by the investigator or qualified designee and advised of their responsibility to notify the licensing authority. The investigator or qualified designee will notify the IEC/IRB.

If these measures are taken, the Sponsor or investigator shall immediately give written notice to the pertinent regulatory authorities consistent with the regional/national requirements and IEC/IRB of the measures taken and the circumstances giving rise to those measures. In any event, the written notice shall be no later than 7 days from the date the measures are taken.

8.3. Clinical Laboratory Tests

Scheduled blood and urine samples for hematology and chemistry panels, Vitamin B_{12} levels, folate levels, and urinalysis will be obtained at each visit during the study, including Screening, Baseline (pre-dose), and approximately 2, 6, 13, 26, 39, 52, 65, and 78 weeks after Baseline (or upon early termination). Blood for measurement of TSH will be obtained at Screening and after 26, 52, and 78 weeks (or upon early termination), with a thyroid hormone panel obtained in the event of abnormality (and further follow up as needed). Clinical laboratory testing will be repeated at the 4-week post-treatment follow-up visit (Visit 11).

A trained and authorized person will collect biological (blood and urine) samples from the subject. Blood samples will be collected by venipuncture from a suitable vein.

Kits with supplies for the collection of blood and urine samples will be provided to each study site before the study initiation visit. The kits will be labeled to identify the clinical study, and will include specimen labels, pre-printed laboratory requisition forms, all supplies needed for specimen collection and shipping, instructions for collection and preparation of specimens, and pre-printed forms to expedite shipment.

A protocol-specific laboratory manual will be provided to each study site. The laboratory manual will include contact details, lists of the contents of collection kits, shipment schedule of collection kits, and detailed guidelines and recommendations for completing laboratory requisition and for specimen collection, preparation, storage, and transportation.

A central laboratory will process all scheduled laboratory blood samples unless otherwise noted. Data will be transferred electronically for inclusion in the database. Investigators are to enter the results of any testing performed at local laboratories (and the corresponding normal ranges) into the eCRF.

8.3.1. Serum Chemistry

The blood volume for each chemistry panel will be approximately 3.5 mL. Blood samples collected for serum chemistry panels are to be destroyed 1 week after testing has been completed. The chemistry panel will include the following analytes:

Sodium	Gamma-glutamyl transpeptidase (GGT)
Potassium	Alanine transaminase (ALT)
Calcium	Aspartate transaminase (AST)
Albumin	Glucose (random, not fasted)
Total protein	Creatinine*
Blood urea nitrogen, urea	Phosphate
Total and direct (conjugated) bilirubin (indirect bilirubin calculated as the difference)	Triglycerides
Alkaline phosphatase	Cholesterol (total)
Lactate dehydrogenase (LDH)	Uric acid
Creatine kinase (CK)	Chloride

* Creatinine clearance is to be estimated by the central laboratory using the Cockcroft-Gault equation

8.3.2. Hematology

The blood volume for each hematology panel will be approximately 2.0 mL. Blood samples collected for hematology panels are to be destroyed 3 days after testing has been completed. The hematology panel will include the following analytes:

Hematocrit	White blood cells (WBC) and absolute and percent differential
Hemoglobin	
Mean corpuscular hemoglobin (MCH)	(neutrophils, lymphocytes, monocytes, eosinophils, basophils); any abnormal cells will be noted
Mean corpuscular hemoglobin concentration (MCHC)	
Red blood cells (RBC) and any immature forms	Platelet count
RBC morphology	Mean cell volume (MCV)
Reticulocyte count and percent	

If the investigator is concerned on the basis of significant hematological abnormalities that Heinz bodies may be present, a blood sample may be sent to the local laboratory for assessment and screening of Heinz bodies; blood samples should be as fresh as possible at the time of slide preparation. If Heinz bodies are present, study medication must be stopped (see Section 6.3.3.1) and hematological indices monitored until this resolves. Another blood sample will be obtained within 1 week and the percent of erythrocytes with Heinz bodies will be recorded if Heinz body determination is considered to be indicated by the investigator.

8.3.3. Urinalysis

Urine samples for urinalysis will be analyzed by the central laboratory using the iQ 200 Systems by IRIS (International Remote Imaging Systems, Inc.). The instrument combines all analyses into one system. Specific gravity is determined by refractometry. Flow microscopy is used to measure RBCs, WBCs, WBC clumps, hyaline casts, unclassified casts, squamous epithelial cells, non-squamous epithelial cells, bacteria, yeast, crystals, mucus, and sperm. A dual wavelength (to adjust for potential colorimetric interference) dipstick will be used for the following chemistries: pH, glucose, ketones, protein, bilirubin, blood, nitrites, and urobilinogen. Color and turbidity will also be reported. These samples will be destroyed 1 week after testing.

Because MT potentially interferes with colorimetric measurements, extra samples will be prepared. If ≥ 2+ protein is reported by dipstick or results cannot be reported due to interference, an unpreserved sample will be analyzed for microalbumin by nephelometry; these samples will be discarded within 2 weeks of receipt. An additional sample will be stored frozen at ≤ -20°C if needed for further analysis to be performed as needed (*e.g.*, sodium concentration and/or renal biomarkers). These frozen samples will be stored for the period of the study, expected to be at least 34 months (depending on recruitment rate).

8.3.4. Other Laboratory Tests

Vitamin B_{12} and folate will be analyzed by the central laboratory using Beckman Coulter Access® Immunoassay Systems which are based on competitive-binding immunoenzymatic assays. Folate levels can be measured accurately over the analytical range of 1.0 to 24.8 ng/mL (2.27 to 56.2 nmol/L) and Vitamin B_{12} over the range of 50 to 1500 pg/mL (37 to 1107 pmol/L). Plasma levels are quantitated by chemiluminescence. Blood samples collected for measurement of Vitamin B_{12} and folate will be destroyed 1 week after testing has been completed.

TSH will be measured at Screening and after 26, 52, and 78 weeks on treatment (or upon early termination); blood samples collected for this purpose will be destroyed 1 week after testing has been completed. In the event of elevation, free triiodothyronine (T_3) and thyroxine (T_4) are to be measured at the next visit. If abnormal, the subject is to be further evaluated (including possibly by measuring thyroxine-binding globulin [TBG] and T_3 uptake; such testing should be undertaken at an unscheduled visit). T_3 uptake samples should be shipped ambient to the central laboratory; samples for TBG measurement should be shipped frozen (-20 ºC) to the central laboratory and will be forwarded to ARUP Laboratories in Salt Lake City, UT, USA. Blood samples collected for measurement of TBG and T_3 uptake will be destroyed after a storage period of 3 months from the time of sample collection at the study site.

Haptoglobin will be measured at Screening (Visit 1) only[5]; separate blood samples will be obtained for this purpose and will be destroyed 1 week after testing has been completed.

A serum pregnancy test for qualitative testing for the beta subunit of human chorionic gonadotropin (β-hCG) will be obtained for all women of childbearing potential at Screening, Baseline (pre-dose), and each subsequent study visit (or upon early termination), including the 4-week post-treatment follow-up visit (Visit 11). In the event of a borderline result, testing will be repeated and if the result is still borderline, quantitative testing for β-hCG will be performed.

---

[5] Haptoglobin may also be measured in response to signs or symptoms of methemoglobinemia or hemolytic anemia.

Subjects are to be screened for G6PD deficiency at Screening (Visit 1) using a commercially available kit supplied to the sites for on-site testing, or by using a suitable laboratory. Blood samples obtained for measurement of G6PD should be immediately discarded after testing has been completed.

8.3.5. Review of Laboratory Results

Laboratory results from each study visit will be assessed in a timely manner. The investigator or an authorized physician sub-investigator must interpret the laboratory findings and confirm their review.

The clinical significance of all laboratory values which are outside the laboratory normal reference range should be noted and commented upon by the investigator.

Abnormal values which are considered by the investigator to be clinically significant, taking the age of the subject into account, should be documented as an AE, unless accounted for by a pre-existing medical condition detailed in the subject's medical history. The diagnosis associated with a clinically significant laboratory abnormality generally should be recorded as the underlying abnormality or diagnosis (*e.g.*, renal insufficiency) if there is sufficient overall information to permit a diagnosis to be made by the investigator. Otherwise, the observed deviation in the laboratory result should be recorded (*e.g.*, elevated creatinine).

The investigator must review and assess laboratory results that represent potential AESIs, *i.e.*, methemoglobin and other hematology panel results including hemoglobin, RBC, reticulocytes, or the appearance of Heinz bodies in the RBCs. If any of these suggest a potential increase in methemoglobin levels, or hemolytic anemia, treatment interruption should be considered (see Section 6.3.3.1). In the event of borderline or deficient folate values not corrected by folate replacement, subjects should be referred to their primary care physician for evaluation and correction or the medical monitor consulted. In the event of borderline or deficient Vitamin B_{12} values (see Section 4.7.8), subjects should be referred to their primary care physician for evaluation and correction or the medical monitor consulted.

Additional tests and other evaluations required to establish the significance or etiology of an abnormal result or to monitor the course of an AE may be obtained from the central laboratory. If a local laboratory needs to be used in some circumstances, the data and corresponding normal ranges will be recorded in the eCRF, entered directly from a laboratory slip. When clinically indicated, the medical monitor should be informed in such circumstances. In particular, if a clinically significant abnormal result is observed that is not resolved by the following visit, repeated tests should be performed until resolution of the abnormality.

8.4. Pulse Co-Oximetry

Methemoglobin and oxygen saturation will be measured at Screening, Baseline (within 1 hour prior to dosing and approximately 2.5 hours post-dose), and every subsequent visit thereafter (approximately 2, 6, 13, 26, 39, 52, 65, and 78 weeks after Baseline, or upon early termination). Testing will be repeated at the 4-week post-treatment follow-up visit (Visit 11).

The measurements will be performed using a handheld pulse co-oximeter (model Rad-57) provided by the Masimo Corporation, Irvine CA (FDA 510(k) Number – K042536). The device uses variable wavelengths to measure methemoglobin. Because this is a light-emitting device, it should be left on only so long as needed to obtain the recording(s) (20 seconds is recommended).

Initially, the device is to be applied to the index, middle, or ring fingers. Any methemoglobin values recorded as greater than 2.0% should be confirmed; two immediate repeat measurements should be obtained such that a total of three methemoglobin readings are obtained on three different fingers at a single visit. The mean of the three readings will be calculated automatically in the clinical database and used as the basis for safety monitoring decisions.

Additional monitoring is necessary for any mean methemoglobin values recorded as greater than 3.5% (see Section 6.3.3.1).

All individual and calculated mean readings will be captured in the clinical database and included in the eCRF.

In a validation study (Barker *et al.*, 2006) performed in comparison to standard co-oximetry, methemoglobin was reported with a bias of 0 and a precision of ±0.45%. Consideration was initially given to using standard co-oximetry to perform the methemoglobin measurement; however, in comparison to standard co-oximetry for which interference is well-documented (Gourlain *et al.*, 1997), pulse co-oximetry allows for less interference from other substances in the blood. The reading is performed quickly, and eliminates the issue of processing whole blood samples as well as any problems with sample degradation.

8.5. Vital Sign Measurements

In the clinic, measurements of the vital signs described below will be made by a trained and authorized person.

8.5.1. Temperature and Respiratory Rate

Oral (sublingual) temperature and respiratory rate (observed over 15 seconds and recorded in breaths/minute) will be recorded at Screening, Baseline (Visit 2), and every subsequent visit thereafter (or upon early termination), including the 4-week post-treatment follow-up visit (Visit 11).

At Visit 2 (Day 1), oral temperature and respiratory rate will be recorded within 1 hour prior to dosing and hourly while subjects are in the clinic, *i.e.*, for at least 4 hours.

Following discharge, subjects who are receiving serotonergic medications and their caregivers will be given an oral thermometer for continued measurement at home. Temperature measurements are to be performed three times a day (in the morning, afternoon, and evening) until approximately 72 hours after the first dose. Each measurement will be recorded in a diary provided by the site to be returned to the site at Visit 3 (after 2 weeks). Given that caregivers will be contacted by telephone 5-7, >7-14, >14-24, 44-52, and 68-76 hours after the first dose of study drug (with a minimum of 1 hour between contacts) and queried for the presence of signs and symptoms of serotonin toxicity in the subject, they will be instructed to have the temperature diary available during these telephone contacts.

8.5.2. Blood Pressure and Pulse

Blood pressure and pulse will be recorded at Screening, Baseline (within 1 hour prior to dosing and approximately 2 hours post dose), and every subsequent visit thereafter (or upon early termination), including the 4-week post-treatment follow-up visit (Visit 11).

At Screening and on Day 1, blood pressure and pulse shall be measured with subjects in a seated position for at least 5 minutes, and again 2 minutes after standing. Thereafter, they will be measured with subjects in the seated position only (for at least 5 minutes).

A manual or automated sphygmomanometer will be used to measure systolic and diastolic blood pressure. Blood pressure results will be recorded in mmHg. Pulse rate will be measured in the radial artery for 60 seconds and will be recorded as beats/minute. If possible, blood pressure and pulse rate preferably should be measured in the same arm at each visit.

8.5.3. Weight and Height

Weight will be measured at Screening (Visit 1), Baseline (Visit 2, pre-dose), at each on-treatment visit beginning with Week 6 (Visits 4 - 10), and at the 4-week off-treatment follow-up visit (Visit 11). Weight will be measured while the subject is clothed with shoes off and recorded in kilograms (kg).

Height will be measured in centimeters at Screening (Visit 1).

8.6. Electrocardiography

At Screening (Visit 1), 12-lead electrocardiogram (ECG) recordings will be obtained in triplicate (within an approximate 2- to 5-minute interval) with the subject in a supine position, suitably rested (for at least 5 minutes). At Baseline (Visit 2), 12-lead ECGs will be performed before administration of the first dose of study drug; the results should be reviewed by the investigator to confirm that the subject may be dosed (see Exclusion Criterion No. 13). The ECGs are to be repeated approximately 3 hours post-dose. At both of these Visit 2 time points, ECG measurement will include triplicate recordings (within an approximate 2- to 5-minute interval). Data entered in the eCRFs will be the average of the three readings. The confirmation of the decision regarding the subject's eligibility for the study is to be based on the local read. A local cardiology consult should be sought if the investigator deems it necessary. For evaluation of subjects with left bundle branch block, a cardiology consult is strongly recommended. In circumstances where a subsequent central reading reports a significantly different QTcF compared with the local read, the discrepancy and further evaluation is to be addressed by medical monitors on a case to case basis.

Subsequently, 12-lead ECGs will be obtained at each study visit, *i.e.*, after 2, 6, 13, 26, 39, 52, 65, and 78 weeks (or upon early termination) and at follow-up (Visit 11); these ECG measurements will be obtained as a single recording, unless there are emergent abnormalities deemed clinically significant by the investigator, in which case triplicate ECGs should be obtained.

All 12-lead ECGs, including those performed at Screening, will be of at least 10-second duration. For subjects with well controlled atrial fibrillation (*i.e.*, heart rate ≤ 84 bpm and appropriate anticoagulation), triplicate ECGs are mandatory at all visits. Electrocardiograms will be acquired according to instructions provided by a centralized ECG reading facility where the ECGs will be centrally assessed. At a minimum, interval data (Fridericia corrected QT interval, QTcF), ventricular rate, and overall interpretation will be reported for each ECG. Data will be transferred electronically for inclusion in the database. Investigators are to enter the machine-read results and any clinical interpretations for the Screening and Baseline (Day -1) ECGs into the eCRF.

Central reading will not be available in subjects with uncontrolled atrial fibrillation (heart rate > 84 bpm) or for subjects with intraventricular conduction defects; therefore, a local ECG reading must be performed. It is recommended that a local cardiologist be involved for correct interpretation and measurements of ECG data, especially in subjects with left bundle branch block.

8.7. Physical and Neurological Examinations

Complete physical and neurological examinations will be conducted by the investigator or a physician sub-investigator at Screening (Visit 1). The complete physical examination is to consist of evaluation of the skin, head, eyes (including size and reactivity of the pupils), ears, nose, throat, neck, thyroid, lungs, heart, lymph nodes, abdomen, and extremities (to include deep tendon reflexes, clonus, and muscle rigidity). Any abnormalities noted should be described.

The complete neurological examination is to consist of evaluation of appearance and behavior (including observation for tremor and abnormal movements) and evaluation of the following: speech, cranial nerves (2-12), motor (muscle strength), muscle tone, sensory abnormalities, coordination, gait, and tendon reflexes. At Screening, the primary aim of this assessment is to exclude neurological disorders other than the condition of interest. Any abnormalities noted should be described.

At Visit 2 (Day 1), targeted examinations (as described below) are to be recorded within 1 hour before and approximately 3 hours after administration of the first dose of study drug (and after completion of ECG measurements); these are to be repeated as needed for subjects who remain in clinic longer than 4 hours. Thereafter, targeted examinations are to be performed at each subsequent visit (or upon early termination), including the 4-week post-treatment follow-up visit (Visit 11).

The targeted examinations are to be focused on, but not limited to, evaluating subjects for potential serotonin toxicity and also as clinically indicated (*e.g.*, targeted to any changes in medical history, in the event of an AE that requires such follow up, or any reported change in the subject's physical condition). At a minimum, they are to include evaluation of deep tendon reflexes, clonus, and muscle rigidity; size and reactivity of pupils; dryness of oral mucosa; intensity of bowel sounds; skin color; and presence or absence of diaphoresis.

Additional targeted examinations may be performed as clinically indicated (*e.g.*, in the event of an AE that requires such follow up or any reported change in the subject’s physical condition).

Findings will be documented in the subject's medical record and in the eCRF.

8.8. Serotonin Toxicity (Syndrome)

Signs and symptoms of potential serotonin toxicity will be rated in the clinic by medically qualified personnel for all subjects at Baseline (Visit 2) and each subsequent visit (or upon early termination), including the follow-up visit (Visit 11). At Baseline (Visit 2), this will be conducted pre-dose and for at least 4 hours after the first dose of study drug while the subject is in the clinic; an assessment is to be completed just prior to discharge from the clinic. This will be rated using a 20-item Serotonin Toxicity (Syndrome) Diagnostic Interview and Rating Guide (Section 24.2). Rating is to be based on adverse events query, targeted physical and neurological testing, vital sign measurements, and MMSE testing.

Upon discharge from the clinic, subjects using serotonergic medication at Baseline will be required to remain in close proximity to a local hospital and remain in telephone contact with the clinic for at least 12-14 hours post-dose and reimbursement for accommodations will be made available if requested by the subject and/or caregiver. Caregivers of subjects using serotonergic medication will also be contacted by telephone 5-7, >7-14, >14-24, 44-52, and 68-76 hours post-dose (with a minimum of 1 hour between contacts); if indicated, more frequent contacts will be made. The presence and absence of signs and symptoms of serotonin toxicity are to be assessed using the Serotonin Toxicity Telephone Assessment described in Section 24.3. Possible post-randomization cases of serotonin toxicity will be considered important medical events and reported as per the procedure for SAEs (see Section 8.1.4 for reporting obligations).

8.9. Columbia-Suicide Severity Rating Scale (C-SSRS)

Suicidal behavior and suicidal ideation will be evaluated using the C-SSRS at Screening (Visit 1); Baseline (Visit 2) post-dose (prior to discharge from the clinic); and at each subsequent visit (or upon early termination), including the 4-week post-treatment follow-up visit (Visit 11). At each assessment, subjects and their caregivers will be asked to indicate whether or not subjects have had an actual attempt, an interrupted attempt, or an aborted attempt and, if so, the number of attempts since the last assessment and in the last week. Subjects will also be queried regarding five aspects of suicidal ideation and their frequency since the last assessment and in the last week: wish to be dead, non-specific active suicidal thoughts, active suicidal ideation with any method (not plan) without intent to act, active suicidal ideation with some intent to act (without specific plan), and active suicidal ideation with specific plan and intent. Any post-randomization affirmative response will be considered an important medical event and reported as per the procedures for an SAE (see Section 8.1.4 for reporting obligations).

Subjects showing indications of suicidal ideation, intent, or action, or who have engaged in self-injurious behavior should be referred for psychiatric evaluation.

9. OTHER ASSESSMENTS

9.1. Magnetic Resonance Imaging (MRI) and ^{18}F-Fluorodeoxyglucose Positron Emission Tomography (FDG-PET)

9.1.1. General Considerations

Brain MRI imaging plays a significant role in this protocol, not only for eligibility evaluation of the subjects, but also as the principal outcome to support disease modification by LMTM. FDG-PET will also be performed for efficacy purposes at all study sites, and will be assessed by an independent nuclear physician, who is trained and not otherwise involved in the clinical conduct of the study. Change in whole brain, ventricular and hippocampal volumes will be quantified at the imaging core laboratory.

The independent central imaging core laboratory will be responsible for image collection, checking of the quality of imaging data, anonymization of the images, pre-processing of MRI and FDG-PET images, presentation of the data to the independent reader(s) (for MRI eligibility and safety evaluations and FDG-PET), and analysis of the MRI and FDG-PET data. The core laboratory will provide SAS datasets to the data management and statistics facility for analysis. All systems and processes used for independent and central reads of this trial will be 21 CFR

Part 11 compliant. Before commencement of independent central evaluations, independent imaging charters will be developed that will describe in detail the imaging acquisition protocols, image collection procedures, quality assurance procedures, site training procedures, reader training, image evaluation procedures (central determination of subjects' eligibility, efficacy, and safety), and communication plans.

All sites will be prospectively trained about imaging requirements including scanner requirements, image acquisition, image transfer to the core laboratory, and timelines that are critical for this trial.

9.1.2. Site Selection and Qualification

A site imaging technical evaluation questionnaire will be distributed to potential clinical sites to evaluate their technical and personnel capabilities that would include machine description, availability of phantoms (if applicable), onsite availability of modality-specific technologist or physicist (depending on MRI or FDG-PET site), site experience in evaluating brain FDG-PET and/or MRI, experience in AD and other dementia trials, *etc.* The site's capability of producing quality data that are necessary for this trial will also be evaluated by appropriate phantom imaging and/or review of imaging data of the site's first subject. Continuous monitoring of the quality of imaging data will be performed throughout the trial. Technical details of quality assurance procedures will be described in a separate imaging manual.

9.1.3. Imaging Methods

A separate imaging manual will be developed that will outline imaging methods for both FDG-PET and brain MRI to harmonize image acquisition across the sites. FDG-PET should be obtained with computerized tomography (CT); brain-dedicated high resolution PET devices, such as the Siemens High Resolution Research Tomograph (HRRT) system, that use a transmission source for attenuation correction will also be considered from expert sites.

9.1.3.1. FDG-PET

An FDG-PET scan will be performed using CT or a brain-dedicated high-resolution PET device, after informed consent has been provided, either at the Screening visit (referred to as Screening/Baseline) or subsequently within 42 days before Baseline (Visit 2). The Screening/Baseline FDG-PET will be used as a baseline for comparison with the Week 39 (Visit 7) and Week 78 (Visit 10) or early termination visit FDG-PET. Use of sedatives prior to FDG-PET scans is not permitted.

If the initial Screening/Baseline FDG-PET is not of sufficient quality to serve as an adequate baseline, then the scan must be repeated within 42 days before Visit 2 as long as the repeat PET does not cause radiation exposure to the subject that exceeds the limits for the clinical trial (4.7 mSV for a single scan and 23.5 mSV in total). If the repeat scan cannot be accomplished within the 42-day window, then the subject must be reconsented and rescreened. For subjects who are rescreened for other reasons and an acceptable FDG-PET scan was already completed during the original screening window, the scan should only be repeated if the original FDG-PET scan occurred > 42 days prior to Visit 2.

The Weeks 39 and 78 FDG-PET scans should be performed within ± 14 days of the designated visit. The allowable time window for the end of treatment (Week 78) FDG-PET scan can be extended to up to 28 days after the last dose of study drug only with Sponsor/medical monitor

approval. The FDG-PET scan performed at Week 39 can be repeated if the central reader determines a quality issue; the FDG-PET scan performed at Week 78 cannot be repeated.

If it is planned that a subject who has completed the study through the Week 78 visit will be entering the open-label extension study, then the end of treatment FDG-PET scan should be performed before the subject begins treatment with open-label LMTM in the extension study.

If a subject discontinues study drug and/or withdraws from the study prematurely (before completion of the Week 78 visit), then an early termination visit should be conducted at which time all assessments identified for the Week 78 visit should be performed. If the subject's last FDG-PET scan was performed < 90 days prior to the early termination date, no additional FDG-PET scan is required. If the subject's last FDG-PET scan was performed ≥ 90 days prior to the early termination date, the FDG-PET scan must be repeated as part of the early termination visit assessments within the time window of the early termination visit (*i.e.*, ± 14 days of the last dose of study drug); the allowable time window for this scan may be extended to up to 28 days after the last dose of study drug only with Sponsor/medical monitor approval.

An overview of the image acquisition method is given below.

1. The subject should fast for at least 6 hours prior to the administration of FDG.
2. Blood glucose should be below 180 mg/dL prior to the administration of FDG (based on current Alzheimer's Disease Neuroimaging Initiative (ADNI) recommendations). This is routinely done in laboratories that use FDG for a variety of clinical indications and is carried out by sticking the finger and measuring glucose levels using the available standard glucomonitors for this purpose.
3. The subject should be injected while resting comfortably in a dimly lit room and remain in this setting for a period of approximately 30 minutes. Any conversation or other interactions that can affect the brain glucose metabolism should be avoided during this period of time.
4. A standard dose of 5 mCi (± 10%) should be injected intravenously over a period of 1 minute through an indwelling catheter ideally placed in the antecubital region (no injection should be made by needle inserted into a vein, which may be subject to extravasation).
5. At 30 minutes (± 5 minutes) following the administration of FDG, the subject should be positioned into the PET instrument and PET (and CT, if applicable) images should be acquired over 30 minutes. The field of view should be decreased to the brain mode so that the highest resolution can be achieved by this approach. A dynamic 6×5-minute protocol should be used.

Subjects will be exposed to radioactivity from the tracer for the FDG-PET scan and, if applicable, radiation from the X-rays in the CT scan. A summary of the estimated amount of radiation exposure from each scan as well as over the course of the study is provided in the table below.

Radiation Source	Amount of Radiation
FDG-PET	3.5 mSV (350 mREM)
Non-diagnostic CT	1.2 mSV (120 mREM)
Total radiation exposure from a single FDG-PET/CT scan	4.7 mSV (470 mREM)
Total radiation exposure over course of study (5 scans, if repeat of baseline scan and Week 39 scan are both required)	23.5 mSV (2350 mREM)

Source: RadMD, LLC/MNI LLC
FDG=^{18}F-fluorodeoxyglucose; PET=positron emission tomography; REM=roentgen equivalent man; mREM=milliREM, or one thousandth of a REM; SV=sievert; mSV=millisievert or one thousandth of a seivert; CT=computed tomography
Note: 1 mSV=100 mREM

9.1.3.2. Brain MRI

In this trial only GE, Siemens, or Philips (1.5-Tesla and 3.0-Tesla) machines will be used to reduce the variability in the imaging data. An MRI scan will be performed at Screening (referred to as Screening/Baseline) and at Weeks 13, 26, 39, 52, 65, and 78, or upon early termination. If needed, MRI scans do not need to occur on the same day as efficacy ratings but should occur within the acceptable window. If the MRI scan cannot be performed due to subject's movement, appropriate sedation prior to scanning is permissible.

The Screening/Baseline MRI scan will be performed, after informed consent has been signed, at the Screening visit or subsequently within 42 days before Baseline (Visit 2). Results of the Screening/Baseline MRI must be available by/at Baseline so that an eligibility determination can be made before a subject is considered for randomization (see Inclusion Criterion No. 1 in Section 5.1 and Exclusion Criterion No. 2 in Section 5.2).

If the initial Screening/Baseline MRI scan is not of sufficient quality, then a repeat Screening/Baseline MRI may be required and performed (within 42 days before Visit 2). If the repeat scan cannot be accomplished within the 42-day window, then the subject must be reconsented and rescreened. For subjects who are rescreened for other reasons and an acceptable MRI scan was already completed during the original screening window, the scan should only be repeated if the original MRI scan occurred > 42 days prior to Visit 2. The Screening/Baseline MRI will also be used as a baseline for comparison with the Weeks 13, 26, 39, 52, 65, and 78 (or early termination visit) MRIs. The MRI scans should be performed within ±14 days of the designated visit and will be evaluated for whole brain, ventricular and hippocampal volumes. If out of window assessments are undertaken, these will be categorized/labeled according to the intended visit designation, regardless of being out of window. The allowable time window for the end of treatment (Week 78) MRI scan may be extended to up to 28 days after the last dose of study drug only with Sponsor/medical monitor approval. If it is planned that a subject who has completed the study through the Week 78 visit will be entering the open-label extension study, then the end of treatment MRI scan should be performed before the subject begins treatment with open-label LMTM in the extension study.

All post-Baseline MRI scans will be evaluated for safety (ARIA). If evidence of ARIA is noted meeting the discontinuation criteria listed in Table 6-6, dosing should be permanently discontinued. Guidance for follow up is included in Section 6.3.3.3.

If a subject discontinues study drug and/or withdraws from the study prematurely (before completion of the Week 78 visit), then an early termination visit should be conducted at which

time all assessments identified for the Week 78 visit should be performed. If the subject's last MRI scan was performed < 90 days prior to the early termination date, no additional MRI scan is required. If the subject's last MRI scan was performed ≥ 90 days prior to the early termination date, the MRI scan must be repeated as part of the early termination visit assessments within the time window of the early termination visit (*i.e.*, ± 14 days of the last dose of study drug); the allowable time window for this scan may be extended to up to 28 days after the last dose of study drug only with Sponsor/medical monitor approval.

An overview of the image acquisition method is given below.

1. The usual MRI subject preparation and safety procedures will be followed. A total duration of approximately 30 minutes will be required.
2. The positioning of the subject in the head coil will follow manufacturer procedures. The MRI sequences utilized in this protocol will be based on the ADNI protocol. The recommended range of acquisition parameters for individual sequences for GE, Siemens, and Philips MRI machines will be developed once technical evaluation forms from all sites have been reviewed and will be described in the imaging manual. The following sequences will be used:
   - T2*-weighted gradient-recalled echo (2-dimensional T2* GRE with slice thickness of 5 mm or less and Echo Time of 20 msec or greater); this sequence enables the detection of microbleeds and superficial siderosis.
   - FLAIR: This sequence allows assessment of other clinically significant focal intracranial pathology and for detection of vasogenic edema; it should be done at all imaging time points.
   - Unenhanced T1-weighted, 3-dimensional sequence (*e.g.*, MP RAGE or SPGR) is required for evaluation of whole brain, ventricular and hippocampal volumes.
   - For eligibility of the subject in addition to sequences described above, the following sequences will also be required:
     - T1-weighted 3-dimensional MP RAGE imaging to exclude space occupying lesions.
     - Diffusion-weighted imaging to exclude a recent vascular event.

9.1.4. Image Transfer and Quality Assurance

The imaging core laboratory will provide an imaging manual to the clinical sites that will provide details about image collection, image transfer, and timelines. Electronic transfer (DICOM format only) is required for this trial for eligibility reads, evaluation of WBV, exploratory efficacy evaluation of ventricular and hippocampal volumes, and safety evaluations for ARIA. Subject data (age, sex and other details) will be removed before central evaluations to reduce the bias and to comply with Health Insurance Portability Accountability Act (HIPAA) and other privacy requirements. Anonymized MRI images are to be immediately transferred to the imaging core laboratory.

Once the images have been received by the imaging core laboratory, they will undergo standardized quality control evaluation by trained technologists. If there are quality issues, the site will be informed and the site will be asked to resolve the quality issues, including rescan of the subject if needed (as allowed; see Sections 9.1.3.1 and 9.1.3.2). Quality issues identified by the independent neuroradiologist will be handled in the same fashion.

9.1.5. Image Evaluation Procedure

All safety and eligibility MRI data will be evaluated centrally by experienced independent readers who are not involved in the clinical conduct of the study and are blinded to subjects' clinical information. Efficacy (whole brain, ventricular and hippocampal volumes) MRI data will be evaluated centrally, as described in Section 9.1.5.3. All FDG-PET data will be evaluated centrally by an experienced independent nuclear physician, who is not otherwise involved in the clinical conduct of the study and blinded to subjects' clinical information. All readers will be prospectively trained before the beginning of independent evaluations. The analysis methods and evaluation methodology, including eCRF details will be described in the Independent Review Charter (IRC).

9.1.5.1. Evaluation of Brain MRI for Subject Eligibility

An experienced neuroradiologist will evaluate the Screening/Baseline brain MRI images for subject eligibility based on the imaging-related inclusion and exclusion criteria (presented in Section 5). If the neuroradiologist identifies any quality issues pertaining to the images, the process detailed in Section 9.1.4 will be followed. If there are no quality issues, the neuroradiologist will proceed with the evaluation.

Subjects will also be evaluated at baseline only for presence and severity of white matter disease based on Fazekas score (Fazekas *et al.*, 1987), rated on a 4-point rating scale as summarized below:

0 = None or a single punctate white matter hyperintense lesion

1 = Multiple punctate lesions

2 = Beginning confluency of lesions (bridging)

3 = Large confluent lesions

Results will be communicated to the sites or designee within 5 business days of image receipt (and resolution of any imaging quality queries) by the core laboratory either confirming imaging eligibility or not confirming eligibility for enrollment (the site radiologist may request re-review based on additional clinical/radiological information; see Section 9.1.6).

9.1.5.2. MRI Evaluation for ARIA

MRI brain scans acquired at Weeks 13, 26, 39, 52, 65, and 78 (±14 days) or early termination will be evaluated for ARIA by a single neuroradiologist. The reader will evaluate the images for changes relative to earlier scans, including with respect to presence or absence of vasogenic edema, macrohemorrhages, microhemorrhages, and superficial siderosis. The findings will be conveyed to the investigator within 5 business days after images are received at the imaging core laboratory if there are no quality issues.

9.1.5.3. Efficacy Evaluations

Both FDG-PET and MRI will be evaluated centrally (FDG-PET scans will be reviewed by an independent nuclear physician) to evaluate the effect of LMTM on modification of disease progression.

The goal of evaluation of MRI is to show that LMTM can reduce the rates of whole brain, ventricular and hippocampal atrophy at 52 and 78 weeks after treatment. This will be shown by

comparing post-treatment data with the baseline data. The MRI data acquired at the clinical sites will be sent to the imaging core laboratory for evaluation of WBV; if an early termination visit MRI scan is performed, it will be sent to the imaging core laboratory for this evaluation. The independent imaging core laboratory will perform quality check before preparing images for evaluation. The whole brain and ventricular volume change evaluations will be quantified using the BBSI and VBSI (Fox and Freeborough, 1997) and the hippocampal volume change will be quantified using the HBSI (Barnes *et al.*, 2007). Changes in whole brain, ventricular and hippocampal volumes will be quantified by the imaging core laboratory. These values of whole brain, ventricular and hippocampal volumes will be utilized for statistical analysis.

The goal of FDG-PET evaluation is to show that LMTM can reduce the decline of glucose uptake at 39 and 78 weeks after treatment. This will be shown by comparing post-treatment data with the baseline data. Exploratory analyses will be performed at 39 and 78 weeks. FDG-PET data acquired at the clinical sites will be sent to the imaging core laboratory. The imaging core laboratory technologist will perform a quality evaluation of the images before the semi-automated drawing of the appropriate region of interest (ROI) on Screening/Baseline and 39- and 78-week images. ROIs will be drawn on the temporal lobes and cerebellum. The principal ROI for analysis will be the temporal lobes. Cerebellum uptake data will be used for uptake image normalization between scans in the same individual and between individuals. Placement of ROI will be confirmed by an independent and experienced nuclear physician. The independent nuclear physician can modify and change the ROIs positions. The extracted ROI values will then be utilized for statistical analysis.

9.1.6. Site Review

The site radiologist will also review the MRI for subject management and preliminary determination of eligibility as well as treatment-emergent abnormalities. A re-review by the central reader may be requested on the basis of additional clinical/radiological information known to the site. Final determination of eligibility from an imaging perspective and presence or absence of ARIA is based on the independent neuroradiologist review.

If there is evidence of ARIA meeting criteria listed in Table 6-6, treatment should be permanently discontinued. Guidance for follow up is included in Section 6.3.3.3. Efficacy assessments are to continue as scheduled and the subject is to be encouraged to continue with study visits until the scheduled end of participation for the subject.

The investigator will always retain the responsibility for management of the subject.

9.2. Resource Utilization in Dementia Questionnaire (RUD Lite)

The RUD was developed as a comprehensive instrument to measure resource use by patients in a clinical trial setting, which in a further step can be calculated into costs (Wimo *et al*., 1998; Wimo *et al*., 2003; Jonsson, 2007). The RUD Lite is a shorter version of the RUD (Wimo and Winblad, 2003). The RUD and RUD Lite assess both formal and informal resource use, making it possible to calculate costs from a societal perspective. The RUD is administered as an interview with the caregiver. The validity and reliability of the RUD instrument has been investigated both in residential care and community care settings by comparing responses to the RUD questions with actual observations of care-giving time (Jonsson, 2007; Wimo and Nordberg, 2007).

The following dimensions are captured on the subjects' part: accommodation/long term care, respite care, hospital care, social service, home nursing care, and work status. In addition, the following aspects are covered from the caregiver perspective: caregiving time for the subject and work status.

The RUD Lite will be performed at Baseline (pre-dose) and at Weeks 26, 52, and 78 (or upon early termination) visits. This assessment is to be performed by raters who are not involved in the ADCS-CGIC or safety ratings (with the exception of the C-SSRS and Serotonin Toxicity Assessment).

9.3. Cerebrospinal Fluid Biomarkers

Cerebrospinal fluid (CSF) samples will be collected at designated study sites only for a subset of subjects who are mentally capable of providing their own separate informed consent and specifically agree to have lumbar puncture performed. This is considered an optional assessment, and any subject may choose freely to not participate in this assessment, yet otherwise continue participation in the study overall.

The CSF samples will be obtained using standard lumbar puncture procedures performed by a certified neurologist, anesthesiologist or anesthetist. The physician will ensure that the subject receives appropriate medical care after the procedure has been performed.

Materials for and details regarding collecting and processing CSF samples will be provided to the investigator. CSF samples will be sent to Covance Central Laboratory Services (CLS) according to provided instructions.

Baseline CSF samples may be collected any time prior to the first dose of study drug so long as all screening procedures have been completed and subject eligibility and willingness to continue have been confirmed. A subsequent sample will be collected at the Week 78 (Visit 10) or early termination visit. The pre-dose CSF sample will be used as a baseline for comparison with the Week 78 (Visit 10) or early termination visit CSF sample. Levels of CSF biomarkers total tau, phospho-tau and $A\beta_{1-42}$ will be measured by the designated central laboratory. The CSF samples will be sent from Covance CLS to the Covance Biomarker Center of Excellence on a monthly basis. Samples are to be batch tested, typically aiming for batches of 32 samples depending on the number and age of available samples, and subsequently destroyed within 10 business days of assay.

The samples are stable for up to 24 months from the time of sample collection at the study site.

9.4. MT Concentrations

Provided the site has a refrigerated centrifuge and adequate capability to reliably freeze samples, blood will be collected at Baseline (Visit 2; pre-dose and approximately 3.5 hours post-dose) and, to the extent possible, at each subsequent on-treatment visit through Week 78 (approximately 20 minutes after ECG recordings) for purposes of population pharmacokinetic (PK) analysis of MT concentrations; samples may also be analyzed for other analytes, such as metabolites, depending on method availability. The time of the prior dose and the time of the blood sample will be recorded and an effort will be made to have a reasonable distribution across the dosing interval. All samples will be tested and disposed of in accordance with the terms of the subjects' consent.

Concentration results will not be made available to the study sites during the conduct of the study. However, results for a given subject (if available) may be provided to the DSMB if requested to aid in interpretation of a significant subject safety issue.

The collection, handling, and shipping of blood samples are described below. These are to be analyzed using validated analytical methods. The analytical results will be reported separately for possible inclusion in future population pharmacokinetic analysis.

9.4.1. Procedure for Blood Sample Collection

Blood samples (6 mL) will be collected into a suitable vacutainer (6.0 mL), as defined in the laboratory manual. All 6 mL of whole blood will be centrifuged at 3000 rpm for 10 minutes at *ca* 4°C; the separated plasma (2 × 1-mL and 2 × 0.5-mL samples) will be transferred into four Nunc polypropylene tubes, as defined in the laboratory manual, and stored at *ca* -20 °C until shipment to the central laboratory for storage. The 2 × 0.5-mL samples will serve as back-up samples for analysis of MT concentrations. The complete sample collection and handling procedures to be followed can be found in the laboratory manual.

9.4.2. Packaging, Labeling, and Shipping of Blood Samples

The samples must be labeled with unique IDs; other labeling information will be detailed in the laboratory manual. Labels must remain intact and indelible throughout processing and frozen storage. Samples will be clearly distinguished from the other bioanalytical samples.

Samples are to be transported in insulated containers filled with dry ice. They will be shipped to the central laboratory where they will be stored frozen and shipped in batches to the analytical laboratory. Samples are to be destroyed after a storage period of 3 months following completion of the clinical study report, which is expected to be a period of approximately 4 years.

9.4.3. Analytical Laboratory

Samples are to be analyzed at the following laboratory:

University of Aberdeen GLP Test Facility
Meston Building
Old Aberdeen
Aberdeen
AB24 3UE
Tel: +44 (0) 1224 272 945
Fax: +44 (0) 1224 272 921

9.5. Genotyping

For subjects who agree and for whom a separate informed consent signed by the subject or legally acceptable representative is obtained to participate in genotyping evaluation of Apolipoprotein E (ApoE), a single blood sample will be obtained. This blood sample may be obtained from a subject at any time after eligibility for randomization and continued participation in the study has been confirmed for that subject. A volume of approximately 10 mL is to be collected in a 10-mL K_2 EDTA plastic tube and immediately stored under frozen conditions (placed on dry ice) prior to shipment.

Samples should then be shipped frozen (on dry ice) to the central laboratory (Covance) on the day of collection where they will be stored at -70°C. Samples will be sent in batches (frozen, on dry ice) to the analytical laboratory responsible for performing the analysis (Athena Diagnostics, Marlborough, MA, USA). Sample analysis will take place within approximately 10 weeks after the collection of samples; samples will be destroyed 6 months after testing is completed.

Genotyping results will not be provided to the study sites or to subjects.

10. STATISTICAL PLAN

A statistical analysis plan (SAP) will be written and finalized prior to database lock and unblinding of treatment codes. Changes from analyses planned in this protocol will be documented in the SAP and/or study report. The database will be locked after a blinded review of data and all queries are resolved and decisions made about the inclusion or exclusion of any spurious data and the handling of unused or missing data.

10.1. Efficacy Endpoints

10.1.1. Primary Efficacy Endpoints

The co-primary efficacy endpoints for the clinical demonstration of efficacy are the following:

- ADAS-cog$_{11}$
- ADCS-ADL$_{23}$

10.1.2. Secondary Efficacy Endpoints

As a secondary efficacy endpoint, brain MRI is to be analyzed to demonstrate disease modification based on the following:

- Reduction in decline in WBV using change from Baseline as measured by the BBSI

Secondary clinical endpoints include the following:

- ADCS-CGIC
- MMSE
- NPI
- MADRS

10.1.3. Exploratory and Other Endpoints

The following endpoints will be considered in an exploratory fashion:

- Reduction in the rate of atrophy as shown by change in ventricular and hippocampal volumes estimated using boundary shifting techniques as evaluated by brain MRI
- Decline in glucose uptake in the temporal lobe on FDG-PET imaging using pre-defined volume-of-interest extracted values
- RUD Lite

The following CSF biomarkers will be considered (in subjects who are mentally capable of providing their own separate informed consent and specifically agree to have lumbar puncture performed):

- total tau
- phospho-tau
- Aβ_{1-42}

Blood will be collected (in subjects by or for whom legally acceptable consent is provided) to explore the influence of the ApoE genotype on the primary and selected secondary endpoints.

Blood will also be collected for purposes of population pharmacokinetic analysis. This will be detailed in a separate SAP and will be reported separately (together with data from other studies).

10.2. Sample Size Justification

It is planned that approximately 700 subjects will be randomized in a 1:1 ratio to LMTM 200 mg/day or placebo groups, respectively (350 subjects in the LMTM group and 350 in the placebo group).

The assumptions and calculations that form the bases of the sample size are presented below. (See Section 10.4 for monitoring of these assumptions and retention, a key factor in analyses.)

10.2.1. ADAS-cog$_{11}$

The ADAS-cog$_{11}$, a co-primary clinical efficacy endpoint, is expected to decline by approximately 4.15 ± 8.76 ADAS-cog$_{11}$ units (weighted mean ± standard deviation [SD]) in untreated/placebo-treated subjects with Alzheimer's disease of mild severity at 18 months. The estimates of placebo decline and standard deviation are based on a meta-analysis of baseline MMSE *versus* ADAS-cog decline from data provided from Doody *et al*. (2014), Salloway *et al*. (2014), and data available from the Critical Path Initiative[6] . These more recent studies suggest that placebo decline is less than estimated from earlier studies (5.4 ± 8.6). The earlier estimates of placebo decline and standard deviation were based on data provided from Gold (2007) and supplemented by data from Aisen *et al*. (2003), Aisen *et al*. (2008), Sano *et al*. (1997), Wilcock *et al*. (2008), the Myriad Phase 3 trial reported at the International Conference on Alzheimer's Disease (ICAD) (2008)[7], TauRx Phase 2 data (2008), more recent Sponsor public presentations of data from the solanezumab[8] and bapineuzumab[9] Phase 3 studies, and data available from the Critical Path Initiative[10].

Based on the use of a two-tailed test at the 5% level of significance, and assuming that the SD of the ADAS-cog$_{11}$ change from baseline to Week 78 is 8.76 units, this sample size will provide 90% power to detect a mean difference between groups of 2.16 units, under the assumption that the effect size corresponds to a 52% reduction in the expected rate of decline and assuming that the placebo decline is 4.15 ± 8.76 units. An effect size of 2.16 units corresponds approximately to the effect size of currently available symptomatic treatments (Birks, 2006). Power determination was based on an ANOVA for difference in expected change from baseline without covariates. The details of the primary efficacy analysis model for ADAS-cog$_{11}$ are provided in Section 10.3.2.3.

10.2.2. ADCS-ADL$_{23}$

The ADCS-ADL$_{23}$, a co-primary clinical efficacy endpoint, is expected to decline by approximately 9.21 ± 14.06 ADCS-ADL$_{23}$ units (weighted mean ± SD) in untreated/placebo-treated subjects with Alzheimer's disease of mild severity at 18 months. The estimates of placebo decline and standard deviation are based on a meta-analysis of baseline MMSE *versus*

[6] C-Path Online Data Repository, Tucson, AZ, Critical Path Institute, 2010.
[7] Subsequently published by Green et al. in the Journal of the American Medical Association (2009).
[8] Seimers and Ricks, Boston, MA, USA 8 October 2012
[9] Salloway et al., Clinical Trials in Alzheimer's Disease Conference, 29 October 2012
[10] C-Path Online Data Repository, Tucson, AZ, Critical Path Institute, 2010.

ADCS-ADL_{23} decline at 18 months from data provided from the Myriad Phase 3 trial reported at International Conference on Alzheimer's Disease (ICAD) (2008) and by Doody *et al*. (2014).

The above sample size will provide at least 90% power for detecting a treatment difference of 3.50 units at a two-sided alpha of 0.05, under the assumption that the effect size corresponds to a 38% reduction in the expected rate of decline and assuming that the placebo decline is -9.21 ± 14.06 units. Power determination was based on an ANOVA for difference in expected change from baseline without covariates. The details of the primary efficacy analysis model for ADCS-ADL_{23} are provided in Section 10.3.2.3.

10.2.3. ADCS-CGIC

Approximately 80% of subjects in the placebo group are expected to have a worsening in ADCS-CGIC, a secondary clinical efficacy endpoint. This estimate is based on combined data from the TauRx Phase 2 trial and the simvastatin CLASP study[11]. If the percentage of subjects declining in a treated group is reduced to 69%, then there will be 90% power to show a statistically significant difference at a two-sided alpha of 0.05. This corresponds to a 14% reduction in the proportion of subjects worsening[12].

10.2.4. MRI

The decline in WBV as measured by BBSI over 18 months is estimated as -20,696 mm^3 with a SD of 12,187 mm^3 in untreated/placebo-treated subjects. The estimates of placebo decline and SD are based on an analysis of data from the ADNI database[13] at 12 and 24 months provided by BioClinica. Assuming a scan rate of 100%, a study withdrawal rate of 30% over 18 months, and a loss of scan data points for imaging reasons of 10%, the final retention rate for scan-pairs is expected to be 63% (*i.e*., 221 scan-pairs in each arm). Based on the use of a two-sided test at the 5% level of significance, there is therefore 90% power to detect a scan effect of +3,829 mm^3 or an 18.5% reduction in rate of brain atrophy relative to that expected in the control arm at 18 months.

10.3. Statistical Analysis

10.3.1. Analysis Populations

All analyses (other than sensitivity analyses to be described in the Statistical Analysis Plan) will be undertaken with subjects included in the randomly assigned treatment group/stratum. The various analysis populations are defined below.

The Safety population will include all randomized subjects who take at least one dose of study drug.

The Intent-to-Treat (ITT) population will include all randomized subjects.

The Modified Intent-to-Treat (MITT) population will include all appropriately consented randomized subjects who take at least one dose of the study drug and have a Baseline and at least one post-Baseline efficacy measurement.

[11] http://www.clinicaltrials.gov/ctshow/NCT00053599; http://www.alzforum.org/therapeutics/simvastatin
[12] The estimate of power is based on a simple comparison of placebo *versus* a single treated group.
[13] Estimates based on ADNI 1 data downloaded from the ADNI website (https://ida.loni.ucla.edu) on July 22, 2014

The Per Protocol (PP) population will include all MITT population subjects who do not have any major protocol violations. Major protocol violations will be determined prior to unblinding.

The Pharmacokinetic (PK) population will include any subject who received study drug and also had a post-dose PK sample drawn.

In the ITT, MITT, and PP populations, treatment group will be defined as the randomization group of the subject. For the Safety and PK populations, subjects will be included in the group based on the treatment received.

The Imaging MITT population will include all randomized subjects who took at least one dose of study drug and have a Baseline and at least one post-Baseline MRI imaging measurement of adequate imaging quality.

10.3.2. Efficacy Analysis

10.3.2.1. Hypotheses

The study has co-primary endpoints for purposes of demonstrating clinical efficacy in the treatment of Alzheimer's disease. The co-primary endpoints are ADAS-cog_{11} and ADCS-ADL_{23}. Both clinical endpoints must reach significance for LMTM 200 mg/day to be designated as superior to placebo.

There are therefore two primary null hypotheses to consider:

- H_{01}: There is no difference between LMTM 200 mg/day and placebo in change from baseline ADAS-cog_{11} at Week 78 (Week 65 if fewer than 60% of subjects are retained in either treatment arm at Week 78)
- H_{02}: There is no difference between LMTM 200 mg/day and placebo in ADCS-ADL_{23} at Week 78 (Week 65 if fewer than 60% of subjects are retained in either treatment arm at Week 78)

For LMTM 200 mg/day to be designated as superior to placebo, both H_{01} and H_{02} need to be rejected.

The study is also designed to evaluate the potential for disease modification using change from Baseline in WBV as measured by the BBSI. Inferential analysis will be undertaken if clinical efficacy is demonstrated on the ADAS-cog_{11} scale at Week 78 (or Week 65 if fewer than 60% of subjects are retained in either treatment arm at Week 78). The following is the additional null hypothesis:

- H_{03}: There is no difference between LMTM 200 mg/day and placebo in reduction in brain atrophy at Week 78 (or Week 65 if fewer than 60% of subjects are retained in either treatment arm at Week 78).

10.3.2.2. Multiple Comparisons and Multiplicity

No adjustment for multiplicity is needed. Both co-primary endpoints must reach significance for LMTM 200 mg/day to be designated as superior to placebo. In addition, a pre-specified fixed sequence of testing will be used for the co-primary and secondary clinical efficacy endpoints. The fixed sequence of testing will be:

- ADAS-cog_{11}

- ADCS-ADL$_{23}$
- Brain MRI to evaluate change in WBV, to support a disease modification claim
- ADCS-CGIC
- MMSE
- MADRS
- NPI

Further imaging results will be analyzed for inferential purposes to support a disease modification claim if statistical significance on the ADAS-cog$_{11}$ scale is reached. The fixed sequence of testing will be:

- Brain MRI to evaluate change in ventricular volume
- FDG-PET temporal lobe averaged bilaterally
- Brain MRI to evaluate change in hippocampal volume

Only the MRI WBV endpoint will be required to establish disease modification.

10.3.2.3. Primary Clinical Efficacy Analysis

The primary analyses will be based on the MITT population.

Change from Baseline in ADAS-cog$_{11}$ and ADCS-ADL$_{23}$ will be analyzed using restricted maximum likelihood based repeated measures linear mixed models with an unstructured covariance matrix. For each endpoint, the model will include fixed effects for treatment group (two levels), time (six levels: corresponding to Weeks 13, 26, 39, 52, 65, and 78), the treatment group by time interaction, AChEI and/or memantine status at randomization (two levels: current ongoing use or not ongoing use) and geographic region (two levels: Americas, Europe/Australia). In addition, the corresponding Baseline values (ADAS-cog$_{11}$ or ADCS-ADL$_{23}$) will be included as a covariate[14]. CDR is a stratification variable but only to ensure a balanced design for some exploratory analyses and is not a covariate in the primary or secondary analyses. The Kenward and Roger method of calculating the denominator degrees of freedom will be used for the tests of fixed effects. Treatment comparisons will be based on the modelled change from Baseline at Week 78 (or Week 65 if fewer than 60% of subjects are retained in either treatment arm at Week 78). LMTM will be compared to placebo at an overall two-sided alpha of 0.05. All available post-Baseline data will be included in the model, with no imputation for assessments for which ADAS-cog$_{11}$ or ADCS-ADL$_{23}$ is not available; the handling of missing ADAS-cog$_{11}$ or ADCS-ADL$_{23}$ items is discussed in Section 10.3.2.7.

10.3.2.4. Additional Analyses of Primary Endpoints

For the ADAS-cog$_{11}$ and ADCS-ADL$_{23}$, the primary repeated measures model for change from Baseline will also be used to compare the treatment groups at each scheduled visit. Marginal means (least-square means in SAS) for change from Baseline will be presented at each scheduled visit, for each treatment group (placebo and treated). Differences showing the effect of treatment

[14] Where subject severity has been misclassified at baseline, severity will be defined by severity allocation at randomization.

at each scheduled visit, along with 95% confidence intervals and p-values, will be provided for the LMTM treatment group *versus* placebo.

In view of the EMA (2008) guidelines, the treatment effect at Week 52 will be compared to the treatment effect at Week 78 in order to investigate a potential disease modifying effect. The difference in treatment effect between Week 78 and Week 52 normalized by the pooled standard errors will be compared to the quantiles of the standard Gaussian distribution. The p-value will be determined as difference from zero based on the cumulative standard Gaussian distribution. A statistically significant positive normalized difference would support the claim for a treatment effect on rate of disease progression (delay of disability).

If there is a statistically significant effect on the rate of disease progression, a simple linear model of the mean treatment effects and placebo declines (output of the linear mixed effects model) will be generated in order to provide estimates of rates of decline and treatment effect on rate of decline.

Additionally, the statistical significance of the effect of treatment on the rate of decline will be tested using a linear mixed effects model of ADAS-cog_{11} and ADCS-ADL_{23} scores that includes fixed effects for treatment group, time, and the interaction between treatment group and time (where time will be treated as quantitative, *i.e.*, the number of nominal weeks from Baseline). Tests of the significance of inclusion of cubic terms of the time effects for both time treated as an ordered categorical variable as before and time treated as a continuous variable (number of nominal weeks from Baseline) will be carried out. If the cubic terms are non-significant, a reduced model will be fit in which the time effects are parameterized as quadratic functions.

For the ADAS-cog_{11} and ADCS-ADL_{23}, additional sensitivity analyses will be carried out using analysis of covariance (ANCOVA) models. The dependent variable will be change from Baseline to Week 78. The model will include geographic region (two levels: Americas, Europe/Australia), AChEI and/or memantine status at randomization (two levels: current ongoing use or not ongoing use), and the corresponding Baseline value. The difference between the marginal means (least-square means in SAS), with 95% confidence interval and p-value, will be provided for the LMTM treatment group *versus* placebo. For the ANCOVA analysis, missing values will be imputed by "last z-score carried forward" (LZCF) imputation, as follows. First, from all observations of MITT subjects, the mean and standard deviation of change from Baseline will be computed for each combination of treatment arm and visit, without imputation. A z-score for each subject at each visit will be computed, defined by $z=(x-m)/s$ where x is the subject's change from Baseline, and m and s are the mean and standard deviation for that subject's treatment arm and that visit, computed earlier. Missing observations are imputed by $x=m+z*s$, where z is the carried-forward z-score, and m and s are the mean and standard deviation for the subject's treatment arm at the visit to be imputed, computed earlier.

Similar analyses will be carried out using the MITT population with Imputation Using Drop-out Reason. For example, the LZCF imputation will be applied for subjects who discontinue due to lack of efficacy / disease progression; for subjects who discontinue for any other reason, the last available observation will be carried forward. Other imputation approaches will be specified in the SAP. In addition, multiple imputation methodology will be used for additional sensitivity analyses.

Because the occurrence of missing data could potentially lead to bias, strenuous efforts will be made to obtain all scheduled efficacy assessments from all randomized subjects, regardless of

whether or not they remain on study treatment. In addition, multiple sensitivity analyses will be completed in order to assess the results of the pre-specified primary analyses. These sensitivity analyses will be described in greater detail in the SAP, and are outlined below.

- The co-primary efficacy analysis will be repeated using the PP population and the ITT population.
- A responder analysis will be performed for ADAS-cog_{11}. A responder will be defined as a subject who has not worsened by more than the mid-point between the marginal means of the linear mixed effects model observed in the placebo and treatment arm on the ADAS-cog_{11} scale at the final visit (Week 78 or Week 65 if fewer than 60% of subjects are retained in either treatment arm at Week 78). Subjects who do not have a final ADAS-cog_{11} score will be classified as non-responders. The proportion of responders will be compared for LMTM 200 mg/day *versus* placebo using the Mantel-Haenszel test adjusting for the randomization strata as defined above. The odds ratio and its 95% confidence interval will be presented. This approach may also be used to analyze additional responder criteria based on the ADAS-cog_{11}. Other thresholds for defining a responder may also be considered.
- Time to decline in test score or inability to complete test will be analyzed using a Kaplan-Meier approach, with onset of decline as the first of two consecutive measurements that are worse than the baseline score by at least 2 units or when an ADAS-cog_{11} score cannot be obtained. The two treatment groups will be compared using the log-rank test.
- In addition, multiple imputation methodology will be used for additional sensitivity analyses.

Additional exploratory analyses of ADCS-ADL_{23} will include separate analyses of treatment effects on Instrumental-ADL (IADL) and Basic-ADL (BADL) as defined by Kahle-Wrobleski *et al*. (2014). Further analyses will be undertaken with particular reference to IADL-Factor 1 items (Global Instrumental) and Basic I and Basic II items as defined by Kahle-Wrobleski *et al*. Of particular clinical interest will be analysis of the effect of treatment on time to loss of 3 IADL items or 1 BADL item overall.

To quantify the effect of exposure to study drug on the treatment effect, an ANCOVA will be carried out. The dependent variable will be change from Baseline to Week 78. The model will include geographic region (two levels: the Americas and Europe/Australia), AChEI and/or memantine status at randomization (two levels: current ongoing use or not ongoing use), the corresponding baseline value, and the following categories for the duration of exposure to study drug based on total duration of exposure, inclusive of interruptions, and alternative categories defined on the basis of total drug exposure in (kilo)grams:

Category of Exposure (Days)
1
2-28
29-92
93-183
184-365
>365

10.3.2.5. Analyses for Disease Modification

Change in whole brain volume (WBV) as measured by the BBSI will be evaluated to demonstrate a potential disease modification effect in an Imaging MITT population which will include all randomized subjects who took at least one dose of study drug and have a Baseline and at least one post-Baseline MRI imaging measurement of adequate imaging quality. The change from Baseline to Week 78 (Week 65 if fewer than 60% of subjects are retained in either treatment arm at Week 78) in WBV using BBSI will be analyzed using a restricted maximum likelihood based repeated measures linear mixed model with an unstructured covariance matrix. The model will include fixed effects for treatment group (two levels), time (six levels: corresponding to Weeks 13, 26, 39, 52, 65, and 78) and the treatment group by time interaction. In addition, Baseline brain volume will be included as a covariate. The Kenward and Roger method of calculating the denominator degrees of freedom will be used for the tests of fixed effects. Treatment comparisons will be based on the modelled change from Baseline at Week 78 (or Week 65 if fewer than 60% of subjects are retained in either treatment arm at Week 78). LMTM will be compared to placebo at an overall two-sided alpha of 0.05. If this is statistically significant, a disease-modifying claim is supported by the analysis. All available post-Baseline data will be included in the model.

If there is a significant effect of treatment on WBV, the following exploratory analysis (not part of the secondary analysis hierachy) will be conducted. Pearson's chi-squared test will be used to assess the association between ADAS-cog_{11} change and WBV change at Week 78 (or Week 65 if fewer than 60% of subjects are retained in either treatment arm at Week 78) in the Imaging MITT population. As the thresholds cannot be defined *a priori*, they will be defined as follows: an ADAS-cog_{11} threshold for comparison of WBV change will be defined as the mid-point between the marginal means for change from Baseline to Week 78 (or Week 65 if fewer than 60% of subjects are retained in either treatment arm at Week 78) for the placebo and treated arms as determined in the primary efficacy analysis for ADAS-cog_{11} indicated above. Using this threshold, the Imaging MITT population will be split into the ADAS-cog_{11} "decliners" and "non-decliners" groups. Similarly, the WBV change will be split into two groups based on a threshold defined as the mid-point between the marginal means for change from Baseline to Week 78 (or Week 65 if fewer than 60% of subjects are retained in either treatment arm at Week 78) for the placebo and treated arms as determined in the efficacy analysis for WBV. Using this threshold the Imaging MITT population will be split into WBV "decliners" and "non-decliners" groups. The same analysis will also be carried out using only subjects in the treated group.

In order to answer the question whether change in WBV at Week 52 is associated with clinical non-decliner status at Week 78 (Week 65 if fewer than 60% of subjects are retained in either treatment arm at Week 78), the same Pearson's chi-squared test analysis as mentioned above will be used. The WBV threshold will be defined as the mid-point between the marginal means for change from Baseline to Week 52. The ADAS-cog$_{11}$ threshold at Week 78 (Week 65 if fewer than 60% of subjects are retained in either treatment arm at Week 78) is defined as above. This analysis likewise will be carried out using only subjects in the treated group, following the same strategy as mentioned above.

As an exploratory analysis for disease modification (not part of the secondary analysis hierachy), Pearson's correlation coefficient and the non-parametric Spearman's correlation coefficient for ADAS-cog$_{11}$ change and WBV change at Week 78 (Week 65 if fewer than 60% of subjects are retained in either treatment arm at Week 78) will be estimated in the Imaging MITT population. The same analysis will also be carried out using only subjects in the treated group.

In order to answer the question whether change in WBV at Week 39 predicts the status at Week 78 (Week 65 if fewer than 60% of subjects are retained in either treatment arm at Week 78), the same correlation analyses as mentioned above will be performed for these weeks. This analysis likewise will be carried out using only subjects in the treated group, following the same strategy as mentioned above.

10.3.2.6. Secondary Efficacy Analysis

10.3.2.6.1. ADCS-CGIC

ADCS-CGIC scores at Week 78 (or Week 65 if fewer than 60% of subjects are retained in either treatment arm at Week 78) will be analyzed using the Cochran-Mantel-Haenszel mean score test, with modified ridit scores. The analysis will be stratified by geographic region (two levels: Americas, Europe/Australia) and AChEI and/or memantine status at randomization (two levels: current ongoing use or not ongoing use). For subjects with missing data at Week 78 (or Week 65, as described above), the last available ADCS-CGIC score will be carried forward. The treatment comparison with placebo will be assessed against a significance level of 0.05.

In order to assess the impact of missing data, multiple sensitivity analyses will be carried out, as follows:

- The analysis described above will be repeated using the data from Weeks 13, 26, 39, 52, and 65 (if not used for the primary analysis), with no imputation for missing data.
- The analysis at Week 78 (or Week 65) will be repeated with missing data replaced by the subject's worst (least favorable) value from the earlier time points.
- ADCS-CGIC results at Week 78 (or Week 65) will be dichotomized as subjects with and without moderate or marked decline. Subjects without moderate decline are those with scores of 1, 2, 3, 4, or 5. Subjects with missing data will be assigned to the "with moderate or marked decline" category. This dichotomized response will be analyzed using the Cochran-Mantel-Haenszel test, stratified by the same factors as described for the primary analysis.
- ADCS-CGIC results at Week 78 (or Week 65) will be dichotomized as subjects with and without any decline, *i.e.*, absence of any decline (scores of 1, 2, 3, 4) *versus* decline

(scores of 5, 6, 7). Subjects with missing data will be assigned to the "with decline" category. This dichotomized response will be analyzed using the Cochran-Mantel-Haenszel test, stratified by the same factors as described for the primary analysis.

Additional repeated measures analyses of the ADCS-CGIC dichotomized as described in each of the preceding two bullets (with and without moderate or marked decline, with and without any decline) will be carried out using the data from Weeks 13, 26, 39, 52, 65, and 78. A Generalized Estimating Equation (GEE) model using the logit link function, the binomial variance function, and the unstructured working correlation model will be fit. This model will include the same covariates as described for the primary analysis. All available data will be used, with no imputation for missing data.

10.3.2.6.2. MMSE, NPI, and MADRS

MMSE, NPI, and MADRS will all be analyzed using repeated measures models, as described for the primary analysis on ADAS-cog_{11}. Analyses will be performed on the MITT population and, if it differs by more than 10% in overall number, the PP population.

The sequence of inferential testing is specified in Section 10.3.2.2.

10.3.2.7. Missing Data

Efficacy data missing for an entire outcome scale or for the majority of the scale (as indicated below) will not be imputed for analysis. Instead, it will be assumed that the data are close to missing at random after accounting for the terms in the model. However, missing items within a scale may be imputed if some items of the scale are present. The handling of partial data for the efficacy endpoints will be described in the SAP.

Early termination efficacy assessments will be allocated to the next target week.

The primary statistical analyses evaluate both cognitive as well as functional changes in the treated compared to the placebo group. No imputations for assessments that have missing values will be performed in order to achieve a conservative estimate of treatment effect. The chosen linear mixed effects model with unstructured covariance matrix as well as the Cochran-Mantel-Haenszel test without imputations will be employed. They account only for those assessments for which values have been obtained; the blurring effect of treatment non-compliance and withdrawal is minimized by this approach. The unstructured covariance matrix in the linear mixed effects models approximates the lack of knowledge in the variance/covariance structure and therefore provides a superior estimator.

In an exploratory analysis the effects of missing data and biased withdrawal will be investigated further. Last observation carried forward approach for missing data in the ADAS-cog_{11} and ADCS-ADL_{23} (see Section 10.3.2.4) will be used. In an exploratory analysis, the following approach to impute numbers for ADAS-cog_{11} and ADCS-ADL_{23} will be tested. If at least two post-baseline assessments are available for a subject, a linear slope-wise imputation will be used with non-fixed intercept. The use of a linear model to approximate decline is supported by the large studies of Doraiswamy *et al.* (2001), Stern *et al.* (1999) and Salloway *et al*. (2014) in subjects with similar severity.The values will be imputed according to these linear model fits; further details will be provided in the SAP (see Section 10.3.2.4).

Models alternate to the primary model proposed will also be used to evaluate the impact of missing data on the primary endpoints. These will be described in the SAP.

10.3.2.8. Exploratory Efficacy Analyses

10.3.2.8.1. Brain MRI

The same analyses as those used for WBV will be performed on hippocampal and ventricular volumes.

10.3.2.8.2. FDG-PET

Effect of LMTM on temporal lobe FDG uptake (normalized to the cerebellum) will be evaluated as an exploratory endpoint in further support of disease modification. The change from Baseline to Week 78 on FDG-PET will be analyzed using a restricted maximum likelihood based repeated measures linear mixed model with an unstructured covariance matrix. The model will include fixed effects for treatment group (two levels), time (two levels: corresponding to Weeks 39 and 78) and the treatment group by time interaction. The Baseline value will be included as a covariate. The Kenward and Roger method of calculating the denominator degrees of freedom will be used for the tests of fixed effects. Treatment comparisons will be based on the modelled change from Baseline at Week 78 (or Week 65 if fewer than 60% of subjects are retained in either treatment arm at Week 78). LMTM will be compared to placebo at an overall two sided alpha of 0.05. All available post-Baseline data will be included in the model, with no imputation for assessments for which an FDG-PET scan is not available. The change from Baseline to Week 39 will be analyzed using the same type of model. For subjects who have a Week 39 scan but not a Week 78 scan, the change from Baseline to Week 39 measure will be used as the response variable in the Week 78 analysis.

In order to evaluate the association between change in FDG-PET and cognitive changes, Pearson's chi-squared test for ADAS-cog_{11} change and FDG uptake (normalized to the cerebellum) at Week 78 (Week 65 if fewer than 60% of subjects are retained in either treatment arm at Week 78) will be estimated in the MITT population. As the thresholds cannot be defined *a priori*, they will be defined as follows: an ADAS-cog_{11} threshold for comparison of change in FDG-PET will be defined as the mid-point between the marginal means for change from Baseline to Week 78 (Week 65 if fewer than 60% of subjects are retained in either treatment arm at Week 78) for the placebo and treated arms as determined in the primary efficacy analysis for ADAS-cog_{11} indicated above. Using this threshold, the population will be split into the ADAS-cog_{11} “decliners” and “non-decliners” groups. Similarly, the FDG-PET change will be split into two groups based on a threshold defined as the mid-point between the marginal means for change from Baseline to Week 78 (Week 65 if fewer than 60% of subjects are retained in either treatment arm at Week 78) for the placebo and treated arms as determined in the efficacy analysis for FDG-PET uptake. Using this threshold the Imaging MITT population will be split into FDG-PET “decliners” and “non-decliners” groups. The same analysis will also be carried out using only subjects in the treated group.

In order to answer the question whether change in FDG-PET at Week 39 is associated with clinical non-decliner status at Week 78 (Week 65 if fewer than 60% of subjects are retained in either treatment arm at Week 78), the same Pearson's chi-squared test analysis as mentioned above will be used. The FDG-PET change will be split into two groups based on a threshold defined as the mid-point between the marginal means for change from Baseline to Week 39. This analysis likewise will only be carried out using only subjects in the treated group.

Additional exploratory analyses may be described in the SAP.

10.3.2.8.3. Subgroup Analyses

Subgroup analyses also will be performed based on the MITT population. The relationship between key subject characteristics and treatment effect will be assessed by adding a term to the ADAS-cog$_{11}$ and ADCS-ADL$_{23}$ analysis models for these key subject characteristics, and then testing the treatment by characteristic interaction for significance. The same types of analyses will be carried out for ADCS-CGIC using logistic regression models with the dependent variable being the proportion of subjects without moderate or marked decline, or without any decline (as defined in Section 10.3.2.4). The subject characteristics that will be included in this type of analysis are the following:

- Male and female
- Age < 75 and ≥ 75 years
- Screening MMSE 20-23 and 24-26
- AChEI and/or memantine users and non-users at randomization (current ongoing use or not ongoing use)
- Use (ongoing or not) of medical food (*e.g.*, Axona, Souvenaid) or alternative pharmacotherapy or medical food for dementia (*e.g.*, Vitamin E, folate [in doses up to 5 mg/day; use of doses of 1 mg/day as supplementation is not included here], a specific neurocognitive vitamin formulation [such as NeuroVits comprising 20 mg Vitamin B_6, 1 mg Vitamin B_{12}, 0.8 mg folate (see Douaud *et al.*, 2013)], ginkgo biloba, hormone replacement therapy, coconut oil, curcumin)
- Renal function (as defined by creatinine clearance ≥ 50 and < 50 mL/min)
- Region (the Americas or Europe/Australia) as well as a variable combining language and region (three levels: Americas, Europe/Australia English-speaking, and Europe/Australia non-English-speaking)
- ApoE genotype (presence or absence of the E4 allele)

Age will also be added as a quantitative variable to test whether treatment effect differs linearly with baseline age.

The ADAS-cog$_{11}$, ADCS-ADL$_{23}$, and ADCS-CGIC analyses will be repeated for subgroups in order to provide descriptive statistics to support any treatment by subject characteristic effects.

The relationship between severity at baseline as defined by CDR or MMSE and treatment response on the ADAS-cog$_{11}$ will be investigated by assessing the severity by treatment interaction effect in an additional model similar to the primary analysis model. Severity at Baseline will be defined by screening CDR (0.5 or 1) and also MMSE 20-23 or 24-26. In addition, the actual baseline MMSE score and screening CDR total score will also be tested in the ADAS-cog$_{11}$ model in place of the categorical severity based on CDR and MMSE, and the interaction between these baseline scores and treatment group will be assessed to determine whether there is a differing effect for subjects with differing baseline severity as indicated by higher or lower MMSE and CDR total scores.

Additional subgroup analyses may be defined in the SAP.

10.3.2.9. Withdrawal / Rebound

To analyze withdrawal and rebound effect, for each subject, the ADAS-cog_{11}, ADCS-ADL_{23}, and ADCS-CGIC change from the final on-treatment visit at Week 78 (or Week 65 if fewer than 60% of subjects are retained in either treatment arm at Week 78) to the 4-week post-treatment visit will be computed for those subjects in whom 4-week post-treatment visit data exists. Summary statistics will be provided within each treatment group and the paired t-test will be used to assess whether the mean change from the end of treatment to the 4-week post-treatment visit is equal to zero, for ADAS-cog_{11}, ADCS-ADL_{23} and MMSE. For ADCS-CGIC, the Wilcoxon signed-rank test will be used.

To investigate the potential bias of this analysis towards subjects who complete the study, the analysis is repeated for all subjects in whom a post final treatment visit exists, irrespective of when it occurs. The change in ADAS-cog_{11}, ADCS-ADL_{23} and MMSE will be normalized to a 4-week effect assuming a linear change after final on-treatment visit. For ADCS-CGIC, the score of the post-treatment visit will be used irrespective of when it occurs.

In addition, subjects with significant worsening will be individually reviewed (to be identified by medical review prior to database lock and unblinding).

10.3.3. Baseline Characteristics and Concomitant Medications

Tabular summaries of demographics, Baseline characteristics, and concomitant medication use will be prepared based on data collected at the Baseline visit (or, if not available, from the Screening visit).

Medical history will be recorded using the Medical Dictionary for Regulatory Activities (MedDRA) version 15.0 or later. Tabular summaries will be prepared of the numbers and percentages of subjects with abnormalities in a given System Organ Classification (SOC). All data, including study eligibility and Screening data, will be listed.

Concomitant medications and recently used medications (anti-dementia and psychiatric drugs used within the prior 3 months) will be coded using the March 2012 (or later) version of the World Health Organization (WHO) drug dictionary. Tabulations will be prepared of all drugs used concomitantly (relative to the first dose of study drug) based on WHO Anatomical Therapeutic Classification (ATC) level 1 term, ATC level 3 term and Preferred Term (generic name) with frequency and percentage of subjects in each dosing arm using each concomitant medication. Separate tabulation will be made of on-treatment changes in psychiatric medications (to be identified by blinded review), together with the reason(s) for such change.

Concomitant medications (inclusive of medical foods, alternative pharmacotherapies and nutritional/vitamin supplements used for dementia) are to be listed with these elements as well as the verbatim drug name. For subjects on digoxin, available plasma concentrations of digoxin obtained from the local laboratory are to be included.

10.3.4. Dosing and Exposure

Total duration of exposure and mean and modal daily dose per subject (including "dose equivalent" for subjects randomized to placebo), will be summarized descriptively by treatment group. Mean, modal, and maximum dose will also be summarized over selected exposure intervals. In addition, tabular summaries of the proportions of subjects with dose interruptions and dose reductions will be prepared for each treatment group.

Listings will encompass dosing, drug accountability, and compliance (percentage of tablets taken relative to intended number).

10.3.5. Safety Analysis

Safety results will be summarized using the safety population. The analysis of safety will include summaries of AEs, laboratory tests of blood and urine, pulse co-oximetry testing, vital signs and weight, ECGs, physical and neurological examinations, suicidality assessment, serotonin toxicity assessments, and cases identified with ARIA.

10.3.5.1. Adverse Events

Adverse events will be coded to a System Organ Class (SOC) and preferred term using MedDRA (version 15.0 or later). Adverse events will be regarded as treatment-emergent (TEAE) if they start on or after the first dose of study drug administration or if they were present prior to the first date of study drug administration and increased in severity or relationship to study drug while on study treatment. Those that resolved prior to the first dose or were ongoing at the same intensity and relationship will be regarded as pre-treatment AEs.

Tabular summaries are described below, with the number and percentage of subjects reporting each type of event presented by treatment group. If a subject reports the same preferred term more than once, it is counted only once within that category. Further, for a given tabulation, the preferred term will only be counted once in its worst severity, greatest relationship to treatment, and worst action taken.

Pre-treatment AEs, those present from the time of consenting until the first date of study drug administration, will be presented separately.

An overall summary table of TEAEs will be produced showing the number and percent of subjects in each treatment group with the following: TEAE, severe TEAE, TEAE related to study drug, serious TEAE, TEAE with outcome of death, and TEAE leading to interruption, dose reduction, or discontinuation from the study. In addition, the summary table will include the number of TEAEs, severe TEAEs, TEAEs related to study drug, and serious TEAEs.

Separate summaries of incidences (number and percentage of subjects) of all individual TEAEs and the subsets of drug-related TEAEs, TEAEs that are severe in intensity, serious TEAEs, and TEAEs leading to change in dose will be provided.

Certain TEAEs, identified by a blinded data review, will be grouped and summarized. These will include gastrointestinal and urinary tract events, falls, rashes, and possible serotonin syndrome. Details are to be defined in the SAP.

The subset of TEAEs that have an onset, increase in severity, or increase in relationship to study drug more than 7 days after the last dose of study drug will be considered post-treatment. An overall summary table of the number and percent of subjects in each treatment group with post-treatment AEs will be prepared.

Subgroup analyses will be performed in which the effects of demographics (sex, age, race/ethnicity) on selected group events will be evaluated. Other subgroups of interest may also be identified.

Interactions between MT and selected concomitant medications will be investigated for selected grouped events using Cox’s proportional hazard models.

All AEs will be presented in a data listing. In addition, listings also will be provided for SAEs, AEs leading to death, AEs leading to discontinuation of study drug, AEs leading to dose reduction and/or interruption, and any other AEs that are of special interest (to be defined prior to database lock).

10.3.5.2. Laboratory Tests of Blood and Urine

Descriptive statistics will be based on central laboratory results as described below. Laboratory results that are obtained from laboratories other than the central laboratory will not be included in tabular summaries. They will, however, be listed separately. Normal ranges will be provided by the central laboratory and each local laboratory used for testing parameters other than Heinz bodies.

The central laboratory will calculate and report creatinine clearance using the Cockcroft-Gault equation. Estimated glomerular filtration rate (eGFR) will be calculated based on the Modification of Diet in Renal Disease (MDRD) Study, to be further described in the SAP.

Categorical laboratory parameters will be summarized by treatment group for each target week using counts and percent of subjects in each result category. Continuous laboratory parameters will be summarized by treatment group for each target week using descriptive statistics for both the original values and the change from Baseline. Visit windows for each target week will be used (see SAP for details). For each analyte, Baseline values will be restricted to those subjects in the safety population for whom there is at least one post-Baseline value.

Selected parameters for which normal ranges differ may additionally be summarized by gender, age, and country (or region).

Box and whisker plots will be presented for selected parameters including hemoglobin, methemoglobin, reticulocytes, neutrophils, and liver function tests.

Shift tables will be provided cross tabulating the number of subjects who are low, normal, or high at Baseline (with respect to the normal range) against results at each target week.

Potentially clinically significant ranges will be defined in the SAP for selected parameters, and the number and percent of subjects meeting these criteria summarized. Tabular summaries will include only those subjects in whom the values represent a treatment-emergent worsening.

Listings of laboratory parameters will be presented. Listings will flag results above and below the normal range as well as those that meet criteria for being potentially clinically significant (whether or not a treatment-emergent worsening). Separate listings for each hematology, chemistry, and urinalysis parameter will show results that meet criteria for being potentially clinically significant. In addition, for subjects who have one or more results that meet criteria, all laboratory results will be displayed.

10.3.5.3. Vital Signs and Weight

Blood pressure, pulse, oral temperature, respiratory rate, and weight will be summarized by treatment group for each target visit using descriptive statistics for both the original values and the change from Baseline. Windows for each target visit will be used (see SAP for details).

Potentially clinically significant vital sign changes will be defined for selected parameters, and the number and percent of subjects meeting these criteria will be summarized by treatment group.

Listings of vital sign measurements will be presented; height will be included in the demographic listing as it is measured only once at Screening. Listings will flag results that meet criteria as being potentially clinically significant. Separate listings for each vital sign parameter will show results that meet criteria for being potentially clinically significant. In addition, for subjects who have one or more results that meet criteria, all corresponding vital sign results will be displayed.

10.3.5.4. Electrocardiogram

ECG results will be provided by the central ECG reader (inclusive of the Screening ECG). Tabular summaries and analyses are to be based on the central ECG data. Machine-read ECG results and interpretations from Screening and Baseline (Day -1) are to be recorded in the eCRF and included in a separate listing; this also applies to any subsequent local interpretations if used to make dosing or patient management decisions.

ECG interval data will be summarized for Screening and Day 1 pre- and post-dose measurements based on the average of the triplicate measures. All other study visit ECG data (interpretations and interval data based on single measurements [or means of triplicate measurements if performed for clinical reasons]) will be summarized separately. ECG interval and ventricular rate data will be summarized by treatment group using descriptive statistics for both the original values and the change from Baseline. Windows for each target visit will be used (additional details provided in the SAP). Parameters to be analyzed include heart rate (HR) and PR, QRS, QT, and corrected QT (using Fridericia's and Bazett's corrections), and RR intervals. Counts and percent of subjects in each result category will be tabulated. Overall interpretations of abnormality(ies) will also be tabulated, with subjects categorized by whether or not they have treatment-emergent abnormalities.

Subjects are also to be categorized and enumerated on the basis of QTc interval and change from Baseline (mean of triplicate Day 1 pre-dose measurements) as follows:

- QTcB/F outliers (in categories of > 450 to ≤ 480, > 480 to ≤ 500, > 500 msec)
- Change in QTcB/F outliers (in categories of > 30 to ≤ 60, > 60 to < 90, ≥ 90 msec)

All HR, interval data, and interpretations will be listed. Any new finding after Baseline (pre-dose on Day 1) will be categorized into diagnostic groups: rhythm, conduction, hypertrophy, arrhythmia, ischemia, infarction, other.

10.3.5.5. ARIA

At each scheduled time point, ARIA data will be summarized descriptively by treatment group. The proportions of subjects who meet case definitions for ARIA, *i.e.*, vasogenic edema, macrohemorrhages, microhemorrhages, or an area of superficial siderosis, will be tabulated by treatment group. Likewise, the number of microhemorrhages will be tabulated by treatment group.

All identified cases of ARIA will be listed, including those identified by the site. All recorded information will be included in listings.

10.3.5.6. Physical and Neurological Examinations

Pre-treatment physical and neurological examination results will be summarized by body system. Evaluations at subsequent visits will be summarized in a treatment-emergent fashion. Summaries will present the number and percentage of subjects with normal and abnormal observations by body system/parameter evaluated. By-subject listings will detail the abnormality(ies).

10.3.5.7. Serotonin Toxicity (Syndrome)

Ratings from the Serotonin Toxicity (Syndrome) Diagnostic Interview and Rating Guide (In-Clinic) and, in those subjects in whom it is applicable, the Serotonin Toxicity Telephone Assessment, will be listed. The total score for each of the four possible diagnostic criteria will be confirmed programmatically.

These data, together with adverse events, vital sign measurements, and physical and neurological examination results, will be reviewed to identify subjects with potential serotonin syndrome. This medical review will be performed prior to database lock and unblinding. The proportion of subjects in each treatment group thus identified will be summarized.

10.3.5.8. Columbia Classification Algorithm of Suicide Assessment (C-CASA)

Results of the C-SSRS will be evaluated and reported according to the C-CASA and listed. AE data will also be reviewed prior to database lock and unblinded to identify any additional potential cases.

10.3.6. Concentration of MT

Blood samples for the measurement of MT concentration (including time relative to prior dose) will be summarized using descriptive statistics at each scheduled collection visit. The statistics n (number of subjects with data), mean, median, standard deviation, minimum, maximum, geometric mean and coefficient of variation will be presented at each scheduled collection visit. PK summaries will be based on the PK population.

Separate summaries will also be presented for subjects with and without dose reductions.

Mean concentration will be plotted against time using linear axes.

The PK data will be modeled using a population pharmacokinetic approach. Parameters such as clearance (including weight-adjusted clearance) will be calculated and demographic, concomitant drug, and disease characteristics explored to identify any that are potentially significant covariates. The possibility of a concentration response relationship may also be investigated. This work will be reported separately to the clinical study report and will be subject to a separate analysis plan.

10.3.7. Pharmacoeconomics

A summary of the planned analyses is provided below; a detailed description of the planned analyses, as well as intended sensitivity analyses, will be provided in the SAP.

In order to investigate the pharmacoenconomic impact of LMTM, a cost consequence analysis (CCA) will be used. With a cost consequence analysis, a broad set of relevant outcomes (here the ADAS-cog$_{11}$, ADCS-ADL$_{23}$ and MMSE) will be tabulated and discussed in relation to costs (Mauskopf *et al.*, 1998). Costs and outcomes are presented separately.

This analysis has a risk of getting non-significant effects regarding costs as clinical trials evaluating efficacy are typically underpowered with respect to pharmacoeconomic results and long-term effects cannot be assessed due to time constraints in the clinical trials. Therefore a modelling approach will complement the analysis.

10.3.7.1. Resource Use

The Resource Utilization in Dementia instrument (RUD) is a comprehensive and validated instrument collecting data on resource use in trials, with the aim to calculate costs from a societal viewpoint. The RUD Lite questionnaire, a short version of RUD mainly discarding the resource use by the caregivers is employed here. All subjects, for whom RUD Lite data was collected at one time point at least, will be included in the summaries.

Together with costs of institutional care, the value of informal care is the most heavy cost driver in dementia care. Three components of caregiver time will be included in the analysis:

- time spent on toilet visits, eating, dressing, grooming, walking and bathing;
- time spent on shopping, food preparation, housekeeping, laundry, transportation, taking medication and managing financial matters; and
- time spent on supervising the subject.

Each component of caregiver time will be calculated as the hours of care on a typical day multiplied by days spent on providing these services. The caregiver time will be summarized for each treatment group by visit and with AChEI and/or memantine status at randomization, severity, and geographic region.

Caregiver work status, whether the subject was admitted to a hospital, whether the subject received services in a hospital emergency room, whether the subject visited any health care professional, and whether the subject received any nursing services will be summarized likewise.

Caregiver relationship with subject and the subject's living accommodations will be summarized descriptively.

10.3.7.2. Unit Costs

Costs will be calculated as the multiplication of the amount of units of resource use and the unit cost for this particular resource. Unit costs for each unit will be collected for each country (if unit cost data are not available for each trial country, then unit cost data for the dominant country only will be used). An average wage will be used as a proxy for the opportunity cost of informal care. Caregiver time for retired carers will be given a value of 35% of caregivers of working age (Johannesson *et al*. 1991).

10.3.7.3. Statistical Analysis

In order to statistically support the descriptive analysis, regression models will be employed. Generalized linear models assuming gamma distributed dependent variables will be used accounting for the skewness of resource use and cost data. Bootstrapping methods will complement the analysis by providing estimates for the confidence intervals (Ramsey *et al*., 2005) in the univariate analyses.

If there is a substantial amount of missing data on resource use (>20%), multiple imputation approaches will be tested.

10.3.7.4. Economic Modelling

As the long term cost-effectiveness of LMTM beyond the trial periods is of great interest, economic evaluations based on various modelling techniques will be used. These models most often have a cost utility analysis (CUA) design in which the relation between costs and outcomes

is expressed as the incremental cost effectiveness ratio (ICER). The quality adjusted life years (QALY) concept is the most frequently used outcome. However, EQ5D data are not collected in this study, only in the open-label extension (Study TRx-237-020). Therefore, external data calibrating EQ5D for ADAS-cog$_{11}$, ADCS-ADL$_{23}$ and MMSE will be used to estimate the implied impact of treatment on QALY. External data for disease progression and mortality as well as data on resource use and costs beyond the trial period will also be used as inputs. From the trial, data on efficacy are used as an empirical core for the model. Within trial data on resource use and costs as well as the efficacy data and derived estimated QALY data will be used to calibrate the external sources in the model. Often-used modelling techniques such as Markov models (Sonnenberg and Leventhal in Wimo *et al*., 1998) and Discrete Event Simulations (DES) (Guo *et al*., 2014) will be tested.

10.3.8. Cerebrospinal Fluid Biomarkers

For subjects who provide CSF samples, levels of CSF biomarkers total tau, phospho-tau, and Aβ_{1-42}, will be summarized descriptively by treatment group at Baseline and Week 78. Change from Baseline to Week 78 will also be summarized. In addition to these three biomarkers, the ratios total tau:Aβ_{1-42}, phospho-tau:Aβ_{1-42}, and phospho-tau:total tau will be derived and summarized.

Change from Baseline at Week 78 will be analyzed for each biomarker and ratio using analysis of covariance (ANCOVA). Treatment, region (two levels: the Americas or Europe/Australia), screening CDR (0.5 or 1), and AChEI and/or memantine status (two levels: current ongoing use or not ongoing use) at randomization will be included as factors, with corresponding Baseline value as a covariate. Treatment differences between the LMTM group and placebo will be presented along with 95% confidence intervals and p-values.

Note, results from early termination visits will be included in the Week 78 summaries and analyses.

Biomarker summaries and analyses will be based on the MITT population.

10.3.9. Genotyping

Genotyping results will be listed and summarized descriptively for each treatment.

10.4. Interim Analyses

No interim futility or efficacy analysis is planned in which treatment groups will be compared.

Recruitment, pooled efficacy data, and discontinuations will, however, be continuously monitored in a blinded fashion and projections on future dropouts calculated in order to check whether the 40% withdrawal assumption from the sample size calculation holds. Should the overall dropout for the duration of the study be projected to exceed 40%, then the number of subjects to be enrolled may be increased in order to power an analysis at 65 or 78 weeks; see Section 10.2.

As discussed in Section 10.2, the rate of placebo decline and the associated SD are the two critical parameters in study design and estimating sample size. In the course of recruitment to the study, it has been observed that there is approximately a 2:1 ratio of CDR questionable (CDR 0.5) to CDR mild (CDR 1.0) subjects at Baseline, whereas the initial sample size calculation assumed a predominance of mild subjects by CDR criteria. Based on Phase 2 data, the magnitude of the effect size appears to be proportional to placebo decline. The Baseline mean and standard

errors for ADAS-cog scores for CDR 0.5 and CDR 1.0 are respectively 15.82 (± 0.32) and 19.93 (± 0.52). Based on historical data (Stern *et al*., 1994) the predicted decline at 18 months for the baseline ADAS-cog score corresponding to CDR 0.5 would be approximately half than that for the ADAS-cog score corresponding to CDR 1.0 at Baseline. Furthermore, the placebo decline in mild subjects seen in large trials reported recently (studies referenced in Section 10.2.1) has been approximately 63% of the decline of studies undertaken earlier (TauRx meta-analysis of all publically available placebo decline data). Taking both of these factors into account, the placebo decline in a 2:1 population is projected to be 3.91 ADAS-cog units at 18 months, smaller than the placebo decline of 4.15 previously projected. This reduces the estimated effect size from 2.16 to 2.03 ADAS-cog units, assuming a 52% reduction in the rate of decline. The number of subjects has therefore been increased from 700 to 800 in order to be able to detect the smaller effect size.

For the $ADCS\text{-}ADL_{23}$ co-primary outcome, the increase in the number of subjects to 800 provides at least 90% power for detecting a treatment difference of 3.23 units at a two-sided alpha of 0.05, under the assumption that the effect size corresponds to a 38% reduction in the expected rate of decline and assuming that the placebo decline is reduced to -8.50 ± 14.06 units rather than -9.21 ± 14.06 units as originally planned.

For the ADCS-CGIC secondary outcome, the increase in the number of subjects to 800 does not impact materially on the reduction in the percentage of subjects declining for 90% power to show a statistically significant difference at a two-sided alpha of 0.05, *i.e*., it remains approximately 69%, or a 14% reduction in the proportion of subjects worsening[15].

For the MRI disease-modifying outcome, the increase in the number of subjects to 800 provides an estimated 288 scan-pairs in each arm. Based on the use of a two-sided test at the 5% level of significance, there is 90% power to detect a scan effect of +3,311 mm^3 or a 16% reduction in rate of disease progression relative to that expected in the control arm at 18 months.

11. REGULATORY

Investigators and all other parties involved in the conduct of the study are responsible for ensuring that the study is conducted at their sites in accordance with the approved protocol and with the principles of the Declaration of Helsinki (most current applicable version), the International Conference on Harmonisation (ICH) Guidelines for Good Clinical Practice (GCP) (CPMP/ICH/135/95, July 1996), and with applicable country and local regulatory requirements and laws.

The Sponsor will be responsible for ensuring that the relevant approval is obtained from the local regulatory authority prior to the start of the study. The relevant documents will be provided to the investigator. The Sponsor or designee will forward any protocol amendments to the regulatory authority and will ensure that SAEs are reported, and progress reports and details of any serious protocol violations are provided as required.

The regulatory authority will be informed should the study be terminated early.

[15] The estimate of power is based on a simple comparison of placebo *versus* a single treated group.

12. APPROVAL OF THE PROTOCOL AND AMENDMENTS

Following authorization by the Sponsor, the final protocol and all related documents (*e.g.*, information sheets and ICFs) will be submitted to the IEC/IRB.

The Sponsor will be responsible for ensuring that regulatory and IEC/IRB approvals are obtained prior to the start of the study. The relevant documents will be provided to the investigator.

Neither the investigator nor the Sponsor will modify this protocol. If modification is necessary, either party must first obtain the concurrence of the other. The party initiating a modification will confirm it in writing, and the investigator will be responsible for informing the IEC/IRB. In case of a substantial amendment, prior approval of the IEC/IRB is required.

TauRx or designee is responsible for submission of a protocol amendment to the regulatory authority. In the event of a substantial amendment, prior approval is required.

13. SERIOUS BREACHES

The investigator and all other parties involved in the conduct of the study will comply with the protocol and ICH GCP. All deviations will be reported to the study monitor.

It is the responsibility of the Sponsor to notify the licensing authority of any serious breach which is likely to affect, to a significant degree, the safety or mental integrity of the subjects of the study or the scientific value of the study.

All serious breaches will be notified to the pertinent regulatory authorities according to the relevant national regulatory requirement. The reporting will be *via* the Sponsor in accordance with TauRx SOPs.

14. INFORMED CONSENT

It is the responsibility of the investigator or a medically trained, medically qualified sub-investigator to obtain informed consent from each subject participating in this study, or his/her representative that is permitted to provide consent in accordance with local legislation. Where required by local law, the person who informs the subject must be a physician.

Subjects and/or their legal representative(s) must give written (signed and personally dated) informed consent prior to study entry and before any study specific procedures are undertaken; in Germany, the subject must be able to provide his or her own written informed consent (§40, section 1 of the German Drug Law [AMG]). The identified caregiver(s) for each subject also must provide written consent to his/her own participation as outlined below. Where there is a change of caregiver, the new caregiver must provide written informed consent.

Potential subjects will be assessed for whether they have capacity to understand the ICF and give consent.

Where possible, fully informed consent will be obtained from the subject. However, subjects entering this study will have mild dementia and many may lack the necessary mental capacity to give fully informed consent. If the potential subject is unable to comprehend the ICF, then one or more legally acceptable representatives will be required to sign the ICF as required by national

law. In this situation, and provided that it is permitted by local legislation, the subject’s agreement to participate in the study will still be obtained to his/her best level of understanding and recruitment will not proceed if the subject refuses or shows significant distress.

CSF samples will be collected at designated study sites only from subjects who are mentally capable of providing their own separate informed consent and specifically agree to have lumbar puncture performed (this is considered an optional assessment).

Informed consent can be obtained only after the aims, methods, anticipated benefits, and known potential hazards of the study have been explained to and discussed with the potential subject and caregiver by the investigator. A subject information sheet, providing a written summary of all relevant information, will be given to the potential subject and caregiver prior to written informed consent being obtained. The caregiver will also be given an information sheet. The information sheet will make clear that access to the subject’s medical records will be required. It is the responsibility of the investigator to ensure that the potential subject and/or caregiver are aware of this. The investigator will explain to the potential subject and caregiver that they are at liberty to refuse to take part in the study or, should they decide to participate, they may withdraw from the study at any time. Such a decision should not, in any way, impinge on the future management of the subject. The potential subject and caregiver will be allowed as much time as they need to decide whether or not to participate in the study and will be provided with a contact point where further information about the study may be obtained.

The study is being performed in the European Union and the United States, two geographic regions that maintain descriptions of clinical studies on the internet. As required by the U.S. Food and Drug Administration, the ICF must contain the following text: “A description of this clinical trial will be available on http://www.ClinicalTrials.gov, as required by U.S. Law. This Web site will not include information that can identify you. At most, the Web site will include a summary of the results. You can search this Web site at any time.” Consistent with European Union law, the ICF also indicates that information will be on https://www.clinicaltrialsregister.eu. Information regarding other national registries will also be included in the ICF, where applicable.

15. INVESTIGATOR RESPONSIBILITIES

The primary responsibility of all investigators participating in the study is for the well-being and interests of their subjects, including subjects enrolled in this study. The investigator has overall responsibility for the conduct of the trial at his/her study site and may delegate specific duties to appropriately trained members of his/her research team or to other hospital staff, *e.g.*, the pharmacy. Any delegation must be clearly documented in a study site specific delegation list.

The investigator is responsible for the following:

- Performing the study in accordance with ICH GCP
- Ensuring that adequate time and appropriate resources are available to perform the study as described in this protocol
- Ensuring that all persons assisting with the trial are adequately qualified, trained, and informed about the protocol, trial-related duties, and functions

- Maintaining a list of sub-investigators and other appropriately qualified persons to whom duties have been delegated
- Signing an Investigator Agreement to confirm acceptance and willingness to comply with the study protocol
- Maintaining adequate control of study drug and appropriate records of drug disposition
- Maintaining adequate records of each subject's participation

Where local laws require it, national regulatory requirements with regard to the inclusion of subjects who are unable to consent will be followed by the investigators. In particular, in Germany, the risk threshold and degree of burden/distress will be monitored constantly by the investigators in accordance with §41, section 3 of the German Drug Law (AMG).

TauRx and the appointed DSMB will constantly evaluate the risk threshold of this particular study by assessing the safety profile according to Sections 4.8 and 8, as well as assessing the safety profile of other ongoing studies with the same active moiety. Any changes in the risk profile during the course of the study will be communicated to the investigators. In addition, the investigators will review adverse events at each visit in accordance with the schedule of assessments (see Table 4-1), and have the right to reduce the dose, interrupt or discontinue study drug for safety reasons as described in Sections 5.3 and 6.3.

The burden/distress associated with participation in this study is addressed in the patient information leaflet. During the study, the investigators should obtain information from the subjects in order to adequately monitor the degree of burden/distress. Subjects are advised that they have the right to discontinue study drug and withdraw from the study at any time for any reason and should inform the investigators accordingly in order to assist the investigators with monitoring activities.

16. CONFIDENTIALITY AND DATA PROTECTION

All study-related documentation is confidential, whether obtained by the investigator or provided by TauRx or their representative.

The investigator must ensure the anonymity of subjects in the trial is maintained on eCRFs, samples, specimens, and other documents leaving the study site and submitted to TauRx or its designees. Subjects must NOT be identified by name, but by an identification code (usually trial number). For all subjects (including subjects who were screened but not enrolled), the investigator must keep a separate log of subject codes, names, and addresses.

To conform to the requirements of EU Directive 95/46/EC, subjects will be explicitly asked to consent to transmission of their data outside the European Economic Area. In the United States, data will be protected consistent with HIPAA.

Confidentiality of the records identifying the subject will be maintained. Representatives of the Sponsor such as monitor(s) or auditor(s), IRB/IEC, and pertinent regulatory authorities will be permitted direct access to these records and other source data/documents as appropriate.

Details of access to the subjects' data will be fully described within the subject information sheet. The consequence of the subject's withdrawal of consent with regards to the use of data will also be described.

17. QUALITY ASSURANCE AND CLINICAL MONITORING

Standard operating procedures (SOPs) will be adhered to for all activities relevant to the quality of the study, including protocol compliance, data collection, quality control, and data analyses and reporting.

All aspects of the study will be subject to a Quality Assurance (QA) audit plan. QA audits will be conducted on critical phases during the clinical and reporting phases of the study. These audits will be carried out by QA personnel, independent of the staff involved in the study, according to relevant SOPs.

Clinical monitoring, both primary and secondary, will be performed by trained clinical research personnel. Clinical monitoring is an integral part of controlling and securing of data integrity and subject safety. The first monitoring visit will be scheduled appropriately after the first subject is screened at a site depending on factors that could impact on data reliability, some of which are mentioned below. The average monitoring frequency will be described in a clinical monitoring plan and will depend on a number of factors including subject screening and recruitment rates, site performance, and quality adherence. Regulatory recommendations and guidelines will be followed. Detailed expected monitoring activity will be described in the clinical monitoring plan, which will be modified on an ongoing basis to ensure subject safety and data integrity.

The monitor will ensure compliance with the protocol, adherence to regulatory and ICH obligations, accurate reporting of AEs, maintenance of trial records including drug accountability records, and correct administration of study procedures including supply and storage of study materials. ICFs will be reviewed to verify that they are correctly signed and dated by the subject and caregiver and the investigator or medically trained sub-investigator. At each monitoring visit, subject data will be reviewed and verified against the medical records.

The monitor will require direct access to laboratory test results and other records needed to verify entries on the eCRF.

The investigator (or his/her designated deputy) agrees to cooperate with the monitor and other clinical research personnel to ensure that any problems detected in the course of these monitoring visits are quickly resolved.

Secondary monitoring of data and/or trial documentation may be carried out by or on behalf of the Sponsor at any stage. Audits of study sites and/or trial processes may be carried out at any stage.

18. DOCUMENTATION

The protocol, its amendments, and any other required documents must be submitted for appropriate regulatory review and approval.

The investigator at each study site must generate and maintain adequate records (medical records, source documents, and eCRFs) to enable the conduct of this study to be fully documented.

Initially, data will be collected on source documents which will then be transcribed to the eCRF. The eCRF may serve as the primary collection medium for any data (to be agreed with the investigator and documented in the Source Data Verification Agreement). Each enrolled subject must have an eCRF completed and this must be reviewed and approved by the investigator.

The documents specified by ICH GCP (*e.g.*, copies of protocols, CRF pages, original copies of test results, reports, drug dispensing logs, correspondence, records of informed consent, and other documents pertaining to the conduct of the study) must be kept on file by the investigator for a minimum of 15 years or for the period of time specified by local law for the preservation of hospital patient documents, whichever is the longest. No study documents should be destroyed without prior written agreement between TauRx and the investigator. Should the site wish to assign the study records to another party, or move them to another location, TauRx must be informed.

A record must be kept of all subjects consenting for the study and subsequently excluded. The reason for non-participation in the study should be recorded.

The following documents must be provided before or at site initiation.

- Protocol and amendments (if applicable) signed and dated by applicable Sponsor representatives, as well as by the investigator
- Regulatory approval (or in absence of document, evidence that study may proceed)
- Signed and dated IEC approval
- Approved subject information sheet, ICF, and advertisement for recruitment (if any)
- eCRFs
- Confidentiality agreement(s)
- Financial disclosure
- Study drug/shipping records
- Signed *curricula vitae* (CVs) for personnel who have signed the authorized delegation log (including principal investigator, all sub-investigators, and designated assistants)
- Authorized signature log/delegation list
- Investigator Brochure with signed and dated Investigator Brochure receipt
- Signed and dated clinical trial agreement
- Research and development (or institution) approval, if applicable
- Signed and dated indemnity/insurance statement (if applicable)
- Laboratory reference ranges and accreditation for all applicable laboratories (central and local, as applicable)
- Pharmacy agreement (if any)
- Instructions for handling investigational product

- Sample label
- SAE forms

19. PUBLICATION

Since this is a multiple site study, the community of investigators and delegated individual investigators shall not publish any partial results before the end of the study or before the analysis and publication of the results of the entire study.

The investigator and/or institution shall have the right to publish, display, or otherwise communicate orally, in writing, or electronically (hereafter a “publication”) the results of their work conducted under this protocol after 12 months from NDA or equivalent filing, or earlier only with explicit consent of Sponsor in advance and in writing.

Sites and/or investigators must provide the Sponsor with the opportunity to review the contents of any proposed abstract or publication concerning the work, including any results of the study, in advance of publication, and agree to delay the publication if, in the Sponsor’s reasonable view, the publication may prejudice the Sponsor’s intellectual property. The Sponsor will make every reasonable effort to consider and release each proposed abstract or publication within 60 days of submission. The investigator and/or site will include where possible comments made by the Sponsor. Authorship will be determined by mutual agreement. Access to data will be in accordance with authorship.

20. INDEMNITY, INSURANCE AND COMPENSATION

A clinical trials insurance and product liability insurance policy will be in place to cover the conduct of this study.

21. ADMINISTRATIVE AND FINANCIAL AGREEMENT

Agreed costs for each participating study site will be met by TauRx. For each study site, an agreement will be prepared and signed off by the relevant authority on behalf of the institution (*e.g.*, National Health Service Trust, University) and by TauRx or its designee before the initiation of the trial. Each investigator and subinvestigator must also sign a Form FDA 3455 or its equivalent to disclose any financial arrangements or interests.

Subjects will be reimbursed by TauRx, through the investigator, for reasonable travel costs to and from the study site and accommodation in certain circumstances by prior agreement with the Sponsor.

22. STUDY ADMINISTRATION

This trial will be conducted in compliance with ICH GCP and other applicable regulatory requirements.

Contract Research Organizations and/or independent contract personnel will be contracted to manage and monitor the trial, to provide services for data management and statistical analysis, to provide regulatory advice and services, to handle the reporting of Serious Adverse Events, to provide services for laboratory and PK analysis, to package and distribute the clinical trial supplies, and to provide quality assurance support and services.

Calibration certification for the following equipment used to generate study data will be confirmed: ECG machines, pulse co-oximeters, pharmacy temperature loggers, and pipettes.

23. REFERENCES

AD2000 Collaborative Group. Long-term donepezil treatment in 565 patients with Alzheimer's disease (AD2000): randomised double-blind trial. Lancet 2004; 363:2105-15.

Aisen PS, Schafer KA, Grundman M, *et al*. Alzheimer's Disease Cooperative Study. Effects of rofecoxib or naproxen vs placebo on Alzheimer disease progression: a randomized controlled trial. JAMA 2003; 289(21):2819-26.

Aisen PS, Schneider LS, Sano M, *et al.* Alzheimer Disease Cooperative Study. High-dose B vitamin supplementation and cognitive decline in Alzheimer disease: a randomized controlled trial. JAMA 2008; 300(15):1774-83.

Allen VJ, Methven L, Gosney MA. Use of nutritional complete supplements in older adults with dementia: Systematic review and meta-analysis of clinical outcomes. J Clin Nutr 2013. http://dx.doi.org/10.1016/j.clnu.2013.03.015.

Baddeley TC, McCaffrey JM, Storey J, *et al.* Complex disposition of methylthioninium redox forms determines efficacy in tau aggregation inhibitor therapy for Alzheimer's disease. J Pharmacol Exp Ther 2015; 352:1-9.

Barker SJ, Curry J, Redford D, *et al.* Measurement of carboxyhemoglobin and methemoglobin by pulse oximetry: A human volunteer study. Anesthesiology 2006; 105(5):892-7.

Barnes J, Boyes RG, Lewis EB, *et al.* Automatic calculation of hippocampal atrophy rates using a hippocampal template and the boundary shift integral. Neurobiol Aging 2007; 28(11): 1657-63.

Berg L, Miller JP, Storandt M, *et al.* Mild senile dementia of the Alzheimer type: 2. Longitudinal assessment. Ann Neurol 1988; 23(5):477-84.

Bierer LM, Hof PR, Purohit DP, *et al.* Neocortical neurofibrillary tangles correlate with dementia severity in Alzheimer's disease. Arch Neurol 1995; 52(1):81-8.

Birks J. Cholinesterase inhibitors for Alzheimer's disease. Cochrane Database of Systematic Reviews, Issue 1, 2006. Article No. CD005593.

Birks JS, Harvey R. Donepezil for dementia due to Alzheimer's disease (Cochrane Review). In: The Cochrane Library, Issue 3, 2004. Oxford: Update Software Ltd.

Birks J, Grimley Evans J, Iakovidou V, *et al.* Rivastigmine for Alzheimer's disease (Cochrane Review). In: The Cochrane Library, Issue 3, 2004, Oxford: Update Software Ltd.

Bowie P, Branton T, Holmes J. Should the Mini Mental State Examination be used to monitor dementia treatments? Lancet 1999; 354(9189):1527-8.

Braak H, Braak E. Neuropathological stageing of Alzheimer-related changes. Acta Neuropathol 1991; 82(4): 239-59.

Bradley KM, Bydder GM, Budge MM, *et al*. Serial brain MRI at 3-6 month intervals as a surrogate marker for Alzheimer's disease. Br J Radiol 2002; 75(894):506-13.

Conclusions of a WHO Technical Consultation on folate and vitamin B_{12} deficiencies. Food Nutr Bull. The United Nations University, 2008;29(2 Suppl):S238-244.

Cummings JL. The Neuropsychiatric Inventory: Assessing psychopathology in dementia patients. Neurology 1997; 48 (5 Suppl 6):S10-S16.

Cummings JL, Mega M, Gray K, *et al*. The Neuropsychiatric Inventory: Comprehensive assessment of psychopathology in dementia. Neurology 1994;44(12):2308-14.

Doody RS, Thomas RG, Farlow M, *et al*. Phase 3 trials of solanezumab for mild-to-moderate Alzheimer's disease. N Engl J Med 2014; 370(4):311-21.

Doraiswamy PM, Kaiser L, Bieber F *et al*. The Alzheimer's Disease Assessment Scale: evaluation of psychometric properties and patterns of cognitive decline in multicenter clinical trials of mild to moderate Alzheimer's disease. Alzheimer Dis Assoc Disord 2001; 15(4):174-83.

Douaud G, Refsum H, de Jager CA, *et al*. Preventing Alzheimer’s disease-related gray matter atrophy by B-vitamin treatment. PNAS 2013; 110(23): 9523-9528.

Fazekas F, Chawluk JB, Alavi A, *et al*. MR signal abnormalities at 1.5 T in Alzheimer's dementia and normal aging. Am J Roentgenol 1987; 149(2):351-356.

Fillenbaum GG, Peterson B, Morris JC. Estimating the validity of the Clinical Dementia Rating Scale: the CERAD experience. Aging (Milano) 1996; 8(6):379-85.

Folstein MF, Folstein SE, McHugh PR. “Mini-mental State”: A practical method for grading the cognitive state of patients for the clinician. J Psychiatr Res 1975; 12(3):189-98.

Fox NC, Freeborough PA. Brain atrophy progression measured from registered serial MRI: Validation and application to Alzheimer’s disease. J Magn Reson Imaging 1997; 7(6):1069-75.

Freeborough PA, Fox NC. The boundary shift integral: an accurate and robust measure of cerebral volume changes from registered repeat MRI. IEEE Trans Med Imaging 1997; 16(5): 623-9.

Galasko D, Bennett D, Sano M, *et al.* An inventory to assess activities of daily living for clinical trials in Alzheimer's disease. The Alzheimer's Disease Cooperative Study. Alzheimer Dis Assoc Disord 1997; 11 Suppl 2:S33-9.

Galasko D, Schmitt F, Thomas R, *et al.* Detailed assessment of activities of daily living in moderate to severe Alzheimer's disease. J Int Neuropsychol Soc 2005; 11(4):446-53.

Gold M. Study design factors and patient demographics and their effect on the decline of placebo-treated subjects in randomized clinical trials in Alzheimer's disease. J Clin Psychiatry 2007; 68(3):430-8.

Gourlain H, Buneaux F, Borron SW, *et al*. Interference of methylene blue with CO-Oximetry of hemoglobin derivatives. Clin Chem 1997; 43(6 pt 1):1078-80.

Green RC, Schneider LS, Amato DA, *et al*. Effect of tarenflurbil on cognitive decline and activities of daily living in patients with mild Alzheimer disease: a randomized controlled trial. JAMA 2009; 302(23):2557-2564.

Guo S, Getsios D, Revankar N *et al*. Evaluating disease-modifying agents: a simulation framework for Alzheimer's disease. Pharmacoeconomics 2014; 32(11):1129-39.

Hughes CP, Berg L, Danziger WL, *et al.* A new clinical scale for the staging of dementia. Br J Psychiatry 1982; 140:566-72.

Izdes S, Altintas ND, Soykut C. Serotonin syndrome caused by administration of methylene blue to a patient receiving selective serotonin reuptake inhibitors. Anesth Analg 2014;2(9):111-112.

Jobst KA, Smith AD, Barker CS, *et al.* Association of atrophy of the medial temporal lobe with reduced blood flow in the posterior parietotemporal cortex in patients with a clinical and pathological diagnosis of Alzheimer's disease. J Neurol Neurosurg Psychiatry 1992; 55:190-4.

Johannesson M, Borqquist L, Jönsson B *et al*. The costs of treating hypertension--an analysis of different cut-off points. Health Policy 1991; 18(2):141-50.

Jonsson L. Assessing health economic outcome in Alzheimer’s disease clinical trials. J Nutr Health Aging 2007; 11(4):353-5.

Kahle-Wrobleski K, Coley N, Lepage B *et al*. Understanding the complexities of functional ability in Alzheimer's disease: more than just basic and instrumental factors. Current Alzheimer Research 2014; 11:357-366.

Kaufer DI, Cummings JL, Christine D. Effect of tacrine on behavioral symptoms in Alzheimer's disease: an open-label study. J Geriatr Psychiatry and Neurol 1996; 9(1): 1-6.

Malouf R, Grimley Evans J. Folic acid with or without vitamin B12 for the prevention and treatment of healthy elderly and demented people. Cochrane Database Syst Rev 2008; 4:CD004514.

Malouf R, Areosa Sastre A. Vitamin B12 for cognition. Cochrane Database Syst Rev 2003; 3:CD004326.

Matsuda H, Kitayama N, Ohnishi T, *et al*. Longitudinal evaluation of both morphologic and functional changes in the same individual with Alzheimer’s disease. J Nucl Med 2002; 43(3):304-11.

Mauskopf JA, Paul JE, Grant DM *et al*. The role of cost-consequence analysis in healthcare decision-making. Pharmacoeconomics 1998; 13(3): 277-88.

Montgomery SA, Asberg M. A new depression scale designed to be sensitive to change. Br J Psychiatry 1979; 134:382-389.

Moore E, Mander A, Ames D *et al*. Cognitive impairment and vitamin B12: a review. Int Psychogeriatr 2012; 24(4):541-556.

Morris JC, McKeel DW Jr, Fulling K, *et al.* Validation of clinical diagnostic criteria for Alzheimer's disease. Ann Neurol 1988; 24(1):17-22.

Morris JC, Ernesto C, Schafer K, *et al.* Clinical Dementia Rating training and reliability in multicenter studies: the Alzheimer's Disease Cooperative Study experience. Neurology 1997; 48(6):1508-10.

Morris JC, Cyrus PA, Orazem J, *et al*. Metrifonate benefits cognitive, behavioral and global function in patients with Alzheimer’s disease. Neurology 1998;50(5):1222-1230.

Mukaetova-Ladinska EB, Garcia-Siera F, Hurt J, *et al.* Staging of cytoskeletal and β-amyloid changes in human isocortex reveals biphasic synaptic protein response during progression of Alzheimer's disease. Am J Pathol 2000; 157(2):623-36.

Nachum-Biala Y, Troen AM. B-vitamins for neuroprotection: Narrowing the evidence gap. BioFactors 2012; 38(2):145-150.

National Institute for Clinical Excellence (NICE) Technology Appraisal Guidance No 19. Guidance for the use of donepezil, rivastigmine and galantamine for the treatment of Alzheimer's disease 2001, London: NICE.

O'Leary F, Allman-Farinelli M, Samman S. Vitamin B_{12} status, cognitive decline and dementia: A systematic review of prospective cohort studies. Br J Nutr 2012; 108(11):1948-1961.

Olin J, Schneider L. Galantamine for dementia due to Alzheimer's disease (Cochrane Review). In: The Cochrane Library, Issue 3, 2004, Oxford: Update Software, Ltd.

Ramsey S, Willke R, Briggs A *et al*. Good research practices for cost-effectiveness analysis alongside clinical trials: the ISPOR RCT-CEA Task Force report. Value Health 2005; 8(5):521-33.

Reisberg B, Doody R, Stöffler A, *et al.* Memantine in moderate-to-severe Alzheimer's disease. N Engl J Med 2003; 348(14):1333-41.

Rogers SL, Farlow MR, Doody RS, *et al.* A 24-week, double-blind, placebo-controlled trial of donepezil in patients with Alzheimer's disease. Neurology 1998; 50(1):136-45.

Rosen WG, Mohs RC, Davis KL. A new rating scale for Alzheimer's disease. Am J Psychiatry 1984; 141(11):1356-64.

Salloway S, Sperling R, Fox NC, *et al*. Two phase 3 trials of bapineuzumab in mild-to-moderate Alzheimer's disease. N Engl J Med 2014; 370(4): 322-33.

Sano M, Ernesto C, Thomas RG, *et al.* A controlled trial of selegiline, alpha-tocopherol, or both as treatment for Alzheimer's disease. N Engl J Med 1997; 336(17):1216-22.

Schneider LS. Assessing outcomes in Alzheimer disease. Alzheimer Dis Assoc Disord 2001; 15(suppl 1):s8-s18.

Schneider LS, Olin JT, Doody RS, *et al.* Validity and reliability of the Alzheimer's Disease Cooperative Study-Clinical Global Impression of Change. Alzheimer Dis Assoc Disord 1997; 11(Suppl 2):S22-32.

Sonnenberg FA, Leventhal EA. Modeling disease progression with Markov models.
In Wimo A *et al*. Health economics of dementia. Wiley 1998:171-96.

Stern Y, Albert SM, Sano M, *et al.* Assessing patient dependence in Alzheimer's disease. J Gerontol 1994; 49(5):M216-22.

Stern Y, Albert S, Tang MX, *et al.* Rate of memory decline in AD is related to education and occupation: cognitive reserve? Neurology 1999; 53(9):1942-7.

Tombaugh TN, McIntyre NJ. The Mini-Mental State Examination: A comprehensive review. J Am Geriatr Soc 1992; 40(9):922-35.

WHO. Serum and red blood cell folate concentrations for assessing folate status in populations. Vitamin and Mineral Nutrition Information System. Geneva: World Health Organization, 2012 (http://apps.who.int/iris/bitstream/10665/75584/1/WHO_NMH_NHD_EPG_12.1_eng.pdf, accessed 3 May 2013)

Wilcock GK, Black SE, Hendrix SB, *et al.* Efficacy and safety of tarenflurbil in mild to moderate Alzheimer's disease: a randomised phase II trial. Lancet Neurol 2008; 7(6):483-93. Erratum in: Lancet Neurol 2008; 7(7):575 and Lancet Neurol 2011;10(4):297.

Wimo A, Winblad B. Resource utilisation in dementia: RUD Lite. Brain Aging. 2003; 3:48-59.

Wimo A, Winblad B, Stöffler A, *et al.* Resource utilisation and cost analysis of memantine in patients with moderate to severe Alzheimer's disease. Pharmacoeconomics 2003; 21(5):327-40.

Wimo A, Wetterholm AL, Mastey V, *et al*. Evaluation of the resource utilization and caregiver time in Antidementia drug trials - a quantitative battery. In: The Health Economics of dementia. Eds. Wimo A, *et al*. 1998. London: John Wiley & Sons. 465-99.

Wimo A, Nordberg G. Validity and reliability of assessments of time. Comparisons of direct observations and estimates of time by the use of the resource utilization in dementia (RUD)-instrument. Arch Gerontol Geriatr 2007; 44:71-81.

Wischik CM, Lai RYK, Harrington CR. Modelling prion-like processing of tau protein in Alzheimer’s disease for pharmaceutical development. In: Brain Microtubule Associated Proteins: Modifications in Disease. Eds. Avila J, Brandt R, Kosik KS 1997. Amsterdam: Harwood Academic Publishers, 185-240.

Wischik CM, Staff RT, Wischik DJ, *et al*. Tau aggregation inhibition therapy: an exploratory Phase 2 study in mild or moderate Alzheimer’s disease. J Alzheimer’s Dis 2015; 44:705-720.

24. APPENDICES

24.1. Assessments by Visit

Before participation in this study may begin, the subject and caregiver will each be provided with an information sheet and both will be given as much time as needed to decide whether or not to participate in the study.

Screening Visit (Visit 1)

Potential subjects will be assessed at one or more screening visits to determine eligibility for the study.

The following assessments will be obtained, performed or measured at Screening:

- Written Informed Consent for participation in the study from the subject (and/or legally acceptable representative[s]) and from the identified caregiver(s) (before any study related procedures may be performed)
- Adverse Events review (after signing of the ICF)
- Demographic information
- Medical history and clinical interview, including details of diagnosis
- Confirmation of probable AD according to NIA/AA criteria
- Prior and concomitant medication review
    - Details of current and recent medication (within 3 months), including dose changes
    - Lifetime history and associated details for AChEIs and memantine
- Modified Hachinski ischemic score
- MMSE
- Total CDR score
- Complete physical and neurological examinations
- Blood pressure and pulse measured in a seated position for at least 5 minutes and then repeated 2 minutes after standing
- Respiratory rate and oral (sublingual) temperature
- Height and body weight
- 12-lead electrocardiogram (ECG) measurements performed in triplicate (three ECG tracings within an approximate 2- to 5-minute interval)
- Blood samples for the following:
    - Chemistry panel (including haptoglobin)
    - Hematology panel
    - G6PD, TSH, Vitamin $B_{12,}$ and folate
    - Serum pregnancy test for women of childbearing potential

- Urine sample for urinalysis
- Methemoglobin and oxygen saturation by pulse co-oximetry
- C-SSRS
- FDG-PET (of sufficient quality, to occur within 42 days prior to Visit 2)
- Brain MRI (of sufficient quality, to occur within 42 days prior to Visit 2)
- Inclusion/exclusion criteria review

If a subject is considered potentially eligible for participation in this study, then the subject's primary medical provider will be notified.

CSF Samples by Lumbar Puncture (Optional Procedure)

CSF samples will be collected at designated study sites only from subjects who are mentally capable of providing their own separate informed consent and specifically agree to have lumbar puncture performed. The Baseline CSF samples may be collected any time prior to the first dose of study drug so long as all screening procedures have been completed and subject eligibility and willingness to continue have been confirmed.

Baseline (Visit 2)

After the Screening assessments, subjects who are considered eligible for participation in this study will return to the study site within 42 days for Baseline assessments and randomization.

Baseline efficacy assessments may be made on the day before randomization and dosing if necessary.

Before Dose Assessments

The following will be performed prior to dosing:

- Inclusion and exclusion criteria review (to confirm continued eligibility)
- ADAS-Cog_{11}
- ADCS-CGIC
- ADCS-ADL_{23}
- NPI
- MADRS
- RUD Lite
- Blood pressure and pulse measured within 1 hour prior to dosing with subject in a seated position for at least 5 minutes and then repeated 2 minutes after standing
- Respiratory rate and oral (sublingual) temperature measured within 1 hour prior to dosing
- Weight
- Concomitant medication recording/review

- Adverse event review
- Blood samples for the following Baseline tests:
  - Chemistry panel
  - Hematology panel
  - Vitamin B_{12} and folate
  - Serum pregnancy test for women of childbearing potential only
- Urine sample for urinalysis
- Methemoglobin and oxygen saturation by pulse co-oximetry within 1 hour prior to dosing
- 12-lead ECG measurements performed in triplicate (three ECG tracings within an approximate 2- to 5-minute interval) (dosing may be held subject to eligibility review based on the local interpretation of triplicate ECGs or the results of the central interpretation in the event of deviations from Screening ECG detected at Baseline and considered clinically significant by the investigator)
- Targeted physical and neurological examinations within 1 hour prior to dosing
- Serotonin Toxicity (Syndrome) Diagnostic Interview and Rating Guide (In Clinic)
- Blood sample for MT concentration (time to be recorded) (at sites with refrigerated centrifuge and appropriate freezer storage capability)
- Blood sample for genotyping (optional; only from those subjects by or for whom legally acceptable consent is provided); may be collected at any time after eligibility for continued participation in the study has been confirmed

Post Dose Assessments

All subjects are to remain in the clinic for at least 4 hours after the first dose. The following will be performed after randomization and dosing are completed at the times indicated:

- Recording of adverse events (throughout in-clinic observation)
- Respiratory rate and oral (sublingual) temperature (hourly until discharge)
- Blood pressure and pulse measured with subject in a seated position for at least 5 minutes and then repeated 2 minutes after standing (approximately 2 hours post-dose)
- Concomitant medication recording/review
- Methemoglobin and oxygen saturation by pulse co-oximetry (approximately 2.5 hours post-dose)
- 12-lead electrocardiogram measurements performed in triplicate (three ECG tracings within an approximate 2- to 5-minute interval) (approximately 3 hours post-dose)
- Targeted physical and neurological examinations (approximately 3 hours post-dose, following ECG measurements); these are to be repeated as needed for subjects who remain in clinic longer than 4 hours

- Blood sample for MT concentration (approximately 3.5 hours post-dose); time to be recorded (at sites with refrigerated centrifuge and appropriate freezer storage capability)
- Serotonin toxicity assessment (following PE and neurological examinations; an assessment is to be completed just prior to discharge from the clinic)
- C-SSRS (prior to discharge from the clinic)
- Any additional assessments required to monitor a treatment-emergent AE to resolution or stabilization

The subject and caregiver will be provided with information about how to correctly take study drug. Study drug will be dispensed to the subject and/or the subject's caregiver. Enough study drug will be dispensed to last until Visit 4.

At discharge from the study unit, subjects (and their caregivers) using any medication with the potential to increase synaptic levels of serotonin will be instructed on signs and symptoms of potential serotonergic toxicity and given an oral thermometer to measure the subject's temperature three times a day (in the morning, afternoon, and evening) until 72 hours after the first dose of study medication. These measurements are to be recorded in a diary to be returned to the clinic at Visit 3. They are to remain in close proximity to a local hospital and remain in telephone contact with the clinic for at least 12–14 hours post-dose; reimbursement for accommodations will be made available if requested by the subject and/or caregiver.

Post-Dose Telephone Contacts (5 – 7, >7 – 14, >14 – 24, 44 – 52, and 68 – 76 Hours after First Dose)

Caregivers of subjects using serotonergic medication will also be contacted by telephone 5-7, >7-14, >14-24, 44-52, and 68-76 hours after the first dose of study drug (with a minimum of 1 hour between contacts) for assessment of potential serotonin toxicity; if indicated, more frequent contacts will be made. They will be informed to have the temperature diary available for these telephone contacts. Instructions for the phone interview are provided in Section 24.3.

Week 2 (Visit 3)

The subjects are to bring all remaining study drug to the clinic to assess compliance. Subjects using any medication with the potential to increase synaptic levels of serotonin are to return their temperature diary to the clinic at this visit.

The following assessments will be performed:

- Blood pressure and pulse measured with subject in a seated position for at least 5 minutes
- Respiratory rate and oral (sublingual) temperature
- Concomitant medication review and recording (*N.B.*, if initiation of a drug with serotonergic potential is considered, the first dose must be given in the clinic and the monitoring outlined on Day 1 followed, *i.e.*, the subject must remain in the clinic for at least 4 hours with close monitoring, oral temperature must be measured following discharge at least three times daily with repeated telephone contacts with the clinic until 68–76 hours post-dose.)
- Adverse event review

- Blood samples for the following tests:
    - Chemistry panel
    - Hematology panel
    - Vitamin B_{12} and folate
    - Serum pregnancy test for women of childbearing potential only
- Urine sample for urinalysis
- Methemoglobin and oxygen saturation by pulse co-oximetry
- 12-lead ECG (single recording, unless there is an emergent abnormality which is of clinical significance in the judgment of the investigator or for subjects with well controlled atrial fibrillation (heart rate ≤ 84 bpm and appropriate anticoagulation) at Baseline, in which cases triplicate ECG recordings will be obtained)
- Targeted physical and neurological examinations
- Serotonin Toxicity (Syndrome) Diagnostic Interview and Rating Guide (In Clinic)
- C-SSRS
- Any additional assessments required to monitor a treatment-emergent AE to resolution or stabilization
- Blood sample for MT concentration (approximately 20 minutes after ECG; time to be recorded) (at sites with refrigerated centrifuge and appropriate freezer storage capability)

Since enough study drug is dispensed at Visit 2 to last until Week 6 (Visit 4), the subject and/or the subject’s caregiver will not be dispensed study drug during this visit.

Week 6 (Visit 4)

The subjects are to bring all remaining study drug to the clinic to assess compliance.

The following assessments will be performed:

- Blood pressure and pulse measured with subject in a seated position for at least 5 minutes
- Respiratory rate and oral (sublingual) temperature
- Weight
- Concomitant medication review and recording (*N.B.*, if initiation of a drug with serotonergic potential is considered, the first dose must be given in the clinic and the monitoring outlined on Day 1 followed, *i.e.*, the subject must remain in the clinic for at least 4 hours with close monitoring, oral temperature must be measured following discharge at least three times daily with repeated telephone contacts with the clinic until 68–76 hours post-dose.)
- Adverse event review
- Blood samples for the following tests:
    - Chemistry panel
    - Hematology panel

  - Vitamin B_{12} and folate
  - Serum pregnancy test for women of childbearing potential only

- Urine sample for urinalysis
- Methemoglobin and oxygen saturation by pulse co-oximetry
- 12-lead ECG (single recording, unless there is an emergent abnormality which is of clinical significance in the judgment of the investigator or for subjects with well controlled atrial fibrillation (heart rate ≤ 84 bpm and appropriate anticoagulation) at Baseline, in which cases triplicate ECG recordings will be obtained)
- Targeted physical and neurological examinations
- Serotonin Toxicity (Syndrome) Diagnostic Interview and Rating Guide (In Clinic)
- C-SSRS
- Any additional assessments required to monitor a treatment-emergent AE to resolution or stabilization
- Blood sample for MT concentration (approximately 20 minutes after ECG; time to be recorded) (at sites with refrigerated centrifuge and appropriate freezer storage capability)

Study drug will be dispensed to the subject and/or the subject’s caregiver. Enough study drug will be dispensed to last until the Week 13 visit (Visit 5).

Telephone Contact (Week 9)

Subjects’ caregivers are to be contacted by telephone at Week 9 for an assessment of safety. An unscheduled visit is to take place if needed in response to a safety concern.

Week 13 (Visit 5)

The subjects are to bring all remaining study drug to the clinic to assess compliance.

The following assessments will be performed:

- ADAS-Cog_{11}
- ADCS-CGIC
- ADCS-ADL_{23}
- Brain MRI
- Blood pressure and pulse measured with subject in a seated position for at least 5 minutes
- Respiratory rate and oral (sublingual) temperature
- Weight
- Concomitant medication review and recording (*N.B.*, if initiation of a drug with serotonergic potential is considered, the first dose must be given in the clinic and the monitoring outlined on Day 1 followed, *i.e.*, the subject must remain in the clinic for at least 4 hours with close monitoring, oral temperature must be measured following

discharge at least three times daily with repeated telephone contacts with the clinic until 68–76 hours post-dose.)

- Adverse event review
- Blood samples for the following tests:
  - Chemistry panel
  - Hematology panel
  - Vitamin B_{12} and folate
  - Serum pregnancy test for women of childbearing potential only
- Urine sample for urinalysis
- Methemoglobin and oxygen saturation by pulse co-oximetry
- 12-lead ECG (single recording, unless there is an emergent abnormality which is of clinical significance in the judgment of the investigator or for subjects with well controlled atrial fibrillation (heart rate ≤ 84 bpm and appropriate anticoagulation) at Baseline, in which cases triplicate ECG recordings will be obtained)
- Targeted physical and neurological examinations
- Serotonin Toxicity (Syndrome) Diagnostic Interview and Rating Guide (In Clinic)
- C-SSRS
- Any additional assessments required to monitor a treatment-emergent AE to resolution or stabilization
- Blood sample for MT concentration (approximately 20 minutes after ECG; time to be recorded) (at sites with refrigerated centrifuge and appropriate freezer storage capability)

Study drug will be dispensed to the subject and/or the subject’s caregiver. Enough study drug will be dispensed to last until the Week 26 visit (Visit 6).

Telephone Contact (Week 19)

Subjects’ caregivers are to be contacted by telephone at Week 19 for an assessment of safety. An unscheduled visit is to take place if needed in response to a safety concern.

Week 26 (Visit 6)

The subjects are to bring all remaining study drug to the clinic to assess compliance.

The following assessments will be performed:

- ADAS-Cog_{11}
- ADCS-CGIC
- ADCS-ADL_{23}
- MMSE
- NPI

- MADRS
- RUD Lite
- Brain MRI
- Blood pressure and pulse measured with subject in a seated position for at least 5 minutes
- Respiratory rate and oral (sublingual) temperature
- Weight
- Concomitant medication review and recording (*N.B.*, if initiation of a drug with serotonergic potential is considered, the first dose must be given in the clinic and the monitoring outlined on Day 1 followed, *i.e.*, the subject must remain in the clinic for at least 4 hours with close monitoring, oral temperature must be measured following discharge at least three times daily with repeated telephone contacts with the clinic until 68–76 hours post-dose.)
- Adverse event review
- Blood samples for the following tests:
    - Chemistry panel
    - Hematology panel
    - Vitamin B_{12} and folate
    - TSH
    - Serum pregnancy test for women of childbearing potential only
- Urine sample for urinalysis
- Methemoglobin and oxygen saturation by pulse co-oximetry
- 12-lead ECG (single recording, unless there is an emergent abnormality which is of clinical significance in the judgment of the investigator or for subjects with well controlled atrial fibrillation (heart rate ≤ 84 bpm and appropriate anticoagulation) at Baseline, in which cases triplicate ECG recordings will be obtained)
- Targeted physical and neurological examinations
- Serotonin Toxicity (Syndrome) Diagnostic Interview and Rating Guide (In Clinic)
- C-SSRS
- Any additional assessments required to monitor a treatment-emergent AE to resolution or stabilization
- Blood sample for MT concentration (approximately 20 minutes after ECG; time to be recorded) (at sites with refrigerated centrifuge and appropriate freezer storage capability)

Study drug will be dispensed to the subject and/or the subject's caregiver. Enough study drug will be dispensed to last until the Week 39 visit (Visit 7).

Telephone Contact (Week 32)

Subjects' caregivers are to be contacted by telephone at Week 32 for an assessment of safety. An unscheduled visit is to take place if needed in response to a safety concern.

Week 39 (Visit 7)

The subjects are to bring all remaining study drug to the clinic to assess compliance.

The following assessments will be performed:

- ADAS-Cog_{11}
- ADCS-CGIC
- ADCS-ADL_{23}
- Brain MRI
- FDG-PET (of sufficient quality)
- Blood pressure and pulse measured with subject in a seated position for at least 5 minutes
- Respiratory rate and oral (sublingual) temperature
- Weight
- Concomitant medication review and recording (*N.B.*, if initiation of a drug with serotonergic potential is considered, the first dose must be given in the clinic and the monitoring outlined on Day 1 followed, *i.e.*, the subject must remain in the clinic for at least 4 hours with close monitoring, oral temperature must be measured following discharge at least three times daily with repeated telephone contacts with the clinic until 68–76 hours post-dose.)
- Adverse event review
- Blood samples for the following tests:
    - Chemistry panel
    - Hematology panel
    - Vitamin B_{12} and folate
    - Serum pregnancy test for women of childbearing potential only
- Urine sample for urinalysis
- Methemoglobin and oxygen saturation by pulse co-oximetry
- 12-lead ECG (single recording, unless there is an emergent abnormality which is of clinical significance in the judgment of the investigator or for subjects with well controlled atrial fibrillation (heart rate ≤ 84 bpm and appropriate anticoagulation) at Baseline, in which cases triplicate ECG recordings will be obtained)
- Targeted physical and neurological examinations
- Serotonin Toxicity (Syndrome) Diagnostic Interview and Rating Guide (In Clinic)
- C-SSRS

- Any additional assessments required to monitor a treatment-emergent AE to resolution or stabilization
- Blood sample for MT concentration (approximately 20 minutes after ECG; time to be recorded) (at sites with refrigerated centrifuge and appropriate freezer storage capability)

Study drug will be dispensed to the subject and/or the subject's caregiver. Enough study drug will be dispensed to last until the Week 52 visit (Visit 8).

Telephone Contact (Week 45)

Subjects' caregivers are to be contacted by telephone at Week 45 for an assessment of safety. An unscheduled visit is to take place if needed in response to a safety concern.

Week 52 (Visit 8)

The subjects are to bring all remaining study drug to the clinic to assess compliance.

The following assessments will be performed:

- ADAS-Cog_{11}
- ADCS-CGIC
- ADCS-ADL_{23}
- NPI
- MADRS
- MMSE
- RUD Lite
- Brain MRI
- Blood pressure and pulse measured with subject in a seated position for at least 5 minutes
- Respiratory rate and oral (sublingual) temperature
- Weight
- Concomitant medication review and recording (*N.B.*, if initiation of a drug with serotonergic potential is considered, the first dose must be given in the clinic and the monitoring outlined on Day 1 followed, *i.e.*, the subject must remain in the clinic for at least 4 hours with close monitoring, oral temperature must be measured following discharge at least three times daily with repeated telephone contacts with the clinic until 68–76 hours post-dose.)
- Adverse event review
- Blood samples for the following tests:
    - Chemistry panel
    - Hematology panel
    - Vitamin B_{12} and folate

  - TSH
  - Serum pregnancy test for women of childbearing potential only

- Urine sample for urinalysis
- Methemoglobin and oxygen saturation by pulse co-oximetry
- 12-lead ECG (single recording, unless there is an emergent abnormality which is of clinical significance in the judgment of the investigator or for subjects with well controlled atrial fibrillation (heart rate ≤ 84 bpm and appropriate anticoagulation) at Baseline, in which cases triplicate ECG recordings will be obtained)
- Targeted physical and neurological examinations
- Serotonin Toxicity (Syndrome) Diagnostic Interview and Rating Guide (In Clinic)
- C-SSRS
- Any additional assessments required to monitor a treatment-emergent AE to resolution or stabilization
- Blood sample for MT concentration (approximately 20 minutes after ECG; time to be recorded) (at sites with refrigerated centrifuge and appropriate freezer storage capability)

Study drug will be dispensed to the subject and/or the subject’s caregiver. Enough study drug will be dispensed to last until the Week 65 visit (Visit 9).

Telephone Contact (Week 58)

Subjects’ caregivers are to be contacted by telephone at Week 58 for an assessment of safety. An unscheduled visit is to take place if needed in response to a safety concern.

Week 65 (Visit 9)

The subjects are to bring all remaining study drug to the clinic to assess compliance.

The following assessments will be performed:

- ADAS-Cog_{11}
- ADCS-CGIC
- ADCS-ADL_{23}
- Brain MRI
- Blood pressure and pulse measured with subject in a seated position for at least 5 minutes
- Respiratory rate and oral (sublingual) temperature
- Weight
- Concomitant medication review and recording (*N.B.*, if initiation of a drug with serotonergic potential is considered, the first dose must be given in the clinic and the monitoring outlined on Day 1 followed, *i.e.*, the subject must remain in the clinic for at least 4 hours with close monitoring, oral temperature must be measured following

discharge at least three times daily with repeated telephone contacts with the clinic until 68–76 hours post-dose.)

- Adverse event review
- Blood samples for the following tests:
    - Chemistry panel
    - Hematology panel
    - Vitamin B_{12} and folate
    - Serum pregnancy test for women of childbearing potential only
- Urine sample for urinalysis
- Methemoglobin and oxygen saturation by pulse co-oximetry
- 12-lead ECG (single recording, unless there is an emergent abnormality which is of clinical significance in the judgment of the investigator or for subjects with well controlled atrial fibrillation (heart rate ≤ 84 bpm and appropriate anticoagulation) at Baseline, in which cases triplicate ECG recordings will be obtained)
- Targeted physical and neurological examinations
- Serotonin Toxicity (Syndrome) Diagnostic Interview and Rating Guide (In Clinic)
- C-SSRS
- Any additional assessments required to monitor a treatment-emergent AE to resolution or stabilization
- Blood sample for MT concentration (approximately 20 minutes after ECG; time to be recorded) (at sites with refrigerated centrifuge and appropriate freezer storage capability)

Study drug will be dispensed to the subject and/or the subject’s caregiver. Enough study drug will be dispensed to last until the Week 78 visit (Visit 10).

Telephone Contact (Week 71)

Subjects’ caregivers are to be contacted by telephone at Week 71 for an assessment of safety. An unscheduled visit is to take place if needed in response to a safety concern.

End of Treatment Visit: Week 78 (Visit 10) or Early Termination Visit

The subjects are to bring all remaining study drug to the clinic to assess compliance.

The following assessments will be performed:

- ADAS-Cog_{11}
- ADCS-CGIC
- ADCS-ADL_{23}
- MMSE
- NPI

- MADRS
- RUD Lite
- Brain MRI
- FDG-PET
- Blood pressure and pulse measured with subject in a seated position for at least 5 minutes
- Respiratory rate and oral (sublingual) temperature
- Weight
- Concomitant medication review and recording (*N.B.*, if initiation of a drug with serotonergic potential is considered, the first dose must be given in the clinic and the monitoring outlined on Day 1 followed, *i.e.*, the subject must remain in the clinic for at least 4 hours with close monitoring, oral temperature must be measured following discharge at least three times daily with repeated telephone contacts with the clinic until 68–76 hours post-dose.)
- Adverse event review
- Blood samples for the following tests:
  - Chemistry panel
  - Hematology panel
  - Vitamin B_{12} and folate
  - TSH
  - Serum pregnancy test for women of childbearing potential only
- Urine sample for urinalysis
- Methemoglobin and oxygen saturation by pulse co-oximetry
- 12-lead ECG (single recording, unless there is an emergent abnormality which is of clinical significance in the judgment of the investigator or for subjects with well controlled atrial fibrillation (heart rate ≤ 84 bpm and appropriate anticoagulation) at Baseline, in which cases triplicate ECG recordings will be obtained)
- Targeted physical and neurological examinations
- Serotonin Toxicity (Syndrome) Diagnostic Interview and Rating Guide (In Clinic)
- C-SSRS
- Any additional assessments required to monitor a treatment-emergent AE to resolution or stabilization
- Blood sample for MT concentration (approximately 20 minutes after ECG; time to be recorded) (at sites with refrigerated centrifuge and appropriate freezer storage capability)
- CSF sample (optional; at designated sites only from subjects who are mentally capable of providing their own separate informed consent and specifically agree to have lumbar puncture performed); lumbar puncture to be obtained after efficacy and other safety assessments are performed

Study drug will not be dispensed. The subject will cease taking study drug after the Week 78 or early termination visit.

Unscheduled Visit

The subjects are to bring all remaining study drug to the clinic to assess compliance. The following assessments are to be performed as appropriate to the safety concern:

- Respiratory rate and oral (sublingual) temperature
- Blood pressure and pulse measured with subject in a seated position for at least 5 minutes
- Weight
- Blood samples will be obtained for the following tests:
    - Chemistry panel
    - Hematology panel
    - Vitamin B_{12} and folate
    - Serum pregnancy test for women of childbearing potential only
- Blood sample for MT concentration (approximately 20 minutes after ECG; time to be recorded) (at sites with refrigerated centrifuge and appropriate freezer storage capability)
- Urine sample for urinalysis
- Methemoglobin and oxygen saturation by pulse co-oximetry
- 12-lead ECG (single recording, unless there is an emergent abnormality which is of clinical significance in the judgment of the investigator or for subjects with well controlled atrial fibrillation (heart rate ≤ 84 bpm and appropriate anticoagulation) at Baseline, in which cases triplicate ECG recordings will be obtained)
- Targeted physical and neurological examinations
- Serotonin Toxicity (Syndrome) Diagnostic Interview and Rating Guide (In-Clinic)
- Recording of adverse events
- Concomitant medication review and recording (*N.B.*, if initiation of a drug with serotonergic potential is considered, the first dose must be given in the clinic and the monitoring outlined on Day 1 followed, *i.e.*, the subject must remain in the clinic for at least 4 hours with close monitoring, oral temperature must be measured following discharge at least three times daily with repeated telephone contacts with the clinic until 68–76 hours post-dose.)
- C-SSRS
- Any additional assessments required to monitor a treatment-emergent AE to resolution or stabilization

The subject is to be scheduled to return to the clinic for the next scheduled visit or additional unscheduled visits as appropriate.

Follow-up Visit (Visit 11)

A follow-up visit will be performed approximately 28 (± 7) days after the last dose of study medication. The following assessments will be performed (and any other assessments necessary to follow the resolution of a treatment-emergent adverse event):

- ADAS-Cog_{11}
- ADCS-CGIC
- ADCS-ADL_{23}
- MMSE
- Blood pressure and pulse measured with subject in a seated position for at least 5 minutes
- Respiratory rate and oral (sublingual) temperature
- Weight
- Concomitant medication review and recording
- Adverse event review
- Blood samples for the following tests:
  - Chemistry panel
  - Hematology panel
  - Vitamin B_{12} and folate
  - Serum pregnancy test for women of childbearing potential only
- Urine sample for urinalysis
- Methemoglobin and oxygen saturation by pulse co-oximetry
- 12-lead ECG (single recording, unless there is an emergent abnormality which is of clinical significance in the judgment of the investigator or for subjects with well controlled atrial fibrillation (heart rate ≤ 84 bpm and appropriate anticoagulation) at Baseline, in which cases triplicate ECG recordings will be obtained)
- Targeted physical and neurological examinations
- Serotonin Toxicity (Syndrome) Diagnostic Interview and Rating Guide (In Clinic)
- C-SSRS

Subjects who complete the study, including the off-treatment follow-up visit, may be offered an opportunity to subsequently receive treatment with LMTM in a separate open-label extension study.

24.2. Serotonin Toxicity (Syndrome) Diagnostic Interview and Rating Guide (In-Clinic)

Administration and Scoring

This guide is designed to enable the investigator to elicit clinical information that will allow diagnosis of serotonin toxicity (serotonin syndrome) by four published criteria and to quantify severity of symptoms.

The assessment has three sections: mental state, neurological and autonomic. These sections can be rated in any order and information gathered from the subject's general physical and neurological examination can be used to rate the items with the proviso that the stated conditions are met.

All items, apart from item 4, can be rated and scored in the clinic after the first dose of study medication. Item 4 can only be rated beginning on Day 2.

For each item a decision must be made as to whether the symptom or sign is absent (score 0) or present (score 1).

For items 7, 17, 18, 19 and 20 the physiological measurement is referenced to the physical and neurological examinations, except on Day 1 when they must also be recorded on the serotonin syndrome scoring sheet.

For items 1, 2, 5, 6, 10, 12, 15, 16 and 17 a further symptom severity rating (SSS score) must also be made.

Assessment scores must then be entered into the serotonin syndrome worksheet.

Interpretation

Subjects should be considered a possible case of serotonin toxicity if they meet the criteria for any of the four criteria following exposure to MT.

For possible cases the investigator is recommended to refer to the subject's Baseline score and consider whether any of the positive items are simply due to the dementia. For example in a subject displaying moderate agitation at Baseline and moderate agitation 3 hours post introduction of MT, it is reasonable to conclude that the observed agitation is not due to serotonin toxicity. Diarrhea on the other hand may be a relatively common AE and should not form the basis for inferring possible serotonin toxicity.

Subjects still meeting criteria for serotonin syndrome after Baseline symptoms have been discounted should be considered probable serotonin syndrome cases.

For all probable cases of serotonin toxicity and for any subject developing pyrexia, possibly due to serotonin toxicity, the advice of a physician with expertise in toxicology must be immediately sought before the subject is allowed to leave the clinic.

A) MENTAL STATE

1) Abnormal Conscious Level (Delirium)

Rate this item positive if the subject on examination displays behavior suggestive of an abnormal conscious level (i.e., clouding, hypervigilance, hypovigilance) supported by an acute deterioration in at least one of the following: attention, thinking, constructional praxis or orientation on cognitive examination.

0 = Symptom absent
1 = Symptom present

SSS score
0 = no change in MMSE orientation
3 = at least 2 point deterioration in MMSE orientation

2) Agitation or Akathisia

Rate this item positive if the subject on examination displays excessive, non-goal directed motor behavior, for example pacing, foot tapping or restlessness, irrespective of whether it is considered to be agitation or akathisia.

0 = Symptom absent
1 = Symptom present

SSS score
0 = None
1 = Slight and intermittent
2 = Moderate (unrest while sitting)
3 = Severe and long lasting (sitting is nearly impossible, subject always feels restless)

3) Elevated Mood

Rate this item positive if any of the following are present on mental state examination: pressured speech, elation, mania or hypomania.

0 = Symptom absent
1 = Symptom present

4) Insomnia

Rate this item positive (beginning on Day 2) if the informant reports the subject to be having more difficulty falling or staying asleep since commencing the study drug.

Symptom not applicable = N/A (use only if informant is not available)
0 = Symptom absent
1 = Symptom present

B) NEUROLOGICAL

5) Myoclonus

Rate this item positive if subject on examination displays sudden shock-like muscle jerks. These may occur spontaneously or in reaction to sensory stimulation or movement.

0 = Sign absent
1 = Sign present

SSS score
0 = No myoclonus
1 = Subject or informant reports short episodes of myoclonus
2 = Subject or informant reports repeated episodes; isolated myoclonic jerks are visible
3 = Persistent, visible myoclonic jerks

6) Tremor

Rate this item positive if subject on examination displays clinically relevant tremor at rest, on maintaining posture, or on intention.

0 = Sign absent
1 = Sign present

SSS score
0 = No tremor
1 = Tremor with small amplitude; functioning is not impaired
2 = Tremor with significant amplitude; functioning (*e.g.*, holding a cup) is moderately impaired
3 = Tremor with high amplitude; functioning is severely impaired

7) Mydriasis

Measure pupillary diameter in daylight.

Pupillary diameter = __________mm (OS)

(Where the left and right pupils differ in diameter, each should be recorded and appropriately labeled)

Pupillary diameter = __________mm (OD)

Rate this item positive if subject on examination has a pupillary diameter (in either one or both eyes) of 5mm or greater.

0 = Enlarged pupil absent
1 = Enlarged pupil present

8) Nystagmus

Rate this item positive if subject displays nystagmus. This should also be elicited by getting the subject to fix on a finger moved rapidly to the midline.

0 = Nystagmus absent
1 = Nystagmus present

9) Clonus

Rate this item positive if there are more than 3 beats of clonus at any site. Clonus must be tested in both upper and lower limbs.

0 = Clonus absent
1 = Clonus present

10) Hyperreflexia

Rate this item positive if reflex is abnormally brisk or associated with clonus, scored as below. Reflexes must be tested in both upper and lower limbs.

0 = Sign absent
1 = Sign present

SSS score
0 = No hyperreflexia
1 = Hyperreflexia without clonus
2 = Hyperreflexia with non-sustained clonus
3 = Hyperreflexia with sustained clonus

11) Hypertonia (Rigidity)

Rate this item positive if muscle tone is increased with features of rigidity. Tone must be tested in both the upper and lower limbs.

0 = Rigidity absent
1 = Rigidity present

12) Dizziness

Rate this item positive if the subject reports feeling dizzy or lightheaded. Similarly rate item positive if there is on examination evidence that subject is unsteady or has poor balance whilst standing or walking. When positive, scores are to be assigned as below.

0 = Symptom absent
1 = Symptom present

SSS score
0 = None

1 = Slight and intermittent feelings of dizziness or imbalance
2 = Subject feels dizzy or imbalance most of the time; functioning (moving, standing) is not impaired)
3 = Subject always feels dizzy or imbalance; functioning (moving, standing) is affected

13) Incoordination

Rate this item positive if the subject on examination displays lack of control or inaccuracy in voluntary movement. Coordination should be tested in both upper (e.g., finger-nose test) and lower (e.g., heel-shin test) limbs.

0 = Incoordination absent
1 = Incoordination present

C) AUTONOMIC

14) Shivering

Rate this item positive if on examination there is involuntary: shaking, trembling, quivering or teeth chattering as if the subject is cold.

0 = Shivering absent
1 = Shivering present

15) Diaphoresis (Sweating)

Rate this item positive if on examination the subject's skin feels moist or beads of perspiration can be seen. This must be rated at rest at normal environmental temperature and scored as below.

0 = Sign absent
1 = Sign present

SSS score
0 = No sweating
1 = Subjective feeling of increased sweating
2 = Moist skin; some beads of perspiration can be seen
3 = Visible beads of perspiration with wet clothes or bed sheet

16) Diarrhea

Rate this item by asking the subject about recent bowel habits.

0 = Sign absent
1 = Sign present

SSS score
0 = No diarrhea

1 = Feces with reduced consistency, but normal frequency
2 = Liquid feces and/or frequency 1-3/day
3 = Like 2 but frequency > 3 /day

17) Pyrexia (Fever)

Record oral temperature =__________ºC

0 = Sign absent
1 = Sign present

Rate this item positive if on examination the oral temperature is 38 ºC or above, scored as below.

SSS score
0 = <37 ºC
1= 37-37.9 ºC
2 = 38-38.9 ºC
3 = ≥ 39 ºC

18) Tachycardia

Measure resting heart rate after 5 minutes in a sitting position.

Resting heart rate =__________bpm

Rate this item positive if on examination (or ECG) the heart rate > 96 bpm.

0 = Absent
1 = Present

19) Tachypnea or Dyspnea

Measure resting respiratory rate *after 5 minutes in a sitting position.*

Resting respiratory rate = __________ bpm

Rate this item positive if either:

i) the subject reports dyspnea (difficulty breathing or shortness of breath) or
ii) there is tachypnea on examination (breathing rate of > 20 breaths / minute)

0 = Absent
1 = Positive

20) Hypertension or Hypotension

Measure blood pressure after 5 minutes in a sitting position.

Systolic blood pressure = __________ mmHg

Diastolic blood pressure =__________mmHg

Rate this item positive if on examination either:

i) systolic blood pressure > 160 mmHg, or diastolic blood pressure > 100 mmHg or
ii) systolic blood pressure < 100 mmHg or diastolic blood pressure < 50 mmHg

0 = Absent
1 = Positive

Serotonin Syndrome Worksheet

**Item**		**Sternbach**	**Hunter**	**Hegerl**	**Radomski**	
					Major	Minor
1	Abnormal Conscious Level (Delirium)	0 1		0 3	0 1	
2	Agitation or Akathisia	0 1	0 1	0 1 2 3		0 1
3	Elevated Mood				0 1	
4	Insomnia					0 1
5	Myoclonus	0 1	0 1	0 1 2 3	0 1	
6	Tremor	0 1	0 1	0 1 2 3	0 1	
7	Mydriasis					0 1
8	Nystagmus		0 1			
9	Clonus		0 1			
10	Hyperreflexia	0 1	0 1	0 1 2 3	0 1	
11	Hypertonia (Rigidity)		0 1		0 1	
12	Dizziness			0 1 2 3		
13	Incoordination	0 1				0 1
14	Shivering	0 1			0 1	
15	Diaphoresis (Sweating)	0 1	0 1	0 1 2 3	0 1	
16	Diarrhea	0 1		0 1 2 3		0 1
17	Pyrexia (Fever)	0 1	0 1	0 1 2 3	0 1	
18	Tachycardia					0 1
19	Tachypnea or Dyspnea					0 1
20	Hypertension or Hypotension					0 1
	**Total Score**					
	**Interpretation**	**Score of 3 or above = case**	**See Algorithm below**	**Score of 6 or above = case**	**Score of 4 major = case<br>Score of 3 major + 2 minor = case**	

Hunter Serotonin Toxicity Criteria - a case is diagnosed if any of the criteria a-e are met.

a) Myoclonus (item 5)
b) Tremor (item 6) and hyperreflexia (item 10)
c) Nystagmus (item 8) and either agitation (item 2) or diaphoresis (item 15)
d) Clonus (item 9) and either agitation (item 2) or diaphoresis (item 15)
e) Hypertonia (item 11), temperature >38°C (item 17), and either nystagmus (item 8) or clonus (item 9)

24.3. Serotonin Toxicity Telephone Assessment

The assessment should be administered to the subject's caregiver *via* the telephone.

Has subject (S) had any new or worsening symptoms since starting ….?

Describe

____
____
____
____
____
____
____

1) Has S appeared more confused since starting …..?

2) Has S appeared more restless or agitated since starting…….?

3) Has S appeared excited or manic since starting …..?

4) Has S been having more difficulty falling or staying asleep since starting …?

5) Has S appeared more jumpy or jerky since starting ….?

6) Has S appeared more tremulous since starting ……?

7) Has S developed any unusual eye movements since starting…?

8) Has S become stiff or rigid since starting ……?

9) Has S complained more of feeling dizzy or lightheaded since starting ….?

10) Has S developed any problems with balance since starting …?

11) Has S become more clumsy or uncoordinated since starting …?

12) Has S started to shiver or tremble since starting ….?

13) Has S appeared or felt more sweaty since starting …..?

14) Has S developed diarrhea (frequent or loose motions) since starting ….?

15) Has S had a temperature above 37 °C since starting……….?

16) Has S complained of feeling breathless or appeared breathless since starting ….?

<u>Interpretation</u>

If a caregiver replies "yes" to any of the above, the subject should be immediately recalled to the clinic and examined for evidence of serotonin toxicity.

24.4. Summary of Changes to the Protocol

24.4.1. Protocol Version 1.1

The final protocol for Study TRx-237-005 (Version 1.0 dated 13 July 2012) has been revised (Version 1.1 dated 24 September 2012) to remove the requirement for prior contact with the medical monitor in the event of a medical emergency and to acknowledge that national laws regarding consent in individuals with reduced decision-making capacity may differ. The affected sections for these changes are detailed below.

Summary of Changes	Affected Section(s) in Revised Protocol (Version 1.1)
Consistent with ICH GCP 4.3, an investigator no longer needs to contact the medical monitor in order to unblind the treatment assignment of a specific subject if needed for managing a medical emergency.	Section 4.5 Randomization and Blinding
Protocol text has been modified to cover all national laws regarding informed consent in individuals with reduced decision-making capacity.	Synopsis (Informed Consent and Methodology subsections)<br>Section 5.1 Inclusion Criteria<br>Section 14 Informed Consent<br>Section 24.1 Appendix A: Assessments by Visit (Screening Visit)

24.4.2. Protocol Version 2.0

The final protocol for Study TRx-237-005 (Version 1.1 dated 24 September 2012) has been revised (Version 2.0 dated 24 October 2012). The majority of the revisions are non-substantial and administrative and/or editorial in nature.

Procedures for which visit windows are now specified or clarified throughout the protocol:

- Telephone contacts for evaluation of serotonin toxicity 48 and 72 hours after the first dose (± 4 hours) and between visits (± 7 days)
- MRI and FDG-PET/CT scans (± 14 days)

Revisions that are changes to inclusion/exclusion criteria, dosing and drug supplies, efficacy and safety assessments, and other procedures are summarized in the table below. Changes that are substantive clarifications in the event of discrepancies are also included.

Summary of Changes	Affected Sections in Revised Protocol (Version 2.0)
Study Personnel	
Identification of the vendors for imaging activities has been updated: BioClinica, Inc. for MRI image acquisition and site interaction, RadMD, LLC for imaging oversight and providing the central blinded readers, and MNI LLC for PET.	Section 1.2 Responsible Personnel
Pharmacovigilance and medical monitoring are to be performed by a different vendor (Worldwide Clinical Trials); contact details and reporting process for SAEs and pregnancy have been revised accordingly.	Section 1.2 Responsible Personnel<br>Section 8.1.4 Serious Adverse Events<br>Section 8.1.5 Pregnancy<br>Section 8.1.6.1 Investigator Reporting of SAEs and Pregnancy to Sponsor
Background	
The expression of the MTC dose has been edited to be in terms of MT rather than the salt, for consistency.	Section 2.2 Nonclinical Data
The discussion of nonclinical data has been updated to include new reproductive toxicity findings; the discussion of contraceptive measures has been updated accordingly.	Section 2.2 Nonclinical Data<br>Section 4.7.5 Contraceptive Measures
Study Population	
The study will now also be conducted at sites in Asia and will no longer be conducted at sites in Australia.	Synopsis / Study Sites<br>Section 4.2 Population
Inclusion and Exclusion Criteria	
The acceptable MMSE range at Screening has been extended to 20-26, inclusive (previously 22-26).	Synopsis / Inclusion Criteria<br>Section 4.2 Population<br>Section 5 Selection of Subjects and Criteria for Withdrawal<br>Section 5.1 Inclusion Criterion No. 2
Examples of acceptable methods of contraception are clarified.	Synopsis / Inclusion Criteria<br>Section 4.7.5 Contraceptive Measures<br>Section 5.1 Inclusion Criterion No. 5

Clarified that genotyping evaluation of Apolipoprotein E will be performed only in subjects by or for whom legally acceptable consent is provided, to cover all national laws regarding informed consent in individuals with reduced decision-making capacity.	Synopsis / Objectives<br>Section 3.3 Exploratory<br>Section 4.4 Schedule of Assessments<br>Section 24.1 Assessments by Visit
Clarified that if a subject is currently taking an AChEI and/or memantine, the subject must have been taking such medication(s) for ≥ 3 months before Screening, and the current dosage regimen and dosage form must have remained stable for ≥ 6 weeks before Screening.	Synopsis / Inclusion Criteria<br>Section 4.2 Population<br>Section 4.7.1 Dementia Medication<br>Section 5.1 Inclusion Criterion No. 8
Exclusion criteria have been expanded with additional examples of significant CNS disorders (Lewy body dementia) and intracranial pathology (large confluent white matter hyperintense lesions).	Synopsis / Exclusion Criteria<br>Section 5.2 Exclusion Criteria Nos. 1 and 2<br>Section 9.1.5.1 Evaluation of Brain MRI for Subject Eligibility
The exclusion criterion for hemoglobin references values at Screening, rather than values at Baseline.	Synopsis / Exclusion Criteria<br>Section 5.2 Exclusion Criterion No. 11
The exclusion criterion for Vitamin B_{12} and folate references values at Screening, rather than values at Baseline. Subjects with borderline or deficient values are to be excluded, rather than values below age/sex appropriate lower limit of the central laboratory normal range, as the central laboratory used in this study has not established normal ranges for either parameter. Guidance for normal, borderline, and deficient ranges for both parameters is provided based on published literature (Kratz *et al*., 2004).	Synopsis / Exclusion Criteria<br>Section 4.7.6 Folate and Vitamin B_{12} (Section added)<br>Section 5.2 Exclusion Criterion No. 11
Dosing and Drug Supplies	
Clarified that study drug is to be permanently discontinued in response to ARIA; further details of ARIA have been provided.	Synopsis / Safety and Tolerability<br>Section 6.3.3.3 ARIA<br>Section 9.1.3.2 Brain MRI<br>Section 9.1.6 Site Review
Enough study drug will be dispensed upon discharge from the study unit at Visit 2 such that study drug will not be dispensed at Visit 3; only a compliance check will be performed at Visit 3.	Synopsis / Methodology<br>Section 4.4 Schedule of Assessments<br>Section 6.5 Dispensing<br>Section 6.6 Compliance<br>Section 24.1 Assessments by Visit
Subjects will be instructed to take each dose of study medication with a full glass of water.	Section 6.2 Study Regimens
Statements that study drug should not be frozen, refrigerated, and protected from direct heat have been removed; packing is suitably protective, as supported by stability data.	Section 6.4 Packaging, Labeling, and Storage<br>Section 6.5 Dispensing
Efficacy Assessments	
Baseline efficacy assessments can be made on the day prior to randomization and dosing.	Synopsis / Methodology<br>Section 4.4 Schedule of Assessments<br>Section 7 Assessment of Efficacy<br>Section 24.1 Assessments by Visit
The secondary efficacy variables, NPI and MADRS, will be rated at Baseline (Visit 2, pre-dose) and after 26, 52, and 78 weeks.	Synopsis / Efficacy<br>Section 4.4 Schedule of Assessments<br>Section 7.2.4 Neuropsychiatric Inventory (NPI)<br>Section 7.2.5 Montgomery-Asberg Depression Rating Scale (MADRS)
The ADCS-CGIC is to be rated in the caregiver first (rather than the subject); the reference point for assessment of change (Baseline visit) has been clarified.	Section 7.1 Raters<br>Section 7.2.2 Alzheimer's Disease Cooperative Study – Clinical Global Impression of Change (ADCS-CGIC)

Safety Assessments	
It has been further clarified that the same in-clinic and follow-up telephone monitoring procedures are to be followed for subjects starting a drug with serotonergic potential during study.	Section 4.7.2 Drugs with Serotonergic Potential<br>Section 24.1 Assessments by Visit
The caregiver will be contacted by telephone either for queries of serotonin syndrome or between in-clinic visits.	Synopsis<br>Section 2.4 Rationale<br>Section 4.1 General Description<br>Section 4.4 Schedule of Assessments<br>Section 6.3.3.2 Serotonin Syndrome<br>Section 8 Assessment of Safety<br>Section 8.5.1 Temperature and Respiratory Rate<br>Section 8.8 Serotonin Syndrome<br>Section 24.1 Assessments by Visit
MCH, MCHC are now specified as analytes to be included in the hematology panel.	Section 8.3.2 Hematology
Vitamin B_{12} will be no longer be measured at Screening only; levels will also be measured at Baseline (Visit 2; pre-dose), Visits 3–10 while on treatment (or upon early termination), and at the follow-up visit (Visit 11).	Synopsis<br>Section 4.4 Schedule of Assessments<br>Section 8 Assessment of Safety<br>Section 8.3 Clinical Laboratory Tests<br>Section 8.3.4 Other Laboratory Tests<br>Section 24.1 Assessments by Visit
Clarification that G6PD testing will be performed using a kit supplied to the sites for on-site testing.	Section 4.4 Schedule of Assessments<br>Section 8.3.4 Other Laboratory Tests
Vital signs are to be measured within 1 hour prior to dosing.	Synopsis / Safety and Tolerability<br>Section 4.4 Schedule of Assessments<br>Section 8.5.1 Temperature and Respiratory Rate<br>Section 8.5.2 Blood Pressure and Pulse<br>Section 24.1 Assessments by Visit
All ECG recordings (including Screening) will be 10 seconds in duration.	Section 4.4 Schedule of Assessments<br>Section 8.6 Electrocardiography<br>Section 24.1 Assessments by Visit
The body systems to be evaluated in the complete neurological examination have been updated.	Section 8.7 Physical and Neurological Examinations
Other Assessments	
The following items have been modified in the overview of the image acquisition method for FDG-PET/CT:<br><br>Blood glucose should be below 180 mg/dL (not 150 mg/dL) prior to the administration of FDG (based on current Alzheimer's Disease Neuroimaging Initiative [ADNI] recommendations).<br><br>A standard dose of 5 mCi (not 10 mCi) should be injected intravenously.<br><br>At 30 minutes (previously stated to be 1 hour) following the administration of FDG, the subject should be positioned into the PET/CT instrument.	Section 9.1.3.1 FDG-PET/CT
Procedures for site review of brain MRI for subject management and determination of subject eligibility have been revised.	Section 9.1.6 Site Review
Nunc polypropylene tubes are to be used for blood sample collection for analysis of MT concentrations instead of plain glass tubes.	Section 9.4.1 Procedure for Blood Sample Collection

New contact provided for sending MT samples for analysis.	Section 9.4.3 Analytical Laboratory
For blood samples obtained for genotyping, the tubes and storage conditions have been modified and shipping instructions have been provided.	Section 9.5 Genotyping
Miscellaneous	
If a caregiver of a subject withdraws his or her consent, the subject must then also be withdrawn if alternative arrangements are not available (*e.g.*, alternate caregiver). The caregiver may also withdraw his or her consent at any time for any reason.	Section 5.3 Discontinuation/Withdrawal<br>Section 14 Informed Consent
Procedures for review and recording of concomitant medications have been clarified.	Section 4.4 Schedule of Assessments<br>Section 4.7 Concomitant Medication<br>Section 24.1 Assessments by Visit
Public registries where general trial data may be posted have been updated.	Section 14 Informed Consent
Publication procedures have been clarified.	Section 19 Publication
Editorial changes and clarifications have been made to the serotonin toxicity (syndrome) diagnostic interview and rating guide.	Section 24.2 Serotonin Toxicity (Syndrome) Diagnostic Interview and Rating Guide (In-Clinic)

24.4.3. Protocol Version 2.1

The final protocol for Study TRx-237-005 (Version 2.0 dated 24 October 2012) has been revised (Version 2.1 dated 12 December 2012). The majority of the revisions are non-substantial and administrative and/or editorial in nature.

Revisions that are modifications or clarifications to inclusion/exclusion criteria, statistical analysis, efficacy and safety assessments/procedures, and other procedures are summarized in the table below. Additional revisions are intended to correct typographical errors, eliminate inconsistencies or add further clarification.

Summary of Changes	Affected Section(s) in Revised Protocol (Version 2.1)
Background	
Further updates have been added to the discussion of recently identified reproductive toxicity findings.	Section 2.2 Nonclinical Data
Updates have been added to the discussion of clinical pharmacokinetic and safety data.	Section 2.3.1 Pharmacokinetics<br>Section 2.3.3 Safety
Inclusion and Exclusion Criteria	
Clarification that subjects are to be less than 90 years of age at Screening to be eligible for enrollment in the study.	Synopsis / Inclusion Criteria<br>Section 5.1 Inclusion Criterion No. 3
For exclusion of subjects meeting DSM IV-TR criteria for substance (including alcohol) related disorders, the specified time period has been reduced from within the past 5 years to within the past 2 years.	Synopsis / Exclusion Criteria<br>Section 5.2 Exclusion Criterion No. 5
The normal ranges for folate and Vitamin B_{12} established by the manufacturer of the test kits used by the central laboratory are now specified; subjects who are below the normal range at Screening (confirmed upon repeat) are to be excluded. Guidance is provided for interpreting values and a brief description of the central laboratory's analytical method is included.	Synopsis / Exclusion Criteria<br>Section 4.7.7 Folate and Vitamin B_{12}<br>Section 5.2 Exclusion Criterion No. 11<br>Section 8.3.4 Other Laboratory Tests
Reference to Baseline has been removed from the exclusion criteria related to QTcB, blood pressure, and heart rate measurements.	Synopsis / Exclusion Criteria<br>Section 5.2 Exclusion Criterion No. 13
Treatment currently or within 3 months before Baseline with moderate to strong inhibitors of CYP1A2 is no longer included in the criteria for subject exclusion.	Synopsis / Exclusion Criterion No. 18<br>Section 5.2 Exclusion Criterion No. 18<br>Section 4.7.2 Drugs with Serotonergic Potential<br>Section 4.7.5 Other Medications
Clarification that the exclusion criteria related to participation in a clinical trial applies to both current and prior participation; clinical trials of medical foods are now included in the criteria.	Synopsis / Exclusion Criteria<br>Section 5.2 Exclusion Criterion No. 19
Efficacy Assessments / Procedures	
The MMSE version to be used in the study is now specified to be a modified version of the original form supplied by Psychological Assessment Resources.	Section 7.2.7 Mini-Mental State Examination (MMSE)
Safety Assessments / Procedures	
Clarification has been added regarding dosing and study continuation decisions to be made based on ECGs on Day 1.<br><br>Dosing may be held based on local interpretation (using the average of the three readings for QTcB and heart rate) and subsequent eligibility decisions made pending receipt of centrally read results in the event of deviations from Screening ECG detected at Baseline and	Synopsis / Methodology<br>Section 4.4 Schedule of Assessments<br>Section 6.3.3.4 QT Interval and ECG Abnormalities<br>Section 8.6 Electrocardiography<br>Section 10.3.5.4 Electrocardiogram<br>Section 24.1 Assessments by Visit

Summary of Changes	Affected Section(s) in Revised Protocol (Version 2.1)
considered clinically significant. A decision as to whether to discontinue the subject or re-schedule the Baseline visit should be made on the basis of the central read; a cardiology consult should be sought if appropriate.<br><br>Subsequent decisions with respect to the ECG may be made on the basis of the central reading, with cardiology consult if appropriate. In addition to the central reading results, local machine-read results from Screening and Baseline are to be recorded in the eCRF and included in a separate listing.	
The C-SSRS will now be applied at Screening (Visit 1), and will not be applied pre-dose at Baseline (Visit 2).	Synopsis / Safety and Tolerability<br>Section 4.4 Schedule of Assessments<br>Section 8.9 Columbia-Suicide Severity Rating Scale (C-SSRS)<br>Section 24.1 Assessments by Visit
Clarification has been added that if dosing is held for more than 30 days in response to methemoglobinemia, the subject should be discontinued.	Section 6.3.3.1 Methemoglobinemia and/or Hemolytic Anemia
RBC morphology is now specified as an analyte to be included in the hematology panel.	Section 8.3.2 Hematology
Clarification that Heinz body determination is not mandated.	Section 8.3.2 Hematology
The reporting and handling requirements for AESIs have been clarified; possible cases of serotonin toxicity and ARIA will be reported to the Sponsor and the procedure will be as for SAEs.	Section 8.1.3 Adverse Events of Special Interest<br>Section 8.1.4 Serious Adverse Events
The measurement of pulse rate has been increased from 30 seconds to 60 seconds.	Section 8.5.2 Blood Pressure and Pulse
The protocol now indicates that the serotonin toxicity assessment requires a qualified medical assessor.	Section 4.4 Schedule of Assessments<br>Section 6.3.3.2 Serotonin Syndrome<br>Section 8.8 Serotonin Syndrome
Statistical Analysis	
For stratification and efficacy analysis, two levels will be used for AChEI and/or memantine status at randomization: current ongoing use, or not ongoing use.	Synopsis / Dose/Route/Regimen<br>Synopsis / Primary Statistical Analyses<br>Section 4.5 Randomization and Blinding<br>Section 10.3.2.3 Primary Clinical Efficacy Analysis<br>Section 10.3.2.8.2 Subgroup Analyses<br>Section 10.3.8 Cerebrospinal Fluid Biomarkers
Other Assessments / Procedures	
Corrected scheduled study days for Weeks 65, 78, and 82.	Section 4.1 General Description<br>Section 4.4 Schedule of Assessments
Subject identification numbering has been clarified; subjects will be assigned a unique number (005-CC-SSS-EE) with the first three digits for the study (005), the next two digits for the country, the next three digits for the site, and the last two digits for the sequential order of enrollment at a given site.<br><br>Subjects who are re-screened will receive a new identification number; the previous number is also to be recorded by the site. Initial screening data and rescreening data will be captured in the database for subjects who are re-screened.	Section 4.5 Randomization and Blinding<br>Section 5.2 Exclusion Criteria
With respect to concomitant medications, "medication" is now clarified to encompass prescription and over-the-counter drugs or	Section 4.7 Concomitant Medication<br>Section 4.7.1 Dementia Medication

Summary of Changes	Affected Section(s) in Revised Protocol (Version 2.1)
biologics, vitamins used in supra-pharmacologic doses, alternative pharmacotherapies for dementia, medical foods, and for women, forms of contraception. Initiation of folate therapy during participation in the study is no longer specified as to be avoided.	
Based on preliminary results from a recent drug-drug interaction study which suggest that MT may inhibit cytochrome P450 3A4 (CYP3A4), additional guidance is provided regarding the monitoring of subjects on drugs known to be metabolized by this enzyme system; examples of these drugs are provided.	Section 4.7.1 Dementia Medication<br>Section 4.7.3 CYP3A4 Substrates (Section added)
For subjects who have been receiving a medical food or a stable dose of alternative pharmacotherapy for dementia, it is preferable that the dose of such therapy has remained stable for ≥ 6 months before randomization; examples of medical foods have been added.	Section 4.7.1 Dementia Medication<br>Section 10.3.2.8.2 Subgroup Analyses
For brain MRI scans, 1.5-Tesla and 3.0-Tesla machines will be used.	Section 9.1.3.2 Brain MRI
For blood samples collected for genotyping, the blood volume will be 10 mL (not 8.5 mL) and sample storage conditions have been clarified (*i.e.*, under refrigeration or preferably frozen). Sample analysis will take place within approximately 10 weeks after the collection of samples; samples will be destroyed within 6 months.	Section 9.5 Genotyping
Where local laws require it, national regulatory requirements with regard to the inclusion of subjects who are unable to consent will be followed by the investigators. In particular, in Germany, the risk threshold and degree of burden/distress will be monitored constantly by the investigators in accordance with §41 (3) of the German Drug Law (AMG). Additional detail is now provided regarding the evaluation of the risk threshold by the Sponsor and the appointed DSMB, as well as the monitoring of the degree of burden/distress by the participating investigators.	Section 15 Investigator Responsibilities

24.4.4. Protocol Version 2.2

The final protocol for Study TRx-237-005 (Version 2.1 dated 12 December 2012) has been revised (Version 2.2 dated 19 March 2013). The revisions are non-substantial and administrative and/or editorial in nature.

Revisions that are modifications or clarifications to study personnel, inclusion/exclusion criteria, statistical analysis, efficacy and safety assessments/procedures, and other procedures are summarized in the table below. Additional revisions are intended to add further clarity to the text, eliminate inconsistencies, or correct typographical errors.

Summary of Changes	Affected Sections in Revised Protocol (Version 2.2)
A new contact is listed under the responsible personnel for pharmacovigilance.	Section 1.2 Responsible Personnel
The study will no longer be conducted at sites in Asia; stratification based on geographic region has been updated accordingly to remove Rest of World as the study will be conducted in North America and Europe.	Section 1.2 Responsible Personnel<br>Synopsis / Study Site(s)<br>Synopsis / Dose/Route/Regimen<br>Synopsis / Primary Statistical Analyses<br>Section 4.2 Population<br>Section 4.5 Randomization and Blinding<br>Section 10.3.2.3 Primary Clinical Efficacy Analysis<br>Section 10.3.2.8.2 Subgroup Analyses<br>Section 10.3.8 Cerebrospinal Fluid Biomarkers
The power calculation based on the planned sample size has been updated to include newer information; the number of subjects has not changed.	Synopsis / Number of Subjects<br>Section 10.2.1 ADAS-Cog_{11}
Added clarification to inclusion criterion to specify adequate contraception for female subjects in Italy (*i.e.*, accept to avoid a pregnancy for at least 3 months prior to Baseline and throughout participation in the study).	Synopsis / Inclusion Criteria<br>Section 5.1 Inclusion Criterion No. 5
Added clarification to inclusion criterion to specify adequate informed consent for subjects in Germany (*i.e.*, subjects must be able to provide their own written informed consent).	Synopsis / Inclusion Criteria<br>Section 5.1 Inclusion Criterion No. 6<br>Section 14 Informed Consent
Added clarification to exclusion criterion to specify that residence in low grade assisted living facility is allowed so long as it is not mandated by an order issued either by the judicial or the administrative authorities.	Synopsis / Exclusion Criteria<br>Section 5.2 Exclusion Criterion No. 7
Clarified the criteria for subject discontinuation based on ARIA findings and added summary of the appropriate imaging follow-up to be performed in response to ARIA findings (to be consistent with the Imaging Charter).	Synopsis / Assessments<br>Section 4.4 Schedule of Assessments<br>Section 6.3.3.3 ARIA<br>Section 9.1.6 Site Review
Corrected lower limit of the range for mean plasma elimination half-life observed in healthy elderly volunteers following single doses of LMTB.	Section 2.3.1 Pharmacokinetics<br>Section 2.4 Rationale for Study
Removed statements referring to patients with moderate Alzheimer's disease for clarity and consistency throughout the protocol.	Section 2.4 Rationale for Study<br>Section 10.3.2.4 Additional Analyses of Primary Endpoints
Clarified the timing of clinical laboratory tests in the schedule of assessments and added footnote to indicate that an unscheduled visit is to take place if needed in response to a safety concern.	Section 4.4 Schedule of Assessments

Summary of Changes	Affected Sections in Revised Protocol (Version 2.2)
For subjects with digoxin levels measured at the local laboratory, results are to be entered into the eCRF and included in a listing.	Section 4.7.3 CYP3A4 and PgP Substrates<br>Section 10.3.3 Baseline Characteristics and Concomitant Medications
Expanded guidance regarding use of anxiolytics and/or sedatives/hypnotics with regards to subject participation in the study.	Section 4.7.4 Drugs Used to Manage Behavioral Disturbance<br>Section 4.7.5 Other Medications
For determining whether or not supplementation should be given for Vitamin B_{12}, the protocol now indicates this evaluation may also include measurement of homocysteine and methylmalonic acid (by a local laboratory) to determine whether or not the low levels are physiologically significant.	Section 4.7.7 Folate and Vitamin B_{12}
In discussion of reasons for discontinuation of study drug, it is now clarified that “lack of efficacy” includes worsening of cognitive capacity, and that in Germany this includes loss of the ability to give consent.	Section 5.3 Discontinuation/Withdrawal
Clarified reasons for termination of the study; the Sponsor reserves the right to terminate the study for duly justified reasons in accordance with the national laws.	Section 5.4 Termination of the Study
Clarified procedures for repeat testing and treatment discontinuation in response to treatment-emergent QT interval and ECG abnormalities.	Section 6.3.3.4 QT Interval and ECG Abnormalities
Study drug packaging has been clarified; to allow for delays in visit scheduling (or if original medication is lost or damaged), one additional 14-tablet blister card will be included in cartons for Visits 2 and 4, and two additional 14-tablet blister cards will be included in cartons for Visits 5, 6, 7, 8, and 9.	Section 6.4 Packaging, Labeling, and Storage
Clarified the assessment of CDR and MMSE questionnaires by an independent evaluator at Worldwide Clinical Trials.	Section 7.1 Raters
Included estimated total blood volume to be collected for each subject over the course of the study.	Section 8 Assessment of Safety
Updated the estimated blood volume to be collected for each hematology panel (decreased from 3.0 mL to 2.0 mL).	Section 8.3.2 Hematology
Updated clinical laboratory sample shipping conditions and recipients of shipments for T3 uptake samples and TBG samples; added clarification regarding serum pregnancy testing and procedures for borderline results.	Section 8.3.4 Other Laboratory Tests
Added guidance regarding use of pulse co-oximeter for measurement of methemoglobin and oxygen saturation, *i.e.*, because this is a light-emitting device, it should be left on only so long as needed to obtain the recording(s); this is consistent with the guidance in the Investigator Brochure.	Section 8.4 Pulse Co-Oximetry
Added details regarding estimated radiation exposure from each FDG-PET/CT scan and total radiation exposure over the course of the study for each subject, as well as revisions to the image acquisition method for consistency with the most current ADNI protocol.	Section 9.1.3.1 FDG-PET/CT
Specified the central laboratory to which CSF samples will be shipped.	Section 9.3 Cerebrospinal Fluid Biomarkers

Summary of Changes	Affected Sections in Revised Protocol (Version 2.2)
Updated sample shipping conditions for blood samples collected for PK analysis; removed contact listed for analytical laboratory due to changes in personnel.	Section 9.4.2 Packaging, Labeling, and Shipping of Blood Samples<br>Section 9.4.3 Analytical Laboratory
Clarified the blinded interim analyses that may be carried out at some point during the study's recruitment period to re-estimate the assumed standard deviation for the change form Baseline to Week 78 in ADAS-Cog_{11}.	Section 10.2 Sample Size Justification<br>Section 10.4 Interim Analyses
An additional sensitivity analysis has been added: repeated measures analysis of the ADCS-CGIC using the Generalized Estimating Equation (GEE) model.	Section 10.3.2.4 Additional Analyses of Primary Endpoints
Deleted text describing the handling of partial data for the efficacy endpoints as this is more appropriately described in the SAP.	Section 10.3.2.7 Missing Data

24.4.5. Protocol Version 3.0

The final protocol for Study TRx-237-005 (Version 2.2 dated 19 March 2013) has been revised (Version 3.0 dated 21 August 2013). Revisions that are modifications or clarifications to the overall protocol / background information; inclusion / exclusion criteria; study drug administration / packaging; efficacy, safety, and other assessments / procedures; statistical analysis; and administrative procedures are summarized in the table below.

Other revisions are editorial and/or non-substantial and are intended to add further clarification, eliminate inconsistencies, or correct typographical errors.

Summary of Changes	Affected Sections in Revised Protocol (Version 3.0)
Title Page	
The Title Page has been revised to list the Scotland address (for the convenience of communications) in addition to the Singapore address (the Sponsor's registered address).	Title Page
Overall / Background	
Address and contact details added for the Sponsor Global Project Lead, Sponsor Head of Safety and Medical Monitoring, and the central laboratory location in the United States. ECG laboratory changed to BioClinica, Inc. and contact numbers added for the University of Aberdeen GLP Test Facility.	Section 1.2 Responsible Personnel
The duration of Screening has now been extended to 6 weeks (42 days). As a result, the total duration of participation for an individual subject is now planned to be 88 weeks. It is anticipated that the study will have an overall duration of approximately 34 months.	Synopsis<br>Section 4.1 General Description<br>Section 4.3 Duration<br>Section 4.4 Schedule of Assessments<br>Section 5.2 Exclusion Criteria No. 2<br>Section 9.1.3.1 FDG-PET<br>Section 9.1.3.2 Brain MRI<br>Section 24.1 Assessments by Visit
Clarified that subjects who complete the study, including the off-treatment follow-up visit, may (rather than will) be offered an opportunity to subsequently receive treatment with LMTM in a separate open-label extension study, as this may be dependent upon other factors such as tolerability and response.	Synopsis<br>Section 4.1 General Description<br>Section 24.1 Assessments by Visit
The target recruitment number is now 700 subjects, 350 per arm; the sample size justification, number of planned screened subjects, and anticipated number of completed subjects have been updated accordingly.	Synopsis<br>Section 4.2 Population<br>Section 4.5 Randomization and Blinding<br>Section 10.2 Sample Size Justification<br>Section 10.2.1 ADAS-cog_{11}<br>Section 10.2.2 ADCS-CGIC<br>Section 10.2.3 FDG-PET<br>Section 10.4 Interim Analyses
Updated background information for clinical pharmacokinetics of MT.	Section 2.3.1 Pharmacokinetics
FDG-PET/CT (scan) now appears as FDG-PET throughout the protocol as the FDG-PET scan will involve using CT or brain-dedicated high resolution PET devices, such as the Siemens HRRT system that uses a transmission source for attenuation correction, will also be considered.	Synopsis<br>Section 2.4 Study Rationale<br>Section 4.4 Schedule of Assessments<br>Section 9.1 ^{18}F-Flurodeoxyglucose Positron Emission Tomography (FDG-PET) and Magnetic Resonance

	Imaging (MRI)<br>Section 9.1.3 Imaging Methods<br>Section 9.1.3.1 FDG-PET
Inclusion / Exclusion Criteria	
Inclusion criterion has been modified for CDR total score at Screening; subjects must have a total score of 1 (a total score of 0.5 is no longer acceptable). As a result, screening severity based on CDR is no longer a randomization stratum and there will be no subgroup analyses performed based on CDR.	Synopsis<br>Section 4.2 Population<br>Section 4.5 Randomization and Blinding<br>Section 5.1 Inclusion Criterion No. 2<br>Section 10.3.2.3 Primary Clinical Efficacy Analysis<br>Section 10.3.2.4 Additional Analyses of Primary Endpoints<br>Section 10.3.2.5 Analyses for Disease Modification<br>Section 10.3.2.6 Secondary Endpoints<br>Section 10.3.2.8.2 Subgroup Analyses<br>Section 10.3.8 Cerebrospinal Fluid Biomarkers
Female subjects are required to be competent to use contraception.	Synopsis<br>Section 5.1 Inclusion Criterion No. 5
Caregiver inclusion criterion has been revised such that subjects may have one or more identified caregivers who meet certain criteria, including living with or seeing subjects for certain hours/days of week or the investigator deems the extent of contact sufficient to detect meaningful change.	Synopsis<br>Section 5.1 Inclusion Criterion No. 7
Additional examples of significant CNS disorders other than AD have been included.	Synopsis<br>Section 5.2 Exclusion Criterion No. 1
Exclusion criterion has been modified to no longer allow subjects with Screening hemoglobin (confirmed upon repeat) below age/sex appropriate lower limit of the central laboratory normal range to be treated and rescreened if levels are corrected.	Synopsis<br>Section 5.2 Exclusion Criterion No. 11
Exclusion criterion has been modified for folate and Vitamin B_{12} and supplementation guidelines provided if values are below < 4.0 ng/mL and/or < 150 pg/mL, respectively, prior to initiating drug. If appropriate Vitamin B_{12} levels can be achieved within the 42-day screening window, the subject may be entered into the study; otherwise the subject must be reconsented and rescreened after the deficit has been corrected. Folate supplementation is to be maintained for the duration of the study, and additional guidance is provided if folate and Vitamin B_{12} levels become deficient during the study.	Synopsis<br>Section 4.7.8 Folate and Vitamin B_{12}<br>Section 5.2 Exclusion Criterion No. 11<br>Section 8.3.5 Review of Laboratory Results
Exclusion criterion has been modified to clarify that evidence of uncontrolled atrial fibrillation on Screening ECG or history of atrial fibrillation is exclusionary only if no currently controlled (heart rate ≥ 85 bpm and/or inappropriate anticoagulation) or where QT interval cannot in the opinion of the investigator be assessed by triplicate ECGs taken within a 2- to 5-minute interval. If better control of the heart rate and of anticoagulation can be achieved after adequate treatment, the subject may be entered into the study if still within the 42-day window, or else the subject must be reconsented and rescreened. Additional guidance is provided regarding a cardiology consult. QTcF is to be used and based on the mean of three triplicate ECGs. The exclusionary limit	Synopsis<br>Section 4.4 Schedule of Assessments<br>Section 5.2 Exclusion Criterion No. 13<br>Section 6.3.3.4 QT Interval and ECG Abnormalities<br>Section 8.6 Electrocardiography<br>Section 10.3.5.4 Electrocardiogram<br>Section 24.1 Assessments by Visit

for males has been raised to >460 msec.	
Subjects with hepatitis are to be excluded from the study. Exclusion criterion has been expanded to include additional examples of concurrent acute or chronic clinically significant immunologic diseases other than AD.	Synopsis<br>Section 5.2 Exclusion Criterion No. 15
Treatment currently or within 3 months before Baseline with anxiolytics and/or sedatives/hypnotics is no longer included as exclusion criteria. Guidance has been clarified regarding subjects' use of these types of medications during participation in the study.	Synopsis<br>Section 4.7.4 Drugs Used to Manage Behavioral Disturbance<br>Section 4.7.5 Other Medications<br>Section 5.2 Exclusion Criterion No. 18
Exclusion criterion regarding current/prior participation in a clinical trial of a product for cognition within the 3 months prior to Screening (unless randomized to placebo) now includes all clinical trials rather than only Phase 3 trials.	Synopsis<br>Section 5.2 Exclusion Criterion 19
Study Drug Administration / Packaging	
Instructions for dosing interruption have been clarified to specify that interruption of dosing may be allowed for up to (maximum duration on any one occasion) 30 days during the first 6 months of treatment and/or 14 days during the second 12 months of treatment.	Synopsis<br>Section 4.1 General Description<br>Section 4.6 Study Treatment<br>Section 6.3.1 Interruption<br>Section 6.3.3.1 Methemoglobinemia and/or Hemolytic Anemia
Subjects should be entered into the study only after careful discussion of the possibility that discoloration in the context of incontinence may prove unacceptable to the subject/caregiver.	Section 6.1.1 Active Ingredient
Study drug should not be dissolved in fluids prior to ingestion. Subjects should not be enrolled if there are swallowing difficulties which prevent taking the medication as instructed and subjects/caregivers should be warned regarding discoloration of teeth and oral mucosa if the product is not swallowed immediately.	Synopsis<br>Section 5.2 Exclusion Criterion No. 8<br>Section 6.2 Study Regimens<br>Section 6.5 Dispensing
Revised description of drug packaging configuration to allow greater flexibility in drug supplies.	Section 6.4 Packaging, Labeling, and Storage
Efficacy / Exploratory Assessments	
Clarified the efficacy rater requirements and ratings to be determined for the ADCS-CGIC.	Section 7.1 Raters<br>Section 7.4 Alzheimer's Disease Cooperative Study – Clinical Global Impression of Change (ADCS-GCIC)
The CSF biomarkers to be assessed have been clarified to include total tau, phospho-tau, and $A\beta_{1-42}$. Information regarding sample shipment, batch testing, destruction, and stability has been added.	Synopsis<br>Section 3.3 Exploratory<br>Section 9.3 Cerebrospinal Fluid Biomarkers<br>Section 10.1.3 Exploratory and Other Endpoints<br>Section 10.3.8 Cerebrospinal Fluid Biomarkers
The change at 39 weeks on FDG-PET will also be compared with that observed at the same time in Study TRx-237-015.	Section 2.4 Rationale for Study
Safety Assessments / Procedures	
Background information regarding safety has been updated to provide more detail regarding the potential risk of MAO inhibition and the theoretical risk of increased serotonin levels with oral LMTM (or its concomitant use with serotonergic drugs). Added potential risk caused by ingestion of foods containing high amounts of tyramine, symptoms of a hypertensive crisis and examples of tyramine-rich foods	Section 2.3.3 Safety<br>Section 2.4 Rationale for Study<br>Section 4.7.2 Drugs with Serotonergic Potential<br>Section 4.7.6 Dietary Tyramine

and beverages to be avoided. Tryptophan or its metabolites need not be avoided despite serotonergic potential.	
MRI scans of the brain obtained at Screening/Baseline are no longer required to be contrast-enhanced.	Synopsis<br>Section 4.4 Schedule of Assessments<br>Section 9.1.3.2 Brain MRI
ECGs will be obtained in triplicate at the Screening visit (within a 2- to 5-minute interval with the subject in a supine position, suitably rested for at least 5 minutes) and at Visit 2; subsequently (at all other visits or upon early termination), single recordings will be made, unless there is an emergent abnormality which is of clinical significance in the judgment of the investigator, in which case triplicate ECG recordings should be made. Eligibility, dosing, and monitoring guidance have been provided for subjects with atrial fibrillation and in subjects with intraventricular conduction blocks, as appropriate.	Synopsis<br>Section 4.4 Schedule of Assessments<br>Section 6.3.3.4 QT Interval and ECG Abnormalities<br>Section 8.6 Electrocardiography<br>Section 10.3.5.4 Electrocardiogram<br>Section 24.1 Assessments by Visit
Methemoglobin and oxygen saturation will be measured within 1 hour before administration of the first dose of study drug, rather than approximately 1 hour prior to dosing.	Synopsis<br>Section 4.4 Schedule of Assessments<br>Section 8.4 Pulse Co-oximetry<br>Section 24.1 Assessments by Visit
The telephone contacts with caregivers of subjects receiving serotonergic medication are now specified to occur with a minimum of 1 hour between contacts. Also, subjects and their caregiver(s) are to remain in close proximity to a local hospital and remain in telephone contact with the clinic for at least 12 to 14 hours after the first dose of medication.	Synopsis<br>Section 2.4 Rationale for Study<br>Section 4.4 Schedule of Assessments<br>Section 6.3.3.2 Serotonin Syndrome<br>Section 8.5.1 Temperature and Respiratory Rate<br>Section 8.8 Serotonin Syndrome (Toxicity)<br>Section 24.1 Assessments by Visit
Added clarification that any subject newly starting a serotonergic drug during the study should be subjected to the same monitoring procedures	Section 6.3.3.2 Serotonin Syndrome
Additional examples of alternative pharmacotherapy for dementia that may be continued during participation in the study are included.	Section 4.7.1 Dementia Medication<br>Section 10.3.2.8.2 Subgroup Analyses<br>Section 10.3.3 Baseline Characteristics and Concomitant Medications
Updated guidance regarding monitoring of concomitant medications is provided based on preliminary results from a recently completed drug-drug interaction study which indicate that MT is a weak inhibitor of CYP3A4, CYP2C8, and CYP2C19, as well as a weak inducer of CYP2B6 and the P-gp transporter. The protocol now refers to the Investigator’s Brochure for examples of drugs metabolized by these enzymes or substrates of P-gp.	Section 4.7.3 CYP and P-gp Substrates
In addition to sedation for claustrophobia, anxiolytics and/or sedatives/hypnotics may also be used to manage agitation or excessive movement during imaging. Low dose trazodone (50 mg) may also be used as needed at bedtime for sleep (with appropriate monitoring for signs/symptoms of serotonin toxicity).	Section 4.7.5 Other Medications
Guidelines for discontinuing treatment due to ECG abnormalities have been revised: QTc interval prolongation text has been removed and QTc interval values have been modified to >500 msec in either sex.	Section 6.3.3.4 QT Interval and ECG Abnormalities
SAE definition now indicates that deaths include suicides, and other medically significant events have been clarified.	Section 8.1.4 Serious Adverse Events

Given that all subjects will receive some amount of LMTM, treatment allocation will not be unblinded for this study; guidance for Sponsor reporting of SUSARs to regulatory authorities has been revised accordingly.	Section 8.1.6.2 Sponsor Reporting of SUSARs to Regulatory Authorities<br>Section 8.1.6.2.1 Unblinding Treatment Allocation
Sample storage periods prior to destruction for clinical laboratory testing (*i.e.*, hematology, chemistry, urinalysis, Vitamin B_{12}, folate, TSH, TBG, T_3 uptake, haptoglobin, and G6PD) have been added.	Section 8.3.1 Serum Chemistry<br>Section 8.3.2 Hematology<br>Section 8.3.3 Urinalysis<br>Section 8.3.4 Other Laboratory Tests
Because MT potentially interferes with colorimetric measurements, the protocol has been updated to require extra samples. Information regarding storage, analysis, and discarding of samples have been added.	Section 8.3.3 Urinalysis
For G6PD deficiency screening, use of a local laboratory is permitted.	Section 8.3.4 Other Laboratory Tests
In the Serotonin Toxicity (Syndrome) Diagnostic Interview and Rating Guide, the rating for mydriasis has been updated to indicate that where the left and right pupils differ in diameter, each should be recorded and appropriately labeled.	Section 24.2 Serotonin Toxicity (Syndrome) Diagnostic Interview and Rating Guide (In-Clinic)
Other Assessments / Procedures	
Additional guidance provided in the event that the initial Screening/Baseline FDG-PET scan or MRI scan is not of sufficient quality to serve as an adequate baseline, for subjects who are rescreened for other reasons and an acceptable scan was already completed during the original screening window, and for subjects who discontinue study drug and/or withdraw from the study prematurely.<br>The estimated amount of total radiation exposure over the course of the study has been updated accordingly.	Section 4.4 Schedule of Assessments<br>Section 9.1.3.1 FDG-PET<br>Section 9.1.3.2 Brain MRI
Specified that for site radiologist review of MRI results for subject management and preliminary determination of eligibility, as well as for treatment-emergent abnormalities, a re-review by the central reader may be requested on the basis of additional clinical/radiological information known to the site.	Section 9.1.5.1 Evaluation of Brain MRI for Subject Eligibility<br>Section 9.1.6 Site Review
Samples collected for pharmacokinetic analysis are to be destroyed after a storage period of 3 months following completion of the clinical study report, which is expected to be a period of approximately 4 years.	Section 9.4.2 Packaging, Labeling, and Shipping of Blood Samples
Additional details regarding storage and shipment of genotyping samples have been provided.	Section 9.5 Genotyping
Statistical Analysis	
The statistical analysis text has been revised for the two co-primary endpoints to indicate that Week 65 (not Week 52) data will be used for analysis if fewer than 60% of subjects are retained in either treatment arm at Week 78.	Synopsis<br>Section 10.3.2.1 Hypotheses<br>Section 10.3.2.3 Primary Clinical Efficacy Analysis<br>Section 10.3.2.4 Additional Analyses of Primary Endpoints
Expected rate of decline (in units) of ADAS-cog_{11} in untreated/placebo subjects has been revised.	Section 10.2.1 ADAS-cog_{11}
Clarified that imaging results will be analyzed for inferential purposes for disease modification if clinical efficacy is demonstrated on the ADAS-cog_{11} scale, and that only the FDG-PET endpoint will be required to establish disease	Section 10.3.2.1 Hypotheses<br>Section 10.3.2.2 Multiple Comparisons and Multiplicity<br>Section 10.3.2.5 Analyses for Disease Modification

modification. The change from baseline comparison is specified to examine whether change in FDG-PET at Week 39 predicts clinical non-decliner status at Week 78 (or Week 65 if fewer than 60% of subjects are retained in either treatment arm being compared at Week 78).	
For the sensitivity analyses of the pre-specified primary analysis, non-responders are now described as subjects who do not have a final ADAS-cog score. Other definitions for responder thresholds may be considered. The dichotomized data for ADCS-CGIC results and thresholds for defining a responder have been clarified. In addition, decline is now specified to be moderate or marked, as well as without any decline.	Section 10.3.2.4 Additional Analyses of Primary Endpoints<br>Section 10.3.2.8.2 Subgroup Analyses
Estimated glomerular filtration rate (eGFR) will be calculated based on the Modification of Diet in Renal Disease (MDRD) Study, to be further described in the SAP.	Section 10.3.5.2 Laboratory Tests of Blood and Urine
Any local interpretation of ECG used to make dosing or patient management decisions is now specified as to be included in the eCRF. Parameters to be included for statistical analyses now also include RR and uncorrected QT intervals.	Section 10.3.5.4 Electrocardiogram
For statistical summaries of ARIA data, the number of microhemorrhages will be tabulated by treatment group.	Section 10.3.5.5 ARIA
Revised so that if the overall dropout for the duration of the study is projected to exceed 40%, then the number of subjects to be enrolled may be increased in order to power an analysis at 65 weeks or 78 weeks.	Section 10.4 Interim Analyses
Other Procedures / Miscellaneous	
In addition to the investigator, all other parties involved in the conduct of the study are also responsible for ensuring that the study is conducted at their sites in accordance with the approved protocol and with the principles of the Declaration of Helsinki, ICH GCP, and with applicable country and local regulatory requirements and laws.	Section 11 Regulatory<br>Section 13 Serious Breaches
Specified timing and criteria of clinical monitoring visits.	Section 17 Quality Assurance and Clinical Monitoring
Accommodation in certain circumstances by prior agreement with the Sponsor has been added to the statement regarding reimbursable travel costs by TauRx to study subjects.	Section 21 Administrative and Financial Agreement
Pipettes have been added as equipment for which calibration certification will be confirmed.	Section 22 Study Administration
New references added or removed as applicable to changes in the body of the protocol; information for previously cited references corrected.	Section 23 References

24.4.6. Protocol Version 4.0

The final protocol for Study TRx-237-005 (Version 3.0 dated 21 August 2013) has been revised (Version 4.0 dated 30 October 2013). Changes that are modifications to the inclusion and exclusion criteria, safety and other assessments or procedures, as well as other administrative revisions are summarized in the table below.

Additional revisions are editorial and are intended to correct typographical errors, eliminate inconsistencies or add further clarification.

Summary of Changes	Affected Sections in Revised Protocol (Version 4.0)
Administrative	
Address and telephone number have been updated for one of the protocol signatories (Statistician).	Section 1.1 Protocol Approval
Only the WCT Global Lead Medical Monitor is identified in the tabular summary of responsible personnel rather than each of the WCT Regional Medical Monitors.	Section 1.2 Responsible Personnel
The locations of study sites are now presented by region rather than country; text referring to “North America” has been revised to “the Americas”. Where appropriate, text referring to “European Union” has been revised to “Europe” so that the region is not restricted to European Union member states.	Section 1.2 Responsible Personnel<br>Synopsis<br>Section 4.2 Population<br>Section 4.5 Randomization and Blinding<br>Section 10.3.2.3 Primary Clinical Efficacy Analysis<br>Section 10.3.2.8.2 Subgroup Analyses<br>Section 10.3.8 Cerebrospinal Fluid Biomarkers
Inclusion / Exclusion Criteria	
Inclusion criterion has been modified for CDR total score to allow a CDR total score of 0.5 or 1 at Screening as the acceptable severity to be eligible for inclusion in the study (rather than restricting to a CDR total score of 1 as in Protocol Version 3.0). As a result, screening severity based on CDR as a randomization stratum and the subgroup analyses based on CDR have been re-incorporated into the protocol.	Synopsis<br>Section 4.1 General Description<br>Section 4.2 Population<br>Section 4.5 Randomization and Blinding<br>Section 5.1 Inclusion Criterion No. 2<br>Section 10.3.2.3 Primary Clinical Efficacy Analysis<br>Section 10.3.2.4 Additional Analyses of Primary Endpoints<br>Section 10.3.2.5 Analyses for Disease Modification<br>Section 10.3.2.6 Secondary Endpoints<br>Section 10.3.2.8.2 Subgroup Analyses<br>Section 10.3.8 Cerebrospinal Fluid Biomarkers
Exclusion criterion regarding the use of drugs associated with methemoglobinemia has been modified to specify drugs known to pose more than a very rare risk (1/10,000 cases) of methemoglobinemia at approved doses (*e.g.*, dapsone, local anesthetics such as benzocaine used chronically, primaquine and related antimalarials); sulfonamides are no longer listed as excluded.	Synopsis<br>Section 5.2 Exclusion Criterion No. 18
Exclusion criterion regarding current or prior participation in a clinical trial of a product for cognition has been modified to specify participation prior to Screening in which the last dose was received within 90 days prior to Screening unless confirmed to have been randomized to placebo.	Synopsis<br>Section 5.2 Exclusion Criterion No. 19

Summary of Changes	Affected Sections in Revised Protocol (Version 4.0)
Safety Assessments / Procedures	
Treatment allocation will be unblinded for SUSAR reporting by the Sponsor to the pertinent regulatory authorities; guidance for SUSAR reporting and unblinding treatment allocation has been revised accordingly.	Section 8.1.6.2 Sponsor Reporting of SUSARs to Regulatory Authorities<br>Section 8.1.6.2.1 Unblinding Treatment Allocation
G6PD deficiency screening is no longer restricted to being performed using a commercially available kit supplied to the sites for on-site testing or by using a local laboratory; the protocol has been modified to allow a suitable laboratory to be used for measurement of G6PD.	Section 8.3.4 Other Laboratory Tests
Other Assessments / Procedures	
For the initial CSF sample, lumbar puncture is no longer restricted to being performed after efficacy and other safety assessments are performed at Baseline, Visit 2 (prior to dosing); the protocol now only specifies that CSF samples are to be collected prior to the first dose of study drug and at the end of treatment (Week 78 or upon early termination).	Synopsis<br>Section 4.4 Schedule of Assessments<br>Section 9.3 Cerebrospinal Fluid Biomarkers<br>Section 24.1 Assessments by Visit
Benzodiazepines are now excluded as sedatives prior to FDG-PET scans.	Section 4.7.4 Drugs Used to Manage Behavioral Disturbance<br>Section 4.7.5 Other Medications<br>Section 9.1.3.1 FDG-PET

24.4.7. Protocol Version 4.1

The final protocol for Study TRx-237-005 (Version 4.0 dated 30 October 2013) has been revised (Version 4.1 dated 6 December 2013). The revisions include the addition of sites in Australia, the extension of the projected duration of the enrollment period (and accordingly, the overall projected duration of the study), clarification of the exclusion criterion regarding the use of drugs associated with methemoglobinemia, clarification of informed consent required for the lumbar puncture procedure to be performed for exploratory assessment of CSF biomarkers, and modification to the allowable timing of the Baseline lumbar puncture procedure. The revisions and affected sections are summarized in the table below.

Additional revisions are editorial and are intended to correct typographical errors, eliminate inconsistencies or add further clarification.

Summary of Changes	Affected Sections in Revised Protocol (Version 4.1)
Administrative	
Sites in Australia have been added; for purposes of randomization and statistical analyses, Europe and Australia will be grouped as one geographic region.	Synopsis<br>Section 4.2 Population<br>Section 4.5 Randomization and Blinding<br>Section 10.3.2.3 Primary Clinical Efficacy Analysis<br>Section 10.3.2.8.2 Subgroup Analyses<br>Section 10.3.8 Cerebrospinal Fluid Biomarkers
The enrollment period has been extended and is now projected to be 12 to 24 months; accordingly, the projected overall duration of the study is now estimated to be 34 to 46 months (extended from 34 months).	Synopsis<br>Section 4.3 Duration<br>Section 8.3.3 Urinalysis
Exclusion Criteria	
Use of drugs associated with methemoglobinemia is exclusionary and examples of drugs for which this occurs at approved doses is given. The language is clarified and reference to a specific incidence is removed as it is not clearly established. The intent of this exclusion criterion is unchanged.	Synopsis<br>Section 5.2 Exclusion Criterion No. 18
Exploratory Assessments	
Clarification has been added that lumbar puncture is to be performed only in subjects who are mentally capable of providing their own separate informed consent and who specifically agree to have it performed. The protocol now allows the Baseline CSF samples to be collected any time prior to the first dose of study drug so long as all screening procedures have been completed and subject eligibility and willingness to continue have been confirmed.	Synopsis<br>Section 3.3 Exploratory Objectives<br>Section 4.4 Schedule of Assessments<br>Section 9.3 Cerebrospinal Fluid Biomarkers<br>Section 10.1.3 Exploratory and Other Endpoints<br>Section 14 Informed Consent<br>Section 24.1 Assessments by Visit

24.4.8. Protocol Version 5.0

The final protocol for Study TRx-237-005 (Version 4.1 dated 6 December 2013) has been revised (Version 5.0 dated 15 May 2014) to identify a new contact for pharmacovigilance and to include modifications and/or clarifications to study drug dose reduction, efficacy, safety, and other assessments or procedures as summarized in the table below.

Additional revisions are editorial and/or intended to add minor clarification.

Summary of Changes	Affected Sections in Revised Protocol (Version 5.0)
Administrative / Background	
A new contact is listed for pharmacovigilance.	Section 1.2 Responsible Personnel
The enrollment period has been extended; accordingly, the projected overall duration of the study is now indicated to be at least 34 months (depending on recruitment rate).	Synopsis<br>Section 4.3 Duration<br>Section 8.3.3 Urinalysis
The background discussion of clinical data has been updated to reflect the cut-off for the most current Development Safety Update Report for LMTM (October 2013).	Section 2.3.3 Safety
Exclusion Criteria	
Exclusion criterion regarding the use of drugs known to pose a risk of methemoglobinemia at approved doses has been clarified to indicate that drugs for which there is a warning or precaution in the labeling about methemoglobinemia at approved doses are exclusionary.	Synopsis<br>Section 5.2 Exclusion Criterion No. 18
Study Drug Administration	
Dose reduction, if warranted, is to be accomplished by omission of either the morning or evening dose, resulting in once daily dosing of one study drug tablet (*i.e.,* 200 to 100 mg/day LMTM, or placebo equivalent). This replaces previous language delineating two 50-mg dose reduction steps (from 200 to 150 mg/day and 150 to 100 mg/day). Alternative study drug packaging and the 75-mg LMTM tablet strength for dose reduction will no longer be supplied.	Synopsis<br>Section 4.1 General Description<br>Section 4.6 Study Treatment<br>Section 6 Treatments Administered<br>Section 6.3.2 Dose Reduction
Safety Assessments / Procedures	
In cases where ARIA is noted, scans are to be repeated every 6 weeks to follow resolution/stabilization of ARIA; it is clarified that stabilization is to be determined based on at least three follow-up scans.	Synopsis<br>Section 4.4 Schedule of Assessments<br>Section 6.3.3.3 ARIA<br>Section 9.1.3.2 Brain MRI<br>Section 9.1.6 Site Review
Methemoglobin readings are to be repeated in response to values that are > 2.0%. The procedure for obtaining repeat readings has been clarified; specifically, two immediate repeat measurements should be obtained such that a total of three readings are obtained one on each of three different fingers (index, middle, ring) at a single visit. The mean of the three readings will be calculated automatically in the clinical database and used as the basis for safety monitoring decisions. All individual and calculated mean readings will be captured in the clinical database and included in the eCRF.	Section 6.3.3.1 Methemoglobinemia and/or Hemolytic Anemia<br>Section 8.4 Pulse Co-Oximetry

Summary of Changes	Affected Sections in Revised Protocol (Version 5.0)
The description of methemoglobin values that are to be considered AESIs has been clarified to indicate that only those values emergent after screening are to be considered AESIs, and whether or not the values are considered SAEs depends on medical and scientific judgment.	Section 8.1.3 Adverse Events of Special Interest
The description of other medically significant events that are to be considered SAEs has been clarified to indicate that only those events that are emergent after screening are to be considered SAEs; with respect to suicidality, serotonin toxicity and ARIA, only post-randomization cases are to be reported as per the procedure for SAEs.	Section 8.1.4 Serious Adverse Events<br>Section 8.8 Serotonin Toxicity (Syndrome)<br>Section 8.9 Columbia-Suicide Severity Rating Scale (C-SSRS)
Additional guidance is provided regarding assessment and screening of Heinz bodies to indicate that blood samples should be as fresh as possible at the time of slide preparation.	Section 8.3.2 Hematology
Imaging Procedures	
In addition to the protocol allowing FDG-PET scans to be repeated for the initial Screening/Baseline scan, clarification has been added that the Week 39 FDG-PET scan may also be repeated if the central reader determines a quality issue; however, the Week 78 FDG-PET scan cannot be repeated.	Section 4.4 Schedule of Assessments<br>Section 9.1.3.1 FDG-PET
As the protocol now allows for up to 5 scans (rather than 4 as previously written), the maximum estimated radiation exposure for FDG-PET scans over the course of the study has increased from 18.8 to 23.5 mSV.	
Use of sedatives prior to FDG-PET scans is no longer permitted.	Section 4.7.4 Drugs Used to Manage Behavioral Disturbance<br>Section 4.7.5 Other Medications<br>Section 9.1.3.1 FDG-PET
Other Assessments / Procedures	
Clarification has been added that the C-SSRS and Serotonin Toxicity Assessment may be rated by Rater 1 (the rater not involved with ADCS-CGIC), in addition to the safety rater.	Synopsis<br>Section 4.4 Schedule of Assessments<br>Section 7.1 Raters<br>Section 9.2 Resource Utilization in Dementia Questionnaire (RUD Lite)
The procedure for blood sample collection for analysis of MT concentrations has been modified. A suitable vacutainer, as defined in the laboratory manual, will be used for blood sample collection (no longer specified in the protocol as a glass sodium heparin vacutainer). All 6 mL of whole blood will now be centrifuged; the separated plasma (2 × 1-mL and 2 × 0.5-mL samples) will be transferred into four Nunc polypropylene tubes. The 2 × 0.5-mL samples will serve as back-up samples for analysis of MT concentrations.	Section 9.4.1 Procedure for Blood Sample Collection
The description of quality assurance and clinical monitoring has been revised to refer to the clinical monitoring plan for further details regarding the average monitoring frequency.	Section 17 Quality Assurance and Clinical Monitoring

24.4.9. Protocol Version 6.0

The final protocol for Study TRx-237-005 (Version 5.0 dated 15 May 2014) has been revised (Version 6.0 dated 4 June 2015) to include modifications and/or clarifications to administrative and background information, study objectives and efficacy/statistical analyses, study drug administration and storage conditions, safety assessments and other procedures. The revisions and affected sections are summarized in the table below.

Additional revisions are included which are editorial and/or intended to add minor clarification.

Summary of Changes	Affected Sections in Revised Protocol (Version 6.0)
Administrative / Background	
The registered address (in Singapore) for the Sponsor has changed and has been updated accordingly.	Title Page
The Sponsor's Global Project Lead has changed for this study and has been updated accordingly. Information has also been updated for Pharmacovigilance (new contact at WCT) and Imaging (addresses and/or telephone numbers have been updated for BioClinica and RadMD, and BioClinica is now identified as responsible for analyses of MRI data).	Section 1.2 Responsible Personnel
The background discussion of clinical data has been updated to reflect the most current Investigator's Brochure and Development Safety Update Report for LMTM.	Section 2.3.2 Efficacy<br>Section 2.3.3 Safety<br>Section 23 References
Objectives and Efficacy / Statistical Analyses	
The primary, secondary, and exploratory endpoints and statistical analyses have been modified. ADCS-ADL_{23} is a co-primary endpoint (previously secondary), ADCS-CGIC is a secondary endpoint (previously co-primary), reduction in decline in glucose uptake in the temporal lobe on FDG-PET imaging is an exploratory endpoint (previously primary), reduction in decline in WBV based on the BBSI by MRI imaging is a secondary endpoint (previously exploratory), and retardation in the rate of brain atrophy as shown by ventricular volume and hippocampal volume as evaluated by brain MRI imaging has been added as an exploratory endpoint. An Imaging MITT population (for analysis of change in WBV) is now defined and will include all randomized subjects who took at least one dose of study drug and have a Baseline and at least one post-Baseline MRI imaging measurement of adequate imaging quality. The sample size justifications for ADAS-cog_{11}, ADCS-ADL_{23}, and the MRI disease-modifying outcomes have also been modified based on the projected rate of placebo decline and the estimated effect size, and sample size calculations are now also discussed for ADCS-ADL_{23} and MRI.	Synopsis<br>Abbreviations<br>Section 2.4 Rationale for Study<br>Section 3.1 Primary<br>Section 3.2 Secondary<br>Section 3.3 Exploratory<br>Section 10.1.1 Primary Efficacy Endpoints<br>Section 10.1.2 Secondary Efficacy Endpoints<br>Section 10.1.3 Exploratory and Other Endpoints<br>Section 10.2.1 ADAS-cog_{11}<br>Section 10.2.2 ADCS-ADL_{23} (Section added)<br>Section 10.2.3 ADCS-CGIC (previously Section 10.2.2)<br>Section 10.2.4 MRI (Section added)<br>Section 10.3.1 Analysis Populations<br>Section 10.3.2 Efficacy Analysis (and all subsections)<br>Section 10.4 Interim Analyses<br>Section 23 References
Although routine brain MRI monitoring for ARIA is no longer required by FDA, MRI scans will still be obtained approximately every 13 weeks as these data will continue to be collected as secondary and exploratory efficacy endpoints. The protocol now identifies the responsible personnel for exploratory efficacy analyses of MRI and FDG-PET data. Both MRI and FDG-PET will be evaluated centrally (FDG-PET scans will be reviewed by an independent nuclear physician) to evaluate the effect of LMTM on modification of disease progression. Clarification has also been added that if MRI assessments are undertaken outside of the allowable window (± 14 days of the designated visit), these will be	Section 1.2 Responsible Personnel<br>Synopsis<br>Section 2.4 Rationale for Study<br>Section 4.4 Schedule of Assessments<br>Section 9.1.1 General Considerations<br>Section 9.1.3.2 Brain MRI<br>Section 9.1.4 Image Transfer and Quality Assurance<br>Section 9.1.5 Image Evaluation Procedure<br>Section 9.1.5.2 MRI Evaluation for ARIA (Section added)<br>Section 9.1.5.3 Efficacy Evaluations

Summary of Changes	Affected Sections in Revised Protocol (Version 6.0)
categorized/labelled according to the intended visit designation, regardless of being out of window.	
Screening MMSE (two levels: 20-23 and 24-26) is now included as a stratum to be included for the analysis of ADAS-cog_{11}, ADCS-ADL_{23}, and ADCS-CGIC.	Section 10.3.2.8.3 Subgroup Analyses
The definition of post-treatment adverse events has been expanded to also include events that increase in severity or relationship to study drug more than 7 days after the last dose of study drug.	Section 10.3.5.1 Adverse Events
A summary of the planned pharmacoeconomic statistical analyses has been added; a detailed description of the planned analyses, as well as intended sensitivity analyses, will be provided in the SAP.	Abbreviations<br>Section 10.3.7 Pharmacoeconomics<br>Section 23 References
Study Drug Administration and Storage	
The modified procedure for dose reduction incorporated in Protocol Version 5.0 dated 15 May 2014 has been reverted; two 50-mg dose reduction steps (from 200 to 150 mg/day and 150 to 100 mg/day) will continue to be implemented, and alternative study drug packaging and the 75-mg LMTM tablet strength will continue to be supplied for dose reduction in this study.	Synopsis<br>Section 4.1 General Description<br>Section 4.6 Study Treatment<br>Section 6 Treatments Administered<br>Section 6.3.1 Interruption<br>Section 6.3.2 Dose Reduction
The study drug storage temperature conditions have been modified based on satisfactory stability updates; study drug is to be stored at not more than 30°C (previously indicated as to be stored below 25°C).	Section 6.4 Packaging, Labeling, and Storage
Safety and Other Assessments / Procedures	
For ECG recordings to be obtained in triplicate, clarification has been added to indicate that the 2- to 5-minute interval is approximate.	Synopsis<br>Section 4.4 Schedule of Assessments<br>Section 5.2 Exclusion Criterion No. 13<br>Section 8.6 Electrocardiography<br>Section 24.1 Assessments by Visit
For MRI scans to be repeated every 6 weeks to follow resolution/stabilization of ARIA in cases where it is noted, stabilization is now indicated to be determined based on three follow-up scans (previously indicated to be based on at least three follow-up scans). Clarification has also been added that the results of reader assessments of MRI images (for evaluation of subject eligibility or ARIA) will be communicated to sites within 5 business days of image transfer to the imaging core laboratory (and resolution of any quality issues/queries).	Synopsis<br>Section 4.4 Schedule of Assessments<br>Section 6.3.3.3 ARIA<br>Section 9.1.5.1 Evaluation of Brain MRI for Subject Eligibility<br>Section 9.1.5.2 MRI Evaluation for ARIA (Section added)<br>Section 9.1.6 Site Review
Guidance has been added regarding categorization/labeling of assessments or visits that are performed outside of the stipulated time window and regarding unscheduled visits.	Section 4.4 Schedule of Assessments
Assessment of CDR total and MMSE is no longer specified to require assessors/raters who are not involved in the assessment of safety. CDR may also be undertaken at Screening by the ADCS-CGIC rater.	Section 4.4 Schedule of Assessments<br>Section 7.1 Raters
Due to lack of availability, change from immediate-release memantine to the extended release formulation (*e.g.*, from 10 mg twice daily to 28 mg once daily) is acceptable.	Section 4.7.1 Dementia Medication
Guidance regarding contraceptive measures now also indicates women of childbearing potential should be encouraged to return to the clinic in the event of a delayed menstrual period to rule out possible pregnancy.	Section 4.7.7 Contraceptive Measures
The discussion of AE recording has been modified to specify that when describing an AE in terms of a diagnosis, events leading up to a diagnosis should be retained.	Section 8.1 Adverse Events

Summary of Changes	Affected Sections in Revised Protocol (Version 6.0)
Clarification has been added for evaluation of sites' technical and personnel capabilities for imaging.	Section 9.1.2 Site Selection and Qualification
The location of the analytical laboratory responsible for the genotyping analysis, Athena Diagnostics, has been updated (now located in Marlborough, MA, USA).	Section 9.5 Genotyping

24.4.10. Protocol Version 7.0

The final protocol for Study TRx-237-005 (Version 6.0 dated 4 June 2015) has been revised (Version 7.0 dated 7 July 2015) to include updates to responsible personnel as well as modifications to the procedures for quality assurance and clinical monitoring. The revisions and affected sections are summarized in the table below.

Summary of Changes	Affected Sections in Revised Protocol (Version 7.0)
The Sponsor’s Head of Safety and Medical Monitoring has changed for this study and has been updated accordingly.	Section 1.2 Responsible Personnel
The Sponsor has appointed Exp-e-Data (UK) Ltd to provide and manage a secure data repository for the upload (as read-only PDFs) and storage of completed rating scales / questionnaires. The uploaded PDFs will be reviewed for the purpose of secondary monitoring to help assure data integrity.	Section 1.2 Responsible Personnel<br>Section 17 Quality Assurance and Clinical Monitoring
The Sponsor has appointed The University of Aberdeen Institute for Complex Systems and Mathematical Biology (ICSMB): (i) to automate reading of PDFs uploaded to the secure server and (ii) to assist with the provision of information to track progress of monitoring and data verification for Sponsor oversight.	Section 1.2 Responsible Personnel<br>Section 17 Quality Assurance and Clinical Monitoring
The Sponsor has appointed the Institute for Clinical Pharmacodynamics (ICPD) to undertake pharmacokinetic analysis.	Section 1.2 Responsible Personnel
