# Supplementary material for: Potential of Low Dose Leuco-Methylthioninium Bis(Hydromethanesulphonate) (LMTM) Monotherapy for Treatment of Mild Alzheimer’s Disease: Cohort Analysis as Modified Primary Outcome in a Phase III Clinical Trial
Source: J Alzheimers Dis. 2017 Nov 28;61(1):435–57. doi: 10.3233/JAD-170560 (PMC5734125; doi:10.3233/JAD-170560)
Supplement: Supplementary Material [file jad-61-jad170560-s003.pdf]

Statistical Analysis Plan

TauRx Therapeutics Ltd

TRx-237-005

Randomized, Double-Blind, Placebo-Controlled, Parallel-Group, 18-Month Safety and Efficacy Study of Leuco-methylthioninium bis(hydromethanesulfonate) in Subjects with Mild Alzheimer's Disease

VERSION 2.0 DATED 30 June 2016

**Sponsor:**	**TauRx Therapeutics Ltd**<br>**Liberty Building,**<br>**Foresterhill Road,**<br>**Aberdeen AB25 2ZP,**<br>**Scotland, UK**
**Prepared by:**	**Paul Pappas**<br>**SynteractHCR, Inc.**<br>**5759 Fleet Street, Suite 100**<br>**Carlsbad, CA 92008**
**Version 1.0 Date:**	**05 February 2016**

Approval

Upon review of this document, including table, listing, and figure shells, the undersigned approves the Statistical Analysis Plan. The analysis methods and data presentation are acceptable.

Signature	Date
______________________________<br>Professor Claude Wischik<br>Executive Chairman<br>TauRx Therapeutics Ltd.	____________
______________________________<br>Dr. Jiri Hardlund, CMO<br>VP Clinical Development<br>TauRx Therapeutics Ltd.	____________
______________________________<br>Charles Davis, Ph.D.<br>Biostatistics Consultant<br>CSD Biostatistics, Inc.	____________
______________________________<br>Paul Pappas<br>Senior Lead Biostatistician<br>SynteractHCR	____________

TABLE OF CONTENTS

LIST OF ABBREVIATIONS **6**
DEFINITIONS **9**
1. INTRODUCTION **11**
2. STUDY OBJECTIVES **11**
3. STUDY DESIGN AND PLAN **12**
4. DETERMINATION OF SAMPLE SIZE **15**
5. GENERAL ANALYSIS CONSIDERATIONS **15**
5.1 CONVENTIONS 16
5.2 STANDARD CALCULATIONS 17
5.3 HANDLING PARTIAL DATES 18
5.4 VISIT WINDOWS 19
5.5 REVIEW OF BLINDED DATA 19
6. ANALYSIS POPULATIONS **21**
7. STUDY POPULATION **22**
7.1 SUBJECT DISPOSITION 22
7.2 PROTOCOL DEVIATIONS 24
7.3 DEMOGRAPHIC AND BASELINE CHARACTERISTICS 24
7.4 MEDICAL HISTORY 25
7.5 PRIOR AND CONCOMITANT MEDICATIONS 25
8. EFFICACY ANALYSES **26**
8.1 EFFICACY VARIABLES 27
8.1.1 Primary Efficacy Variables 27
8.1.2 Secondary Efficacy Variables 27
8.1.3 Exploratory Efficacy Variables 27
8.2 ADJUSTMENTS FOR COVARIATES 28
8.3 HANDLING OF DROPOUTS OR MISSING DATA 29
8.4 INTERIM ANALYSIS AND DATA MONITORING 29
8.5 EXAMINATION OF SUBGROUPS 30
8.6 MULTIPLE COMPARISONS/MULTIPLICITY 31
8.7 MULTICENTER STUDIES 32
9. METHODS OF EFFICACY ANALYSIS **32**
9.1 PRIMARY EFFICACY ANALYSES 32
9.2 SECONDARY EFFICACY ANALYSES 36
9.2.1 Ventricular Volume 37
9.2.2 Integrated Analyses 39
9.2.3 ADCS-CGIC 41
9.2.4 MMSE, MADRS, and NPI 42
9.3 EXPLORATORY RESPONDER ANALYSES 42

9.3.1 ADAS-cog$_{11}$ Responder 43
9.3.2 ADCS-ADL$_{23}$ Responder 43
9.3.3 ADCS-CGIC Responder 43
9.4 SENSITIVITY ANALYSES 44
9.4.1 ADAS-cog$_{11}$ and ADCS-ADL$_{23}$ 44
9.4.2 ADCS-CGIC 47
9.5 ADDITIONAL ANALYSES 48
9.5.1 Analyses by Visit 48
9.5.2 Time to Event Analyses 49
9.5.3 Withdrawal and Rebound Effect 50
9.6 EXPLORATORY ANALYSES 50
9.6.1 FDG Temporoparietal Region Uptake and Relationship to MRI 50
9.6.2 Additional MRI Analyses 51
9.6.3 Voxel-Based Morphometry 52
9.6.4 Statistical Parametric Mapping for PET 52
9.6.5 Pharmacoeconomics 53
9.6.6 CSF Biomarkers 54
9.7 PHARMACOKINETIC ANALYSES 55
10. SAFETY ANALYSES 55
10.1 EXTENT OF EXPOSURE 56
10.2 ADVERSE EVENTS 58
10.2.1 Adverse Events of Special Interest 60
10.2.2 Selected Subsets of Adverse Events (Referred to as TauRx AE Groupings) 60
10.2.3 Subgroup Analyses 65
10.2.4 Time to Event Analyses of Adverse Events 65
10.3 CLINICAL LABORATORY EVALUATION 66
10.4 ADDITIONAL ANEMIA ANALYSES 68
10.5 PULSE CO-OXIMETRY 68
10.6 VITAL SIGNS 69
10.7 PHYSICAL AND NEUROLOGICAL EXAMINATION 70
10.8 ELECTROCARDIOGRAM 70
10.9 MRI DATA (BIOCLINICA) 72
10.10 SEROTONIN TOXICITY (SYNDROME) 73
10.11 COLUMBIA-SUICIDE SEVERITY RATING SCALE (C-SSRS) 73
11. CHANGES TO PROTOCOL-SPECIFIED ANALYSES 74
12. REFERENCES 75
APPENDICES 76
APPENDIX A: POST-BASELINE VISIT WINDOWS 76
APPENDIX B: CRITERIA FOR DETERMINING POTENTIALLY CLINICALLY SIGNIFICANT VALUES IN LABORATORY TEST RESULTS 77

APPENDIX C: CRITERIA FOR DETERMINING POTENTIALLY CLINICALLY SIGNIFICANT VALUES IN VITAL SIGNS 79
APPENDIX D: CRITERIA FOR IDENTIFYING ECG VALUES THAT REFLECT POTENTIALLY CLINICALLY SIGNIFICANT CHANGE 80
APPENDIX E: RULES FOR DETERMINING "WORST" VALUE 81
APPENDIX F: RULES FOR SCORING THE DISEASE ASSESSMENT SCALES 83
APPENDIX G: LIST OF REVIEW FILES PROVIDED BY TAURX TO SYNTERACTHCR 89

**LIST OF ABBREVIATIONS**	
AA	Alzheimer’s Association
AChEI	acetylcholinesterase inhibitor
ADAS-cog_{11}	Alzheimer's Disease Assessment Scale – Cognitive Subscale (11-item)
ADCS-ADL_{23}	Alzheimer’s Disease Cooperative Study – Activities of Daily Living
ADCS-CGIC	Alzheimer’s Disease Cooperative Study – Clinical Global Impression of Change
ADNI	Alzheimer’s Disease Neuroimaging Initiative
AE	adverse event
AESI	adverse event of special interest
ALT	alanine aminotransferase
ANCOVA	Analysis of covariance
ApoE	Apolipoprotein E
ARIA	amyloid related imaging abnormalities
AST	aspartate aminotransferase
ATC	Anatomical Therapeutic Chemical
BADL	Basic-ADL
BBSI	brain boundary shift integral
BMI	body mass index
BP	blood pressure
bpm	beats per minute
CBC	complete blood count
CDR	Clinical Dementia Rating
CK	creatine kinase
CI	confidence interval
cm	centimeter(s)
CDR	Clinical Dementia Rating
CrCl	creatinine clearance
CRF	case report form
CRO	clinical research organization
CSF	certain cerebrospinal fluid
CSR	clinical study report

LIST OF ABBREVIATIONS

C-SSRS	Columbia-Suicide Severity Rating Scale
DBP	diastolic blood pressure
DDE	drug dictionary enhanced
DSMB	Data and Safety Monitoring Board
ECG	Electrocardiogram
eGFR	estimated glomerular filtration rate
EMA	European Medicines Agency
FDA	Food and Drug Administration
FDG-PET	^{18}F-fluorodeoxyglucose positron emission tomography
g, kg, mg	gram(s), kilogram, milligram
G6PD	Glucose-6-phosphate dehydrogenase
GEE	Generalized Estimating Equation
GGT	gamma-glutamyl transpeptidase
HBSI	Hippocampal Boundary Shift Integral
HTLV-III	Human T-Cell Lymphocytic Virus Type III
HR	heart rate
ICF	Informed Consent Form
ITT	Intent-to-Treat
LDH	lactate dehydrogenase
LMTM	leuco-methylthioninium bis(hydromethanesulfonate)
LOCF	last observation carried forward
LZCF	last z-score carried forward
MADRS	Montgomery-Asberg Depression Rating Scale
MCV	mean cell volume
MedDRA	Medical Dictionary for Regulatory Activities
MetHb	Methemoglobin
MMSE	Mini-Mental State Examination
MRI	Magnetic Resonance Imaging
MT	Methylthioninium
NIA	the National Institute of Aging
NPI	Neuropsychiatric Inventory
PET	Positron Emission Tomography

LIST OF ABBREVIATIONS	
PP	Per Protocol
QTcB	Bazett's QT correction
QTcF	Fridericia's QT correction
RTF	rich text format
RUD	Resource Utilization in Dementia
SAE	serious adverse event
SAP	statistical analysis plan
SBP	systolic blood pressure
SD	standard deviation
SI	The International System of Units
SSRI	selective serotonin reuptake inhibitor
TEAE	treatment-emergent adverse event
TSH	thyroid stimulating hormone
VV	Ventricular Volume
WBV	whole brain volume
WHO	World Health Organization

DEFINITIONS

Adverse Event (AE)	An adverse event is any untoward medical occurrence in a patient or clinical investigation subject which does not necessarily have a causal relationship with treatment. An AE can therefore be any unfavorable and unintended sign (including an abnormal laboratory finding, for example), symptom, or disease, whether or not considered related to the medicinal product.
Adverse Events of Special Interest (AESI)	AESIs, as specified by the protocol, include:<br>• Methemoglobin values > 3.5% (confirmed on repeat result), signs or symptoms consistent with methemoglobinemia or hemolytic anemia, or observation of Heinz bodies<br>• A case meeting any one of the four criteria for serotonin syndrome<br>• Any possible case of amyloid related imaging abnormalities (ARIA)
Intent-to-Treat (ITT) Population	All randomized subjects
Modified Intent-to-Treat (MITT) Population	All randomized subjects who take at least one dose of the study drug and have both a baseline and at least one post-baseline efficacy assessment of ADAS-cog_{11} or ADCS-ADL_{23} prior to the 4-week post-dose follow-up assessment.
Per-Protocol (PP) Population	All MITT population subjects who do not have any important protocol deviations.
MRI Imaging Population	The MRI imaging population will include all subjects with a Screening/Baseline and at least one valid post-baseline volumetric assessment; subjects with an intercurrent medical event that could confound the volumetric measurement will be identified prior to unblinding and they will be excluded from the MRI imaging population.
PET Imaging Population	The PET population will include all subjects with a Screening/Baseline PET scan and at least one valid post-baseline volumetric assessment; subjects with an intercurrent medical event that could confound the PET measurement will be identified prior to unblinding and they will be excluded from the PET imaging population.

Safety Population	All randomized subjects who take at least one dose of study drug.
Serious AE (SAE)	A serious adverse event (SAE) is an adverse event that results in any of the following outcomes: death, life-threatening, persistent or significant disability/incapacity, requires or prolongs in-patient hospitalization, and congenital anomaly/birth defect. A medically significant AE is also an SAE.
Treatment-emergent AE (TEAE)	AEs with an onset time on or after the time of the initial dose of study drug or that worsen in intensity or treatment attribution.

1. INTRODUCTION

This document outlines the statistical methods to be implemented during the analyses of data collected within the scope of TauRx Therapeutics Ltd Protocol TRx-237-005 [Randomized, Double-Blind, Placebo-Controlled, Parallel-Group, 18-Month Trial of Leuco-methylthioninium bis(hydromethanesulfonate) in Subjects with Mild Alzheimer's Disease]. The purpose of this plan is to provide specific guidelines from which the analysis will proceed. Any deviations from these guidelines will be documented in the clinical study report (CSR).

2. STUDY OBJECTIVES

The primary objectives of this double-blind, placebo-controlled, parallel-group, randomized, 18-month, study of LMTM 8mg/ day given alone and 200 mg/day given alone in subjects with mild Alzheimer’s disease are stated below.

1. To demonstrate clinical efficacy of leuco-methylthioninium bis(hydromethanesulfonate) (also known as LMTM, TRx0237) in mild Alzheimer’s disease based on change from baseline on the following co-primary endpoints:
    - Alzheimer’s Disease Assessment Scale – Cognitive Subscale (ADAS-cog$_{11}$)
    - Alzheimer’s Disease Cooperative Study – Activities of Daily Living (ADCS-ADL$_{23}$)
2. To assess the safety and tolerability of LMTM 8mg/day as well as 200 mg/day given for up to 78 weeks

The secondary objectives are:

3. To further demonstrate disease modification based on the following key secondary endpoint:
    - Reduction of worsening of brain atrophy as measured by an increase in Ventricular Volume (VV) quantified by the ventricular boundary shift integral through MRI imaging
4. To evaluate the effect of LMTM on a global measure, Alzheimer’s Disease Cooperative Study – Clinical Global Impression of Change (ADCS-CGIC) - independently rated

5. To evaluate the effects of LMTM on other aspects of Alzheimer's disease including cognition (Mini-Mental Status Examination [MMSE]), behavior (neuropsychiatric inventory, NPI), and mood (Montgomery-Asberg Depressoin Rating Scale, MADRS)

The exploratory objectives are:

6. To determine the effects of LMTM on Alzheimer's disease modification by showing an effect on brain atrophy quantified by a reduction in Whole Brain Volume using change from baseline as measured by the brain boundary shift integral (BBSI) and decline in hippocampal and temporoparietal volume as evaluated by MRI
7. To evaluate the effect of LMTM on Alzheimer's disease modification as evidenced by reduction in decline in glucose uptake in the mean of left and right temporal lobes on ^{18}F-fluorodeoxyglucose positron emission tomography (FDG-PET) imaging
8. To determine the effects of LMTM on resource utilization using the Resource Utilization in Dementia (RUD) Lite
9. To explore changes in certain cerebrospinal fluid (CSF) biomarkers of Alzheimer's disease (total tau, phospho-tau, and Aβ1−42) in subjects who are mentally capable of providing their own separate informed consent and specifically agree to have lumbar puncture performed
10. To explore the influence of the Apolipoprotein E (ApoE) genotype on the primary and selected secondary outcomes (in subjects by or for whom lgally acceptable consent is provided)

3. STUDY DESIGN AND PLAN

This study is a multinational, randomized, placebo-controlled, double-blind, parallel-group, 78-week outpatient study with eight post-baseline on-treatment visits planned (Visits 3 -10), followed by an off-treatment follow-up for all subjects to occur 4 weeks after completion of randomized treatment (Visit 11) in subjects with mild Alzheimer's disease (Clinical Dementia Rating (CDR) global score of 0.5 or 1 and Mini-Mental State Examination (MMSE) of 20 to 26, inclusive).

The study is being conducted at approximately 108 study sites in the Americas (US, Canada), Europe, and Australia.

Eligibility for enrollment is assessed initially at one or more Screening visits, which are to occur within 6 weeks (42 days) of Baseline. A centrally-read MRI of the brain is included as part of Screening.

Subjects enrolled into this study are to have a diagnosis of dementia and probable Alzheimer's disease according to the criteria of the National Institute of Aging (NIA) / Alzheimer's Association (AA). Subjects are to have Alzheimer's disease of mild severity, as indicated by a Clinical Dementia Rating (CDR) total score (sometimes referred to as global score) of 0.5 or 1 and a Mini-Mental State Examination (MMSE) score of 20 to 26 (inclusive) at Screening (Visit 1).

Eligible subjects are randomly assigned in a 1:1 ratio at the Baseline visit to either the LMTM 200 mg/day group, or the placebo group. A central randomization list is used, with the randomization scheme stratified by use of AChEI and/or memantine (current ongoing use or not ongoing use), geographic region (the Americas, Europe/Australia), and screening severity (CDR of 0.5 or 1).

Baseline efficacy and safety assessments as well as exploratory imaging for disease modification are obtained prior to the start of treatment and repeated at designated visits throughout the treatment period. The CDR total score and MMSE are rated at Screening (Visit 1). The efficacy assessments, *i.e.*, the ADAS-cog_{11}, ADCS-ADL_{23} and ADCS-CGIC are rated at Baseline (Visit 2, pre-dose) for enrolled subjects and repeated on-treatment at Weeks 13, 26, 39, 52, 65, and 78 (Visits 5, 6, 7, 8, 9 and 10 or earlier, upon study discontinuation) by assessors/raters who are not involved in the assessment of safety. The ADCS-CGIC can only be rated by an independent rater not involved in other efficacy or safety assessments (Baseline efficacy assessments can be made on the day before randomization and dosing.) The MMSE, NPI and MADRS are rated after 26, 52, and 78 weeks of treatment. All, except NPI and MADRS are repeated at the 4-week off-treatment follow-up visit (Visit 11).

The pharmacoeconomic assessment using the RUD Lite is rated at Baseline (Visit 2, pre-dose), and after 26, 52, and 78 weeks of treatment.

Brain MRI (obtained approximately every 13 weeks) will be evaluated to determine whether there is a change in brain atrophy rates as quantified by a reduction of whole brain, volume, an increase in ventricular volume and a reduction in hippocampal and temporoparietal atrophy rates over the period of the study. FDG-PET is to be performed within the 42 days before Baseline and after 39 and 78 weeks of treatment. The purpose is to assess reduction in decline of glucose uptake in the temporal lobes. Change in whole brain, ventricular, hippocampal and temporoparietal volumes are to be quantified at the

imaging core laboratory. FDG-PET data is to be evaluated by an independent, nuclear physician who is experienced in neuro PET, not involved in the clinical conduct of the study, and trained on the study endpoints; these are further described in Section 9.1 of the protocol.

Safety assessments are performed throughout study participation, including at Visit 2 (prior to dosing and during the 4-hour post-dose evaluation) and during the treatment period. Study visits during the treatment period are to occur at time points approximately 2, 6, 13, 26, 39, 52, 65, and 78 weeks after Baseline. At each in-clinic visit, adverse events (AEs) are to be recorded, vital signs measured, ECGs obtained, targeted physical and neurological examinations performed, clinical laboratory testing (*e.g.*, hematology, serum chemistry panels, urinalysis, Vitamin B_{12}, and folate) performed, Columbia-Suicide Severity Rating Scale (C-SSRS) rated, potential for serotonin toxicity assessed, serum pregnancy test performed (women of childbearing potential only) and MetHb and oxygen saturation measured by pulse co-oximetry. These assessments are also conducted at a 4-week post-treatment follow-up visit (Visit 11). TSH is to be measured after 26, 52, and 78 weeks of treatment with a thyroid hormone panel obtained in the event of abnormality. Subjects are also to be followed for occurrence of ARIA by MRI performed after 13, 26, 39, 52, 65, and 78 weeks of treatment. If ARIA is reported, subjects are to be re-scanned every 6 weeks after discontinuation of study drug until imaging abnormalities are resolved or stabilized (based on at least three follow-up scans).

During intervening times, caregivers are to be contacted by telephone at approximately 9, 19, 32, 45, 58, and 71 weeks. Additional telephone contacts are to occur in subjects entering the study on serotonergic medication; in addition to in-clinic observation, caregivers are to be contacted by telephone at 5–7, >7–14, >14–24, 44-52, and 68-76 hours after the first dose of study drug (with a minimum of 1 hour between contacts).

CSF biomarkers are to be explored for a subset of subjects who are mentally capable of providing their own separate informed consent and specifically agree to have lumbar puncture performed. Baseline CSF samples may be collected any time prior to the first dose of study drug so long as all screening procedures have been completed and subject eligibility and willingness to continue have been confirmed. A subsequent sample is to be collected at the end of treatment (Week 78 or upon early termination).

Apolipoprotein E (ApoE) genotype also is to be determined for subjects for whom legally acceptable consent is obtained. A single blood sample may be collected any time after eligibility for randomization and continued participation in the study has been confirmed at Baseline (Visit 2).

Blood is to be collected for determination of MT concentrations prior to dosing (Visit 2) and approximately 3.5 hours after the first dose. Blood is also to be collected at all subsequent visits during the treatment period (after ECG recording). These collections are restricted to those sites with a refrigerated centrifuge and appropriate freezer capacity.

A schedule of assessments is listed in protocol Section 4.4.

The Treatment Period extends through Visit 10 (Week 78); subjects who discontinue study earlier are to have an Early Termination visit. Subjects who complete study through the final off-treatment follow-up visit (Visit 11) are to be offered an opportunity to transition directly into an open-label extension study (separate protocol).

4. DETERMINATION OF SAMPLE SIZE

It was planned that approximately 800 subjects are to be randomized in a 1:1 ratio to LMTM 200 mg/day, or placebo groups, respectively (400 subjects in LMTM group and 400 subjects for the placebo group).

5. GENERAL ANALYSIS CONSIDERATIONS

The statistical analyses will be reported using summary tables, figures, and data listings. Unless otherwise noted, all statistical testing will be two-sided and will be performed at the 0.05 significance level; for parallel tests the Bonferroni correction will be applied. Tests will be declared statistically significant if the calculated p-value (or Bonferrnoi corrected p-value) is < 0.05. Continuous variables will be summarized with means, standard deviations, medians, minimums, and maximums. Categorical variables will be summarized by counts and by percentage of subjects in corresponding categories.

All summary tables will be presented by randomized treatment group. Disposition, discontinuations, major protocol deviations, demographic and other baseline characteristics, medical history, and prior and concomitant medication summaries will also include a total summary column; i.e., both treatment groups pooled. Summary tables presenting results by study visit will include all scheduled study visits using informative visit labels (i.e., Baseline, Week 2, Week 6, Week 13, etc.).

Individual subject data obtained from the case report forms (CRFs), central clinical laboratory (local laboratory results are entered on the CRFs), central ECG readers, imaging core laboratories (MRI and FDG-PET), selected IWRS data, and any derived data will be presented by subject in data listings. Listings will include relative study day, where negative values will indicate pre-treatment visits, and data collected after discontinuation of study drug will be flagged as follow-up. All data captured on the CRF, including specific

descriptions of ‘other’ and comments fields, will be included on the listings. Listings will be sorted by subject number.

The analyses described in this plan are considered *a priori*, in that they have been defined prior to database lock and prior to breaking the blind. Any analyses performed subsequent to breaking the blind will be considered *post-hoc* and exploratory. *Post-hoc* analyses will be labeled as such on the output and identified in the CSR.

All analyses and tabulations will be performed using SAS Version 9.4 or higher. Tables, listings, and figures will be presented in RTF format. Upon completion, all SAS programs will be validated by an independent programmer. In addition, all program output will undergo a senior level statistical review. The validation process will be used to confirm that statistically valid methods have been implemented and that all data manipulations and calculations are accurate. Checks will be made to ensure accuracy, consistency with this plan, consistency within tables, and consistency between tables and corresponding data listings. Upon completion of validation and quality review procedures, all documentation will be collected and filed by the project statistician or designee.

5.1 Conventions

The precision of original measurements will be maintained in summaries, when possible. Means, medians and standard deviations will be presented with an increased level of precision; means and medians will be presented to one more decimal place than the raw data, and the standard deviations will be presented to two more decimal places than the raw data.

Summaries of continuous variables that have some values recorded using approximate values (e.g., < or >) will use imputed values. The approximate values will be imputed using the closest exact value for that measurement. For tables where rounding is required, rounding will be done to the nearest round-off unit. For example, if the round-off unit is the ones place (i.e., integers), values ≥ XX.5 will be rounded up to XX+1 while values < XX.5 will be rounded down to XX.

For percentages, unless they are calculated to be exactly 0% or 100%, values of very small or very large percentages will be reported as <0.1% and >99.9%.

For by-visit tables, percentages will be based on available data and denominators will generally exclude subjects with missing values. For frequency counts of categorical variables, categories whose counts are zero will be displayed for the sake of completeness.

For example, if none of the subjects discontinue due to “lost to follow-up”, this reason will be included in the table with a count of 0.

Wherever a calendar date is presented in a listing, the corresponding Study Day will be included.

5.2 Standard Calculations

Variables requiring calculation will be derived using the following formulas:

- **Baseline** - A baseline value, unless specified otherwise, is the last non-missing value recorded prior to the first dose of study drug. If an assessment has both a date and time that exactly match the date and time of first dose of study drug, the assessment will be counted as baseline.
- **Study day –** For a given date (*date*), study day is calculated as days since the date of first dose of study drug (*firstdose*):

  Study day = date – firstdose + 1, where date ≥ firstdose
  Study day = date – firstdose, where date < firstdose
- **Days –** Durations, expressed in days, between one date (*date1*) and another later date (*date2*) are calculated using the following formula: duration in days = (date2-date1+1).
- **Weeks –** Durations, expressed in weeks, between one date (*date1*) and another later date (*date2*) are calculated using the following formula: duration in weeks = (date2-date1+1)/7.
- **Months –** Durations, expressed in months, between one date (*date1*) and another later date (*date2*) are calculated using the following formula: duration in months = (date2-date1+1)/30.4375.
- **Years –** Durations, expressed in years, between one date (*date1*) and another later date (*date2*) are calculated using the following formula: duration in years = (date2-date1+1)/365.25.
- **Body Mass Index (BMI) -** BMI (kg/m^2) = weight (kg) / [[height (cm)/100]^2]
- **Estimated Glomerular Filtration Rate (eGFR) -**
  eGFR (mL/min/1.73 m^2) = 175 × (serum creatinine in mg/dL)$^{-1.154}$ × (Age in years)$^{-0.203}$ × (0.742 if female) × (1.212 if African American).

Note that age on consent date is collected on the CRF and will not be calculated.

5.3 Handling Partial Dates

If only a partial date is available and is required for a calculation, the following standards will be applied:

- Start dates
  a. For missing start day only - Day will be imputed as the first day of the month (i.e., 1) with the following exception: if the partial date falls in the same month and year as the date being used in the calculation (e.g., first dose date, informed consent date), then the partial date will be imputed to equal the date being used for the calculation.
  b. For missing start day and month - Day and month will be imputed as the first day of the year (i.e., 1 January) with the following exception: if the partial date falls in the same year as the date being used in the calculation (e.g., first dose date, informed consent date), then the partial date will be imputed to equal the date being used for the calculation.

- Stop dates
  a. For missing stop day only - Day will be imputed as the last day of the month (i.e., 28, 29, 30, or 31) or the last day of study contact if earlier
  b. For missing stop day and month - Day and month will be imputed as the last day of the year (i.e., 31 December) or the last day of study contact if earlier

In case the imputation rules above cause a contradiction, these will be resolved individually.

Completely missing start dates or end dates will be reviewed, and values will be imputed based on discussion at the blinded data review meeting. For tables and listings, ‘ongoing’ or ‘unknown’ will be used if appropriate and no date is available; if dates are missing, a missing code will be used. For numerical analysis, for end dates that are missing but are highlighted as ‘ongoing’, the last visit date will be used unless otherwise decided at the blinded data review meeting.

The date of last dose of study drug will be taken from the Study Exit Status CRF page whenever available. If this is missing, then the latest start date of AE with study drug action withdrawal is used. Otherwise, the last assessment date, e.g. date of vital signs or laboratory measurements, excluding the follow-up visit, or telephone contact date where study drug continuation is indicated, whichever is later, will be used. This treatment end date imputation was confirmed during the blinded data review, however manual review conclusions could override the algorithm’s determination.

Any partial dates will be displayed in data listings without imputation of missing days and/or months (e.g., MAR2011, 2009). Regardless, the imputed date will be used in the calculation of the corresponding Study Day, which is to be included in the listing.

5.4 Visit Windows

Screening / Baseline assessments can take place over 2 days. For tabular purposes, these will be considered as having occurred on the same day; listings will present the corresponding dates and study days.

Since study visits do not always take place exactly as scheduled in the protocol, it is necessary to assign the actual observation times to "analysis visit windows" for analysis purposes. Post-baseline visit windows for each target visit are defined in Appendix A, and will be applied to all (safety, efficacy, etc.) measurements. As an exception, Day 1 Pre-dose and Post-dose assessments will be considered separately and will not use visit windows; post-dose primary efficacy assessments on Day 1 are not treated as baseline – subjects with baseline primary efficacy assessments after the first dose on Day 1 are therefore excluded from the corresponding analysis. For each summary and value if there are multiple values within a visit window, the "worst" value as defined in Appendix E will be used for that visit window summary except for the efficacy endpoints where the rules are described in Section 9.1. These visit windows will be applied to all visits, including scheduled, unscheduled, and early termination visits. On-treatment measurements are defined as the post-dose measurements collected up to 14 days after the last dose date and prior to the 4-week follow-up visit.

5.5 Review of Blinded Data

A number of analyses will require review of blinded data. The following reviews will occur periodically while the study is ongoing, with a final blinded review occurring just prior to database lock:

- Assignment of subjects into the Per Protocol and Imaging Populations.
- Concomitant medications are reviewed independent of Subject ID to identify the following:
    - Medications with serotonergic potential (listed in the Investigator's Brochure, Attachment 4 or identified in literature searches performed subsequent to the IB approval date). TauRx will provide an Excel file for medications indicating serotonergic potential (see Appendix G).

  - Medical food (e.g., Axona, Souvenaid) or alternative (non-licensed) pharmacotherapy for dementia (e.g., Vitamin E, folate [in doses up to 5 mg/day; use of doses of 1 mg/day as supplementation is not included here], a specific neurocognitive vitamin formulation [such as NeuroVits comprising 20 mg Vitamin B_6, 1 mg Vitamin B_{12}, 0.8 mg folate (see Douaud et al., 2013)], ginkgo biloba, hormone replacement therapy, coconut oil, curcumin). (To be used for efficacy subgroup analysis, see Section 8.5.)
  - Any antipsychotic medication that has not been correctly flagged by site
  - Other medications or treatments of interest.
- On-treatment changes in anti-dementia medications (licensed as well as non-licensed).
- Review of Day 1 adverse events (to confirm whether they are pre-dose or post-dose).
- Review of site’s classification of protocol-specified AESIs for correctness (only those listed in the protocol are to be flagged in the database as such (see Section 10.2.1). Each potential ARIA case will be confirmed and then categorized as vasogenic edema (ARIA-E), hemorrhagic ARIA (ARIA-H) or both.
- Review of adverse event summaries and listings to identify MedDRA preferred terms associated with the established TauRx AE Groupings of adverse event terms (see Section 10.2.2), as well as for other adverse event terms of possible medical interest for grouping.
- Review of protocol and consent deviations to identify major deviations that would exclude subjects from the per-protocol population.
- Review of subjects with a missing or partial last dose date on the Study Exit Status CRF in order to determine an appropriate imputed date or other missing or partial dates for a parameter of interest to determine an appropriate attribution to treatment (e.g., AEs, baseline values, etc.).
- Review of subjects' C-SSRS data together with AE reports of suicidal ideation or behavior to confirm all individual cases.
- Categories to be used to summarize “Leaving Full-Time Education” will be determined after blinded review of the frequency distribution. The categories to be used for age are: <17, 17 – 19, 20 – 24, and >24.

- Subjects with total scores that meet the definition for potential serotonin toxicity will have their scores reviewed to identify cases of treatment-emergent potential serotonin syndrome.
- Reasons for withdrawal per subject and categorization of these into potentially treatment and not-treatment related reasons.
- Confirmation of subjects who continued in the study off-treatment and the reason for stopping treatment

The following decision will be made at the blinded data review:

- Whether Week 65 or Week 78 will be the designated time point for the primary analysis based on the percentage of subjects with Week 78 efficacy assessments (if fewer than 60% of subjects are retained at Week 78, Week 65 will be used). The BDRM has confirmed that the designated timepoint for analysis is Week 78.

6. ANALYSIS POPULATIONS

The following subject populations will be used for efficacy analyses:

- Intent-to-Treat (ITT) population will include all randomized subjects. Treatment assignment will be based on the randomized treatment.
- Modified Intent-to-Treat (MITT) population will include all randomized subjects who take at least one dose of the study drug and have both a baseline and at least one post-baseline efficacy assessment in the Treatment Period (prior to the 4-week post-dose follow-up assessment). Treatment assignment will be based on the randomized treatment. The efficacy endpoints considered for the MITT definition are: ADAS-cog_{11} and ADCS-ADL_{23}.
- Per-Protocol (PP) population will include all MITT population subjects who do not have any important protocol violations that could affect the efficacy analysis. Important protocol violations will be determined prior to unblinding irrespective of their classification as minor/major/critical. Treatment assignment will be based on the randomized treatment.
- Subjects will be included in their randomized stratum as reflected by the IWRS rather than eCRF.

If subjects need to be excluded from the ITT and MITT populations because of significant non-compliance with GCP, a corresponding file note will document this. Primary efficacy data for these subjects will be summarized separately (mean and standard deviation per treatement group). Such subjects will be excluded from the PP population whether or not they are excluded from the ITT and MITT populations.

In addition, at sites where data, inclusive of safety data, are deemed unreliable due to significant GCP violations, all data will be excluded from analysis. (Failure to obtain Investigator signature in advance of the data cut-off date is one such example that will result in exclusion of all data.) For completeness, safety data will be provided in an appendix to the study report, however, these data will not be included in electronic analysis datasets.

The MRI imaging population will include all MITT subjects with a Screening/Baseline and at least one valid post-baseline volumetric assessment; subjects with an intercurrent medical event that could confound the volumetric measurement will be identified prior to unblinding and they will be excluded from the MRI imaging population.

The PET population (exploratory analysis) will include all MITT subjects with a Screening/Baseline PET scan and at least one valid post-baseline volumetric assessment; subjects with an intercurrent medical event that could confound the PET measurement will be identified prior to unblinding and they will be excluded from the PET imaging population. The BDRM has confirmed that PET population is identical to the MITT population.

The Pharmacokinetic (PK) population will include any subject who received study drug and also had a post-dose PK sample drawn. Treatment assignment will be based on the treatment actually received.

The Safety population will include all randomized subjects who take at least one dose of study drug. Treatment assignment will be based on the treatment actually received; in the event of dose reduction, a subject will be categorized based on the original dose for purposes of tabular summaries. If a subject receives an incorrect treatment transiently, they will be assigned to the predominant treatment group (i.e., the treatment group for which they received the greatest number of doses).

7. STUDY POPULATION

7.1 Subject Disposition

Subject disposition information will be summarized for all subjects in the ITT population by treatment group, overall and by region, and country. Summaries will include: the number of randomized subjects, the number of subjects in each analysis population, the number of subjects completing the treatment period (subsets on and off treatment), the number of subjects completing the safety follow-up visit, the number of subjects completing the study (and subsets completing the study on and off treatment), all reasons

for discontinuation of study drug and study, and the primary reason for discontinuation of study.

The visit schedules and their timing are given in the following table as given in Section 4.4 of the protocol:

<table>
<tr><td rowspan="2">Visit Name:</td><td rowspan="2">Screening</td><td rowspan="2" colspan="2">Baseline
Day 1</td><td colspan="8">Treatment Period (78 Weeks)</td><td rowspan="2">*Safety Follow-up*</td></tr>
<tr><td colspan="7"></td><td>EOS (ET)</td></tr>
<tr><td>Overall Visit Number:</td><td>1</td><td colspan="2">2</td><td>3</td><td>4</td><td>5</td><td>6</td><td>7</td><td>8</td><td>9</td><td>10</td><td>11</td></tr>
<tr><td>Weeks Relative to Baseline Day:</td><td rowspan="3">≤ 42</td><td rowspan="3">*Pre-Dose*</td><td rowspan="3">*Post-Dose*</td><td>2</td><td>6</td><td>13</td><td>26</td><td>39</td><td>52</td><td>65</td><td>78</td><td>82</td></tr>
<tr><td>Scheduled Study Day:</td><td>15</td><td>43</td><td>92</td><td>183</td><td>274</td><td>365</td><td>456</td><td>547</td><td>575</td></tr>
<tr><td>(Allowable Time Window):</td><td>(±3)</td><td>(±3)</td><td>(±7)</td><td>(±14)</td><td>(±14)</td><td>(±14)</td><td>(±14)</td><td>(±14)</td><td>(±7)</td></tr>
</table>

The timing of the early discontinuations of study drug (for Safety Population) and study participation in the Treatment Group (for the ITT Population) will each be assessed by summarizing discontinuations by reason during each of the following time periods (Appendix A), overall and by region:

**Category of Exposure (days)**
1
2-28
29-67
68-137
138-228
229-319
320-410
411-501
>501

Subjects terminating in the follow-up period will be summarized separately.

7.2 Protocol Deviations

In accordance with ICH E3, Sponsor-defined eligibility deviations and post-randomization protocol deviations will be identified and listed separately by subject. Sources for these deviations may include IWRS, CTMS (Clinical Trial Management System) and the clinical database. Deviations will be classified as follows (also see Appendix G):

Deviation type/code as provided by Sponsor includes but is not limited to the following:

- Informed consent
- Randomization error
- Safety
- MRI
- Efficacy
- IP / Treatment deviation
- Other protocol deviations

Deviation are then categorized into:

- Critical
- Major
- Minor

Should there be a for-cause reason to disqualify data from any non-compliant site, a Note-To-File letter, signed by the Sponsor, will be provided to SynteractHCR Biostatistics to do so. This Note-To-File letter should include description of the event/reason and an unbiased assessment of impact. These are considered as a major deviation and will be excluded from PP population.

A tabulation of protocol deviations by type/code overall and categorization will be provided. A by-subject listing of deviations will also be provided.

7.3 Demographic and Baseline Characteristics

Demographic variables to be summarized include the following: age at informed consent, gender, ethnicity, race, and geographic region.

General baseline characteristics to be summarized include the following: height, weight, body mass index (BMI), creatinine clearance, eGFR, whether or not the subject provided his/her own informed consent, and age at leaving full-time education.

Disease specific baseline characteristics include: time from diagnosis of Alzheimer's disease to informed consent (years), ApoE genotype (in the subset who provide consent), CSF biomarkers (total tau, phospho-tau, and $A\beta_{1-42}$), mini-Mental State Examination (MMSE), Clinical Dementia Rating (CDR) overall score, use of an acetylcholinesterase inhibitor (AChEI) and/or memantine, disease severity, use of a selective serotonin reuptake inhibitor (SSRI), use of drugs of serotonergic potential, and use of medical food or alternative pharmacotherapy for dementia.

Demographic and baseline characteristics will be summarized for all the analysis populations. Summaries will also be provided by region, country and site, for the ITT population.

Additional demographic and baseline characteristics will be presented in a listing only, including childbearing status (for females), main method of adequate contraception, occupation, occupation and social class (if available), and handedness. The occupation and social classes are: managerial, professional, lesser professional, secretarial, skilled manual, semi-skilled ii, semi-skilled i, unskilled ii, and unskilled i (Great Britain: Office of Population Censuses and Surveys, 1990).

Subjects who are mis-stratified at the time of randomization will be flagged in the listing.

7.4 Medical History

Verbatim terms on CRFs will be mapped to preferred terms and system organ classes using the Medical Dictionary for Regulatory Activities (MedDRA) (version 16.0).

The number and percentage of subjects with a given medical history will be summarized for each system organ class, high level group terms, and preferred terms to be defined. Summaries will be provided for the Safety Population. The verbatim and coded medical histories will be included in a listing.

7.5 Prior and Concomitant Medications

Verbatim terms on CRFs will be mapped to Anatomical/Therapeutic/Chemical (ATC) class and Generic Drug Names using the World Health Organization (WHO) dictionary (WHODDE B2, March 1, 2013 release).

Prior medications are those medications discontinued before the start of study drug. Concomitant medications are those medications taken at the start of study drug or initiated after the initial dose of study drug. Prior medications and concomitant medications will be summarized separately for each treatment by WHO ATC level 1 term, ATC level 3 term and Preferred Term (generic name) with frequency and percentage of subjects in each dosing arm using each prior medication and each concomitant medication. For concomitant medications, a separate tabulation will be made of those medications used at the start of study drug. At each level of subject summarization, a subject is counted once if he/she reported one or more medications at that level. Each summary will be ordered alphabetically by each level of ATC class and generic drug name within each level of ATC class. Prior and Concomitant medications will also be listed separately with these elements as well as the verbatim drug name. Those medications that are initiated after the last dose of study drug will be flagged.

In addition, tabulations and listings will be provided for the following subsets of concomitant medications (other groupings of interest, if applicable):

- Medications with serotonergic potential (listed in the Investigator's Brochure, Attachment 4 or identified in literature searches performed subsequent to the IB approval date)
- Newly initiated anti-psychotic medications (on-treatment initiation - identified on the concomitant medications eCRF along with the specific behavioral reason for use)
- Anti-dementia medications (licensed as well as non-licensed)

The summary of prior medications and the overall summary and subsets of concomitant medications will be provided for Safety population.

For subjects on digoxin, available plasma concentrations of digoxin obtained from the local laboratory will be included in a listing.

8. EFFICACY ANALYSES

For the FDA, the primary analyses will be based on the MITT population; for EMA the primary efficacy analysis will be based on the ITT population. Supportive efficacy analyses will be performed using the PP population. Additional sensitivity analyses will be performed using the the MITT population, the ITT population, a ‘completer population’ (all subjects excluding those that have not completed the study on treatment – exclusive of follow up), and/or the PP population.

8.1 Efficacy Variables

8.1.1 Primary Efficacy Variables

The co-primary efficacy endpoints for the clinical demonstration of efficacy are the following:

- Change from Baseline to Week 78 (Week 65 if fewer than 60% of subjects are retained at Week 78) in ADAS-cog_{11}
- Change from Baseline to Week 78 (Week 65 if fewer than 60% of subjects are retained at Week 78) in ADCS-ADL_{23}

The Alzheimer's Disease Assessment Scale – Cognitive Subscale is a global cognitive measure. The ADAS-Cog_{11} total score is defined as the sum of the scores from 11 individual items. Scoring details are provided in the Administration and Scoring Manual: Alzheimer's Disease Assessment Scale – Cognitive (ADAS-cog11), 2012. Its value can range from 0 to 70. The handling of partial data is described in Appendix F.

The ADCS-ADL_{23} includes 23 items that were derived from a larger set of items describing performance of activities of daily living (ADL) by Alzheimer's disease subjects. This scale ranges from 0 to 78, with lower scores suggesting greater functional impairment. The scoring algorithm is described in Appendix F.

8.1.2 Secondary Efficacy Variables

The following are the secondary clinical efficacy endpoints:

- Ventricular Volume using change from baseline in VBSI as measured by MRI imaging (key secondary endpoint utilized to demonstrate a disease modifying effect)
- ADCS-CGIC
- MMSE
- MADRS
- NPI

8.1.3 Exploratory Efficacy Variables

The following endpoints will be considered in an exploratory fashion:

- MRI: Change in whole brain, hippocampal and temporoparietal volume. Other

regional differences using whole brain statistical mapping techniques making no assumptions about the form or location of treatment differences, using segmented T1 volumetric MR images.

- FDG-PET: Change in glucose uptake to the temporoparietal lobes and other parameters as using pre-defined volume-of-interest extracted values. Other regional differences identified using whole brain statistical mapping making no assumption about the form or location of any differences, using the FDG-PET images.
- RUD Lite
- CSF biomarkers of Alzheimer's disease

Cerebrospinal fluid (CSF) samples will be collected at designated study sites for a subset of subjects. The following CSF biomarkers will be assessed:

- total tau
- phospho-tau
- $A\beta_{1-42}$
- $A\beta_{1-42}$ / phospho-tau and $A\beta_{1-42}$ / total tau ratios

Blood will be collected (in subjects by or for whom legally acceptable consent is provided) to explore the influence of Apolipoprotein E (ApoE) genotype on the primary and selected secondary endpoints. In sensitivity analyses only, ApoE will be used to determine whether inclusion as a covariate (presence or absence of the ApoE 4 allele) influences treatment effect analyses. In exploratory analyses, ApoE subgroups will be analyzed according to number of ApoE 4 alleles.

Blood will also be collected for purposes of population pharmacokinetic analysis. This will be detailed in a separate Statistical Analysis Plan (SAP) and will be reported separately.

8.2 Adjustments for Covariates

The models for the ADAS-cog_{11}, ADCS-ADL_{23}, ADCS-CGIC, MMSE, NPI and MADRS will include adjustments for the following covariates (based on the randomization strata): disease severity as measured by CDR (two levels), and geographic region (2 levels: the Americas, and Europe/Australia). Geographic region will not be included for imaging parameters.

AChEI and/or memantine status at randomization (two levels: current ongoing use or not ongoing use) is considered an important factor, such that it will be included unless stated otherwise as two interaction terms: AChEI and/or memantine status times visit as well as AChEI and/or memantine status times treatment (8mg/day LMTM, 200mg/day LMTM)

The models for ADAS-cog_{11}, ADCS-ADL_{23}, MMSE, NPI and MADRS will also include the baseline value as a covariate.

8.3 Handling of Dropouts or Missing Data

Efficacy data missing for an entire outcome scale or for the majority of the scale (as indicated below) will not be imputed for the primary analysis of ADAS-cog_{11} and ADCS-ADL_{23}. Instead, it will be assumed that the data are close to missing at random after accounting for the terms in the model. However, missing items within a scale may be imputed if some items of the scale are present. The handling of partial data for the efficacy endpoints are described in Appendix F.

Additional sensitivity analyses of ADAS-cog_{11} and ADCS-ADL_{23} will be carried out to assess the impact of missing data including last z-score carried forward (LZCF), last observation carried forward (LOCF), and multiple imputation as detailed in Section 9.4.

For ADCS-CGIC, last observation carried forward (LOCF) and worst observation carried forward (WOCF) will be used to impute missing values complemented by analysis without imputation.

8.4 Interim Analysis and Data Monitoring

The data to be provided to the Date Safety Monitoring Board (DSMB) are described in the DSMB charter and a separate SAP.

There are no interim efficacy analyses planned for this study.

Recruitment and discontinuations will, however, be continuously monitored in a blinded fashion and projections on future dropouts calculated in order to check whether the 40% withdrawal assumption from the sample size calculation holds. Should the overall dropout for the duration of the study be projected to exceed 40%, then the number of subjects to be enrolled may be increased in order to power an analysis at 65 or 78 weeks.

8.5 Examination of Subgroups

Subgroup analyses also will be performed for ADAS-cog11, ADCS-ADL23 based on the MITT population as well as VV for the imaging population by repeating the analysis restricted to the subgroup and by adding interaction terms of the form characteristics of the subgroup times treatment allocation as covariates to test overall interaction for significance; the interaction term will not be tested at a particular visit, thus three-way interaction terms will not be used. The same types of analyses will be carried out for ADCS-CGIC using logistic regression models with the dependent variable being the proportion of subjects without moderate or marked decline, or without any decline (as defined in Section 9.3). The subject characteristics that will be included in this type of analysis are the following:

- Male and female
- Age < 75 and ≥ 75 years
- Screening MMSE 20-23 and 24-26
- Screening CDR 0.5 and 1 as randomized as well as actual CDR recalculated based on the subitem scores.
- Duration of AChEI and/or memantine use at baseline (more than 6 months, less than or equal to 6 months).
- Users and non-users of medical food (*e.g.*, Axona, Souvenaid) or alternative pharmacotherapy for dementia (*e.g.*, Vitamin E, folate [in doses up to 5 mg/day; use of doses of 1 mg/day as supplementation is not included here], a specific neurocognitive vitamin formulation [such as NeuroVits comprising 20 mg Vitamin B_6, 1 mg Vitamin B_{12}, 0.8 mg folate (see Douaud *et al*., 2013)], ginkgo biloba, hormone replacement therapy, coconut oil, curcumin) at randomization
- Renal function (as defined by creatinine clearance above and less than or equal to 50 mL/min)
- Geographic Region (the Americas, Europe/Australia) as well as Geographic Region/Language (the Americas, Europe/Australia English-speaking, and Europe/Australia non-English-speaking)
- Fazekas score (0 or 1 for both scores, 2 for either score). There are two Fazekas scores recorded in the MRI database, one for periventricular hyperintensity and one for deep white-matter hyperintensity.
- ApoE genotype (presence or absence of the E4 allele) for subjects for whom legally acceptable consent is obtained. The ApoE genotype data is contained in the

Covance lab dataset. Subjects can have 0, 1, or 2 copies of the E4 allele, so presence of the E4 allele is defined as either 1 or 2 copies.

Age will also be added as a continuous variable to test whether treatment effect differs linearly with baseline age.

The interaction terms added will be of the form treatment by characteristic, a visit by characteristic term will also be added. In addition to the model-based analysis with interaction terms, a "simple" subgroup analysis will be reported, rerunning the primary analysis on each subgroup in turn.

The relationship between severity at baseline as defined by CDR or MMSE and treatment response on the ADAS-cog_{11} will be investigated by assessing the overall severity by treatment interaction effect in an additional model similar to the primary analysis model. Severity at Baseline will be defined by screening CDR (0.5 or 1) and also MMSE 20-23 or 24-26. In addition, the actual baseline MMSE score will also be tested in the ADAS-cog_{11} model in place of the categorical severity based on MMSE, and the interaction between this baseline score and treatment group will be assessed to determine whether there is a differing effect for subjects with differing baseline severity as indicated by higher or lower MMSE total score. This analysis will be repeated for ADCS-ADL_{23} and Ventricular Volume.

8.6 Multiple Comparisons/Multiplicity

P-values will be adjusted using Bonferroni correction for parallel comparisons; as a fixed sequence of tests will be used these corrected p-values are passed down to the next test without further corrections.

The fixed sequence of secondary testing will be:

- Brain MRI to evaluate change in VV, to support a disease modification claim
- ADCS-CGIC
- MMSE
- MADRS
- NPI

Further imaging results will be analyzed for inferential purposes to support a disease modification claim. The fixed sequence of testing will be:

- Brain MRI to evaluate change in WBV
- FDG-PET temporal lobe averaged bilaterally

- Brain MRI to evaluate change in hippocampal and temporoparietal volumes

Only the MRI VV endpoint will be required to establish disease modification.

If the primary analysis is deemed positive (both co-primaries need to reach statistical significance), then in the secondary analysis additional inferences will be studied using a fixed sequence of tests as defined above. Each test within this sequence will use a two-sided type I error of 0.05. Inferences will continue to be drawn until the first non-significant p-value is reached, at which point inference will stop. All tests refer to the Week 78 (Week 65 if fewer than 60% of subjects are retained at Week 78) change value.

8.7 Multicenter Studies

Because the study will include a large number of study sites relative to the total number of subjects, the effect of study site will not be included in the statistical analysis models. However, geographic region (2 levels: the Americas, Europe/Australia) will be included as a stratification factor for efficacy analyses. Subgroup analyses will also be performed by a variable combining geographic region and language (3 levels: the Americas, Europe/Australia English-speaking, Europe/Australia non-English-speaking). Please refer to Section 8.2 for detailed specifications of the covariates.

9. METHODS OF EFFICACY ANALYSIS

9.1 Primary Efficacy Analyses

For each of two treatment comparisons (200mg/day LMTM given alone and 8mg/day LMTM given alone), the study has two co-primary endpoints for purposes of demonstrating clinical efficacy in the treatment of Alzheimer's disease: ADAS-cog_{11} and ADCS-ADL_{23}. Both clinical endpoints in each treatment comparison must reach significance based on the use of a two-sided test at the alpha=0.025 level of significance for LMTM to be designated as superior to placebo for that treatment comparison.

There are four primary null hypotheses to consider:

H_{01}: There is no difference between LMTM 200 mg/day given alone and placebo (as randomized) in change from baseline ADAS-cog_{11} at Week 78 (Week 65 if fewer than 60% of subjects are retained at Week 78)

H_{02}: There is no difference between LMTM 200 mg/day given alone and placebo (as randomized) in change from baseline ADCS-ADL_{23} at Week 78 (Week 65 if fewer than 60% of subjects are retained at Week 78)

H_{03}: There is no difference between LMTM 8 mg/day given alone and 8 mg/day given together with AChEI and/or memantine in change from baseline ADAS-cog_{11} at Week 78 (Week 65 if fewer than 60% of subjects are retained at Week 78)

H_{04}: There is no difference between LMTM 8 mg/day given alone and 8 mg/day given together with AChEI and/or memantine in change from baseline ADCS-ADL_{23} at Week 78 (Week 65 if fewer than 60% of subjects are retained at Week 78)

For LMTM 200 mg/day to be designated as superior to placebo as randomized, both H_{01} and H_{02} need to be rejected at an alpha of 0.025. For LMTM 8 mg/day given alone to be designated as superior to 8 mg/day given together with AChEI and/or memantine, both H_{03} and H_{04} need to be rejected at an alpha of 0.025.

For the FDA, the primary analyses will be based on the MITT population. For the EMA, the primary analyses will be based on the ITT population. For each summary and value if there are multiple values within a visit window, the value whose date is closest to the scheduled date shall be used. In the case that more than one value is equidistant from the scheduled date, the later will be used. A post-baseline on-treatment assessment is defined as those recorded up to 14 days after the last dose date and prior to the 4-week post-dose follow-up assessment.

The primary analysis specification refers to "number of subjects retained at Week 78". We define "number of subjects retained at visit X" as follows: If a subject has an on-treatment assessment with a non-missing score for either ADAS-cog_{11} or for ADCS-ADL_{23} at visit X, or at any scheduled visit subsequent to X, then this subject is defined to be retained at visit X. Otherwise the subject is defined to be not retained at visit X. Note that this is a per-subject definition, not a per-endpoint definition.

For the ADAS-cog_{11}, summary statistics will be tabulated by visit and treatment group using observed data. Least squares means (marginal means), treatment differences in least squares means, and 95% confidence intervals will also be included.

For FDA, the primary analysis will be restricted to scheduled (Week 13, 26, 39, 52, 65, and 78) as specified in the window definitions in Appendix A) on-treatment visits. This analysis also forms the basis for the primary EMA intervention effect analysis as described below, which provides the outcomes for the ITT population. As an intermediate sensitivity analysis (described below), the MITT population, which includes any non-follow-up visit with a valid ADAS-cog_{11} or ADCS-ADL_{23} score, will also be analyzed.

Change from baseline in ADAS-cog$_{11}$ will be analyzed using a restricted maximum likelihood based repeated measures linear mixed model. The model will include fixed effects for treatment group (two levels), time (six levels, corresponding to Weeks 13, 26, 39, 52, 65, and 78), the treatment group by time interaction, AChEI and/or memantine status at randomization (two levels: current ongoing use or not ongoing use) by time interaction, AChEI and/or memantine status at randomization (two levels: current ongoing use or not ongoing use) by treatment group interaction, baseline severity (CDR, two levels) and geographic region (2 levels: the Americas, Europe/Australia). In addition, the baseline ADAS-cog$_{11}$ will be included as a covariate. An unstructured covariance model will be used. The Kenward and Roger method of calculating the denominator degrees of freedom will be used for the tests of fixed effects. Treatment comparisons will be based on the modeled change from Baseline at Week 78 (Week 65 if fewer than 60% of subjects are retained at Week 78). There will be no imputation for assessments for which ADAS-cog is not available; the handling of missing ADAS-cog$_{11}$ sub-items is discussed in Appendix F.

The primary analysis of ADCS-ADL$_{23}$ will be the same as for ADAS-cog$_{11}$ except that the repeated measures model will include the baseline ADCS-ADL$_{23}$ score as a covariate, in place of the baseline ADAS-cog$_{11}$ score.

The two treatment comparisons will be investigated in a parallel implementation of sequential tests. Comparison A (200mg/day given alone) will test null hypotheses H_{01} and H_{02}, Comparison B (8mg given alone) will test H_{03} and H_{04}. The sequence of tests in each arm will be ADAS-cog$_{11}$ first; if significant, ADCS-ADL$_{23}$ will be tested at an alpha of 0.025.

The individual tests will be implemented through contrast statements based on the repeated measures linear mixed model.

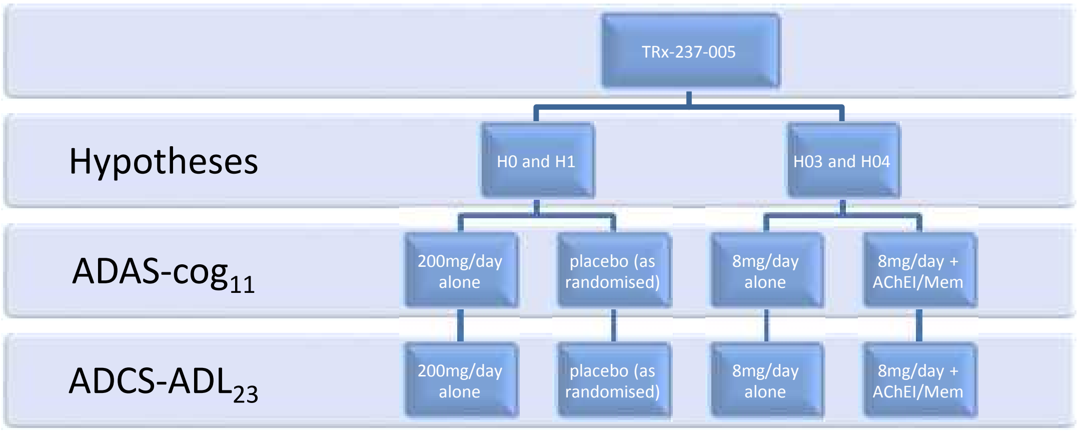

Adjustments for the randomization strata (severity, AChEI and/or memantine status at randomization, and geographic region) will be based on how the subject was randomized; a sensitivity analysis will be provided that tests actual status of covariates at baseline. The choice of Week 65 or Week 78 as the designated time point for the primary analysis dependent of the number of subjects who remain in the study at Week 78 will be made following blinded data review and documented prior to unblinding. If Week 65 is the designated time point for primary analysis, the primary model will be adjusted accordingly, i.e. time will have five levels corresponding to Weeks 13, 26, 39, 52 and 65, and the unstructured covariance matrix will have dimension 5×5 instead of dimension 6×6.

For completeness, as an exploratory analysis LMTM 200 mg/day given together with AChEI and/or memantine will be compared to placebo as randomized; the corresponding test will be implemented as a contrast statement as well.

Data listings will include all observed data for the co-primary endpoints. The listings will also display rater initials so any changes in raters can be identified. For both the ADAS-cog_{11} and the ADCS-ADL_{23}, the calculated total score adjusted for any missing items as described in Appendix F, will also be included.

As a sensitivity analysis, these analyses will be repeated including subjects who had scheduled off-treatment visits; in addition, the visit window for Week 13 will be days 1-137; likewise, non-follow-up visits that occurred after Week 78 (or Week 65 if fewer than 60% of subjects are retained at Week 78) (according to Appendix A) will be treated as Week 78 (or 65) unless there was a scheduled Week 78 (or 65) visit for this subject already. Subjects with a baseline after first dose of study drug on Day 1 will be included in this sensitivity analysis as well.

Per EMA’s suggestions, as a sensitivity analysis the above specified analysis for ADCS-ADL_{23} will be repeated for Basic ADCS-ADL_{23} as well as Instrumental ADCS-ADL_{23}; details about Basic as well as Instrumental ADCS-ADL_{23} are specified in Appendix F.

Additionally, per the EMA’s request, an intervention effect as defined below as the primary endpoint for ADAS-cog_{11} and ADCS-$ADL_{23,}$ rather than the treatment effect, will be reported. Using the same mixed-effects repeated measures (MMRM) model, an estimate of the treatment effect E at the final time point as specified as the primary analysis described above, will be obtained. For each subject who withdrew from the study the decision will be made as part of the blinded data review meeting whether the withdrawal was potentially treatment related. For potentially treatment related drop-outs, the fraction

of subjects randomized to active treatment who withdrew at a given time point is calculated. If a subject is determined to withdraw for non-treatment related reasons, no further adjustment is made as the mixed effects model correctly accounts for subjects who are missing at random.

In this intervention effect analysis, patients who withdraw for potential treatment-related reasons are assumed to retain 100% of the treatment effect they had attained up to the point of withdrawal but do not continue to benefit from treatment afterwards. This corresponds to the estimate I = (E0*w0 + E13*w13 + E26*w26 + E39*w39 + E52*w52 + E65*w65) + E78*(1-w0-w13-w26-w39-w52-w65), where E13 is the treatment effect at week 13, w13 is defined as the fraction of subjects who withdrew for potentially treatment-related reasons and have a non-missing score for either ADAS-cog_{11} or for ADCS-ADL_{23} at week 13 and do not have any on-treatment assessments for these two measures at any scheduled visit subsequent to week 13. The other wxx are defined likewise. Wxx is calculated for each treatment group separately, for active treatment groups only. w0 refers to subjects that had no measurement taken after the baseline measurement and thus E0 is zero. This analysis will be implemented as a contrast statement.

Intercurrent illnesses and concomitant medications that could confound efficacy assessments were identified during the blinded data review meeting. As further sensitivity analyses, e.g. the primary efficacy analyses described above based on the mITT population, will be repeated excluding the individual assessments that are potentially affected.

Not part of the analysis sequence , a non-inferiority test will investigate if the 8mg/day given alone is non-inferior to the 200mg/day given alone. The non-inferiority margin will be one unit, in agreement with the time to decline threshold.

9.2 Secondary Efficacy Analyses

The primary population for secondary efficacy analyses will be the MITT population for the FDA and ITT population for the EMA; for volumetric analyses, the MRI Imaging Population will replace the MITT for FDA. For each summary and value if there are multiple visits within a visit window, the rules as specified in Sections 5.4 and 9.1 will be used for that visit window summary.

The secondary efficacy analyses will be tested in fixed order for both arms; the significance level remains at 0.025.

9.2.1 Ventricular Volume

The study is also designed to evaluate the potential for disease modification using change from Baseline in ventricular (VV). The change in ventricular brain volume will be quantified using the VBSI (Fox and Freeborough, 1997).

The VV is less sensitive to confounding hydration effects and covers partly temporoparietal areas which are of particular interest in AD pathologies. Therefore, the VV is used as the primary imaging parameter. Inferential analysis will be undertaken at Week 78 (or Week 65 if fewer than 60% of subjects are retained at Week 78). The corresponding null hypotheses are

H_{05}: There is no difference between LMTM 200 mg/day given alone and placebo as randomized in reduction in brain atrophy at Week 78 (or Week 65 if fewer than 60% of subjects are retained at Week 78).

H_{06}: There is no difference between LMTM 8 mg/day given alone and LMTM 8 mg/day given together with AChEI and/or memantine at baseline in reduction in brain atrophy at Week 78 (or Week 65 if fewer than 60% of subjects are retained at Week 78).

The tests will be performed at an alpha of 0.025 as they are sequential tests in their respective arms as defined for the primary analyses.

The change in VV will be analyzed using a restricted maximum likelihood based repeated measures linear mixed effects model with an unstructured covariance matrix, with time (six levels: corresponding to Weeks 13, 26, 39, 52, 65 and 78), the treatment group by time interaction, AChEI and/or memantine status at randomization (two levels: current ongoing use or not ongoing use) by time interaction, AChEI and/or memantine status at randomization (two levels: current ongoing use or not ongoing use) by treatment group interaction, disease severity (CDR) and the corresponding Baseline value (corresponding Baseline VV is defined as the last non-missing value prior to the first dose of study drug). The primary comparison will be based on the modeled change from Baseline at Week 78 (Week 65 if fewer than 60% of subjects are retained at Week 78). As for the primary analysis, the analysis will be performed for scheduled on-treatment visits according to windows defined in Appendix A. If this is statistically significant to the 2.5% significance level as stated above, a disease modifying claim is supported by the analysis. There will be stringent quality control of longitudinal atrophy data and so correction with reference to total intracranial volume will not be used.

Additionally, we report an intervention effect as defined for the primary endpoints for the VV; as a sensitivity, the analyses will be repeated with actual status of the covariates at baseline rather than the as-stratified status.

As a sensitivity analysis, these analyses will be repeated including subjects who had scheduled off-treatment visits; additionally, the visit window for Week 13 will be days 1-137; likewise, non-follow-up visits that occurred after Week 78 (or Week 65 if fewer than 60% of subjects are retained at Week 78) (according to Appendix A) will be treated as Week 78 (or 65) unless there was a scheduled Week 78 (or 65) visit for this subject already. Subjects with a baseline after first dose of study drug on Day 1 will be included in this sensitivity analysis as well.

If there is a significant effect of treatment on VV, an exploratory analysis (not part of the secondary analysis hierarchy) will be conducted to examine the association between change in VV and change in ADAS-cog_{11} at Week 78 (Week 65 if fewer than 60% of subjects are retained at Week 78) will be assessed by dichotomizing the change values and using Pearson's chi-square test to analyze the resulting 2×2 table.

For ADAS-cog_{11}, the threshold for dichotomization will be defined as average of the estimated least squares means (MITT population-weighted LS means) for change from Baseline to Week 78 (Week 65 if fewer than 60% of subjects are retained at Week 78) in the respective treatment comparisons (8mg/day alone vs 8mg/day + AChEI/memantine and 200mg/day given alone vs placebo as randomized). Using this threshold, the population will be split into ADAS-cog_{11} “non-decliners” (i.e., those with change ≤ the threshold; later defined as responders in this document) and “decliners” (i.e., those with change > the threshold) groups. Similarly, the VV change will be split into two groups based on a threshold defined as the average of the estimated least squares means (LSM1 + LSM2)/2 for change from Baseline to Week 78 (Week 65 if fewer than 60% of subjects are retained at Week 78) in the respective treatment comparisons . Using this threshold, the population will be split into VV “decliners” (i.e., change > the threshold) and “non-decliners” (i.e., change ≤ the threshold) groups.

The number and percent of subjects in each cell of the 2×2 table will be tabulated along with the p-value from the Pearson's chi-squared test.

In order to answer the question of whether change in VV at Week 39 predicts clinical non-decliner status at Week 78 (Week 65 if fewer than 60% of subjects are retained at Week 78), the same analysis as mentioned above will be performed for these weeks. The VV threshold will be defined as the average of the least squares means (MITT population-weighted LS means) for change from Baseline to Week 39 in their respective

treatment comparisons. The ADAS-cog_{11} threshold at Week 78 (Week 65 if fewer than 60% of subjects are retained at Week 78) is defined as above.

The correlation between VV and ADAS-cog_{11}, using the original values without any dichotomization, will also be summarized using the Pearson correlation coefficient and the Spearman rank correlation coefficient. The same weeks as for the dichotomised analysis will be used.

These correlation analyses will be performed using observed on-treatment data only, and will be carried out within their respective treatment comparisons. All threshold values will be based on the least squares means from the MITT analysis.

9.2.2 Integrated Analyses

A companion Phase 3 study, TRx-237-015, comprises patients with AD of mild severity (MMSE 20 to 26 at baseline) and patients with AD of moderate severity (MMSE 14 to 19 at baseline); has the following treatment arms (LMTM 8 mg/day, LMTM 150 mg/day and 250 mg/day, all with or without AChEI/memantine); and has the last on-treatment visit at Week 65. The current Phase 3 study, TRx-237-005, comprises patients with AD of mild severity only; it has the following treatment arms (LMTM 8 mg/day, LMTM 200 mg/day both with or without AChEI/memantine); and has the last on-treatment visit at Week 78.

In view of the relatively small number of subjects receiving LMTM treatment alone and to achieve a better estimate of the treatment effect size in mild AD, data from both Phase 3 AD studies will be integrated as follows:

1. Pooled analyses will be performed on data from patients with AD of mild severity at baseline. The three main repeated measures analyses described in Sections 9.1 and 9.2.1 will be repeated, but with the following fixed-effect terms:

- pooled treatment (two levels, placebo and active)
- study (two levels, 005 and 015)
- time (five levels, corresponding to Weeks 13, 26, 39, 52, 65)
- a study-by-time interaction term
- a pooled treatment-by-time interaction term
- AChEI and/or memantine status at randomization (two levels) by time interaction term
- AChEI and/or memantine status at randomization (two levels) by pooled treatment

interaction term

- geographic region (two levels: the Americas, non-Americas)
- baseline score (ADAS-cog$_{11}$ or ADCS-ADL$_{23}$ or Ventricular Volume, as appropriate)

Treatments are pooled as follows:

- 8 mg/day will become the ‘low dose group’ for this integrated analysis
- 150 mg/day, 200 mg/day, and 250 mg/day will become the ‘high dose group’ for this integrated analysis

As for the primary analyses, the comparisons for the ‘treatment effects’ will be:

- high dose group (high dose given alone) vs placebo as randomized
- 8 mg/day dose given alone vs 8 mg/day + AChEI/memantine

These analyses will be repeated with an extra study-by-time-by-pooled treatment interaction term. If there is no evidence of a qualitative interaction (i.e., if the three-way interaction is not statistically significant, and the treatment effects at Week 65 are in the same direction and have similar magnitudes), then the overall treatment effect at Week 65 from the first model will be reported. Otherwise, the average treatment effect at Week 65 across the two studies from the second model will be reported.

2. The three main repeated analyses described in Sections 9.1 and 9.2.1 will be repeated, but on the subgroup of patients with AD of mild severity at baseline, with pooled treatment arms (as described above), and without the baseline severity term. For each outcome measure, meta analyses will be performed combining the outcome from this simplified analysis of TRx-237-015 data with the outcome of the corresponding TRx-237-005 analysis. They will be performed in two ways: first combining the Week 65 results from both studies, second combining the Week 65 results from study TRx-237-015 with the Week 78 results from study TRx-237-005.

These integrated analyses of studies TRx-237-015 and TRx-237-005 will be conducted after completion of study TRx-237-005. A corresponding alpha level will be provided if possible.

9.2.3 ADCS-CGIC

ADCS-CGIC scores at Week 78 (or Week 65 if fewer than 60% of subjects are retained at Week 78) will be analyzed using the Cochran-Mantel-Haenszel mean score test, with modified ridit scores. The analysis will be stratified by geographic region (2 levels: the Americas, Europe/Australia), disease severity (CDR). The 8 mg/day and 200 mg/day treatment groups will be subdivided into the treatment groups of interest as for the primary analysis, 200 mg/day given alone vs placebo as randomized (Comparison A) and 8 mg/day vs 8 mg/day + AChEI/memantine (Comparison B). For subjects with missing data at Week 78 (or Week 65, as described above), the last available ADCS-CGIC score will be carried forward. Subjects who do not have any on-treatment assessments will be excluded from the MITT LOCF analysis. The LOCF procedure is as follows: if a patient has one or more on-treatment assessments in the Week 78 window, use the one closest to the scheduled date. Otherwise, use the latest on-treatment score prior to this visit window. The comparisons will be assessed against a 2-sided significance level of 0.025.

In order to assess the impact of missing data in an exploratory analysis (not part of the secondary analysis hierarchy), the following two sensitivity analyses will be carried out:

- The analysis described above will be repeated using the data from Weeks 13, 26, 39, 52 and 65 (if not used for the primary analysis), with no imputation for missing data.

  This sensitivity analyses will be attempted with the same stratification variables as stated for the secondary ADCS-CGIC analysis. If it fails because of too few cases at some visit, the entire analysis (i.e. all visits) will be repeated with stratification geographic region removed. If this fails, the analysis will be deemed unreliable and discarded.

- The analysis at Week 78 (or Week 65) will be repeated with missing data replaced by the subject's worst (least favorable) value from the earlier time points (WOCF). The WOCF procedure is as follows. First, for each visit window, pick the on-treatment observation closest to the scheduled date. Then, if the Week 78 score is missing, carry forwards the worst observation from all preceding scheduled visit windows.

Additionally, an analysis of ADCS-CGIC will be carried out using a restricted maximum likelihood based repeated measures linear mixed model with an unstructured covariance matrix in which no data will be imputed; for this analysis ADCS CGIC will be treated as a continuous variable with ranges from -3 to 3:

- 3 = marked improvement
- 2 = moderate improvement
- 1 = minimal improvement
- 0 = no change
- -1 = minimal worsening
- -2 = moderate worsening
- -3= marked worsening

The corresponding analysis will be carried out in the same way as the primary analysis for ADAS-cog_{11}, with the only difference being that there is no baseline value to be included as a covariate in the model. The same analysis will be repeated using modified ridit score. This analysis will be repeated for the ITT population taking the withdrawal rates into account as described in the primary analyses for EMA (Section 9.1).

9.2.4 MMSE, MADRS, and NPI

The MMSE, MADRS, and NPI will be analyzed using a repeated measures model, similar to that described for the primary analysis on ADAS-cog_{11}. The model will include fixed effects for treatment group (two levels), time (three levels, corresponding to Weeks 26, 52, and 78), the treatment group by time interaction, AChEI and/or memantine status at randomization (two levels: current ongoing use or not ongoing use) by time interaction, AChEI and/or memantine status at randomization (two levels: current ongoing use or not ongoing use) by treatment interaction, disease severity (CDR), and geographic region (2 levels: the Americas, Europe/Australia). In addition, the baseline MMSE/MADRS/NPI will be included as a covariate, where Baseline is defined as the last non-missing value prior to the first dose of study drug. An unstructured covariance model will be used. The Kenward and Roger method of calculating the denominator degrees of freedom will be used for the tests of fixed effects.

The comparisons implemented as contrast statements will be

- 200 mg/day given alone vs placebo as randomized
- 8 mg/day given alone vs 8 mg/day given with AChEI/memantine (as determined by randomization stratification).

9.3 Exploratory Responder Analyses

Responder analyses will be conducted for ADAS-$cog_{11,}$ ADCS-ADL_{23} and for ADCS-CGIC by dichotomizing each endpoint. These analyses will be conducted for the MITT, ITT, and PP populations.

9.3.1 ADAS-cog_{11} Responder

For ADAS-cog_{11}, a responder for the two treatment comparisons (A and B) separately will be defined as a subject whose change from baseline is less than or equal to a threshold T defined as follows:

- Let LSM1 be the LS Mean of change from baseline for the corresponding control group from the primary endpoint analysis
- Let LSM2 be the LS Mean of change from baseline for the LMTM given alone group from the primary endpoint analysis
- Then T = (LSM1 + LSM2)/2

Thresholds will be chosen based on the MITT population-weighted LS means from the primary mixed-effects model.

Subjects who do not have a final ADAS-cog_{11} score will be classified as non-responders. The proportion of responders will be compared for LMTM given alone group *versus* the corresponding control using the Cochran-Mantel-Haenszel test, adjusting baseline disease severity (CDR) and geographic region (2 levels: the Americas, Europe/Australia) as the strata. Odds ratios and 95% confidence intervals will be presented. Additional responder definitions for the ADAS-cog_{11} may be specified after blinded data review.

9.3.2 ADCS-ADL_{23} Responder

For ADCS-ADL_{23} responders will be defined as for ADAS-cog_{11}. The analysis will be exactly the same as the ADAS-cog_{11} responder analysis.

9.3.3 ADCS-CGIC Responder

For ADCS-CGIC, responders will be defined in two different ways:

- Subjects without moderate or marked decline (scores of 1, 2, 3, 4 or 5)
- Subjects without any decline (scores of 1, 2, 3, or 4)

Subjects who do not have an ADCS-CGIC result will be classified as non-responders.

Each of the two ADCS-CGIC responder endpoints will be analyzed with the Cochran-Mantel-Haenszel test, split for the two treatment comparisons (A: 200 mg/day given alone vs placebo as randomized and B: 8 mg/day given alone and 8 mg/day + AChEI/memantine determined as randomized), correcting for disease severity (CDR), and

geographic region (2 levels: the Americas, Europe/Australia) as the strata. A separate test will be performed at each visit. The proportion of responders at each visit will be presented along with the odds ratio, the 95% confidence interval, and p-value for the odds ratio.

An additional repeated measures analysis of the ADCS-CGIC responder endpoints will be carried out using the data from Weeks 13, 26, 39, 52, 65 and 78. A Generalized Estimating Equation (GEE) model using the logit link function, the binomial variance function, and the unstructured working correlation model will be fit. This model will split for the two treatment comparisons (A: 200 mg/day given alone vs placebo as randomized and B: 8 mg/day given alone and 8 mg/day + AChEI/memantine determined as randomized), accounting for disease severity (CDR), and geographic region (2 levels: the Americas, Europe/Australia) as covariates. All available data will be used, with no imputation for missing data.

9.4 Sensitivity Analyses

Sensitivity analyses for ADAS-cog_{11}, ADCS-ADL_{23}, and ADCS-CGIC will be conducted to assess the impact of missing data and to assess alternative repeated measures models.

9.4.1 ADAS-cog_{11} and ADCS-ADL_{23}

The primary analysis of ADAS-cog_{11} will be repeated using the PP population as well as restricted to the group of subjects that completed the study (i.e., had a week 78 on-treatment visit).

In addition, maximum likelihood (rather than restricted maximum likelihood) based repeated measures models with polynomial time effects with time treated as a continuous variable (number of nominal weeks as well as actual study week defined by study day divided by 7) will be assessed. Tests of the significance of the cubic time effects will be carried out in each treatment group and overall. A reduced model will be fitted in which the time effects are parameterized as quadratic functions. An additional reduced model will be fitted in which the time effects are parameterized as linear functions. The estimated annualized change in mean values and standard errors from Week 0 to Week 78 and from Week 39 to Week 78 (or Week 65 if fewer than 60% of subjects are retained at Week 78) will be presented.

A multiple imputation analysis will be carried out using the MITT and ITT populations. The missing values of ADAS-cog_{11} will be imputed with multiple imputation methodology using PROC MI in SAS. A multivariate normal imputation model will be used by treatment group with a seed of 237005 and will include ADAS-cog_{11} at Baseline and at each week during the treatment period. The imputation model will have exactly the same covariates as

the primary models. A total of 50 imputed datasets will be generated for this analysis; only on-treatment observations are used. Each of the imputed datasets will be analyzed by time point using an ANCOVA model with the same covariates as the original model. The comparison will be for Comparison A 200 mg/day given alone vs placebo as randomized, and Comparison B 8mg/day given alone vs 8 mg/day given together with AChEI/memantine. The MIANALYZE procedure in SAS will be used to combine the ANCOVA results at each time point.

The following "last z-score carried forward" (LZCF) and last observation carried forward (LOCF) will analyze the ITT as well as the MITT population.

Missing values will be imputed by "last z-score carried forward" (LZCF) imputation, as follows. First, from all on-treatment observations of MITT subjects, the mean and standard deviation of change from Baseline will be computed for each combination of treatment comparison and visit, without imputation. A z-score for each subject at each visit will be computed, defined by z=(x-m)/s where x is the subject's change from Baseline, and m and s are the mean and standard deviation of change from baseline for that subject's treatment comparison at that visit, computed earlier. Missing observations are imputed by x=m+z*s, where z is the carried-forward z-score, and m and s are the mean and standard deviation for the subject's treatment comparison at the visit to be imputed, computed earlier. For subjects who do not have any post-baseline on-treatment measurements, thus whose z-score is missing, post-baseline values are imputed by x=m. Subjects without a baseline are excluded; only z-scores for on-treatment observations are carried forward. This data will be analyzed by time point using an ANCOVA model with effects as for the primary model. The comparison will be for Comparison A 200 mg/day given alone vs placebo as randomized, and Comparison B 8mg/day given alone vs 8 mg/day given together with AChEI/memantine.

Finally, missing data will be imputed using the last observation carried forward (LOCF) method followed by an ANCOVA analysis using the same model as described above for LZCF. For subjects who do not have any post-baseline on-treatment measurements, the baseline value is carried forward. Any subjects without a baseline are excluded. Imputations will be done on the total score level and not the individual item level. Only on-treatment observations are carried forward.

To quantify the effect of exposure to study drug on the treatment effect, an ANCOVA will be carried out. The dependent variable will be change from Baseline to Week 78 (or Week 65). The model will include geographic region (2 levels: the Americas, Europe/Australia), disease severity (CDR), and baseline value. The comparison will be for Comparison A 200 mg/day given alone vs placebo as randomized, and Comparison B 8mg/day given alone vs

8 mg/day given together with AChEI/memantine. The model will be fitted within each subset of following categories for the duration of exposure to study drug (based on total duration of exposure, inclusive as well as exclusive of interruptions, and alternative categories defined on the basis of total drug exposure in (kilo) grams; cf Section 10.1):

Category of Exposure (days)
1-67
68-137
138-228
229-319
320-410
411-501
>501

It is very likely that there are insufficiently many subjects in certain categories to allow the ANCOVA to converge; if this is the case the analysis will not be presented for this category. Mean values and Standard Deviations will be provided in any case.

Further sensitivity analyses are planned to determine the effect of withdrawals on estimation of intervention effect (as required by the EMA). These include the alternative assumption that the subjects withdrawing from treatment do not retain any treatment effect; the intervention effect I in this case is I=E*(1-w), where w is the fraction of subjects who withdraw for potential treatment related reasons. This is implemented as a contrast statement as well.

A further sensitivity analyses will be conducted in subjects who withdraw. The validity of the assumption of missing at random made by the MMRM will be investigated by tabulating the mean, standard deviation and number of cases of post-withdrawal treatment effects by time whenever available. This includes off-treatment follow-up data from subjects who discontinue treatment but remain on study as well as any 1 month follow up data for patients who withdrew. Additionally, the correlation between pre-withdrawal treatment effects and time of withdrawal will be examined; the hypothesis to be tested at each week (13, 26, 39, 52, and 65) is whether there is difference in assessment (ADAS-cog_{11}) change from baseline value among groups that are classified based on the withdrawal time. A linear model will be implemented to explore the strength of the relationship between time of withdrawal (quantified by the following groups: withdrawn between nominal Weeks 13 and 26, between nominal Weeks 26 and 39, between nominal Weeks 39 and 52, after Week 52) and pre-withdrawal treatment effect in separate tests. In order to be able to run these tests, all groups will be included in the model when testing for the pre-withdrawal treatment effect at Week 13. Similarly, the "between nominal Weeks

26 and 39", "between nominal Weeks 39 and 52", "between nominal Weeks 52 and 65" and "after Week 65" will be included when testing for the pre-withdrawal treatment effect at Week 26; and so on. At each Week (13, 26, 39, 52, and 65), a mixed effect model will be utilized to explore the treatment effect where the relevant withdrawn groups, as listed above, are included as class covariates. Only the measurements up to the corresponding week are included in the mixed model. For the analysis at Week 13, ANCOVA will be used instead since there are no repeated measurements up to Week 13.

The sensitivity analyses described above for ADAS-cog_{11} will also be performed for ADCS-ADL_{23}.

The following additional analyses will be performed based on the concomitant medication (specifications for AChEI and memantine can be found in Section 10.2.3):

a. In the subgroup of baseline AChEI and/or memantine non-users, Pearson's chi-squared test will be used to test if there is any evidence that the different treatment arms are more or less likely to start AChEI and/or memantine medication during the study.
b. Primary analyses for ADAS-cog_{11} and ADCS-ADL_{23} will be repeated for the subgroup of subjects who are baseline AChEI and/or memantine users or baseline non-users and did not started AChEI and/or memantine during the study.
c. Primary analyses for ADAS-cog_{11} and ADCS-ADL_{23} will be repeated for the subgroup of subjects who are AChEI and memantine treatment naïve compared to subjects who stopped AChEI and/or memantine prior to study entry.

It is very likely that there are insufficiently many subjects for the three above comparisons; if this is the case, this analysis will not be carried out, Mean values and Standard Deviations will be presented instead.

9.4.2 ADCS-CGIC

For the modified ADCS-CGIC, analyses by visit will be performed using the PP population and using LOCF for missing values. The analyses will be repeated for the MITT, ITT and PP populations using observed data, with no imputation of missing values.

The actual usage of ACHEI/memantine will be used rather than the as-randomized classification; all key analyses will be repeated using the actual status.

As a further sensitivity, individual assessemnts are removed as per decision during the blinded data review meeting; those assessments are considered to be potentially confounded due to (serious) adverse events or changes in concomitant medicine. The identified assessments are removed from all populations (ITT, MITT, PPP, and completers, see below).

The ADCS-CGIC analyses will be repeated for the subgroup of subjects for whom the order of the assessment was ‘caregiver first/subject second’ and for the subgroup for whom the order was ‘subject first/caregiver second’.

9.5 Additional Analyses

9.5.1 Analyses by Visit

For the ADAS-cog_{11} and ADCS-ADL_{23}, the primary repeated measures model for change from baseline in ADAS cog_{11} will also be used to compare the treatment groups at each scheduled visit. Least-square means for change from baseline will be presented at each scheduled visit, for each comparison group. Differences between least-square means at each scheduled visit, along with 95% confidence intervals and p-values, will be provided. For the ADCS-CGIC, the analysis of ADCS-CGIC will be repeated at each scheduled visit.

For ADAS-cog_{11} and ADCS-ADL_{23}, the treatment effect at Week 39 will be compared to the treatment effect at Week 78 in order to investigate a potential disease modifying effect. At each time point, the treatment effect is defined as the least squares mean difference between active treatment and corresponding control in each treatment arm (Comparison A 200 mg/day given alone vs placebo as randomized, and Comparison B 8mg/day given alone vs 8 mg/day given together with AChEI/memantine). Within each group, the treatment effect at Week 39 will be compared to the treatment effect at Week 78 using appropriate contrasts within the context of the primary repeated measures linear mixed model analysis. The difference in treatment effect between Week 78 and Week 39 normalized by the pooled standard errors will be compared to the quantiles of the standard Gaussian distribution. The p-value will be determined as difference from zero based on the cumulative standard Gaussian distribution. A statistically significant positive normalized difference would support the claim for a treatment effect on rate of disease progression (delay of disability). The estimated decline in each arm from Week 39 to Week 78 will be reported, as will the difference in treatment effects between Week 39 and Week 78.

9.5.2 Time to Event Analyses

The distributions of time to decline in ADAS-cog_{11} total score will be summarized using the Kaplan-Meier method, with onset of decline defined as the first of two consecutive measurements that are worse than the baseline score by at least one unit. Time to decline will be calculated as date of onset of decline – date of randomization + 1.

The time to decline analysis is based on visit window. The one measurement per visit window is selected including off-treatment measurements but excluding unscheduled measurements. Subjects without any decline will be censored at the date of last ADAS-cog_{11} assessment, but if a subject missed the scheduled visit, the subject is censored at the target day of the scheduled visit. The analysis will be run for both MITT and for ITT populations.

Any missing total score for a scheduled visit will be treated as a worsening from baseline by at least one unit, for that visit. This applies whether the missing total score is due to a missed visit, or early termination from the study, or a missing total score at a visit that was attended. Because of this convention for missing values, it is possible that the first of two consecutive measurements that are worse than the baseline score by at least one unit, occurs at a missed visit. In that case, since there is no date associated with the missed visit, the time to decline will be calculated based on the scheduled date of the missing visit.

The Cox proportional hazards regression model with effects for each treatment comparison and the randomization stratification variables disease severity (CDR) and geographic region (2 levels: the Americas, Europe/Australia) will be used to compare

- Comparison A: 200 mg/day given alone vs placebo as randomized, and
- Comparison B: 8mg/day given alone vs 8 mg/day given together with AChEI/memantine

by way of Hazard Ratio, 95% confidence interval and p-value (for alpha 0.025). Subjects without any decline will be censored at the date of last assessment. A graph of the Kaplan-Meier estimates will be provided.

This analysis will likewise be repeated for each subgroup of “responders” and “non-responders” as defined above based on the primary ADAS-cog_{11} analysis.

However, to explore a potentially confounding effect of delayed treatment onset, a landmark analysis may be conducted as described above with Week 26 as baseline.

9.5.3 Withdrawal and Rebound Effect

To analyze withdrawal and rebound effect, for each subject, the ADAS-cog_{11}, ADCS-CGIC and ADCS-ADL_{23} change from the final on-treatment visit at Week 78 (or Week 65 if fewer than 60% remain in the study at Week 78) to the 4-week post-treatment visit will be computed for those subjects for whom 4-week post-treatment visit data exists. For the 4-week post-treatment visit, the off-treatment measurement that is the closest to the 28 days after treatment stop date will be selected. Summary statistics will be provided within each comparison group and the ANOVA will be used to assess whether the mean change from the end of treatment to the 4-week post-treatment visit is equal to zero for each comparison group. For ADCS-CGIC, the Wilcoxon signed-rank test will be used.

To investigate the potential bias of this analysis towards subjects who complete the study, the analysis is repeated for all subjects in whom a post final treatment visit exists, irrespective of when it occurs. The change in ADAS-cog_{11} and ADCS-ADL_{23} will be normalized to a 4-week effect assuming a linear change after final on-treatment visit. Only on-treatment assessment at or after the Week 13 visit window will be used. For ADCS-CGIC, the score of the post-treatment visit will be used irrespective of when it occurs.

In these analyses, if two visits are equally far away from the target 4-week post-treatment visit, the visit that occurred later will be selected for the analyses.

9.6 Exploratory Analyses

9.6.1 FDG Temporoparietal Region Uptake and Relationship to MRI

PET scans are optional and are performed only for subjects by or for whom legally acceptable consent is provided at selected sites. The study was not designed specifically to detect significant differences between treatment groups in the FDG temporal lobe uptake. The change in FDG temporal lobe uptake on PET will be analyzed using PET Imaging Population subjects.

The change from Baseline in mean temporoparietal Region FDG uptake (average of right/left parietal cortices and right/left lateral temporal cortices, normalized to the cerebellum and pons, separately – in effect there will 2 sets of normalized values) will be analyzed using a restricted maximum likelihood based repeated measures linear mixed model. The model will include fixed effects for treatment group, time (two levels, corresponding to Week 39 and Week 78), the treatment group by time interaction, AChEI and/or memantine status at randomization (two levels: current ongoing use or not ongoing use) by time interaction, AChEI and/or memantine status at randomization (two levels:

current ongoing use or not ongoing use) by time interaction, and disease severity (CDR). In addition, the baseline value will be included as a covariate (Baseline is defined as the first non-missing value prior to the first dose of study drug). An unstructured (2×2) covariance model will be used. The Kenward and Roger method of calculating the denominator degrees of freedom will be used for the tests of fixed effects. The aim is to compare the two treatment arms as for the primary analyses:

- Comparison A 200 mg/day given alone vs placebo as randomized, and
- Comparison B 8mg/day given alone vs 8 mg/day given together with AChEI/memantine

In order to evaluate the relationship between change in FDG-PET and cognitive changes, Pearson's correlation coefficient and Spearman's rank correlation coefficient for ADAS-cog_{11} change at Week 78 and FDG uptake change at Weeks 78 will be tabulated. Similarly, Pearson's correlation coefficient and Spearman's rank correlation coefficient for ADAS-cog_{11} change at Week 78 and FDG uptake change at Weeks 39 will be tabulated. These correlation coefficients will be calculated within each treatment group (Comparison A: 200 mg/day given alone vs placebo as randomized, and Comparison B: 8mg/day given alone vs 8 mg/day given together with AChEI/memantine).

The relationship between changes in whole brain, ventricular, hippocampal, and temporoparietal volumes, and temporoparietal Region FDG uptake will be explored using the Pearson correlation and non-parametric Spearman's rank correlation analyses.

The above analyses will be repeated in its entirety for the following sub-regions:

- Temporal left
- Temporal right
- Parietal left
- Parietal right

9.6.2 Additional MRI Analyses

Change in whole brain volume as well as hippocampal and temporoparietal volume will be analyzed using a restricted maximum likelihood based repeated measures linear mixed effects model with an unstructured covariance matrix. The model will be exactly the same as for change in ventricular volume with the same comparisons. The hippocampal and temporoparietal volume change will be quantified using Tensor-Based Morphometry (Calmon *et al.*, 2000).

9.6.3 Voxel-Based Morphometry

In order to investigate the effect of treatment regional grey matter volumes without an a priori assumption of the location of potential differences, intrinsic in the ROI approach described previously. Statistical parametric mapping comparison using a longitudinal VBM approach will be implemented. Drug effects will be compared at Week 26, 39, 52, 65, 78 and follow-up. The MRI images will be segmented and spatially normalized using DARTEL. The conventional smoothing step will be applied to the registered data (8mm smooth). Using the SPM12 GUI, will compare the change in GM (gray matter) volumes between the treated and control arms at every time point. A Family-Wise Error (FWE) correction for multiple comparison will be implemented and will consider any voxel to be significant if the corrected FWE is smaller than 0.05. A small volume correction (SVC) will be applied, which is a technique that limits the extent of the multiple comparison correction by limiting the size of volume compared. The SVC will utilize: 1) parietal temporal lobes volume; 2) parietal lobes; and 3) temporal lobes separately.

This analysis will be provided by an expert imaging center. No analyses are planned in this SAP.

9.6.4 Statistical Parametric Mapping for PET

In order to investigate the effect of treatment on FDG uptake without an a priori assumption of the location of potential differences, intrinsic in the ROI approach described previously. A statistical parametric mapping comparison using SPM12 software will be used. Drug effects will be compared first at Week 39 and second at Week 78. The FDG PET images will first be scaled so that each voxel within the image represents the SUVr using a cerebellum reference region. The images will then be registered and spatially normalized to the FDG template supplied with SPM12. The conventional smoothing step will be applied to the registered data (8 mm smooth). Using the SPM12 GUI we will compare the change in FDG uptake between the treated and control arms at both first and second follow-up. A Family Wise Error (FWE) correction for multiple comparison will implemented and will consider any voxel or cluster of voxels to be significant if the corrected FWE is smaller than 0.05. A small volume correction (SVC) will be applied, which is a technique that limits the extent of the multiple comparison correction by limiting the size of volume compared. A SVC will utilize: 1) parietal temporal lobes volume; 2) parietal lobes; and 3) temporal lobes separately.

This analysis will be provided by an expert imaging center. No analyses are planned in this SAP.

9.6.5 Pharmacoeconomics

In order to investigate the pharmacoeconomic impact of LMTM (at the winning dose), a cost consequence analysis (CCA) will be performed. With a cost consequence analysis, a broad set of relevant outcomes (here the ADAS-cog$_{11}$, ADCS-ADL$_{23}$ and MMSE) will be tabulated and discussed in relation to costs. Costs and outcomes are presented separately.

This analysis has a risk of yielding non-significant effects regarding costs as clinical trials evaluating efficacy are typically underpowered with respect to pharmacoeconomic results and long-term effects cannot be assessed due to time constraints in the clinical trials. Therefore, a modelling approach will complement the analysis.

Resource Use

The Resource Utilization in Dementia instrument (RUD) is a comprehensive and validated instrument collecting data on resource use in trials, with the aim to calculate costs from a societal viewpoint. The RUD Lite questionnaire, a short version of RUD mainly discarding the resource use by the caregivers is employed here. All subjects, for whom RUD Lite data was collected at one time point at least, will be included in the summaries.

Together with costs of institutional care, the value of informal care is the heaviest cost driver in dementia care. Three components of caregiver time will be included in the analysis:

- time spent on toilet visits, eating, dressing, grooming, walking and bathing;
- time spent on shopping, food preparation, housekeeping, laundry, transportation, taking medication and managing financial matters; and
- time spent on supervising the subject.

Each component of caregiver time will be calculated as the hours of care on a typical day multiplied by days spent on providing these services. The caregiver time will be summarized for each treatment group by visit and by AChEI and/or memantine status at randomization, severity, and geographic region.

Caregiver work status, whether the subject was admitted to a hospital, whether the subject received services in a hospital emergency room, whether the subject visited any health care professional, and whether the subject received any nursing services will be summarized likewise.

Caregiver relationship with subject and the subject’s living accommodations will be summarized descriptively.

Unit Costs
Costs will be calculated as the multiplication of the amount of units of resource use and the unit cost for this particular resource. Unit costs for each unit will be collected for each country (if unit cost data are not available for each trial country, then unit cost data for the dominant country only will be used). An average wage will be used as a proxy for the opportunity cost of informal care. Caregiver time for retired carers will be given a value of 35% of caregivers of working age.

These analyses will be repeated at the non-winning dose.

Statistical Analysis
In order to statistically support the descriptive analysis, regression models will be employed. Generalized linear models assuming gamma distributed dependent variables will be used accounting for the skewness of resource use and cost data. Bootstrapping methods will complement the analysis by providing estimates for the confidence intervals in the univariate analyses.

If there is a substantial amount of missing data on resource use (> 20%), multiple imputation approaches will be tested.

Economic Modelling
As the long-term cost-effectiveness of LMTM beyond the trial periods is of great interest, economic evaluations based on various modelling techniques will be used. These models most often have a cost utility analysis (CUA) design in which the relation between costs and outcomes is expressed as the incremental cost effectiveness ratio (ICER). The quality adjusted life years (QALY) concept is the most frequently used outcome. However, EQ5D data are not collected in this study, only in the open-label extension (Study TRx-237-020). Therefore, external data calibrating EQ5D for ADAS-cog_{11}, ADCS-ADL_{23} and MMSE will be used to estimate the implied impact of treatment on QALY. External data for disease progression and mortality as well as data on resource use and costs beyond the trial period will also be used as inputs. From the trial, data on efficacy are used as an empirical core for the model. Within trial data on resource use and costs as well as the efficacy data and derived estimated QALY data will be used to calibrate the external sources in the model. Often-used modelling techniques such as Markov models and Discrete Event Simulations (DES) will be tested.

9.6.6 CSF Biomarkers

CSF samples for biomarker analysis are collected in a subset of subjects who are mentally capable of providing their own separate informed consent and specifically agree to have lumbar puncture performed. Levels of $A\beta_{1-42}$, phospho-tau:$A\beta_{1-42}$, and phospho-tau are

measured in the CSF samples using the INNO-BIA AlzBio3 Luminex-based assay. For MITT subjects who provide a CSF sample at Screening/Baseline and at least one post-baseline CSF sample, levels of CSF biomarkers total tau, phospho-tau, and $A\beta_{1-42}$, will be summarized descriptively by treatment group at Baseline and Week 78. For all CSF biomarker summaries, Baseline is defined as the last non-missing value prior to the first dose of study drug. Change from Baseline to Week 78 will also be summarized. In addition to these three biomarkers, the ratios total tau: $A\beta_{1-42}$, phospho-tau:$A\beta_{1-42}$, and phospho-tau:total tau will be derived and summarized.

Change from Baseline at Week 78 will be analyzed for each biomarker and ratio using analysis of covariance (ANCOVA). Comparison groups and covariates will be as for the key primary and secondary analyses. Results will be presented along with 95% confidence intervals and p-values. Results from early termination visits will be included in these Week 78 summaries and analyses.

The correlation between change from Baseline to Week 78 in each CSF biomarker (including the ratios) and change from Baseline to Week 78 in ADAS-cog_{11} and ADCS-CGIC will be examined, as described above for ventricular volume.

If there are less than 5 subjects in a comparison group, then only descriptive statistics will be provided.

9.7 Pharmacokinetic Analyses

A listing of concentration data and time of sample collection relative to dosing will be generated. Population pharmacokinetic analyses will be described in a separate plan.

10. SAFETY ANALYSES

All safety analyses will be based on the Safety population. Summaries will be presented by treatment allocation.

In addition, key summaries will be further split by AChEI and/or memantine usage; this includes but is not restricted to summaries of Adverse Events and select laboratory parameters. Furthermore, AChEI and/or memantine usage will be cross-tabulated with usage of antipsychotic as well as anti-depressant drugs. AChEI and/or memantine usage will be determined based on the concomitant medication information rather tham the randomization stratification classification.

10.1 Extent of Exposure

All subjects are to receive 2 tablets twice daily. Interruption(s) of study drug administration and resumption of dosing at the same or at a reduced dose is allowed. If a dose reduction is warranted, there are two ways that a step-down in dose will be implemented:

- By dispensing new study drug packages to the subject in a manner designed to maintain blinding (the subject will continue to take 2 tablets daily, but will take tablets from the newly dispensed packages) or
- By instructing the subject to omit morning dose in their original packaging and take only 1 tablet daily.

There are three sources of data for determining exposure:

- The Drug Accountability Log CRF provides a sequential record of dispensing and return of tablets (including kit ID dispensed and a comment field).
- The Dose Adjustment / Interruption Log CRF provides a record of the start and stop dates of each treatment interruption and dose changes (including a comment field).
- The Study Exit Status CRF provides a record of the stop date for the last dose of study drug; the Day 1 Pre-Dose – Date and Time on “In-Clinic First Dose of Study Drug” CRF provides information about the first dose of study drug.

For each subject, the total duration of exposure, exposure accounting for interruptions, mean and modal daily doses (including a dose of 0 for days not dosed and the reduced dose when applicable), cumulative dose and dosing compliance, will be summarized descriptively by treatment group. Notations on the Drug Accountability Log CRF about missed doses are not accounted for programmatically; these will be included in the listing.

Total duration of exposure is defined as the difference between the last dose date and the first dose date, plus 1 day. The total exposure duration defined above includes any periods where the dose is interrupted. A second calculation of exposure duration that excludes dose interruptions will also be performed.

Mean daily dose is calculated as follows:

$$\frac{\sum_{i=1}^{n} \text{Dose}_i(\text{mg/day}) \times \text{Duration(days) on Dose}_i}{\text{Total Duration (days)}}$$

The cumulative dose (i.e., the numerator in the above formula) will also be summarized.

Compliance rate over the entire treatment period is calculated using the following formula:

$$\frac{100 \times (\text{Total Tablets Dispensed} - \text{Total Tablets Returned})}{[\text{Total Duration} - \text{Duration of Dose Interruption}] \times 2 - \text{Duration of Tablet Reduction}}$$

where duration of tablet reduction refers to the number of days when subjects only takes 1 tablet as documented on the Dose Adjustment / Interruption Log CRF.

In addition, tabular summaries of the proportions of subjects with dose interruptions and dose reductions will be prepared for each treatment group.

The frequency and percentage of subjects in the following duration of exposure categories (based on total duration of exposure, inclusive of interruptions) will be presented:

**Category of Exposure (days)**
1
2-28
29-67
68-137
138-228
229-319
320-410
411-501
>501

The cumulative number and percentage of subjects across the categories will also be summarized, as well as total subject years of exposure, defined as the sum of exposure in years, will also be presented. Study drug exposure will be summarized for all the analysis populations.

All study drug administration and compliance data will be presented in listings, including chronological dates of administration, number of tablets dispensed and returned with corresponding package ID and type, treatment duration, mean and modal daily dose, compliance, and any associated comments; cases will be highlighted where the incorrect kit/treatment was dispensed. An additional listing will include study drug adjustment and interruption information, including whether there were any dose reductions or interruptions, the last dose taken prior to the dose change, the start and end date of the change, the number of tablets per day, and any associated comments.

10.2 Adverse Events

Adverse events will be coded to System Organ Class (SOC) and preferred term using MedDRA (version 16.0). The coding process is described in the Data Management Plan.

The tabular summaries will be provided for AEs, with the number and percentage of subjects reporting each type of event presented by treatment group. If a subject reports the same preferred term more than once, it is counted only once within that category. Further, for a given tabulation, the preferred term will only be counted once at its worst severity and strongest relationship to treatment.

Pre-treatment adverse events are defined as adverse events that have an onset prior to the time of the first dose and after the informed consent date. A summary of pre-treatment adverse events by MedDRA system organ class and preferred term will be provided and will be flagged in the overall listing of the adverse events.

Adverse events will be regarded as treatment-emergent (TEAE) if they start on or after the time of first dose of study drug administration or if they were present prior to the first dose of study drug administration and increased in severity or relationship to study drug while on study drug.

The following summaries for TEAEs will be provided:

- An overall summary table of TEAEs summarizing the number and percent of subjects, and the number of unique TEAEs, in the following categories: any TEAE, severe TEAE, TEAE related to study drug, serious TEAE, TEAE with outcome of death, AESIs, TEAE leading to dose reduction, TEAE leading to dose interruption, TEAE leading to withdrawal of study drug, and TEAE leading to dose reduction, dose interruption, or withdrawal of study drug.
- Subject incidence of TEAEs and total number of unique TEAEs by MedDRA system organ class and preferred term.
- Subject incidence and total number of unique Day 1 TEAEs, defined as all TEAEs occurring on the same calendar day as dosing (Day 1) with an onset on or after the time of the first dose of study drug.
- Subject incidence of TEAEs by MedDRA system organ class, preferred term, and worst severity.

- Subject incidence of TEAEs by MedDRA system organ class, preferred term, and strongest relationship[1] to study drug (Related/Not Related). Events reported as "Possibly Related," or "Related" will be included in the Related category. Events reported as "Unlikely Related" or "Not Related" will be included in the Not Related category. At each level of subject summarization, a subject is classified according to the strongest relationship, as determined by the investigator, if the subject reported one or more events. AEs with a missing relationship will be considered related for this summary.
- Subject incidence of serious TEAEs (SAEs) and total number of unique serious TEAEs by MedDRA system organ class and preferred term (and subsets for fatal and nonfatal SAEs).
- Subject incidence of severe TEAEs and total number of unique severe TEAEs by MedDRA system organ class and preferred term. AEs with missing severity will be considered severe for inclusion in this summary. These will all be tabulated separately for those that are considered to be Related as determined by the Investigator (as defined above).
- Subject incidence of TEAEs leading to change in dose and total number of unique TEAEs leading to change in dose (dose reduction, dose interruption, and study drug withdrawal, separately and combined) by MedDRA system organ class and preferred term.
- Subject incidence and total number of unique protocol-specified AESIs (as defined below in Section 10.2.1), by MedDRA system organ class and preferred term. AESIs are indicated on the Adverse Events CRF.

Post-treatment AEs are defined **as the subset of TEAEs** that have an onset more than 14 days after the last dose of study drug or that worsen in intensity or treatment attribution. A summary of post-treatment adverse events by MedDRA system organ class and preferred term will be provided along with a flag in the overall listing.

Summaries for subsets of TEAEs of interest (TauRx AE Groupings), planned subgroup analyses (e.g., by demographics), and time to first occurrence analyses are described in the sections following.

---

[1] Relationship is determined by the Investigator

All AEs will be presented in a data listing. In addition, listings will also be provided for SAEs (including subsets for fatal and non-fatal SAEs), AEs leading to dose reduction and/or interruption, AEs leading to discontinuation of study drug, and AESIs. ARIAs will be flagged on the AESI listing and categorized as vasogenic edema (ARIA-E), hemorrhagic ARIA (ARIA-H) or both.

10.2.1 Adverse Events of Special Interest

Several adverse events are of special interest because additional steps are to be taken by the investigator to assess and manage them. AESIs include:

- Methemoglobin values > 3.5% (confirmed on repeat result; see Protocol Section 8.4), signs or symptoms consistent with methemoglobinemia or hemolytic anemia, or observation of Heinz bodies. For purposes of this study, only post-randomization methemoglobin values meeting these criteria would be considered an AESI and whether or not this is considered an SAE depends on medical and scientific judgment.
- A case meeting any one of the four criteria for serotonin syndrome (outlined in Protocol Section 24.2) is a medically significant event and thus will be reported and handled as an SAE (see Protocol Section 8.1.6).
- Any possible case of ARIA (see Protocol Section 6.3.3.3 and Section 9.1.5.2) is a medically significant event and thus will be reported to the Sponsor and the procedure will be as for an SAE (see Protocol Section 8.1.6); asymptomatic subjects with ≤ 4 new microhemorrhages need not be handled as an SAE. TauRx Drug Safety is to confirm each ARIA case.

AESIs based on the above criteria will be confirmed by the Sponsor.

10.2.2 Selected Subsets of Adverse Events (Referred to as TauRx AE Groupings)

The groupings, listed below will be used for the present study. Summaries of adverse events by MedDRA preferred term will be provided for each grouping:

**AE Grouping:<br>MedDRA Preferred Terms**	**AE Sub-Grouping:<br>MedDRA Preferred Terms**

<table>
<tr><th>AE Grouping:
MedDRA Preferred Terms</th><th>AE Sub-Grouping:
MedDRA Preferred Terms</th></tr>
<tr><td rowspan="3"><u>Targeted Gastrointestinal Events:</u>
abdominal discomfort
abdominal distension
abdominal pain
abdominal pain lower
abdominal pain upper
change of bowel habit
colitis
colitis microscopic
defaecation urgency
diarrhoea
duodenal ulcer haemorrhage
duodenitis
dyspepsia
epigastric discomfort
faecal incontinence
flatulence
frequent bowel movements
gastric disorder
gastric ulcer
gastritis
gastrointestinal haemorrhage
gastrointestinal pain
irritable bowel syndrome
nausea
occult blood positive
oesophageal irritation
oesophagitis
peptic ulcer
procedural vomiting
rectal discharge
vomiting</td><td><u>Diarrhea Sub-Grouping</u>:
change of bowel habit
defaecation urgency
diarrhoea
faecal incontinence
frequent bowel movements
irritable bowel syndrome</td></tr>
<tr><td><u>Gastrointestinal Irritation Sub-Grouping</u>:
colitis
colitis microscopic
duodenal ulcer haemorrhage
duodenitis
gastric disorder
gastric ulcer
gastritis
gastrointestinal haemorrhage
oesophageal irritation
oesophagitis
peptic ulcer</td></tr>
<tr><td><u>Nausea/Vomiting Sub-Grouping</u>:
dyspepsia
epigastric discomfort
nausea
procedural vomiting</td></tr>
</table>

**AE Grouping: MedDRA Preferred Terms**	**AE Sub-Grouping: MedDRA Preferred Terms**
**Renal and Urinary Disorders (Including Infections):**<br>cystitis<br>cystitis interstitial<br>dysuria<br>escherichia urinary tract infection<br>genital burning sensation<br>hypertonic bladder<br>incontinence<br>micturition urgency<br>pollakiuria<br>polyuria<br>strangury<br>suprapubic pain<br>urethral pain<br>urge incontinence<br>urinary incontinence<br>urinary retention<br>urinary tract infection<br>urinary tract infection bacterial<br>urine odour abnormal	**Urinary Tract Infection Sub-Grouping:**<br>cystitis<br>escherichia urinary tract infection<br>urinary tract infection<br>urinary tract infection bacterial<br><br>**Urinary Frequency/Urgency Sub-Grouping**<br>incontinence<br>micturition urgency<br>pollakiuria<br>polyuria<br>urge incontinence<br>urinary incontinence
**Anemia**:<br>anaemia<br>anaemia macrocytic<br>blood folate decreased<br>folate deficiency<br>haematocrit decreased<br>haemoglobin decreased<br>Heinz bodies<br>iron deficiency anaemia<br>methaemoglobinemia<br>normochromic normocytic anaemia<br>oxygen saturation decreased<br>red blood cell count decreased<br>reticulocyte count decreased<br>vitamin B12 decreased<br>vitamin B12 deficiency	None proposed.

**AE Grouping:**<br>**MedDRA Preferred Terms**		**AE Sub-Grouping:**<br>**MedDRA Preferred Terms**
**Falls and Related Terms:**<br>ankle fracture<br>cervical vertebral fracture<br>clavicle fracture<br>concussion<br>coordination abnormal<br>craniocerebral injury<br>dizziness<br>facial bones fracture<br>fall<br>femoral neck fracture<br>fibula fracture<br>foot fracture<br>gait disturbance<br>hand fracture<br>head injury<br>hip fracture<br>humerus fracture<br>joint dislocation<br>joint injury<br>laceration<br>limb injury	pelvic fracture<br>periorbital haemorrhage<br>pubis fracture<br>radius fracture<br>rib fracture<br>soft tissue injury<br>spinal compression fracture<br>spinal fracture<br>stress fracture<br>subdural haematoma<br>syncope<br>tibia fracture<br>traumatic haemotoma<br>ulna fracture<br>upper limb fracture<br>wound<br>wrist fracture	**Falls Sub-Grouping**<br>fall<br>syncope<br>dizziness

AE Grouping:<br>MedDRA Preferred Terms	AE Sub-Grouping:<br>MedDRA Preferred Terms
**Hypersensitivity**:<br>dermatitis<br>dermatitis allergic<br>drug eruption<br>drug hypersensitivity<br>eosinophil count increased<br>erythema<br>pruritus<br>pruritus generalized<br>rash<br>rash maculo-papular<br>rash pruritic<br>skin exfoliation<br>skin lesion<br>swelling face	**Rash Sub-Grouping**:<br>dermatitis<br>dermatitis allergic<br>drug eruption<br>pruritus<br>pruritus generalized<br>rash<br>rash maculo-papular rash pruritic<br>skin exfoliation<br>skin lesion
**Renal Function Impairment**:<br>blood urea increased<br>creatinine renal clearance decreased<br>hypercreatininaemia<br>blood creatinine increased<br>renal failure<br>renal failure acute<br>renal failure chronic<br>renal impairment	None proposed.
**Hepatic Function Impairment**:<br>alanine aminotransferase increased<br>aspartate aminotransferase increased<br>bilirubinuria<br>blood alkaline phosphatase increased<br>blood bilirubin increased<br>blood bilirubin unconjugated increased<br>gamma-glutamyltransferase<br>gamma-glutamyltransferase increased<br>hepatic enzyme increased<br>hepatitis<br>liver function test abnormal<br>transaminases increased	None proposed.

10.2.3 Subgroup Analyses

The summaries of TauRx AE groupings described in Section 10.2.2, as well as the most common TEAEs (occurring in more than 20% in any treatment group), will be repeated for the following subgroups of subjects:

- Age group: < 75 years and ≥ 75 years
- Gender: male/female
- Race: white/non-white
- Use of AChEI/memantine at Baseline: AChEI and/or memantine, both AChEI and memantine, AChEI but not memantine, memantine but not AChEI. The subgroups based on use of AChEI/memantine at Baseline will be derived from the concomitant medications dataset. AChEI medication is defined as one whose WHOODD preferred name is one of the following: DONEPEZIL, DONEPEZIL HYDROCHLORIDE, GALANTAMINE, GALANTAMINE HYDROBROMIDE, RIVASTIGMINE, RIVASTIGMINE HYDROGEN TARTRATE. Memantine is defined as medication whose preferred name is MEMANTINE or MEMANTINE HYDROCHLORIDE
- Use of concomitant medications with serotonergic potential (listed in the Investigator's Brochure, Attachment 4 or identified in literature searches performed subsequent to the IB approval date) at any time during the study: yes/no
- Renal function (based on eGFR, mL/min/1.73 m^2): normal (≥90), mild (60-89), moderate (30-59), severe (15-29)

Other subgroup analyses of interest may be identified during data review (e.g., based on concomitant medications or medical conditions).

Relative risks with associated 95% confidence intervals will be reported for TEAEs where the incidence in both the placebo and associated treatment group is at least 5%. The relative risks compared to the placebo group will be provided for each subgroup. Breslow-Day test for homogeneity of the odds ratios across subgroups will be presented.

10.2.4 Time to Event Analyses of Adverse Events

Time to event analyses will be conducted using a Kaplan-Meier approach for each of the TauRx AE Grouping subsets defined in Section 10.2.2. For each subject, time to first event (any first event in each group) will be calculated as date of first onset – date of first treatment + 1, where date of first onset refers to the earliest onset of any event within the group. Subjects without any events in a given subset will be censored at the date of the end

of study participation for the particular subject; this is defined as the date when the subject was last known to be event free. Descriptive statistics will be summarized in a tabular fashion.

The Cox proportional hazards regression model with effects for the respective treatment groups as defined for the primary analysis and the randomization stratification variables of disease severity and geographic region (2 levels: the Americas and Europe/Australia) will be used to compare the treatment groups to their respective control groups using Hazard Ratio, 95% confidence interval and p-value. Tabular summaries and a graph of the Kaplan-Meier estimates will be provided.

10.3 Clinical Laboratory Evaluation

Central laboratory data are transferred electronically by Covance. The Data Transfer Specification document provides a detailed description of the content and format of the laboratory datasets. Both conventional and SI units are provided and will be summarized in separate tables and listings. The eGFR values will be calculated using the formula provided in Section 5.2 and will be included in the tables and listings for clinical chemistry parameters. Results from local laboratories are entered into the CRF by the site; these will not be included in summary tabulations but will be included in the data listings together with the corresponding normal ranges. Values for local laboratory parameters will be converted to the corresponding alternative units, either conventional or SI, depending on how reported.

For the summary tabulations of laboratory results, Baseline is defined as the last non-missing value prior to first dose of study drug. Laboratory tests obtained on the date of the first dose will be assigned to pre-treatment; the relative times of blood sampling and dosing will be checked programmatically to confirm this assumption. For each analyte, Baseline values will be restricted to those subjects in the safety population for whom there is at least one post-Baseline value (either overall or for the corresponding target visit).

Visit windows are used when results are presented by target visit (see Appendix A). For each analyte, if a subject has multiple values within a visit window, the “worst” value as defined in Appendix E will be used for that visit window summary.

Continuous laboratory parameters will be summarized using descriptive statistics at baseline and at each post-baseline target visit; the last available on-treatment value will also be summarized.

For each visit, changes from baseline will also be summarized by n, Mean, Median, SD and (Min, Max) for continuous laboratory parameters. In addition, continuous laboratory

parameters will be summarized by shift table according to changes from Baseline relative to the reference range (low, normal, high). Shift tables for folate and vitamin B_{12} will present two categories based on the level cut-off value indicating deficiency. For folate these categories are < 4 ng/mL (<10 nmol/L) and ≥ 4 ng/mL (≥10 nmol/L). For vitamin B_{12}, these categories are < 150 pmol/L (203 pg/mL) and ≥ 150 pmol/L (203 pg/mL).

Categorical laboratory parameters will be summarized by treatment group for each target visit using counts and percent of subjects in each category. A shift table will also be presented for categorical parameters (urinalysis shift tables will only include pH and specific gravity). Missing categories will be included in the shift tables.

In addition to the above summaries, two further analyses will be presented:

1. A graphical Box-Whisker plot with x-axis being categories of Urinalysis Protein (Negative, +1, +2, +3); and Y-axis being microalbumin/nephelometry.
2. A listing of T3/T4 if TSH is elevated (above upper limit of normal). This listing will be done only for the subjects with elevated TSH at one or more visits and will present data for all visits. The listing will at a minimum include columns indicating Visit, TSH value, T3, T4; and thyroid hormone uptake (Thyroid Uptake Assay comprising TBG (Thyroxine Binding Globulin) and thyroid hormone uptake) if available.

Box and whisker plots will be presented for selected parameters including hemoglobin, reticulocytes, neutrophils, and liver function tests. Other parameters may be identified during data review.

Thresholds for potentially clinically significant (PCS) laboratory abnormalities are defined in Appendix B for selected parameters. When there are thresholds provided for low and high values, they will be handled separately. Summaries will include the number and percent of subjects with treatment-emergent PCS values, restricted to those subjects in whom the values represent a post-Baseline worsening (PCST); the number meeting criteria at Baseline will also be summarized. A PCST is defined as any PCS event that happened at any post-Baseline visit and has a value more out of range than the value for a particular test at Baseline (see Appendix E for directionality of worsening).

Listings of laboratory parameter results will be presented. Listings will include flags for those values outside the reference range as well as flags for those that meet criteria for being PCS (whether or not a treatment-emergent worsening); additionally, results evaluated by the investigator as abnormal/clinically significant will be flagged in the listing.

Separate listings for each hematology, chemistry, and urinalysis parameter will include only subjects with treatment-emergent PCS values and, for these subjects, all laboratory results for the parameter meeting PCS criteria and related parameters (e.g., ALT, AST, bilirubin, neutrophils, WBC, etc.) will be provided.

10.4 Additional Anemia Analyses

An additional analysis of reticulocyte and hemoglobin changes will be provided to assess the effects of supplementation with folate and/or vitamin B12. Change from baseline will be summarized descriptively and separately for those subjects who are supplemented and those subjects who are not supplemented at baseline, by visit. Whether or not a subject is supplemented will be determined from the concomitant medications dataset using the ATC level 3 code (B03B) of “VITAMIN B12 AND FOLIC ACID”.

Another analysis will be performed for subjects who are not supplemented at Baseline but who subsequently start supplementation post-baseline, to assess changes in reticulocytes and hemoglobin in response to supplementation. The last value recorded up to and including the start date of supplementation will be used as the pre-supplementation value. The first hemoglobin value that is recorded at least 4 weeks after the start of supplementation will be used as the post-supplementation value. The pre-supplementation and post-supplementation values will be summarized along with the changes. The analysis will be restricted to those subjects who have no change in study drug dosing during this period.

For each of these analyses, if supplementation starts on the same day as a hemoglobin assessment, the subject will be considered as not supplemented for that assessment.

10.5 Pulse Co-oximetry

For the summary tabulations of methemoglobin and oxygen saturation results on Day 1, Baseline is the pre-dose value. Baseline will not be imputed.

Pre-dose, post-dose (target 2.5 hours post-dose), and change in methemoglobin and oxygen saturation will be summarized using descriptive statistics.

Measurements subsequent to Day 1 will be summarized descriptively by target visit; Baseline will be the last non-missing value prior to the first dose of study drug). Visit windows are used (see Appendix A) and if a subject has multiple values within a visit window, the highest methemoglobin and lowest oxygen saturation values will be used for that visit window summary.

In addition, for methemoglobin, the number and percent of subjects with values that are >2% and >3.5% will also be tabulated overall and by target visit. If repeated methemoglobin values are available at the same nominal time point, the average of the available replicates will be used for analysis. A box and whisker plot will be provided for methemoglobin.

A listing of all methemoglobin and oxygen saturation results, including replicates, will be presented. Any results considered abnormal or clinically significant by the investigator will be flagged.

10.6 Vital Signs

For the summary tabulations of blood pressure, pulse, temperature, and respiratory rate on Day 1, baseline is defined as the pre-dose value; pre-dose baseline will not be imputed. Pre-dose, post-dose (target 2 hours post-dose for seated and standing blood pressure and pulse and hourly for at least 4 hours for temperature and respiratory rate) and change in each parameter will be summarized using descriptive statistics. Change in blood pressure and pulse upon standing will also be summarized.

Similar summaries will be generated for blood pressure and temperature in the subset of subjects identified as concomitantly taking the following pharmacological subgroup of serotonergic drugs on Day 1: SSRIs, SNRIs, or MAOIs.

Vital sign measurements subsequent to Day 1 will be summarized descriptively by target visit using the visit windows defined in Appendix A and the last available on-treatment visit; Baseline will be the last non-missing value prior to the first dose of study drug. These include seated blood pressure and pulse, temperature, respiratory rate, and body weight. Change from Baseline will also be calculated and summarized. If a subject has multiple values within a visit window, the “worst” value as defined in Appendix E will be used for that visit window summary.

Potentially clinically significant vital sign changes are defined for selected parameters in Appendix C. When there are thresholds provided for low and high values, they will be handled separately. Summaries will include the number and percent of subjects with treatment-emergent PCS values, restricted to those subjects in whom the values represent a post-Baseline worsening (PCST). A PCST is defined as any PCS event that happened at any post-Baseline visit and has a value more out of range than the value for a particular parameter at Baseline (see Appendix E for directionality of worsening).

Listings of vital sign measurements including height at Screening will be presented. Listings will flag results that meet criteria as being PCS (whether or not a treatment emergent worsening).

Separate listings for each vital sign parameter will include only subjects with treatment-emergent PCS values and, for these subjects, all corresponding vital sign results will be provided.

10.7 Physical and Neurological Examination

The results of complete physical and neurological examinations performed at screening will be summarized by body system. Evaluations based on the targeted examinations performed at subsequent visits will be summarized in a treatment-emergent fashion. For the physical examination, each body system is assessed as Normal or Abnormal, and if Abnormal, then Clinically Significant or Not Clinically Significant. For the neurological examination, each body system is assessed as Normal, Abnormal or Absent, and if Abnormal, then Clinically Significant or Not Clinically Significant.

Summaries will present the number and percentage of subjects within each category by visit and by body system/parameter evaluated. By subject listings will detail the abnormality(ies).

10.8 Electrocardiogram

The central ECG data, provided by the central ECG reader (BioClinica), is the primary source of ECG data for purposes of tabular summarization. The ECG Data Transfer Plan provides a detailed description of the content and format of the central ECG dataset. ECG raw data include heart rate [HR], PR, RR, QRS, QT, and corrected QT intervals using Bazett's formula [QTcB] and Fridericia's formula [QTcF]).

There are two additional sources of ECG data, the ECG (local read) CRF and the ECG (Central read) CRF. The ECG (local read) was to be used on Day 1 pre-dose in triplicate to confirm eligibility for dosing and at other times including Screening, as deemed medically necessary by the Investigator or recommended by the protocol. The ECG (central read) CRF is for the investigator to provide a clinical interpretation of whether or not an abnormality noted by the central cardiology reader is clinically significant or not.

For the summary tabulations of ECG interval data and heart rate on Day 1, baseline is the pre-dose value; pre-dose baseline will not be imputed. Pre-dose, post dose (target 3 hours post dose) and change from pre-dose in each parameter will be summarized using

descriptive statistics. Measurements are to be made in triplicate (within an approximate 2- to 5-minute interval) and the summaries will be based on the average of the three.

Subsequently, 12-lead ECGs are to be obtained at every post-Baseline visit (or upon early termination), including the 4-week post-treatment follow-up visit (Visit 10); these ECG measurements are to be obtained as a single recording unless the subject has atrial fibrillation controlled by an anticoagulant (in which case triplicate recordings must continue). ECG interval and heart rate data subsequent to Day 1 will be summarized descriptively by target visit using the visit windows defined in Appendix A; Baseline will be the mean of the last non-missing triplicate values prior to the first dose of study drug). For subjects with triplicate measurements, the average for a given target window will be used. For each parameter, if a subject has multiple values (or averaged values in the case of a triplicate recording) within a visit window, the "worst" value as defined in Appendix E will be used for that visit window summary.

Summaries of ECG data will also be presented by gender.

The numbers and proportions (%) of subjects with abnormal ECGs identified by the central cardiology read and those that are determined to be clinically significant by the Investigator, will be summarized for Baseline, on-treatment target visit, and the follow-up visit.

Subjects are also to be categorized and enumerated on the basis of QTc intervals and change from Baseline as follows:

- QTcB outliers (> 450 to ≤ 480, > 480 to ≤ 500, > 500 msec)
- QTcF outliers (> 450 to ≤ 480, > 480 to ≤ 500, > 500 msec)
- Change in QTcB outliers (> 30 to ≤ 60, > 60 to < 90, ≥ 90 msec)
- Change in QTcF outliers (> 30 to ≤ 60, > 60 to < 90, ≥ 90 msec)

Thresholds for potentially clinically significant ECG changes are defined for selected parameters in Appendix D (gender specific QTcF/QTcB; PR interval; and low and high heart rate). When there are thresholds provided for low and high values, they will be handled separately. Summaries will include the number and percent of subjects with treatment-emergent PCS values, restricted to those subjects in whom the values represent a post Baseline worsening (PCST). A PCST is defined as any PCS event that happened at any post-Baseline visit and has a value that is worse than the value for a particular test at Baseline (see Appendix E for directionality of worsening).

To further evaluate an effect on PR interval, the numbers and proportions (%) of subjects with pertinent abnormal ECGs identified by the central cardiology read will be summarized by standardized finding term, specifically left axis deviation and/or left or right bundle branch block, for Baseline and post-baseline visits. A supportive listing of the central cardiology read will also be provided. In cases where standardized finding terminology is not available, verbatim terms will be displayed.

Central ECG data will be listed chronologically within a subject, inclusive of individual and mean of triplicate values for interval data and heart rate. The listings will also include the central cardiology interpretations of abnormalities and overall interpretation and whether or not these are considered clinically significant by the investigator. Change from Baseline in Investigator's overall interpretation of central ECG data will be summarized.

The data from the ECG (local read) CRF from Screening and Day 1 will be separately listed. This will include the site's recording of the date and time of the ECGs as well as ventricular rate and QTcF intervals (entered as triplicate results and the mean of the triplicate recordings) at Screening and Day 1 post-dose only, and the Investigator's interpretation of whether or not abnormal and, if abnormal, whether or not it is clinically significant. Screening and Day 1 post-dose ECGs will also include the description of any abnormalities and whether or not the subject meets study entry criteria (if before the first dose of study drug) or protocol withdrawal criteria (if after the first dose of study drug). The descriptions of abnormal ECGs may include comments such as notations of whether or not the ECG was suitable for QT analysis or ECG is not measurable, no matter if this ECG is suitable for QT analysis.

10.9 MRI Data (BioClinica)

ARIA cases are those determined from blinded medical review of MRI data and, in the case of fewer than ≤4 new cerebral microhemorrhages, symptomology to meet the protocol specified definition. These confirmed ARIA cases are captured as adverse events of special interest, discussed in Section 10.2.1.

With respect to the centrally read MRI data, the distribution of the number of microhemorrhages newly identified relative to the baseline will be tabulated by treatment group and visit, as well as for all post-baseline visits combined. Additionally, to investigate the occurrence of microhemorrhages further, number of subjects falling into the following four classes will be presented: (a) not present at baseline and not present afterwards, (b) not present at baseline but present afterwards, (c) present at baseline and present afterwards, and (d) present at baseline and new microhemorrhages post baseline. The summaries will be presented in absolute numbers as well as rates per treatment group, n (%). Furthermore, the two groups, (b) and (d), are deemed to be particularly relevant and will be compared to

typical literature placebo rates, if feasible. The MRI dataset to be used for these analyses is named ARIASTAT, with STATUS='New' indicating presence of new ARIA. Individual microhemorrhages are indicated by MB1, MB2, etc. in the LABEL variable, allowing the number of new microhemorrhages relative to Baseline to be counted for each subject at each visit.

This information as reported to the site will be included in a listing. All MRI scan data will be included in the database and included in a listing.

10.10 Serotonin Toxicity (Syndrome)

Ratings from the Serotonin Toxicity (Syndrome) Diagnostic Interview and Rating Guide (In-Clinic) and, in those subjects in whom it is applicable, the Serotonin Toxicity Telephone Assessment, will be listed.

The total score for each of the four possible diagnostic criteria will be confirmed programmatically (see the Serotonin Syndrome Diagnosis Sheet in the study protocol for a description of the scoring) (if available) and provided together with the site entered score. The listing will also include the total scores for each of the four possible diagnostic criteria.

10.11 Columbia-Suicide Severity Rating Scale (C-SSRS)

Results of the C-SSRS will be evaluated and reported according to the Columbia Classification Algorithm of Suicide Assessment (C-CASA). The following outcomes are C-SSRS categories and have binary responses (yes/no) (Nilsson, *et al.* 2013). The categories have been re-ordered from the actual scale to facilitate the definitions of the composite and comparative endpoints, and to enable clarity in the presentation of the results.

Category 1 – Wish to be Dead
Category 2 – Non-specific Active Suicidal Thoughts
Category 3 – Active Suicidal Ideation with Any Methods (Not Plan) without Intent to Act
Category 4 – Active Suicidal Ideation with Some Intent to Act, without Specific Plan
Category 5 – Active Suicidal Ideation with Specific Plan and Intent
Category 6 – Preparatory Acts or Behavior
Category 7 – Aborted Attempt
Category 8 – Interrupted Attempt
Category 9 – Actual Attempt (non-fatal)
Category 10 – Completed Suicide

Composite endpoints based on the above categories are defined below and will be summarized.

Suicidal ideation: A “yes” answer at any time during treatment to any one of the five suicidal ideation questions (Categories 1-5) on the C-SSRS.

Suicidal behavior: A “yes” answer at any time during treatment to any one of the five suicidal behavior questions (Categories 6-10) on the C-SSRS.

Suicidal ideation or behavior: A “yes” answer at any time during treatment to any one of the ten suicidal ideation and behavior questions (Categories 1-10) on the C-SSRS.

While not part of C-CASA, the C-SSRS also includes two pertinent questions that will also be summarized:

- Self-injurious behavior without suicidal intent; and
- Suicidal behavior (separate question from that used in the C-CASA).

Fisher’s exact test will be used to compare the LMTM treatment group to the placebo group restricted to the number of subjects with any suicidal ideation or behavior. All recorded information will be included in listings.

A table will be provided that summarizes the Reports of Suicidal Thoughts elicited by the C-SSRS and/or spontaneously reported as Adverse Events.

11. CHANGES TO PROTOCOL-SPECIFIED ANALYSES

Changes are implemented to enhance the findings of the study as follows.

- Based on the results of a companion Phase 3 study, TRx-237-015, the treatment comparisons have been revised.
- Based on a correspondence with the EMA, the primary analysis population for the EMA will be the ITT population.
- Based on recommendations of an MRI expert panel, the Ventricular Volume (VV), which is less sensitive to confounding hydration effects and covers partly temporoparietal areas and therefore of particular interest in AD pathologies, replaces the whole brain volume (WBV) as the primary imaging parameter; with WBV utilized for investigating the impact of treatment on overall rate of whole brain atrophy and therefore used as a confirmatory imagining parameter.

Any additional changes will be documented by amendment of this document. Should additional analyses be required in response to data review, they will be noted in the Clinical Study Report in Section 9.8.

12. REFERENCES

1. Aisen PS, Schafer KA, Grundman M, *et al*. Alzheimer's Disease Cooperative Study. Effects of rofecoxib or naproxen vs placebo on Alzheimer disease progression: a randomized controlled trial. JAMA 2003; 289(21):2819-26.
2. Aisen PS, Schneider LS, Sano M, *et al.* Alzheimer Disease Cooperative Study. High-dose B vitamin supplementation and cognitive decline in Alzheimer disease: a randomized controlled trial. JAMA 2008; 300(15):1774-83.
3. Alzhermier’s Cooperative Study, Administration and Scoring Manual Alzheimer’s Disease Assessment Scale – Cognitive (ADAS-cog), 10 March 2012.
4. Birks, J. (2006) Cholinesterase inhibitors for Alzheimer's disease. Cochrane Database of Systematic Reviews Issue 1, Article No. CD005593.
5. C-Path Online Data Repository, Tucson, AZ, Critical Path Institute, 2010.
6. Gold M. Study design factors and patient demographics and their effect on the decline of placebo-treated subjects in randomized clinical trials in Alzheimer's disease. J Clin Psychiatry. 2015; 68(3):430-8.
7. Great Britain: Office of Population Censuses and Surveys. Standard occupational classiffication. London: HMSO; 1990.
8. Kahle-Wrobleski K, Coley N, Lepage B, et al. Understanding the Complexities of Functional Ability in Alzheimer’s Disease: More Than Just Basic and Instrumental Factors. Current Alzheimer Research. 2014, 11:357-66.
9. Nilsson M, Suryawanshi S, Gassmann-Mayer C, Dubrava S, McSorley P, Jiang K, et al. Columbia–Suicide Severity Rating Scale Scoring and Data Analysis Guide, 2013.
10. Salloway et al., Clinical Trials in Alzheimer’s Disease Conference, 29 October 2012
11. Sano M, Ernesto C, Thomas RG, *et al*. A controlled trial of selegiline, alpha-tocopherol, or both as treatment for Alzheimer's disease. The Alzheimer's Disease Cooperative Study. N Engl J Med 1997; 336(17):1216-22.
12. Seimers and Ricks, Boston, MA, USA 8 October 2012
13. Wilcock GK, Black SE, Hendrix SB, *et al.* Tarenflurbil Phase II Study investigators. Efficacy and safety of tarenflurbil in mild Alzheimer's disease: a randomised phase II trial. Lancet Neurol 2008; 7(6):483-93. Erratum in: Lancet Neurol 2008; 7(7):575

APPENDICES

Appendix A: Post-Baseline Visit Windows

The visit windows do not apply to the off-treatment visits, which are always assigned as follow-up, regardless of when they occur. Unscheduled and early termination visits are included in the windowing algorithm. Off-treatment visits are defined as visits that occur after the last dose date +14 days, follow-up visits will be treated as-off treatment visits irrespective of this rule.

Visit windows for safety assessments (laboratory tests, pulse co-oximetry, vital signs, and ECG) are defined in the following table:

Window Label	Time Interval (Study Day*)	Scheduled Day
Day 1 Post-Dose	1	1
Week 2	2 to 28	15
Week 6	29 to 67	43
Week 13	68 to 137	92
Week 26	138 to 228	183
Week 39	229 to 319	274
Week 52	320 to 410	365
Week 65	411 to 501	456
Week 78	>501	547

*calculated as in Section 5.2.

Visit windows for efficacy assessments (ADAS-cog_{11}, ADCS-CGIC, ADCS-ADL_{23}, MRI, FDG-PET, NPI, MADRS and MMSE) are defined in the following table:

Window Label	Time Interval (Study Day*)	Scheduled Day
Pre-Dose**	1	1
Unscheduled***	1 to 67	NA
Week 13	68 to 137	92
Week 26	138 to 228	183
Week 39	229 to 319	274
Week 52	320 to 410	365
Week 65	411 to 501	456
Week 78	502 to 592	547

*calculated as in Section 5.2.
** Pre-Dose includes values on Day 1 but prior to the first dose.

*** Unscheduled includes Post-Dose Day 1 value and beyond up to day 67.

Appendix B: Criteria for Determining Potentially Clinically Significant Values in Laboratory Test Results

Test	Criteria - System International Units	Criteria – Conventional Units
Hemoglobin	Female: ≤ 95 g/L;<br>Male: ≤ 115 g/L<br>Decrease of ≥ 20%	Female: ≤ 9.5 g/dL;<br>Male: ≤ 11.5 g/dL<br>Decrease of ≥ 20%
Hematocrit	Female: ≤ 0.32; Male: ≤ 0.37	Female: ≤ 32%;<br>Male: ≤ 37%
WBC count	≤ 2.8 × 10^9/L<br>≥ 16 × 10^9/L	≤ 2800/µL or ≥ 1600/µL
Neutrophils	≤ 1.0 × 10^9/L	≤ 1000/µL
Eosinophils	≥ 0.7 × 10^9/L	≥ 700/µL
Platelet count	≤ 75 × 10^9/L<br>≥ 700 × 10^9/L	≤ 75 × 10^3/µL<br>≥ 700 × 10^3/µL
Sodium	<130 mmol/L<br>>150 mmol/L	<130 mEq/L<br>>150 mEq/L
Potassium	< 3.0 mmol/L<br>> 5.5 mmol/L	< 3.0 mEq/L<br>> 5.5 mEq/L
Calcium	< 1.75 mmol/L<br>> 3.00 mmol/L	< 7.00 mg/dL<br>> 12.00 mg/dL
Triglycerides, fasting/nonfasting/unknown	> 3.39 mmol/L	> 300 mg/dL
Glucose, fasting/nonfasting/unknown	< 2.775 mmol/L<br>>13.875 mmol/L	< 50 mg/dL<br>> 250 mg/dL
Uric acid	Female: >475.8 µmol/L; Male: >594.8 µmol/L	Female: > 8 mg/dL;<br>Male: >10 mg/dL
Albumin	<25 g/L	<2.5 g/dL
Total bilirubin	≥ 34.2 µmol/L	≥ 2 mg/dL
ALT	≥ 3 × ULN	≥ 3 × ULN
AST	≥ 3 × ULN	≥ 3 × ULN
Alkaline phosphatase	≥ 3 × ULN	≥ 3 × ULN
TpI	>ULN	>ULN
Methemoglobin	>3.5%	>3.5%
Urea	> 17.85 mmol/L	> 50 mg/dL
Creatinine	≥ 177 µmol/L	≥ 2 mg/dL

Test	Criteria - System International Units	Criteria – Conventional Units
Cholesterol	>10.36 mmol/L	> 400 mg/dL
CK	≥ 3 × ULN	≥ 3 × ULN
GGT	≥ 3 × ULN	≥ 3 × ULN
LDH	≥ 3 × ULN	≥ 3 × ULN
Phosphorus	< 0.646 mmol/L<br>> 1.777 mmol/L	Low: <2.0 mg/dL<br>High: >5.5 mg/dL
Urinalysis – Protein	Increase of ≥ 2 units	Increase of ≥ 2 units
Urinalysis – Glucose	Increase of ≥ 2 units	Increase of ≥ 2 units
Urinalysis – Blood	Increase of ≥ 2 units	Increase of ≥ 2 units
Urinalysis – Bilirubin	Increase of ≥ 2 units	Increase of ≥ 2 units

ULN = Upper Limit of Normal

Appendix C: Criteria for Determining Potentially Clinically Significant Values in Vital Signs

Test	Criteria
Systolic Blood Pressure (SBP) – Seated (mmHg)	Increase of ≥ 20 mmHg from baseline and ≥ 180 mmHg
	≤ 90 mmHg
	Decrease of ≥ 20 mmHg from baseline and ≤ 90 mmHg
Diastolic Blood Pressure (DBP) – Seated (mmHg)	Increase of ≥ 15 mmHg from baseline and ≥ 105 mmHg
	≤50 mmHg
Pulse – Seated (beats/min)	Increase of ≥ 15 beats/min from baseline and > 120 beats/min
	Decrease of ≥ 15 beats/min from baseline and ≤ 50 beats/min
	Increase of ≥ 15 beats/min from baseline and ≥ 120 beats/min
Orthostatic Change	Decrease upon standing in SBP of ≥ 20 mmHg and ≥ 10 mmHg in DBP
Temperature	Increase of ≥ 2.0^{o}C from baseline and ≥ 38.0^{o}C
	Decrease of ≥ 2.0^{o}C from baseline and ≤ 36.0^{o}C
Weight	Decrease of ≥ 7% from baseline<br>Decrease of ≥ 10% from baseline
	Increase of ≥ 7% from baseline<br>Increase of ≥ 10% from baseline

Appendix D: Criteria for Identifying ECG Values that Reflect Potentially Clinically Significant Change

Parameter	Criterion
QTcF/QTcB	>470 msec (females), >450 msec (males)
PR Interval	>200 msec
Heart Rate	<48 beats per minute<br>>96 beats per minute

Appendix E: Rules for Determining "Worst" Value

"Worst" Clinical Laboratory Value

Rule	Parameters
Highest value	Hematology: eosinophils, basophils, monocytes, methemoglobin, reticulocytes
	Serum chemistry: ALT, AST, ALK-P, creatinine, total bilirubin, urea (nitrogen), uric acid, LDH, TSH, creatine kinase, GGT, triglycerides, cholesterol (total)
	Urinalysis: all categorical results
Lowest value	Hematology: neutrophils, RBC count, HCT, hemoglobin, platelets
	Serum chemistry: albumin, creatinine clearance, total protein, oxygen saturation
Farthest from normal range midpoint	Hematology: WBC count, lymphocytes, MCV, MCH, MCHC
	Serum chemistry: glucose (random), Na, K, phosphate, TBG, T4, calcium, chloride
	Urinalysis: specific gravity, pH

"Worst" Vital Sign and Weight Measurement

Parameter	Criterion for "Worst" Vital Sign
Systolic blood pressure	Value farthest from 125 mmHg
Diastolic blood pressure	Value farthest from 75 mmHg
Pulse	Value farthest from 75 beats per minute
Respiratory Rate	Highest
Weight	Greatest weight loss from baseline

"Worst" ECG Measurement

Parameter	Criterion for "Worst" ECG
PR	Highest
QT (QTcF and QTcB)	Highest
QRS	Value farthest from midpoint of normal range
Heart Rate	Value farthest from midpoint of normal range

"Worst" Efficacy Measurement

Parameter	Criterion for "Worst" Efficacy Measurement
ADAS-cog$_{11}$	Highest
ADCS-CGIC	Highest
ADCS- ADL$_{23}$	Lowest
MMSE	Lowest
NPI	Highest
MADRS	Highest
Glucose uptake by FDG-PET of the temporal lobes	Lowest
Whole brain volume by MRI	Lowest
Ventricular volume by MRI	Highest
Hippocampal volume by MRI	Lowest
total tau	Highest
phospho-tau	Highest
Aβ1−42	Highest

Appendix F: Rules for Scoring the Disease Assessment Scales

1.ADAS-cog$_{11}$

The ADAS-cog11 total score is the sum of four domain scores, and ranges from 0 to 70. The domain scores are the sum of item subscores within each domain, as listed in the following table.

Domain	Subscore	Maximum Subscore
Memory	Word Recall	10
	Word Recoginition	12
	Remembering Test Instructions	5
Praxis	Constructional Praxis	5
	Ideational Praxis	5
Orientation	Orientation	8
Language	Naming Objects and Fingers	5
	Following Commands	5
	Spoken Language Ability	5
	Word-Finding Difficulty	5
	Comprehension	5

The Word Recall subscore will be calculated as the mean of the non-missing scores from the three trials, rounded to 2 decimal places. The other 10 subscores are obtained directly from the CRF page.

Within a given domain, there may be some missing subscores. If the sum of the maximum possible scores of the non-missing items is greater or equal to one half (50%) of the maximum possible score for the domain, the domain score will be scaled up to be out of the maximum possible score for the domain, as calculated in the following formula,

$$\frac{\text{Sum of Non-missing Subscores}}{\text{Sum of Maximum Possible Score of Each Non-missing Item}} \times (\text{Maximum Possible Domain Score})$$

Otherwise, the domain score will be missing. If any domain score is missing, the ADAS-cog$_{11}$ total score will be missing.

2. ADCS-ADL$_{23}$

The ADCS-ADL$_{23}$ total score ranges from 0 to 78, and is the sum of 23 items as listed in the following table.

Item Number	Maximum Score	Item Number	Maximum Score	Item Number	Maximum Score
1	3	9	3	17	3
2	3	10	3	18	3
3	3	11	3	19	3
4	3	12	3	20	2
5	3	13	4	21	3
6	7	14	3	22	3
7	5	15	4	23	4
8	3	16	4	Total	78

Some of the item scores (questions 16 and 20) are simple sums of sub-item scores[2]. If a sub-item is missing for these two items, the maximum score for the item is reduced correspondingly.

Some of the item scores (questions 8, 18 and 19) consist of a leading Yes/No/Don't Know question, which, if answered "No" or "Don't Know", imply that the sub-item scores should be treated as non-missing and equal to 0. If a sub-item is still missing, the maximum score for the item is reduced correspondingly.

If any other item is missing, the maximum score for the item is set to 0.

After these rules have been applied, let *m* be the maximum obtainable score, which might be less than 78. If m is equal to 78, the ADCS-ADL score is the sum of the item scores.

[2] In non-English-language workbooks, Question 20 is presented as a leading question followed by two sub-items, whereas in other workbooks it is presented simply as two sub-items. In all cases, Question 20 is stored in the clinical database as two separate subitems, and so the rule for "simple sub-item scores" applies.

Otherwise, let s denote the sum of the scores of non-missing items. If $m/78 \geq 2/3$, ADCS-ADL_{23} total score = $78 \times s/m$. Otherwise ADCS-ADL_{23} total score will be missing.

Since 19c was missing initially in English-speaking regions' questionnaires, to calculate the total score, two methods will be employed as described below:

1. Apply the scale-up method exactly as described above. This is the method to be used in the primary analysis.
2. Set 19c to missing for all assessments, and use the scale-up method described above to obtain the total score. This is to be used for a sensitivity analysis.

3. MMSE

The MMSE score ranges from 0 to 30, and is defined as the sum of 11 items as listed in the following table.

**Item**	**Maximum Score**
Orientation – Time (including questions about year, season, month, week, date)	5
Orientation – Place (including questions about state, county, city/town, building and floor)	5
Memory – Registration (including registration word 1-3)	3
Attention and Concentration (including five "what is 100 taken away 7" questions)	5
Memory – Recall (including questions of "Recall Word" 1-3)	3
Language – Naming (including questions about pencil/pen and watch)	2
Language – Repetition ("Repeat what I say")	1
Language – Reading Comprehension ("Close your eyes")	1
Praxis – Ideational (Including questions of "Take in right hand" "Fold in Half" "Put on Floor").	3
Language – Writing Spontaneous ("Please Write a Sentence")	1
Praxis – Coping Drawing ("Please copy this design")	1
Total	30

When some of the item subscores are missing, the following algorithm will be used to calculate the MMSE score: Let s denote the sum of the scores of non-missing items, and m

denote the sum of maximum possible scores of the non-missing items. If $m/30 \geq 2/3$, then MMSE score = $30 \times s/m$. Otherwise the MMSE score will be missing. Some of the item scores are simple sums of sub-item scores. If a sub-item is missing for these items, the maximum score for the item is reduced correspondingly.

4.MADRS

The MADRS score ranges from 0 to 60, and is defined as the sum of 10 items as listed in the following table.

**Item (0: best; 6: worst)**	**Range of Score**
1. Apparent Sadness	0 - 6
2. Reported Sadness	0 - 6
3. Inner Tension	0 - 6
4. Reduced Sleep	0 - 6
5. Reduced Appetite	0 - 6
6. Concentration Difficulties	0 - 6
7. Lassitude	0 - 6
8. Inability to Feel	0 - 6
9. Pessimistic Thoughts	0 - 6
10. Suicicdal Thoughts	0 - 6
Total Score	0 - 60

When some of the item scores are missing, the following algorithm will be used to calculate the MADRS total score: Let s denote the sum of the scores of non-missing items, and m denote the sum of maximum possible scores of the non-missing items. If number of non-missing items ≥ 7 (2/3 of 10),, then MADRS total score = $60 \times s/m$. Otherwise the MADRS total score will be missing.

5. NPI

The NPI total score ranges from 0 to 144 and is defined as the sum of frequency x severity of 12 items as listed in the following table. Caregiver distress score ranges from 0 to 60.

Frequency is rated as:
1. Rarely – less than once per week
2. Sometimes – about once per week
3. Often – several times per week but less than every day
4. Very often – once or more per day

Severity is rated as:
1. Mild – produces little distress in the patient
2. Moderate – more disturbing to the patient but can be redirected by the caregiver
3. Severe – very disturbing to the patient and difficult to redirect

The score for each domain is: domain score = frequency x severity

Distress is scored as:
0. Not at all
1. Minimally (almost no change in work routine)
2. Mildly (some change in work routine but little time rebudgeting required)
3. Moderately (disrupts work routine, requires time rebudgeting)
4. Severely (disruptive, upsetting to staff and other residents, major time infringement)
5. Very Severely or Extremely (very disruptive, major source of distress for staff and other residents, requires time usually devoted to other residents or activities)

Thus, for each behavioral domain there are four scores:
• Frequency
• Severity
• Frequency x severity
• Caregiver distress

**Domain**	**Absent**	**Freq**	**Severity**	**Freq x Severity**	**Distress**
A. Delusions	0	1-4	1-3	0-12	0-5
B. Hallucinations	0	1-4	1-3	0-12	0-5
C. Agitation/Aggression	0	1-4	1-3	0-12	0-5
D. Depression/Dysphoria	0	1-4	1-3	0-12	0-5
E. Anxiety	0	1-4	1-3	0-12	0-5
F. Elation/Euphoria	0	1-4	1-3	0-12	0-5

Domain	Absent	Freq	Severity	Freq x Severity	Distress
G. Apathy/Indifference	0	1-4	1-3	0-12	0-5
H. Disinhibition	0	1-4	1-3	0-12	0-5
I. Irritability/Lability	0	1-4	1-3	0-12	0-5
J. Abberrant Motor Behavior	0	1-4	1-3	0-12	0-5
K. Sleep and Nighttime Bahvior Disorder	0	1-4	1-3	0-12	0-5
L. Appetite and Eating Changes	0	1-4	1-3	0-12	0-5
Total Score				0-144	0-60

‘N/A’ should be treated as missing value. If the response to the gateway question for a domain is ‘No’, then the domain score and domain caregiver distress score are set to zero for that domain. If number of non-missing domain scores (frequency x severity) ≥ 8 (2/3 of 12), then NPI total score = $144\times s/m$, where s denotes the sum of the non-missing domain scores and m denotes the sum of maximum possible non-missing domain scores. Otherwise the NPI total score will be missing. Distress score is derived in the same way except that distress score = $60\times s/m$.

Appendix G: List of Review Files Provided by TauRx to SynteractHCR

1. Excel file indicating medications indicating serotonergic potential. File to include ATCCODE and PREFERRED NAME – currently receiving as an update in advance of each DSMB meeting.
2. Protocol deviations – identified by subject and a flag per each deviation type
3. AE groupings – identified via unique SOC and preferred term
4. AEs confirmed by TauRx to be an ARIA case – subject identification, AE verbatim and appropriate ARIA categorization to be provided
5. Medications identified as medical food for Alzheimer's or alternative pharmacotherapy for dementia (used for efficacy subgrouping)
6. Medications: Anti-dementia (Synteract will provide a medication list including ATCTEXT3, ATCTEXT4, ATC4, PRENAME, DRUGNAME for only 2 Who codes: N06DA and N06DX. In addition, a similar list will be provided when ATCTEXT4= ANTICHOLINESTERASES to select for AChEI, and PREFNAME=MEMANTINE or PREFNAME=MEMANTINE HYDROCHLORIDE to select for memantine.)

Statistical Analysis Plan (Addendum)

TauRx Therapeutics Ltd

TRx-237-005

Randomized, Double-Blind, Placebo-Controlled, Parallel-Group, 18-Month Safety and Efficacy Study of Leuco-methylthioninium bis(hydromethanesulfonate) in Subjects with Mild Alzheimer's Disease

VERSION 1.0 DATED 04 July 2016

Sponsor: **TauRx Therapeutics Ltd**
Liberty Building,
Foresterhill Road,
Aberdeen AB25 2ZP,
Scotland, UK

Prepared by: **Paul Pappas**
SynteractHCR, Inc.
5759 Fleet Street, Suite 100
Carlsbad, CA 92008

Approval

Upon review, the undersigned approves the Statistical Analysis Plan Addendum.

Signature	Date
Professor Claude Wischik<br>Executive Chairman<br>TauRx Therapeutics Ltd.	4 July 2016
Dr. Jiri Hardlund, CMO<br>VP Clinical Development<br>TauRx Therapeutics Ltd.	04 JULY 2016
Charles Davis, Ph.D.<br>Biostatistics Consultant<br>CSD Biostatistics, Inc.	04 July 2016
Paul Pappas<br>Senior Lead Biostatistician<br>Synteract	04July2016

This addendum clarifies certain analyses described in the body of the SAP.

9.1 Primary Efficacy Analyses

For the purposes of this note, it is useful to define some subgroups. First, define *achmem=NO* to mean those subjects who were stratified as not receiving AChEI and/or Memantine at baseline, and *achmem=YES* to mean all other subjects. Now, define four groups of subjects: (I) those randomized to 200mg/day with achmem=NO, (II) those randomized to 8mg/day with achmem=NO, (III) those randomized to 8mg/day with achmem=YES, (IV) those randomized to 200mg/day with achmem=YES.

The SAP specifies *"The two treatment comparisons will be investigated in a parallel implementation of sequential tests. Comparison A (200mg/day given alone) will test null hypotheses H01 and H02, Comparison B (8mg given alone) will test H03 and H04. [...] The individual tests will be implemented through contrast statements based on the repeated measures linear mixed model."*

The contrast used for testing H01 will report the difference between the estimated response in group (I), and the averaged estimated response in groups (II) and (III). This averaging will be based on the number of on-treatment ADAScog observations at Visit 10 in those two groups.

The contrast used for testing H02 will be similar, but based on ADCS-ADL observations at Visit 10 rather than ADAScog.

The SAP also calls for ITT versions of the tests, and specifies *"For potentially treatment related drop-outs, the fraction of subjects randomized to active treatment who withdrew at a given time point is calculated. [...] patients who withdraw for potential treatment-related reasons are assumed to retain 100% of the treatment effect they had attained up to the point of withdrawal but do not continue to benefit from treatment afterwards."*

The contrast used for the ITT versions of H01 and H02 will be as follows. The fraction of subjects withdrawing at each time point is calculated for group (I). The treatment effect at each time point corresponds to the difference between the response in group (I) and the averaged responses for groups (II) and (III). This averaging will be based on the number of subjects in those two groups at baseline.

The contrast used for the ITT versions of H03 and H04 will be as follows. The fraction of subjects withdrawing at each time point is calculated for group (II). This overrides the text in the SAP referring to "*the fraction of subjects randomized to active treatment*". The

treatment effect at each time point corresponds to the difference between the response in group (II) and that in group (III).

9.2 Secondary efficacy analyses

The contrasts for Ventricular Volume, ADCS-CGIC as a numerical score, ADCS-CGIC as a modridit score, MMSE, MADRS, and NPI, will all use contrasts corresponding to those described for the primary analysis.

9.5 Additional analyses

In addition to the contrasts defined for the primary MITT analysis, three further contrasts will be reported: (I) versus (III), (IV) versus the average for (II) and (III), and (IV) versus (III).
